# Purine metabolism: a pan-cancer metabolic dysregulation across circulation and tissues

**DOI:** 10.1186/s12943-025-02482-9

**Published:** 2025-10-14

**Authors:** Mengjie Yu, Cheng Liu, Minmin Cao, Dou Yang, Tongshan Wang, Jing Xu, Danxia Zhu, Guangji Wang, Jiye Aa, Wei Zhu

**Affiliations:** 1 State Key Laboratory of Natural Medicines, China Pharmaceutical University, Nanjing, Jiangsu 210009 P.R. China; 2 Department of Gastroenterology, Nanjing Drum Tower Hospital Clinical College of Nanjing Medical University, 321 Zhongshan Road, Nanjing, Jiangsu 210008 P.R. China; 3 Department of Oncology, The Jiangyin Hospital Affiliated to Medical College of Southeast University, Jiangyin, Jiangsu 214400 P.R. China; 4 Department of Oncology, The First Affiliated Hospital with Nanjing Medical University, Guangzhou Road, Nanjing, Jiangsu 210029 China; 5 Department of Oncology, The Third Affiliated Hospital of Soochow University, 185 Juqian Road, Changzhou, Jiangsu 213000 P.R. China

**Keywords:** Pan-cancer, Circulation, Metabolomics, Purine metabolism, Hypoxanthine

## Abstract

Tumors function as organ-like entities within complex ecosystems, interacting with diverse components of their microenvironment, including blood and lymphatic vessels, neurons, immune cells, metabolites, and cytokines, to drive tumorigenesis and progression. Our pan-cancer study investigated the universal tumor hallmarks, integrating metabolite characteristics with molecular mechanisms. Metabolomic profiling on plasma from 2,561 patients across 20 cancer types and 604 healthy controls in two clinical centers, identified three biomarkers in pan cancers: elevated levels of hypoxanthine and reduced levels of cysteine and pyruvic acid. Given the profound significance of hypoxanthine, we further discovered 33 core purine metabolism-related genes in The Cancer Genome Atlas (TCGA) pan-cancer tissues, and their influences on immunomodulation and overall survival. Lastly, candidate therapeutic compounds, intervening purine metabolism, were proposed based on pharmaco-transcriptomics and pharmaco-proteomics analysis. Through interdisciplinary multi-omics investigations, such approaches may enhance insight into antitumor immunotherapy by targeting cancer metabolic reprogramming.

**Supplementary Information:**

The online version contains supplementary material available at 10.1186/s12943-025-02482-9.

## Introduction

Contrary to the conventional paradigm of genetic mutation-driven oncogenic progression [1], contemporary interdisciplinary studies (e.g., system biology [2], mechanobiology [3], and quantum biology [4]), have established a multi-dimensional comprehension of cancer as a complex systemic disorder [5]. As a methodological approach for exploring the tumorigenesis dynamics, pan-cancer analysis can reveal conserved mechanisms by which diverse cancer cells disrupt the homeostatic balance of the organism through a multi-scale perspective spanning molecular, cellular, and organ-level dimensions [6–9].

Positioned downstream within the genome-transcriptome-proteome-metabolome biological axis, metabolomics directly measures the dynamic alterations in pathological states within tumors, whereas transcriptomics reflects the regulatory framework controlling gene expression [10–15]. Integrating cancer tissue transcriptomics and circulating metabolomics could provide a comprehensive perspective of tumor and microenvironment co-evolution paths, enabling precise targeting of cancer metabolic reprogramming to enhance immunotherapy efficacy [16, 17].

To enhance our understanding of the role of metabolism in cancer, we systematically characterized the plasma metabolic pan-cancer landscape and developed a universal and high-accuracy diagnostic model specifically based on the combination of hypoxanthine, cysteine, and pyruvic acid using machine learning methodologies across 20 distinct cancer types. Given hypoxanthine exhibits the strongest predictive impact across cancers, with elevated levels observed in various cancer types, we identified 33 key gene signatures from a pool of 143 genes associated with purine synthesis in 17 cancer types using data from The Cancer Genome Atlas (TCGA), and validated protein expression using the Human Protein Atlas (HPA) and Clinical Proteomic Tumor Analysis Consortium (CPTAC) databases. Furthermore, we explored the interactions between purine metabolism-related genes and the tumor immune microenvironment (TIME), and identified potential inhibitors targeting purine metabolic reprogramming through pharmaco-multi-omics integration. Our study elucidates conserved pathogenic mechanisms across cancers via comprehensive metabolic profiling and subsequent tissue-level molecular analysis. The findings indicate that purine metabolism is intricately associated with tumor immunology, achieved through dual regulation of immune cell function and immune evasion mediated by a series of enzymatic reactions within the TIME.

## Methods

### Participants cohorts

Two distinct cohorts, each comprising individuals with pathologically confirmed cancer, were recruited for this study. Cohort 1 was sourced from the First Affiliated Hospital with Nanjing Medical University (Center 1) over the period from January 2016 to December 2022. This cohort consisted of 1,998 participants, including 1,646 cancer patients representing 20 different cancer types (median age of 63 years, with 51.28% male), alongside 352 healthy individuals (median age of 58 years, with 49.72% male). Cohort 2, designated for external validation purposes, was collected from the Jiangyin People’s Hospital (Center 2) between January 2019 and December 2022. This cohort included 1,167 participants, comprising 915 cancer patients across 5 cancer types (median age of 61 years, with 54.07% male) and 252 healthy individuals (median age of 50 years, with 54.32% male).

Participants exhibited no metabolic abnormalities (e.g., hypoproteinemia, weight loss, or negative nitrogen balance). Fasting blood samples were collected between 6:00–8:30 AM, stored at 4 °C, and cryopreserved at − 80 °C within 6 h post-plasma isolation. The study protocol (No. 2016-SRFA-149) was approved by the Ethics Committee of the First Affiliated Hospital with Nanjing Medical University, with informed consent obtained from all participants.

### Circulating metabolites analysis

Plasma samples were processed and derivatized as previously described [18]. Briefly, 50 µL plasma was mixed with 200 µL methanol containing 1,2-¹³C₂-myristic acid (5 µg/mL), vortex-mixed for 5 min, and centrifuged (20,000×g, 10 min, 4 °C). Supernatant (100 µL) was evaporated to dryness (Speed-Vac SC250EXP). The dried plasma samples were subjected to methoxylation by adding 30.0 µL of a 10.0 mg/mL methoxyamine pyridine solution to the residue, followed by incubation at room temperature for 16 h. Subsequently, the samples were trimethylsilylated for an additional 1.0 h by adding 30.0 µL of N-methyl-trimethylsilyltrifluoroacetamide (MSTFA) and 1% trimethylchlorosilane (TMCS) as a catalyst. Finally, external standard (30 µL heptane/methyl myristate, 15 µg/mL) was added for gas chromatography-mass spectrometry (GC-MS) stability assessment.

GC-MS analysis (Shimadzu GCMS-QP2010 Ultra, HP-5MS column) used split injection (8:1 ratio, 250 °C injector, He carrier gas at 1.5 mL/min). The oven program: 80 °C (3 min) → 300 °C (20 °C/min ramp, 5 min hold). MS parameters: 70 eV ionization, 3.2 mA current, 220 °C source/interface, full-scan mode (50–700 m/z), 19 min runtime. Quality control (QC) samples were prepared from the pooled plasma using the aforementioned preparation procedure. To minimize systematic variations, all samples were analyzed in a randomized order, with QC samples interspersed throughout the sequence.

Chromatograms were acquired, and peaks were deconvoluted using Shimadzu GC Postrun Analysis software. Peaks exhibiting a signal-to-noise (S/N) ratio below 10 were excluded. The retention index for each compound was calculated by comparing its retention time to those of a C8-C40 alkane series (generating retention indices between 800 and 4000). Compound identification was achieved by matching both mass spectra and retention indices against authentic reference standards and entries in the NIST and Wiley mass spectral libraries, as well as an in-house spectral library maintained by the State Key Laboratory of Natural Medicines, China Pharmaceutical University. Additionally, the Human Metabolome Database (HMDB; http://www.hmdb.ca) was queried to search for potential metabolites.

### Statistical analysis of circulating metabolomics

Metabolite abundances were normalized using 1,2-¹³C₂-myristic acid as an internal standard (IS). Peak areas were adjusted according to the formula: Normalized Area = (Metabolite Peak Area)/(IS Peak Area) × (Mean IS Area in QC Pool). Subsequently, probabilistic quotient normalization (PQN) was applied prior to statistical analysis [19]. Differences between groups were assessed using independent-samples t-tests (normal data) or Mann-Whitney U tests (non-normal data). Resulting *p*-values were adjusted via the Benjamini-Hochberg procedure to establish the false discovery rate (FDR), with statistical significance defined as FDR < 0.05. Principal component analysis (PCA) was conducted using SIMCA-P 14.1 (Sartorius) to evaluate group clustering and separation. Receiver operating characteristic (ROC) curve analysis and pathway mapping were performed with MetaboAnalyst 6.0, while visualizations were created using GraphPad Prism 8.0.

### Construction and evaluation of machine learning predictive model

A cancer diagnosis prediction model was constructed via a random forest classification algorithm. Hyperparameter optimization employed 15-fold cross-validation, yielding a final model incorporating 100 decision trees without maximum depth constraints. Minimum impurity split improvement threshold was set at 0.0, with the Gini index serving as the impurity criterion. Cohort 1 underwent stratified random sampling partitioned into training (85%) and validation (15%) subsets, while Cohort 2 data served for external validation. Performance metrics included area under the curve (AUC), accuracy, F1-score, Kappa-value, positive predictive value (PPV), negative predictive value (NPV), sensitivity, and specificity. All analyses were conducted using R 4.0, Python 3.7, and the Extreme Smart Analysis platform (https://www.xsmartanalysis.com/).

### Derivation of gene signatures in a pan-cancer dysregulated metabolic pathway across tissues

Hypoxanthine, formed by degradation of inosine monophosphate (IMP) in the de novo purine biosynthesis pathway, showed markedly upregulation and strongest predictive performance in our study. Therefore, we further conducted the transcriptome analysis of purine metabolism in pan cancers tissues. Totally 143 purine metabolism-related genes (PMRGs) were listed in metabolic atlas (https://metabolicatlas.org/). We obtained high throughput sequencing (HTS) gene expression data of 17 cancer types and normal tissues adjacent to the tumors from TCGA, including bladder urothelial carcinoma (BLCA), breast carcinoma (BRCA), cervical squamous cell carcinoma and endocervical adenocarcinoma (CESC), cholangiocarcinoma (CHOL), colon adenocarcinoma (COAD), esophageal carcinoma (ESCA), kidney renal clear cell carcinoma (KIRC), liver hepatocellular carcinoma (LIHC), head and neck squamous cell carcinoma (HNSC), lung adenocarcinoma (LUAD), lung squamous carcinoma (LUSC), pancreatic adenocarcinoma (PAAD), prostate adenocarcinoma (PRAD), rectum adenocarcinoma (READ), stomach adenocarcinoma (STAD), thyroid carcinoma (THCA), and uterine corpus endometrial carcinoma (UCEC). The dysregulated PMRGs were calculated by “edgeR” R package. The cut-off criteria were FDR < 0.05 and fold change (FC) ≥ 1.5. Based on mass spectrometry (MS) analysis between 10 cancer types including COAD, ESCA, HNSC, KIRC, LIHC, LUAD, LUSC, OV, PDAC, and UCEC and matched normal tissues from the CPTAC, differentially protein expression profiles were calculated through FC and evaluated by Wilcoxon rank-sum with FDR. We also analyzed immunohistochemistry staining profiles using 14 tumor tissues micro arrays, including breast, cervical, colorectal, endometrial, head and neck, liver, lung, ovarian, pancreatic, prostate, renal, stomach, thyroid, and urothelial cancers, based on the proportion of patients with high and medium staining levels from the HPA.

### Evaluation of cancer-associated molecular features

Based on TCGA HTS data in 17 cancer types, the fractions of 22 infiltrating immune cell types were calculated using CIBERSORT (https://cibersort.stanford.edu/index.php/) [20], and the stromal, immune levels, and tumor purity were estimated using “ESTIMATE” R package [21]. Obtained from UCSC Xena platform (https://xena.ucsc.edu/) detected by the Illumina Infinium HumanMethylation450 BeadChips platform, the sum of methylation levels of 485,577 CpG sites was recognized as overall DNA methylation level. We measured the total number of somatic variants per mega base (MB) of genome called tumor mutation burden (TMB) using “maftools” R package. Pearson’s correlation method was used to calculate the correlation of the above cancer-associated phenotypes and dysregulated pan-cancer PMRGs.

### Analysis of overall survival factors in pan-cancer tissues

We downloaded a combined pan-cancer cohort of TCGA, Therapeutically Applicable Research to Generate Effective Treatments (TARGET), and the Genotype-tissue Expression (GTEx) samples (*n* = 19,109) from UCSC Xena. High-quality tumor prognostic data were obtained from TARGET and Liu et al.’s published study [22]. Clinical survival data from 6,895 patients across 17 tumor types were included for the subsequent prognostic modeling. In our research, we firstly chose to use Cox proportional hazards regression model [23] and log-rank test in each tumor type to assess the association between individual gene expression levels and overall survival. An initial significance threshold of *P* < 0.20 was used. For tumor types in which no genes met this criterion, the *p*‑value threshold was incrementally relaxed, up to a maximum of 0.60, until at least one candidate gene was identified for further analysis. In addition, only genes showing a consistent direction of dysregulation (up‑ or down‑regulation) in cancer were retained.

Candidate genes passing the univariate screening were subjected to variable selection using least absolute shrinkage and selection operator (LASSO) regression with 10‑fold cross‑validation (CV). Best lambda was screened from lambda.min or lambda.1se value. For tumor types in which both univariate Cox regression and LASSO selection yielded only a single gene, this gene was directly entered into the prognostic modeling stage without performing stepwise Cox regression, as stepwise selection is only applicable when multiple candidates are present. When more than one gene was retained, multivariable modeling was performed using either direct multivariate Cox regression or stepwise Cox regression selected according to the Akaike Information Criterion (AIC) or Bayesian Information Criterion (BIC). In all cases where stepwise Cox regression was applied, AIC‑ and BIC‑based selections produced identical final models.

For certain tumor types, the resulting genomic model was integrated with relevant clinical variables to construct an integrated clinico‑genomic model if clinical factors with prognostic significance were available. The performance of each model was comprehensively evaluated across four key aspects: discrimination, calibration, risk stratification, and clinical utility. For discrimination, this involved calculating the concordance index (C-index) along with its 95% confidence intervals (CI) estimated through 1000 bootstrap resamplings, as well as time-dependent ROC curves with corresponding AUC values at 1, 3, and 5 years. Calibration was assessed using calibration curves and calibration slopes. Risk stratification was performed via Kaplan-Meier survival estimation, and clinical utility was evaluated employing both nomogram and decision curve analysis (DCA).

All prognostic factor analyses were conducted in R software (version 4.4.2). The following R packages were used: ‘’survival’’ (for Cox proportional hazards regression analyses), ‘’glmnet’’ (for least absolute shrinkage and selection operator regression with cross-validation), ‘’caret’’ package for model training and validation, ‘’survminer’’ package for Kaplan-Meier survival analysis and visualization, ‘’timeROC’’ package for time-dependent receiver operating characteristic curve analysis, ‘’rms’’ package for nomogram construction and calibration curve analysis, ‘’boot’’ package for bootstrap resampling to estimate confidence intervals, ‘’ggplot2’’ package for data visualization, ‘’pec’’ package for prediction error curve analysis, ‘’dcurves’’ package for decision curve analysis, and ‘’dplyr’’ package for data manipulation and preprocessing.

### Prediction of compounds for metabolism-related gene signatures

Connectivity Map (cMap) analysis was conducted by querying pan-cancer purine metabolism-related up-regulated gene tag and down-regulated gene tag for finding perturbagens that could give rise to similar expression signatures via clue.io software platform (https://clue.io/query). The negative normalized connectivity score less than 0 indicated a suppressive effect of compound on the up-regulated genes. Negative log10 transformed FDR q-values (fdr_q_nlog10) > 2 was set as the filter condition. Furthermore, pharmaco-transcriptomics and pharmaco-proteomics data were also obtained from Drugbank database (https://go.drugbank.com/).

## Results

### The pan-cancer cohort

**Fig. 1:**
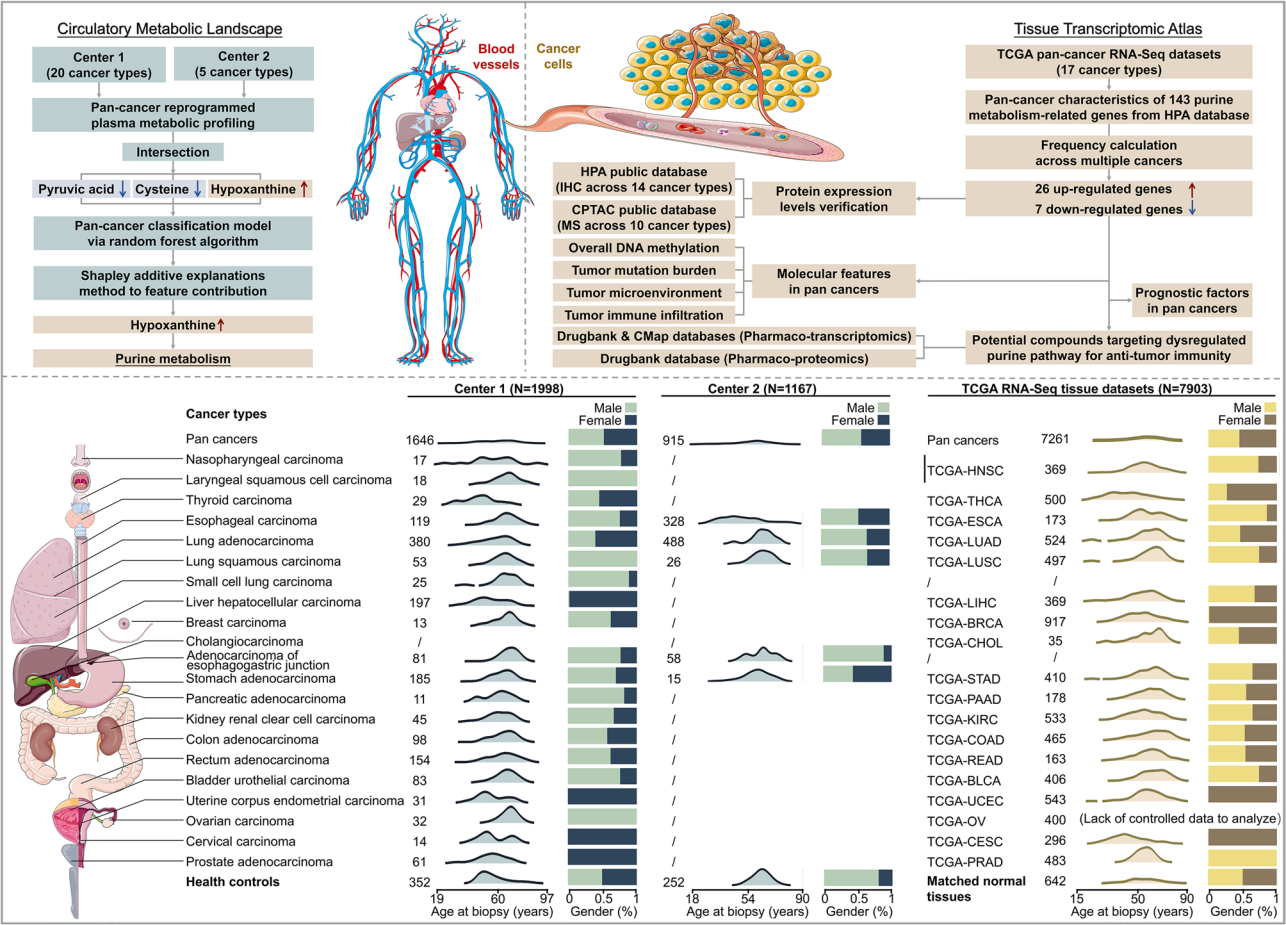
Overview of the pan-cancer study. **A** Schematic representation of the workflow used in this study. **B** Age and gender distribution of patients included for each cancer and the healthy cohort

In this study, we conducted a comprehensive characterization of plasma metabolites in two pan-cancer cohorts sourced from distinct clinical centers. Center 1 includes 1,646 patients representing twenty cancer types, including nasopharyngeal carcinoma (NPC, *n* = 17), laryngeal squamous cell carcinoma (LSCC, *n* = 18), THCA (*n* = 29), ESCA (*n* = 119), BRCA (*n* = 197), LUAD (*n* = 380), LUSC (*n* = 53), small cell lung carcinoma (SCLC, *n* = 25), LIHC (*n* = 13), PAAD (*n* = 11), adenocarcinoma of esophagogastric junction (AEG, *n* = 81), STAD (*n* = 185), KIRC (*n* = 45), ovarian carcinoma (OC, *n* = 14), UCEC (*n* = 31), CESC (*n* = 61), BLCA (*n* = 83), PRAD (*n* = 32), COAD (*n* = 98), and READ (*n* = 154), along with healthy controls (HC, *n* = 352). An additional plasma cohort from Center 2 consists of 915 patients across five cancer types: ESCA (*n* = 328), LUAD (*n* = 488), LUSC (*n* = 26), AEG (*n* = 58), and STAD (*n* = 15), along with HC (*n* = 252). Plasma samples were collected at the time of diagnosis and prior to the initiation of treatment. The methodology of this study and the distribution of subjects are comprehensively illustrated in Fig. 1.

### Pan-cancer circulating metabolite biomarkers

**Fig. 2:**
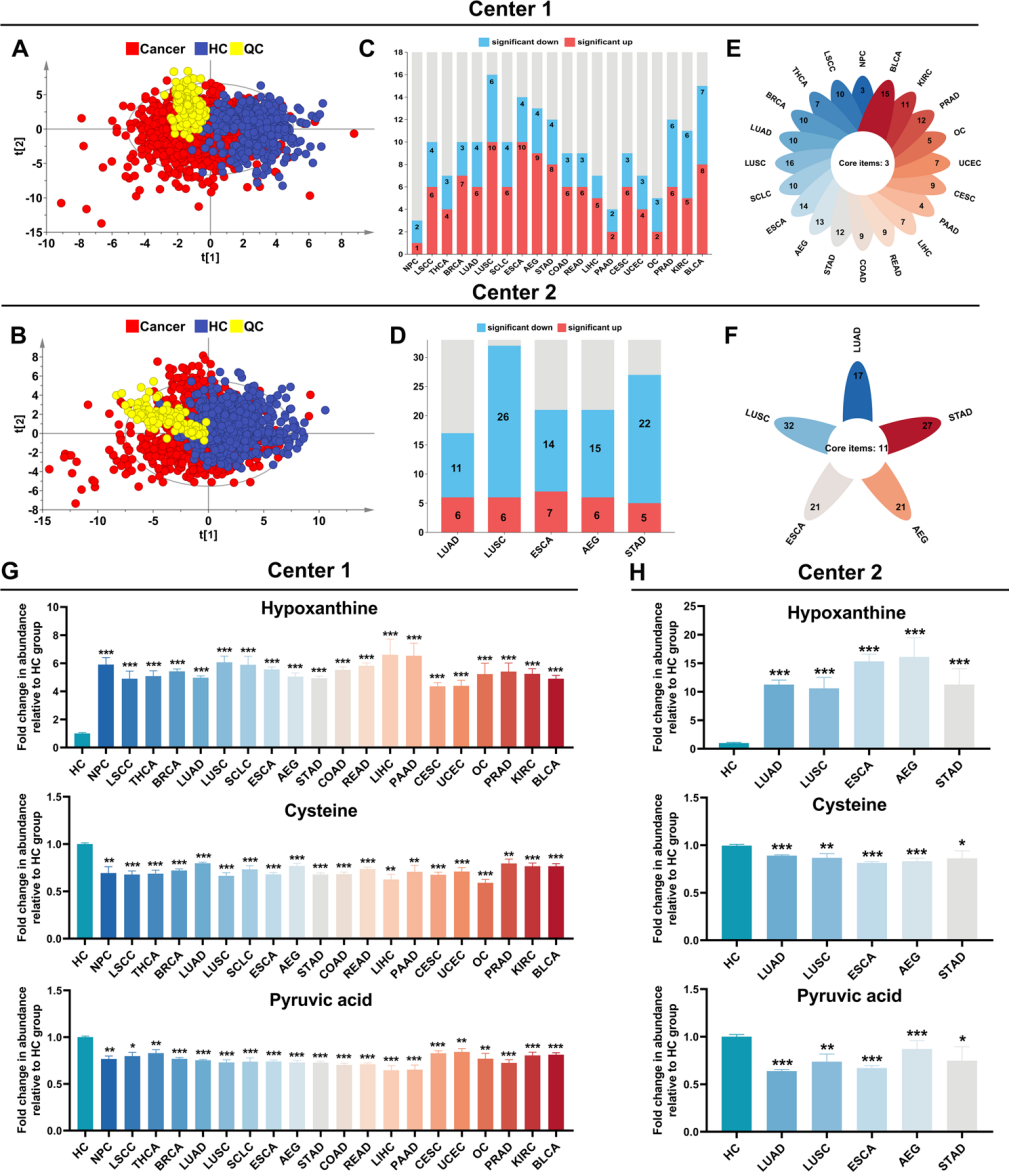
Fig. 2 Plasma metabolic landscape of cancer patients compared with healthy controls in two centers. **A** Principal Component Analysis (PCA) was conducted to compare cancer patients and healthy controls in Center 1. **B** A barplot illustrates the number of metabolites significantly upregulated, significantly downregulated, or showing no significant differential expression across all cancer types in Center 1. **C** A petaloid diagram displays the common metabolites across all cancer types in Center 1. Each petal represents a different cancer type, with distinct colors for each group. The central core shows the total number of shared metabolites, while the numbers on each petal indicate the count of differential metabolites specific to that cancer type. **D** PCA was conducted to compare cancer patients and healthy controls in Center 2. **E** A barplot demonstrates the number of metabolites significantly upregulated, significantly downregulated, or with no significant differential expression across all cancer types in Center 2. **F** A petaloid diagram shows the common metabolites across all cancer types in Center 2. Each petal represents a different cancer type, with distinct colors for each group. The central core displays the total number of shared metabolites, while the numbers on each petal indicate the count of differential metabolites specific to that cancer type. **G-H** Relative abundance of hypoxanthine, pyruvic acid, and cysteine in cancer patients and healthy controls in Center 1 and Center 2. (*, **, and *** denote p-values: 0.01 ≤ p < 0.05, 0.001 ≤ p < 0.01, and p < 0.001, respectively)

GC-MS analysis identified 58 plasma metabolites, including amino acids, carboxylic acids, carbohydrates, fatty acids, steroids, and other classes (Supplementary Figure S1 and Supplementary Table S1). PCA demonstrated significant separation between cancer patients and healthy controls (Fig. 2A, B), confirming global metabolome alterations in cancer. Pooled QC samples showed tight clustering, with 85% of metabolites exhibiting < 20% relative standard deviation (RSD) (Supplementary Table S2), supporting analytical robustness.

To systematically identify metabolic aberrations across diverse cancer types, differential expression analyses were conducted comparing each cancer type with its corresponding healthy control group. For cancers exhibiting sex-specific prevalence (e.g., prostate adenocarcinoma), analyses were confined to sex-matched cohorts. Metabolites achieving statistical significance (defined as FDR < 0.05 and fold change > 1.25 or < 0.8) were classified as differentially metabolites. Our analyses revealed consistent evidence of significant metabolic reprogramming in all malignancies relative to controls (Supplementary Tables S3-S27; Fig. 2C-F). Furthermore, these distinct metabolite profiles robustly differentiated various cancers from healthy individuals (Supplementary Figures S2, S3).

Notably, cross-center analysis spanning 20 malignancies revealed a conserved dysregulation pattern involving three key metabolites: hypoxanthine, a critical intermediate in purine metabolism, was significantly elevated across malignancies, while cysteine and pyruvic acid consistently showed reduced levels (Fig. 2G–H). Collectively, these findings suggest that these metabolites exhibit potential as pan-cancer circulating biomarkers.

### Metabolic biomarker-driven machine learning predictive model in pan-cancer cohort

**Fig. 3:**
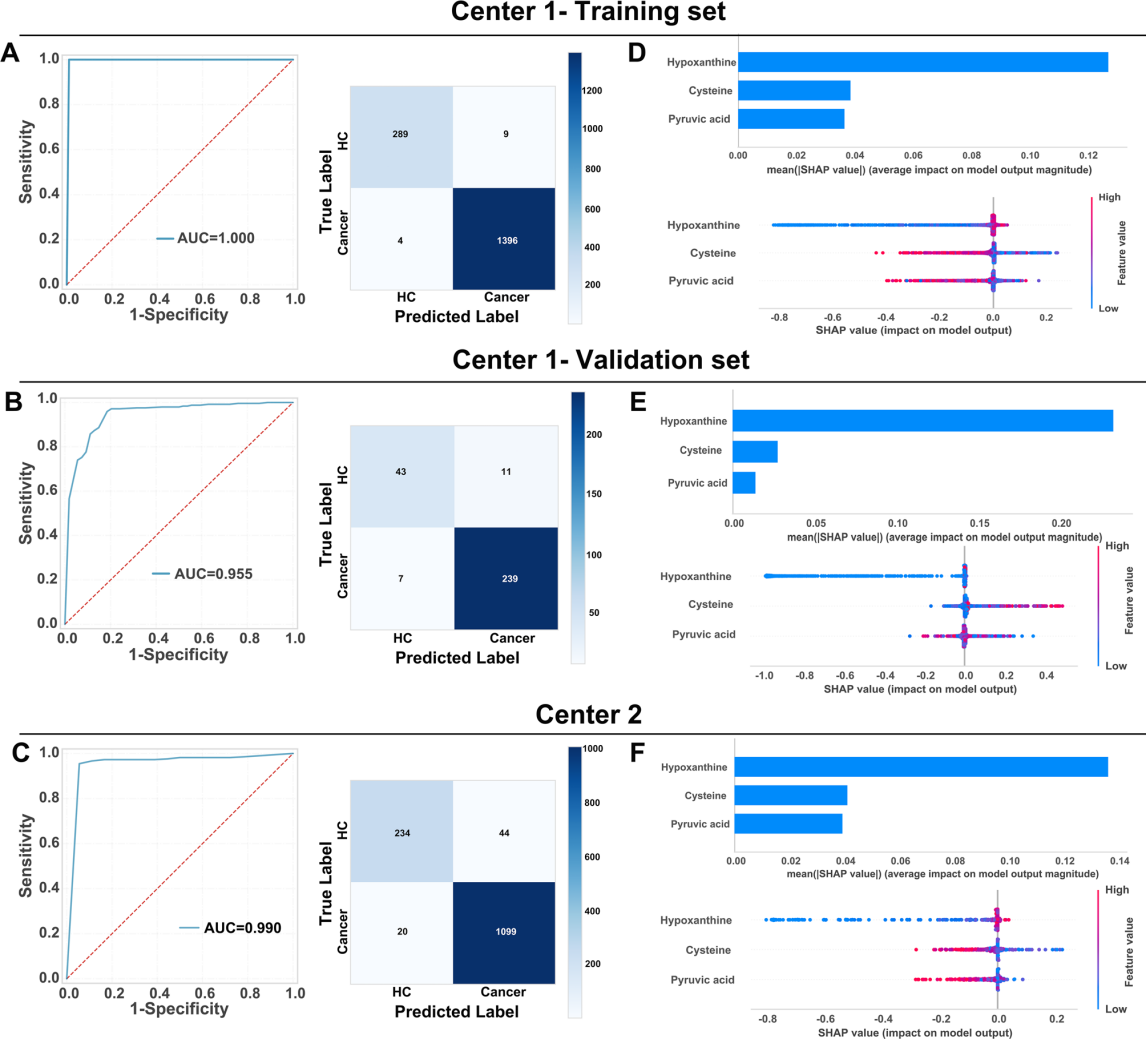
Machine learning-derived prediction model based on plasma metabolome for pan-cancer diagnosis. **A** The receiver operating characteristic (ROC) curve and confusion matrix for the Center 1-training set. **B** The contributions of the three key metabolites to the predictive model in the Center 1-training set, as assessed by the Shapley Additive Explanations (SHAP) method. **C** The ROC curve and confusion matrix for the Center 1-validation set. **D** The contributions of the three key metabolites to the predictive model in the Center 1-validation set. **E** The ROC curve and confusion matrix for the Center 2. **F** The contributions of the three key metabolites to the predictive model in the Center 2

To address the critical need for precise early cancer detection via metabolic reprogramming profiling, we developed a machine learning-driven pan-cancer diagnostic framework utilizing a synergistic biomarker panel comprising three metabolites: hypoxanthine, pyruvic acid, and cysteine. The optimized random forest classifier demonstrated exceptional discriminatory capacity, achieving ideal performance in the Center 1 training cohort (AUC = 1.00) while maintaining strong generalizability in validation (AUC = 0.955) and external testing at Center 2 (AUC = 0.990). Confusion matrices confirmed robust classification accuracy across both centers, with correct predictions significantly outnumbering errors (Fig. 3A, B, C).

To enhance the validation of the model, it was applied to distinct cohorts of cancer patients alongside their respective healthy control groups. The model exhibited outstanding performance across all cancer types, with AUC values ranging from 0.96 to 1.00, and demonstrated near-perfect sensitivity (0.93–1.00) and specificity (0.89–1.00). Additional performance metrics corroborated its robustness: accuracy (0.93–1.00), F1-score (0.93–1.00), Kappa coefficient (0.91–1.00), PPV (0.92–1.00), and NPV (0.90–1.00). The model’s discriminative capacity was further substantiated by prediction scores, which achieved optimal classification accuracy at a threshold of 0.57 (Supplementary Figure S4).

Shapley Additive Explanations (SHAP) analysis identified hypoxanthine as the most influential predictor (Fig. 3D-F). This robust feature importance aligns with hypoxanthine’s central role in purine metabolism and nucleotide depletion pathways—key characteristics of malignant transformation. Notably, hypoxanthine demonstrated excellent diagnostic discrimination between cancer patients and healthy controls, achieving AUC values of 0.89–0.99, sensitivities of 0.79–1.00, and specificities of 0.78–1.00 (Supplementary Figure S5).

### Tissue-resolved pan-cancer transcriptomic atlas of purine metabolism

Tumor cells may map their intrinsic transcriptional programs to peripheral blood through crosstalk with the tumor microenvironment, suggesting that circulating metabolites may reflect the metabolic activity of tumor tissues. Although tumor heterogeneity poses challenges, circulating metabolomics can provide insights into systemic metabolic states. Integration of pan-cancer metabolic reprogramming patterns with transcriptomics revealed shared oncogenic features, offering potential molecular targets for pan-cancer precision medicine. In our multicenter studies, pan-cancer plasma metabolomics identified hypoxanthine, a central node in purine metabolism, as the top-ranked predictor in both independent cohorts. To further investigate, we conducted tissue transcriptomic analyses focusing on purine metabolic pathways. Three cancer types (SCLC, AEG, and OV) were excluded due to inadequate data coverage (e.g., missing RNA-Seq or metabolomics data). After curation, RNA-Seq data from TCGA were analyzed, including 7,261 primary tumor samples and 642 normal adjacent tissues across 17 cancer types (filtered at FDR < 0.05 and |log2FC| ≥0.58; Supplementary Table S28).

**Fig. 4:**
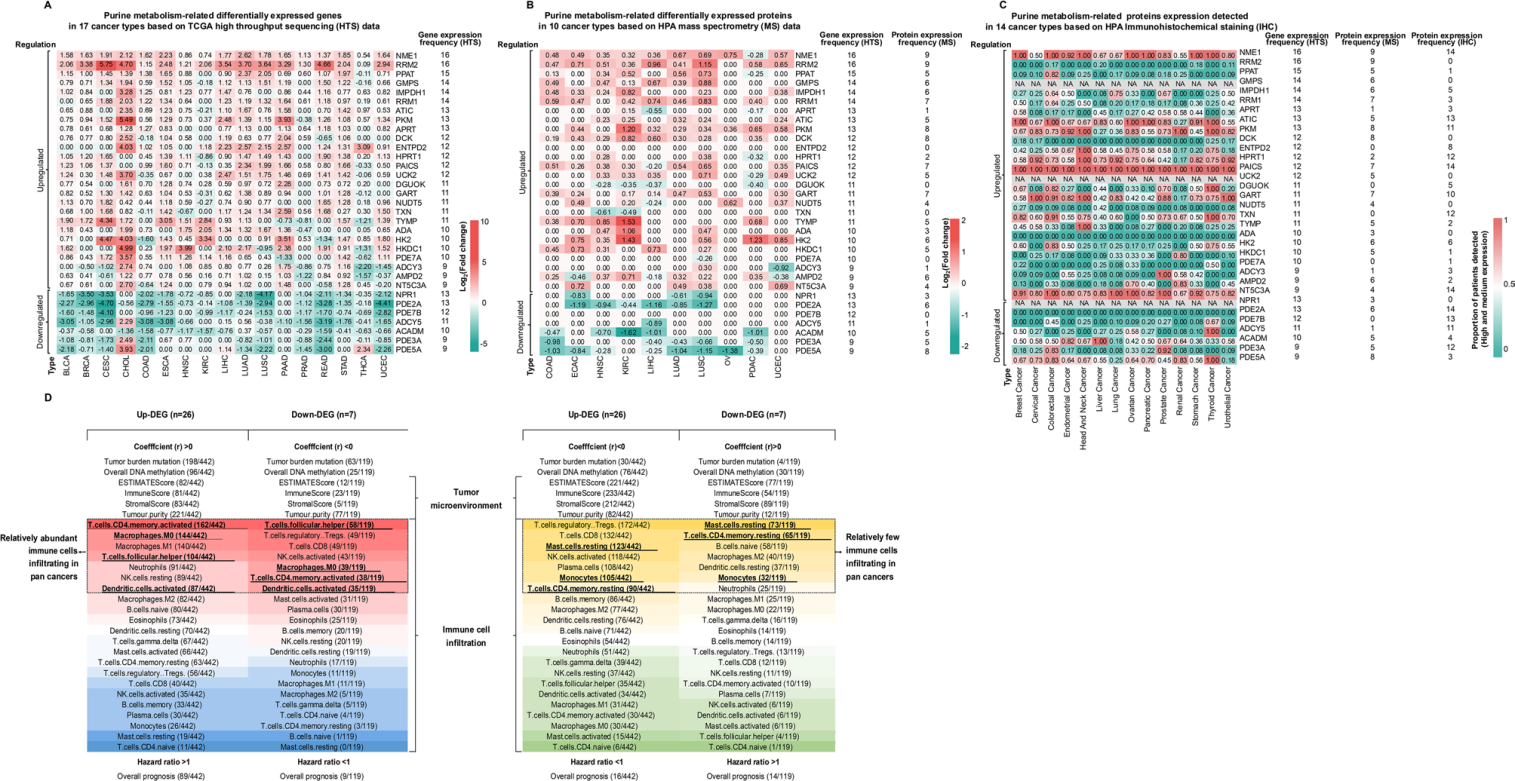
Results of pan-cancer bioinformatics analysis of purine metabolism-related genes. **A**-**C** Characteristics of purine metabolism-related pan-cancer signatures heatmaps calculated by mRNAs and protein expression levels. Three heatmaps showing the dysregulated genes with 17 cancer types in TCGA RNA-Seq datasets, 10 cancer types in CPTAC mass spectrometry (MS) data downloaded from HPA, and 14 cancer types in HPA immunohistochemical staining data. The color code represents the expression levels of signatures which are significant at FDR < 0.05 and foldchange (FC) > 1.5 or < 0.667. **D** Heatmaps of molecular features of purine metabolism-related pan-cancer signatures. Tumor burden mutation (TMB), overall DNA methylation, immune microenvironment (IME), and immune cell infiltration (ICI) were evaluated by Pearson’s correlation method. Overall prognosis was calculated by Cox proportional hazards regression model and log-rank tests. Pan-cancer cell frequencies of 22 immune cell types were annotated in parentheses on each line. Red-to-blue and yellow-to-green gradients indicate the decreasing cell proportion and the top one-third of immune cells were bordered with black dashed boxes. Black boxes represent the first 7 types of immune cells out of 22. For identifying 4 relatively abundant types of underlined immune cells in pan cancers, immune cell types positively correlated with up-regulated genes were compared with cell types negatively correlated with down-regulated genes. In the same way, cell types negatively correlated with down-regulated genes were compared with cell types positively correlated with up-regulated genes to screen out 3 relatively few types of underlined immune cells infiltrating in pan cancers

Using a predefined frequency cut-off (≥ 9 occurrences across 17 cancer types), we identified 26 up-regulated and 7 down-regulated PMRGs, defining them as core pan-cancer signature genes (Fig. 4A and Supplementary Table S29). Notably, up-regulated genes showed low expression in KIRC, PRAD, and THCA. Unexpectedly, *ADCY5* (Adenylate Cyclase 5), *PDE3A* (Phosphodiesterase 3 A), and *PDE5A* (Phosphodiesterase 5 A) were up-regulated in CHOL but down-regulated in most other cancers, potentially reflecting tissue-specific metabolic adaptations. In PAAD, only *ADCY5* was down-regulated, while the remaining 6 genes showed no significant changes.

To verify PMRGs expression at the protein level, we utilized MS data obtained from CPTAC and immunohistochemical staining (IHC) profiles provided by HPA (Fig. 4B and C, and Supplementary Table S29). Cancer patients have been annotated for different staining levels: high, medium, low, and not detected in HPA database. We calculated the proportions of high and medium levels of the total population and the cut-off value is 0.5. Among them, GMPS (Guanine Monophosphate Synthase), UCK2 (Uridine-Cytidine Kinase 2) and NPR1 (Natriuretic Peptide Receptor 1) expression levels cannot be found in IHC profiles. NME1 (Nucleoside Diphosphate Kinase 1), ATIC (5-Aminoimidazole-4-Carboxamide Ribonucleotide Formyltransferase/IMP Cyclohydrolase), PKM (Pyruvate Kinase M1/2), PAICS (Phosphoribosylaminoimidazole Carboxylase, Phosphoribosylaminoimidazole Succinocarboxamide Synthetase), GART (Phospho- ribosylglycinamide Formyltransferase), and PDE2A (Phosphodiesterase 2 A) exhibited an excellent agreement between mRNA and protein levels.

### Molecular features and prognostic values of pan-cancer dysregulated purine metabolism-related signatures

We systematically analyzed pan-cancer correlations of 22 immune infiltrating cell types across 17 cancer types. The top 30% of immunocytes (7 cell types, selected based on correlation strength ranks) with the strongest associations to 26 up-PMRGs and 7 down-PMRGs were prioritized (demarcated by dashed black boxes in Fig. 4D). Cancer-enriched immune subsets were defined as the intersection of cell types exhibiting both positive correlation with upregulated genes and negative correlation with downregulated genes (bolded and underlined labels), while cancer-underrepresented subsets showed the inverse pattern (negative correlation with up-PMRGs and positive correlation with down-PMRGs). Red-to-blue and yellow-to-green gradients indicate graded changes in cell proportion. Activated CD4 + T cells, M0 macrophages, T follicular helper cells, and activated dendritic cells were more prevalent in tumors, whereas resting mast cells, monocytes, and resting CD4 + memory T cells were less frequent in pan-cancer analyses. Complete correlation results for all 22 immune cell types and dysregulated gene signatures are provided in Supplementary Figure S6-S7 and Table S29.

Next, we evaluated tumor mutational burden, genome-wide DNA methylation, and immune microenvironment (estimated by ESTIMATE score) across the same 17 cancer types. No significant differences (FDR > 0.05) were observed when comparing molecular features associated with up- and down-regulated PMRGs, suggesting these metrics may reflect coexisting biological states rather than causality.

To assess prognostic significance, we analyzed 33 core pan-cancer PMRGs (previously defined purine metabolic reprogramming genes) using standardized expression and survival data from UCSC Xena. The workflow of prognostic model construction and gene inclusion overview are presented in Supplementary Figure S8. Supplementary Table S30 summarizes the number of alived (0) and deceased (1) patients, total sample sizes, and the number of gene variables entered into univariate Cox proportional hazards regression for each of the 17 tumor types analyzed. The proportion of deceased patients varied substantially, from 9.5% (THCA) to 59.1% (LUSC). Baseline data were analyzed for both clinical variables and gene expression levels [log_₂_ (raw count + 1) normalized] between different outcome groups within each tumor type. Detailed distributions of these baseline characteristics and expression levels are presented in Supplementary Tables S31. Tumor types did not meet the recommended events‑per‑variable (EPV) criteria for model construction. Therefore, the available data for each tumor type were not split into separate training and testing sets. In addition, no external validation dataset was available. Internal validation was instead performed using bootstrap resampling (1,000 iterations) to assess model stability and estimate the 95% confidence intervals of the performance metrics. Statistically significant differences in race were identified among BLCA, ESCA, and STAD, in gender in ESCA, and in tumor stage (1 = stage I + II, 2 = stage III + IV) among BLCA, BRCA, COAD, ESCA, KIRC, HNSC, LIHC, LUAD, READ, STAD, THCA, and UCEC, using the chi square test with FDR adjustment (FDR < 0.05 considered significant).

Univariate Cox proportional hazards regression and log-rank tests were applied to initially evaluate overall survival and identify the potential prognostic gene variables for LASSO regression and multivariable Cox modeling (Supplementary Figure S9-S10). Forrest plots revealed heterogeneous prognostic effects across cancer types. The most notable prognostic gene was PAICS, an up-regulated PMRG associated with worse survival in 7 cancers: BRCA (HR = 1.46, 95% CI = 1.16–1.83, *P* < 0.01), CESC (HR = 1.46, 95% CI = 1.07–2.01, *P* < 0.05), HNSC (HR = 1.25, 95% CI = 1.03–1.50, *P* < 0.05), LIHC (HR = 1.28, 95% CI = 1.01–1.62, *P* < 0.05), LUAD (HR = 1.44, 95% CI = 1.21–1.73, *P* < 0.0001), PAAD (HR = 1.70, 95% CI = 1.19–2.45, *P* < 0.01), and THCA (HR = 3.39, 95% CI = 1.30–8.83, *P* < 0.05).

Following the modeling procedures described above, optimal prognostic models were successfully established for each tumor type listed in Supplementary Table S32. The number of dysregulated purine metabolism‑related genes included in the final models ranged from 1 to 8 across tumor types. The optimal prognostic modeling pathways adopted for each tumor type are summarized in Supplementary Figure S11, alongside the specific genes incorporated into each optimal model, as follows: BLCA: *IMPDH1*, *PDE7B*; BRCA: *PAICS*, *TXN*; CESC: *GART*, *HK2*; CHOL: *RRM2*; COAD: *ADA*; ESCA: *HPRT1*, *NT5C3A*; HNSC: *ADA*, *ADCY5*, *HPRT1*, *PKM*; KIRC: *ACADM*, *IMPDH1*, *NME1*, *PDE7B*, *RRM2*, *TYMP*; LIHC: *ADA*, *ATIC*, *IMPDH1*, *PDE2A*, *PPAT*, *RRM2*, *TXN*, *UCK2*; LUAD: *PDE5A*, *RRM2*, *TXN*, *UCK2*; LUSC: *ADA*, *PKM*; PAAD: *HKDC1*, *RRM2*; PRAD: *APRT*; READ: *APRT*, *PAICS*; STAD: *APRT*, *PAICS*; THCA: *IMPDH1*; UCEC: *ADA*, *UCK2*. The detailed evaluation figures for the optimal genomic and integrated clinico‑genomic models derived for 17 tumor types are provided in Supplementary Figures S12-S28. Notably, due to insufficient survival data and biased distribution of clinical information, integrated clinico‑genomic models could not be constructed or evaluated for PRAD and READ. While the performance metrics, including C-index and AUC values, varied across tumor types, it is important to acknowledge that the observed performance, particularly in some cancer types, might be influenced by limitations such as sample size or the specific clinical information available. Therefore, these results should be interpreted with caution, acknowledging the inherent heterogeneity of pan-cancer data.

For all other tumor types, integrated clinico‑genomic models consistently demonstrated superior discrimination, accuracy, and net benefit compared to their respective genomic models. Overall, the set of prognostic genes retained in the final models showed marked heterogeneity across tumor types. Specifically, *ADA* and *RRM2* were the most frequently retained genes, appearing in five tumor types. *IMPDH1* was included in four tumor types, while *APRT*, *PAICS*, *TXN*, and *UCK2* each occurred in three tumor types. *HPRT1*, *PKM*, and *PDE7B* were retained in two tumor types, with *PDE7B* being down‑regulated. The remaining genes, *ATIC*, *GART*, *HK2*, *HKDC1*, *NME1*, *NT5C3A*, *PPAT*, *TYMP*, *ACADM*, *ADCY5*, *PDE2A*, and *PDE5A*, were present in only one tumor type.

### Potential drugs targeting pan-cancer dysregulated purine metabolism-related signatures

**Fig. 5:**
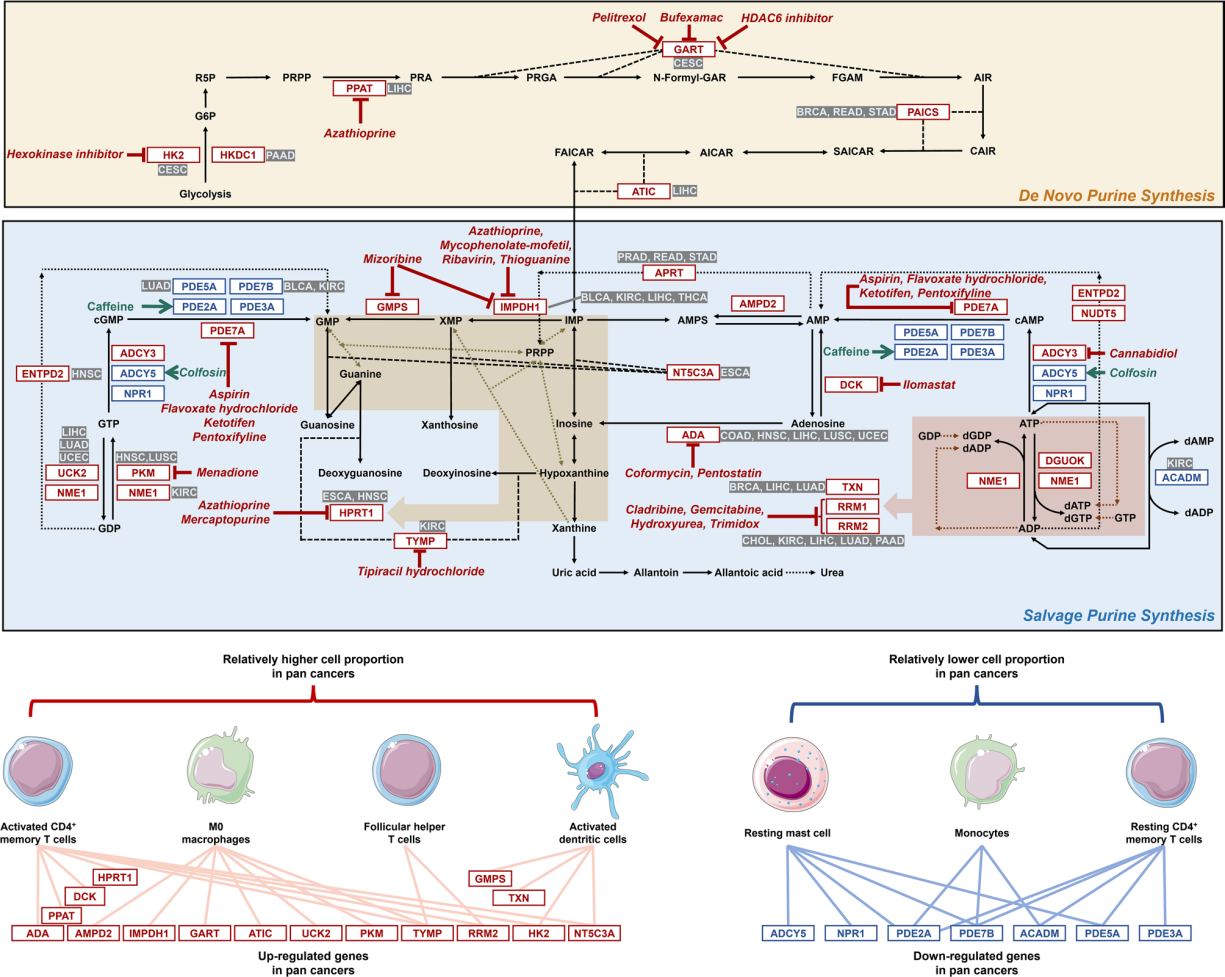
Potential drugs targeting pan-cancer dysregulated purine metabolism-related signatures. The diagram depicts the de novo (top) and salvage (bottom) purine synthesis pathways. Gene/protein nodes highlighted with red boxes are up‑regulated purine‑metabolism‑related genes across cancers, and those in blue boxes are down‑regulated. For each pathway gene/protein node, tumor types in which the gene showed significant prognostic association in our modeling analyses are indicated adjacent to the box. Candidate drugs potentially targeting specific pathway genes are shown in red or green italic font. The lower panels illustrate immune cell subsets whose relative proportions were found to be positively (left) or negatively (right) correlated with the expression of up‑regulated or down‑regulated genes, respectively, in the pan‑cancer analysis. G6P: glucose-6-phosphatase; R5P: ribose 5-phosphate; PRPP: phosphoribosyl pyrophosphate; PRA: 5-phosphoribosylamine; PRGA: 5-phosphoribosylglycinamide; N-Formyl-GAR: 5’-phosphoribosyl-N-formylglycinamide; FGAM: 5’-phosphoribosylformylglycinamidine; AIR: 5-aminoimidazole ribonucleotide; CAIR: 4-carboxy-5-aminoimidazole ribonucleotide; SAICAR: phosphoribosylaminoimidazolesuccinocarboxamide; AICAR: 5-amino-4-imidazolecarboxamide; FAICAR: 5-formamidoimidazole-4-carboxamide ribotide; IMP: inosinic acid; AMPS: adenylosuccinate; AMP: adenosine monophosphate; cAMP: cyclic adenosine monophosphate; ATP: adenosine triphosphate; ADP: adenosine diphosphate; XMP: xanthosine-5-phosphate; GMP: guanosine 5’-monophosphate; cGMP: cyclic guanosine monophosphate; GTP: guanosine-5’-triphosphate; GDP: guanosine diphosphate; dADP: deoxyadenosine diphosphate; dAMP: deoxyadenosine monophosphate; dGDP: deoxyguanosine diphosphate; dGTP: deoxyguanosine triphosphate

Pharmaco-multi-omics data downloaded from public databases (DrugBank and cMap) was used to computationally predict potential compounds targeting gene signatures associated with pan-cancer dysregulated purine metabolic pathway (Supplementary Figure S29). The de novo and salvage purine synthesis pathways were annotated based on the Metabolic Atlas (https://metabolicatlas.org/), as summarized in Fig. 5. Several gene, especially ADA mRNA and RRM2 mRNA, the most frequently retained prognostic gene signatures across multiple tumor types (five in total for each), have candidate drugs indicated in the pathway diagram that were identified from our drug‑matching procedure with relatively high weights. In our analysis, enzyme GART was computationally predicted as a target for pelitrexol, bufexmac, and HDAC6 (Histone Deacetylase 6) inhibitor. The hexokinase inhibitor was predicted to specifically target HK2 (Hexokinase 2). Similarly, azathioprine showed predicted binding affinity for PPAT (Phosphoribosyl Pyrophosphate Amidotransferase), IMPDH1 (Inosine Monophosphate Dehydrogenase 1), and HPRT1 (Hypoxanthine Phosphoribosyltransferase 1). IMPDH1 was also predicted to interact with mycophenolate-mofetil, ribavirin, thioguanine, and mizoribine, while mercaptopurine was associated with HPRT1. Additionally, GMPS was identified as a potential target of mizoribine. Other predicted interactions included aspirin, flavoxate hydrochloride, ketotifen, and pentoxifylline with PDE7A (Phosphodiesterase 7 A), and menadione with PKM. TYMP (Thymidine Phosphorylase) was linked to tipiracil and its hydrochloride form, whereas coformycin and pentostatin were predicted to target ADA (Adenosine Deaminase). RRM1 (Ribonucleoside-diphosphate Reductase Subunit M1) and RRM2 (Ribonucleoside-diphosphate Reductase Subunit M2) were potentially modulated by cladribine, gemcitabine, hydroxyurea, and trimidox. Finally, ilomastat, cannabidiol, caffeine, and colfosin showed predicted activity against DCK (Deoxycytidine Kinase), ADCY3 (Adenylate Cyclase 3), PDE2A, and ADCY5, respectively.

## Discussion

In this study, we employed an analytical framework structured around three interconnected levels—“phenomena, patterns, and essence”—to advance our understanding of cancer progressively from effects to causes and from superficial to fundamental insights. Multi-dimensional data integration (metabolomics, transcriptomics, proteomics) enabled us to systematically map pan-cancer biological regulatory networks across circulation and tumor tissue levels from a systems biology perspective. Furthermore, we combined transcriptomic analysis of the tumor microenvironment with proteomic data for drug prediction suggesting potential immunotherapeutic agents targeting reprogrammed metabolic pathways.

We employed a GC-MS-based metabolomics approach to systematically investigate plasma metabolites across 20 cancer types. By conducting simultaneous detection and analysis on a unified platform, we developed a high-accuracy machine learning model for pan-cancer screening, containing upregulated hypoxanthine and downregulated pyruvic acid and cysteine. The elevation of circulating hypoxanthine may be indicative of a link to potential purine metabolic reprogramming (PMR) in cancer cells, though direct evidence within tumor tissue is needed. This study highlights these shared metabolic changes at a pan-cancer level, offering new insights into the exploration of pan-cancer biomarkers. However, the mechanistic links between these metabolic alterations and oncogenic processes remain poorly defined. Future studies should employ CRISPR-mediated metabolite depletion in relevant models (e.g., patient-derived organoids), coupled with prospective validation in multi-center cohorts to explore whether these metabolic alterations are functionally test the hypothesis that these metabolites contribute to immune evasion by suppressing anti-tumor immunity.

Previous studies have demonstrated the importance of purine metabolism in the metabolic reprogramming characteristic of malignant tumors, across multiple cancers such as kidney cancer [24], breast cancer [25], lung cancer [26, 27], hepatocellular carcinoma [28], pancreatic cancer [29], cholangiocarcinoma [30], and glioma [31]. A recent study revealed that maintaining nucleotide pools to promote tumor growth and survival relies on both de novo and salvage routes in cancers [32]. Xanthine oxidoreductase (XOR), responsible for the oxidation of hypoxanthine to xanthine and xanthine to uric acid (UA) [33, 34], can generate reactive oxygen species (ROS) that further promotes inflammation and induce autophagy and apoptosis resistance [9, 35]. Hu et al. reported an artificial metabzyme catalyzing the metabolic conversion of xanthine into uric acid in cancers [36]. Tumor cells often remodel metabolic pathways to cause drug resistance in metabolic-targeted therapies [37]. Combined with immune checkpoint blockade (ICB) therapy, agents blocking purine metabolism regulating DNA/RNA synthesis, such as methotrexate and fluorouracil, could potentially be explored to enhance anti-tumor immunity [27, 38]. Future studies should investigate whether targeting both synthesis routes simultaneously can improve drug resistance. Our metabolomic findings provided a theoretical foundation for our further investigation into pan-cancer PMR.

Then, based on gene-metabolite networks obtained from the Metabolism Atlas database, we identified 33 core gene signatures (pan-cancer PMRGs) dysregulated across cancers and validated their protein expression levels to explore genotype-phenotype associations. This integrative approach synergized tissue transcriptomics, tissue proteomics, and circulating metabolomics, enabling a systematic and comprehensive investigation into cancer systems biology. However, a significant observation was that protein expression levels for most up-regulated pan-cancer PMRGs did not agree with transcript levels in IHC staining. While this disagreement could partially stem from the small sample size, it strongly suggests the possibility of complex post-transcriptional or post-translational regulation impacting functional protein levels; this discordance underscores a key challenge in establishing direct genotype-phenotype correlations solely based on transcriptomics. To investigate molecular features of pan-cancer PMRGs, we systematically analyzed correlations between PMRG expression and TMB, TIME, overall DNA methylation, and immune cell infiltration (ICI) in pan-cancer tissues. We also identified prognostic factors from pan-cancer PMRGs in TCGA tumor tissues. Activated CD4 + memory T cells were frequently present, while resting CD4 + memory T cells were less prevalent, except in CESC, HNSC, PRAD, and STAD. Consistent with previous studies proposing that a higher ratio of activated CD4 + memory T cells indicates better prognosis [39–43], our data revealed associations between several of the eight upregulated pan-cancer PMRGs and overall prognosis across multiple cancer types, particularly in TCGA-LIHC, TCGA-LUAD, and TCGA-PAAD. These associations point to potential links, but mechanistic drivers require experimental validation. In some solid cancers, tumor-infiltrating CD4 + T cells exhibit higher expression of coinhibitory receptors associated with exhaustion and poor ICB response/survival than adjacent tissue-infiltrating CD4 + T cells [44–48]. Based on observed immunological associations in our pan-cancer study, our subsequent computational analysis of pan-cancer PMRGs predicted that aspirin could potentially inhibit salvage purine synthesis, possibly mediated by PDE7A targeting — an anti-tumor metastatic effect documented via other mechanisms [49–51]. Similarly, azathioprine was predicted to exert multi-target effects by modulating PPAT, IMPDH1, and HPRT1 genes to potentially interfere with de novo and salvage purine synthesis pathways. Other compounds, including cannabidiol (targeting ADCY3), colfosin (targeting ADCY5), and caffeine (targeting PDE2A), were also computationally flagged as having immunomodulatory potential via these pathways. Pan-cancer research offers a valuable entry point for investigating strategies aimed at counteracting tumor immune evasion.

Despite encouraging results, this study has limitations including a small, low-diversity external validation cohort dominated by lung cancer cases, necessitating future multi-center studies with balanced tumor subtypes to verify generalizability; methodological inconsistencies in transcriptomic data (e.g., RNA-seq protocols and batch effects) risking reproducibility; a pan-cancer design obscuring tumor-type-specific heterogeneity; and most critically, the absence of functional validation for causal claims (e.g., modulating PMRGs or metabolites in immune evasion assays). Future research must therefore integrate multi-omics with experimental validation to mechanistically link metabolic reprogramming to immune evasion.

Purines, as fundamental building blocks of DNA and RNA, provide sufficient purine nucleotides for cellular functions. A detailed functional understanding of key regulatory molecules in purine metabolism might eventually reveal their impact on cancer cell proliferation, survival, and immune evasion, providing evidence that could guide future translational research regarding metabolic reprogramming in tumors. Our study provides pan-cancer associations and computational predictions to inform such future mechanistic and therapeutic investigations.

## Supplementary Information

Supplementary Material 1.

Supplementary Material 2.

Supplementary Material 3.

Supplementary Material 4.

Supplementary Material 5.

Supplementary Material 6.

## Data Availability

The data that support the findings of this study are available from the corresponding author upon reasonable request.
