# Supplementary material for: Purine metabolism: a pan-cancer metabolic dysregulation across circulation and tissues
Source: Mol Cancer. 2025 Oct 14;24:255. doi: 10.1186/s12943-025-02482-9 (PMC12522462; doi:10.1186/s12943-025-02482-9)
Supplement: Supplementary file 1 — Supplementary Material 1. [file 12943_2025_2482_MOESM1_ESM.pdf]

Purine metabolism: a pan-cancer metabolic dysregulation across circulation and tissues

Mengjie Yu[1,#], Cheng Liu[2,#], Minmin Cao[3,#], Dou Yang[1], Tongshan Wang[4], Jing Xu[4], Danxia Zhu[5], Guangji Wang[1,*], Jiye Aa[1,*], Wei Zhu[4,*]

[1] State Key Laboratory of Natural Medicines, China Pharmaceutical University, Nanjing, Jiangsu 210009, P.R. China.

[2] Department of Gastroenterology, Nanjing Drum Tower Hospital Clinical College of Nanjing Medical University, 321 Zhongshan Road, Nanjing, Jiangsu 210008, P.R. China.

[3] Department of Oncology, The Jiangyin Hospital Affiliated to Medical College of Southeast University, Jiangyin, Jiangsu 214400, P.R. China.

[4] Department of Oncology, The First Affiliated Hospital with Nanjing Medical University, Guangzhou Road, Nanjing, Jiangsu 210029, P.R. China.

[5] Department of Oncology, The Third Affiliated Hospital of Soochow University,185 Juqian Road, Changzhou, Jiangsu 213000, P.R. China.

*Correspondence: Wei Zhu, Department of Oncology, The First Affiliated Hospital with Nanjing Medical University, Guangzhou Road, Nanjing, Jiangsu 210029, China. Email: zhuwei@njmu.edu.cn. Jiye Aa, State Key Laboratory of Natural Medicines, China Pharmaceutical University, Nanjing, Jiangsu 210009, P.R. China. Email: jiyea@cpu.edu.cn. Guangji Wang, State Key Laboratory of Natural Medicines, China Pharmaceutical University, Nanjing, Jiangsu 210009, P.R. China. Email: guangjiwang@hotmail.com.

[#] Mengjie Yu, Cheng Liu and Minmin Cao contributed equally to this work.

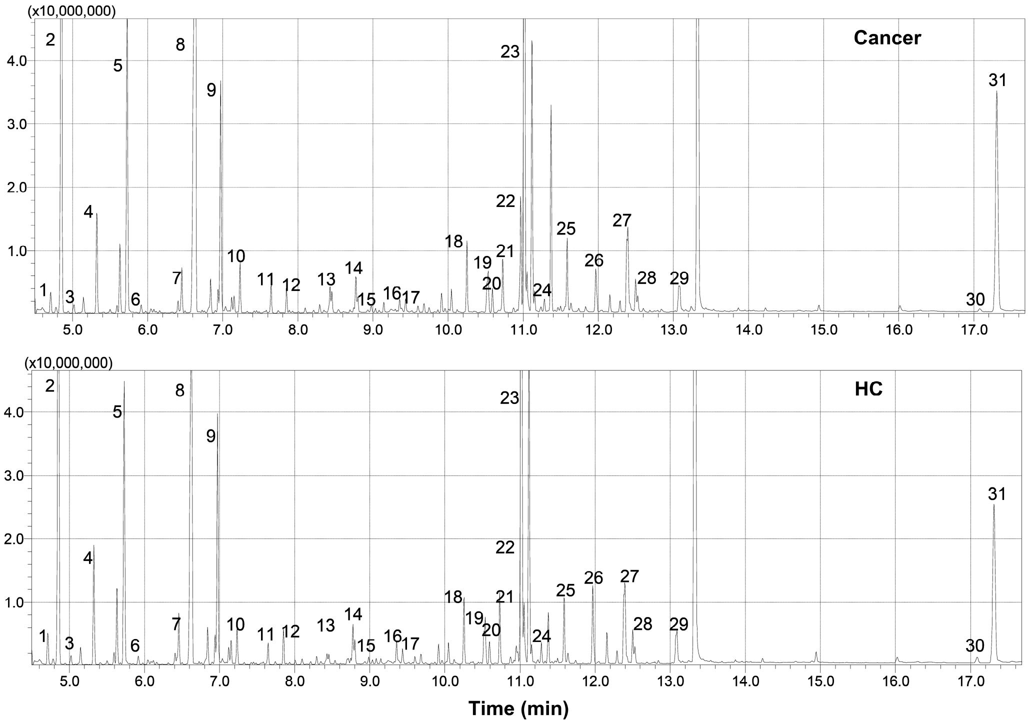

Supplementary Figure S1. Representative total ion current chromatogram obtained from GC-MS analysis of plasma samples collected from a cancer patient and a healthy volunteer. The identified peaks include: 1, Pyruvic acid; 2, Lactic acid; 3, Glycolic acid; 4, Alanine; 5, Oxalic acid; 6, 3-Hydroxybutyric acid; 7, Valine; 8, Urea; 9, Phosphate; 10, Glycine; 11, Serine; 12, Threonine; 13, Aminomalonic acid; 14, Pyroglutamic acid; 15, Cysteine; 16, Glutamic acid; 17, Phenylalanine; 18, Glutamine; 19, Hypoxanthine; 20, Myristic acid-1,2-$^{13}C_2$ (internal standard); 21, Fucose; 22, Fructose; 23, Glucose; 24, Tyrosine; 25, Palmitic acid; 26, myo-Inositol; 27, Linoleic acid; 28, Stearic acid; 29, Arachidonic acid; 30, alpha-Tocopherol; 31, Cholesterol.

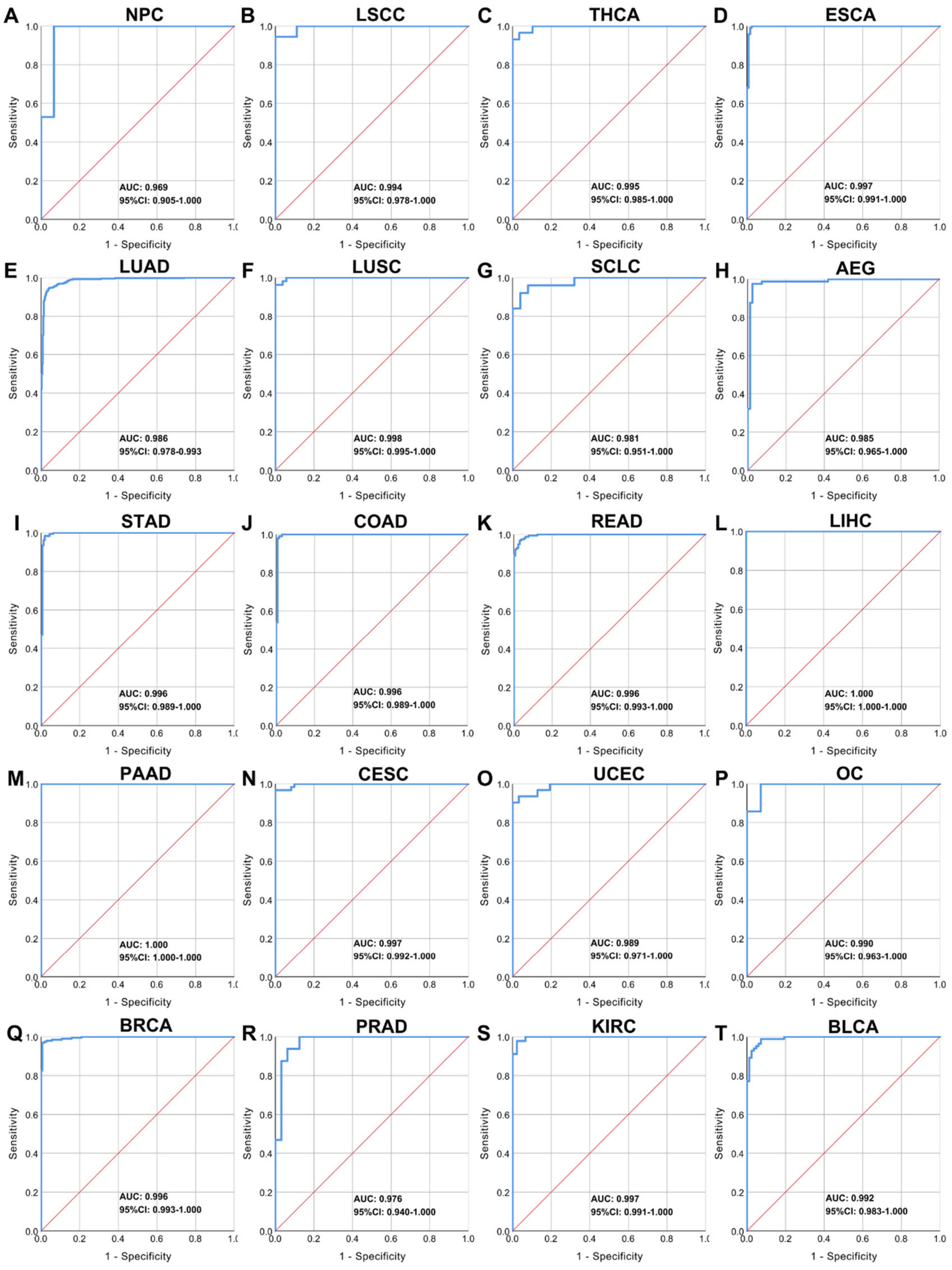

Supplementary Figure S2. The receiver operating characteristic (ROC) curve analysis diagram of the Logistic classification model based on the differential metabolite combinations constructed in Center 1 for cancer patients and healthy controls.

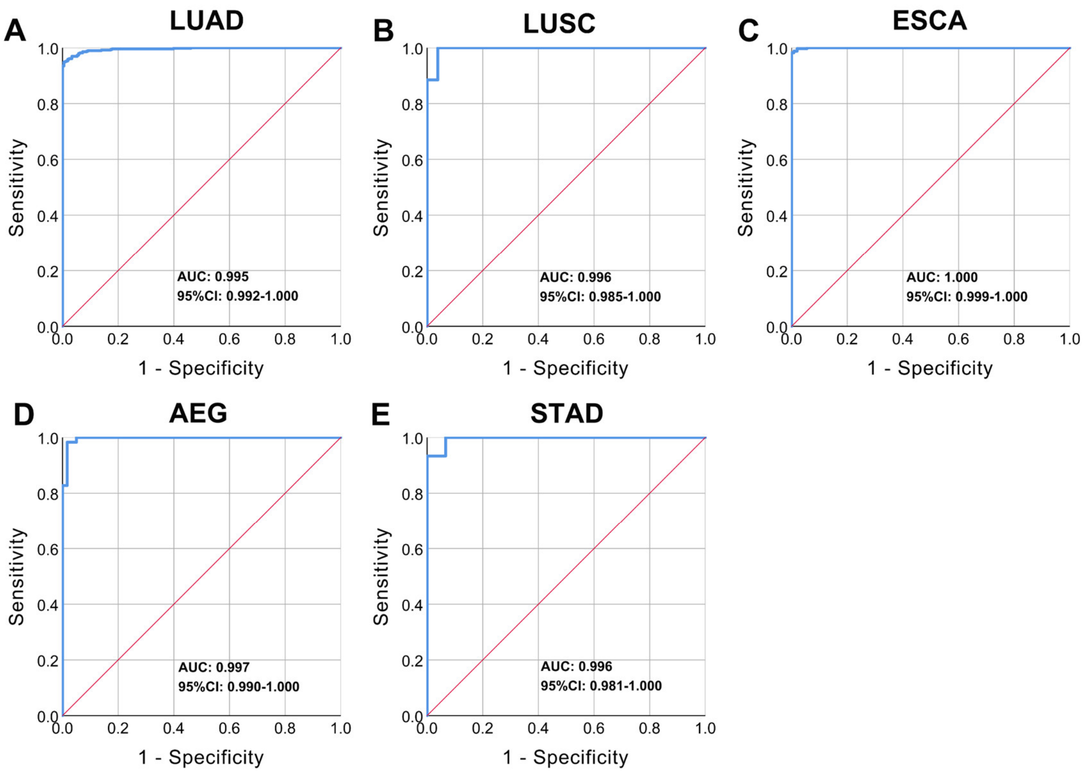

Supplementary Figure S3. The receiver operating characteristic (ROC) curve analysis diagram of the Logistic classification model based on the differential metabolite combinations constructed in Center 2 for cancer patients and healthy controls.

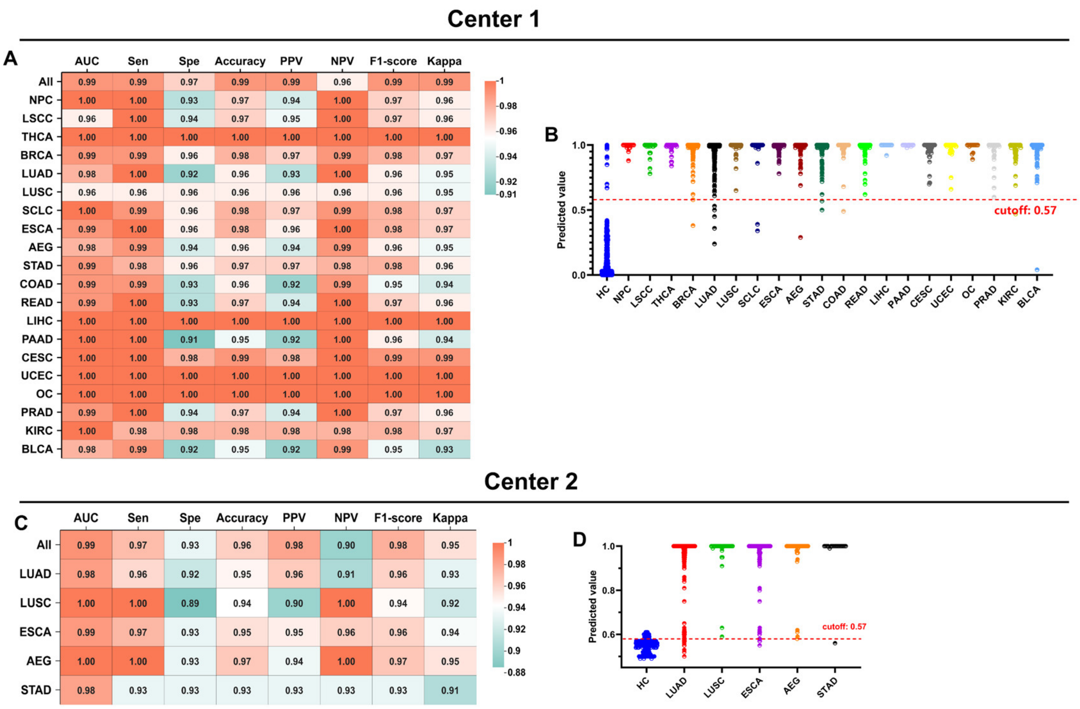

Supplementary Figure S4. The classification performance of the pan-cancer diagnostic model for individual cancer patients and healthy controls. (A, C) Heatmap showing the performance of the prediction model in distinguishing between cancer patients and healthy individuals in Center 1 (A) and Center 2 (C). PPV=positive predictive value; NPV=negative predictive value. (B, D) The performance of the prediction model for distinguishing cancer patients from healthy individuals in Center 1 (B) and Center 2 (D). The dotted line represented the cutoff value of 0.57. AUC= area under the curve, Sen=sensitivity, Spe=specificity.

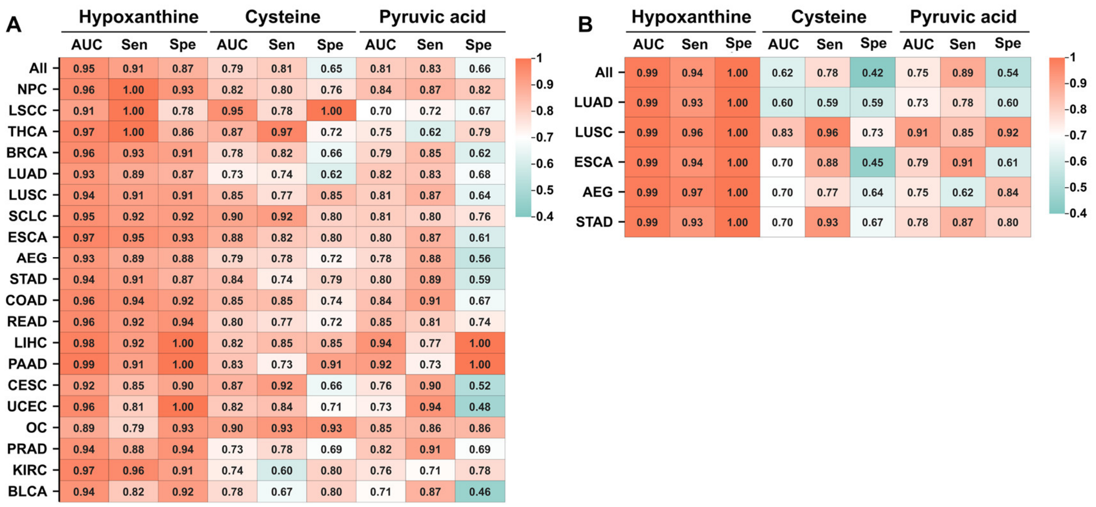

Supplementary Figure S5. Heatmap showing the performance of hypoxanthine, cysteine and pyruvic acid in distinguishing between cancer patients and healthy individuals in Center 1 (A) and Center 2 (B). AUC= area under the curve, Sen=sensitivity, Spe=specificity.

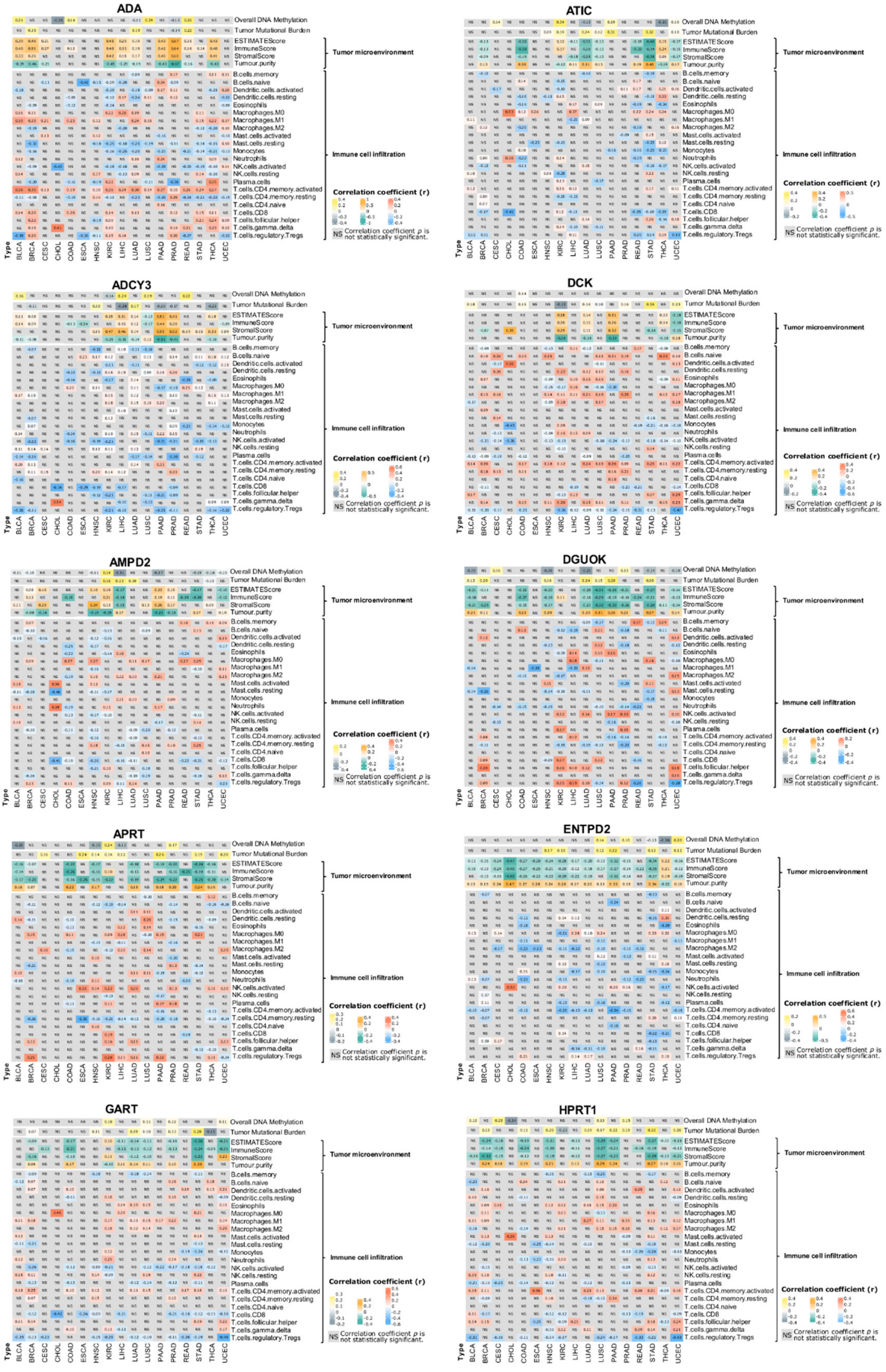
ADA
ATIC
ADCY3
DCK
AMPD2
DGUOK
APRT
ENTPD2
GART
HPRT1
Overall DNA Methylation
Tumor Mutational Burden
ESTIMATEScore
ImmuneScore
StromalScore
Tumour.purity
Tumor microenvironment
B.cells.memory
B.cells.naive
Dendritic.cells.activated
Dendritic.cells.resting
Eosinophils
Macrophages.M0
Macrophages.M1
Macrophages.M2
Mast.cells.activated
Mast.cells.resting
Monocytes
Neutrophils
NK.cells.activated
NK.cells.resting
Plasma.cells
T.cells.CD4.memory.activated
T.cells.CD4.memory.resting
T.cells.CD4.naive
T.cells.CD8
T.cells.follicular.helper
T.cells.gamma.delta
T.cells.regulatory.Tregs
Immune cell infiltration
Correlation coefficient (r)
NS Correlation coefficient p is not statistically significant.
Type
BLCA
BRCA
CESC
CHOL
COAD
ESCA
HNSC
KIRC
LIHC
LUAD
LUSC
PAAD
PRAD
READ
STAD
THCA
UCEC

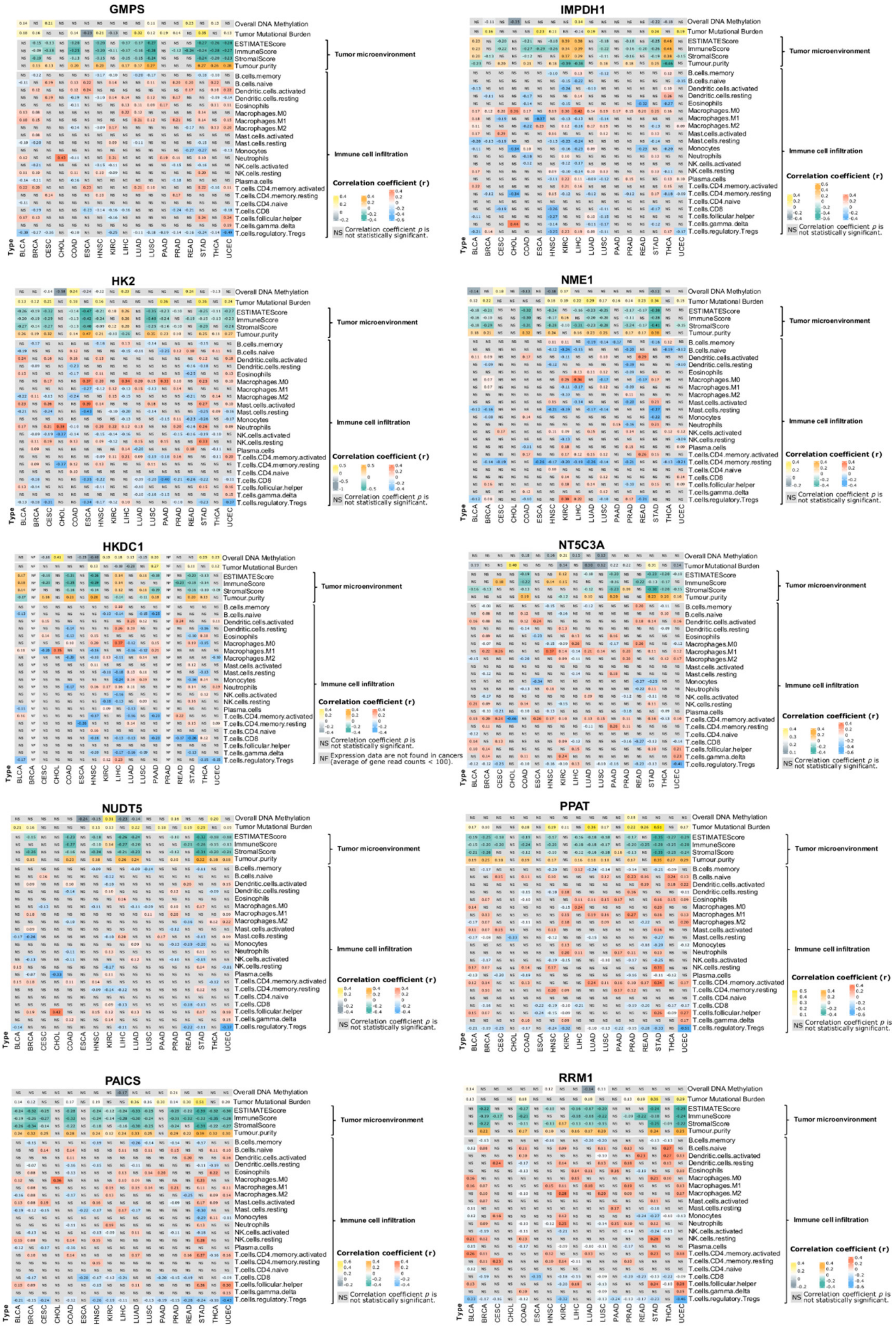
GMPS
IMPDH1
HK2
NME1
HKDC1
NT5C3A
NUDT5
PPAT
PAICS
RRM1
Overall DNA Methylation
Tumor Mutational Burden
ESTIMATEScore
ImmuneScore
StromalScore
Tumour.purity
Tumor microenvironment
B.cells.memory
B.cells.naive
Dendritic.cells.activated
Dendritic.cells.resting
Eosinophils
Macrophages.M0
Macrophages.M1
Macrophages.M2
Mast.cells.activated
Mast.cells.resting
Monocytes
Neutrophils
NK.cells.activated
NK.cells.resting
Plasma.cells
T.cells.CD4.memory.activated
T.cells.CD4.memory.resting
T.cells.CD4.naive
T.cells.CD8
T.cells.follicular.helper
T.cells.gamma.delta
T.cells.regulatory.Tregs
Immune cell infiltration
Correlation coefficient (r)
NS Correlation coefficient p is not statistically significant.
NF Expression data are not found in cancers (average of gene read counts < 100).
Type
BLCA
BRCA
CESC
CHOL
COAD
ESCA
HNSC
KIRC
LIHC
LUAD
LUSC
PAAD
PRAD
READ
STAD
THCA
UCEC

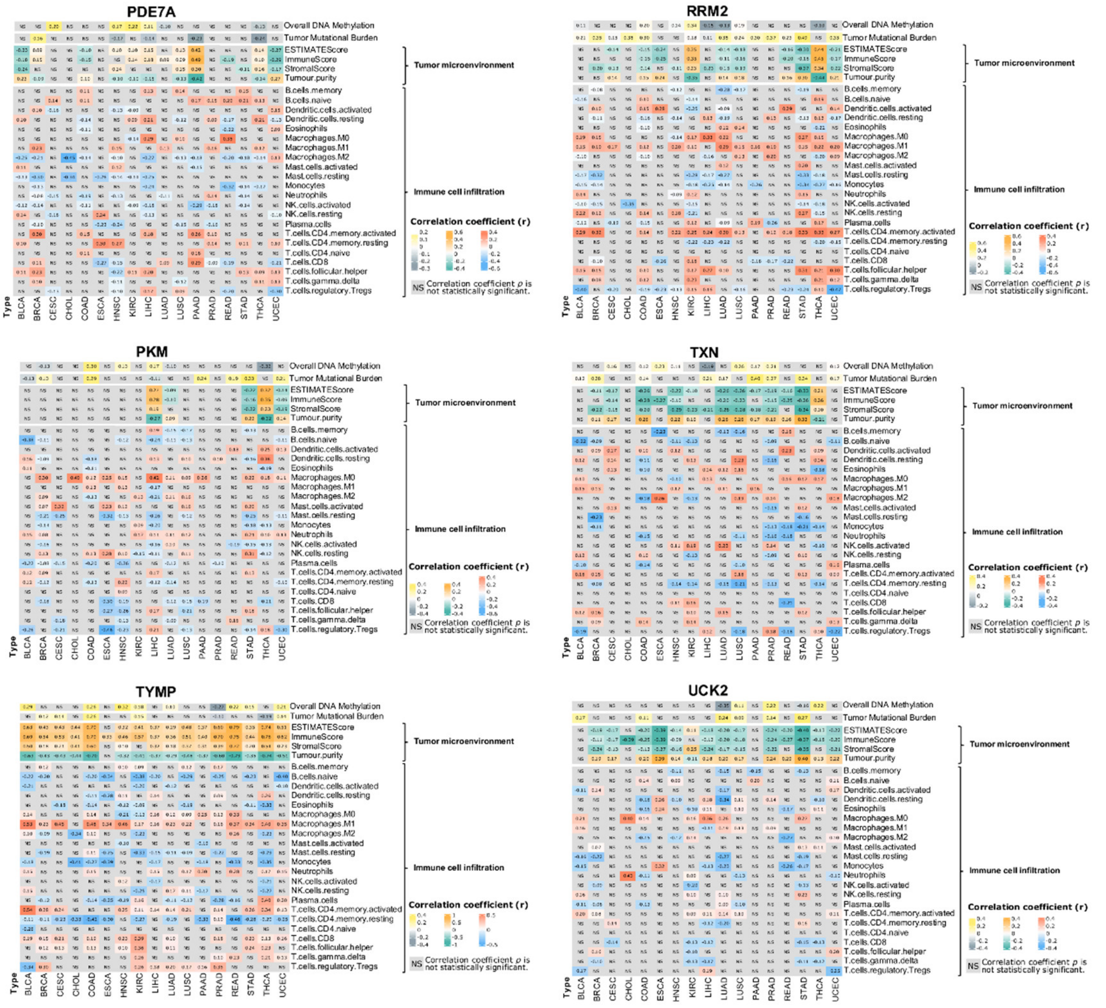

Supplementary Figure S6. Pan-cancer molecular features of purine metabolism-related upregulated gene signatures about tumor burden mutation, overall DNA methylation, tumor microenvironment, immune cell infiltration, and overall survival.

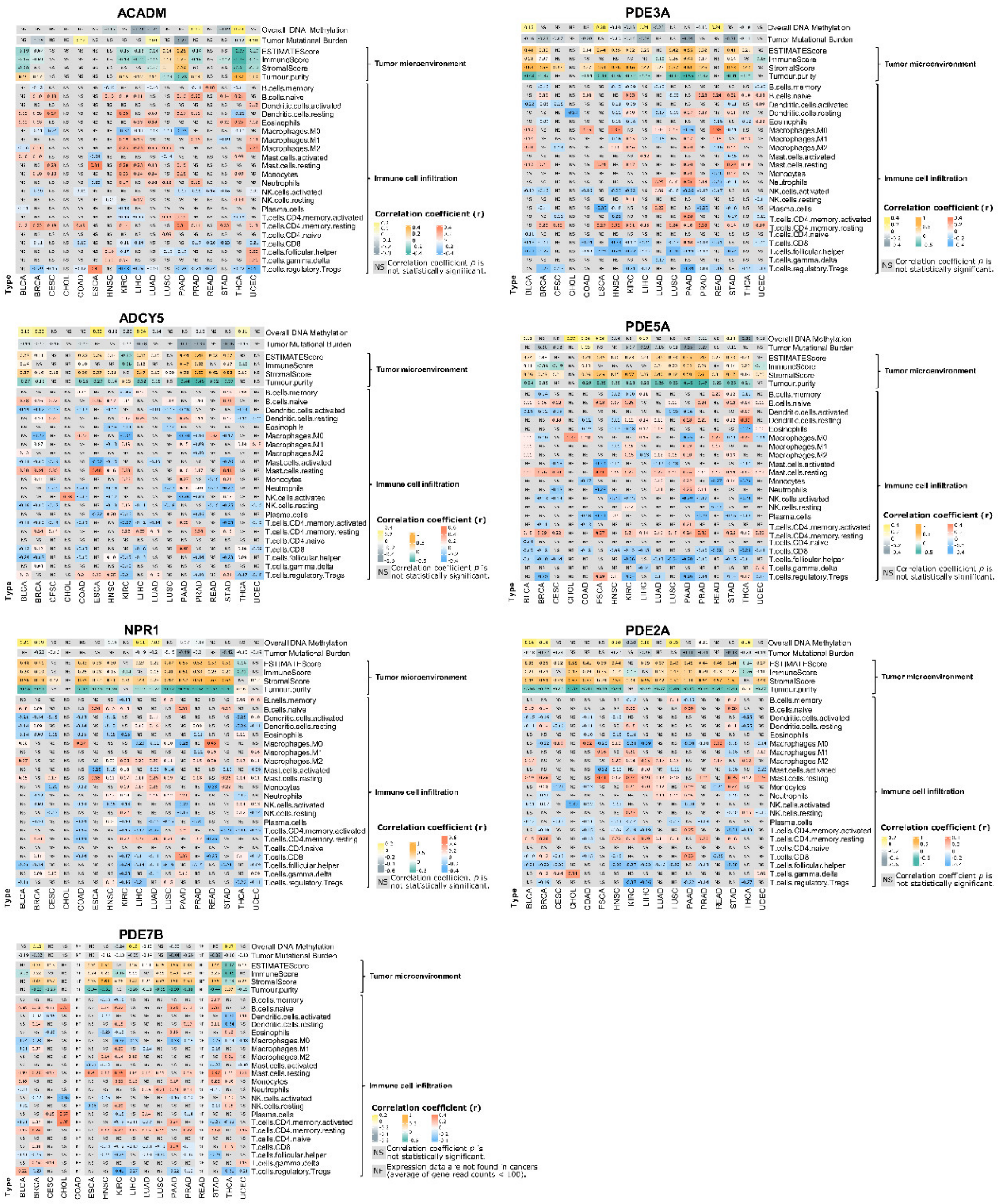

Supplementary Figure S7 Pan-cancer molecular features of purine metabolism-related downregulated gene signatures about tumor burden mutation, overall DNA methylation, tumor microenvironment, immune cell infiltration, and overall survival.

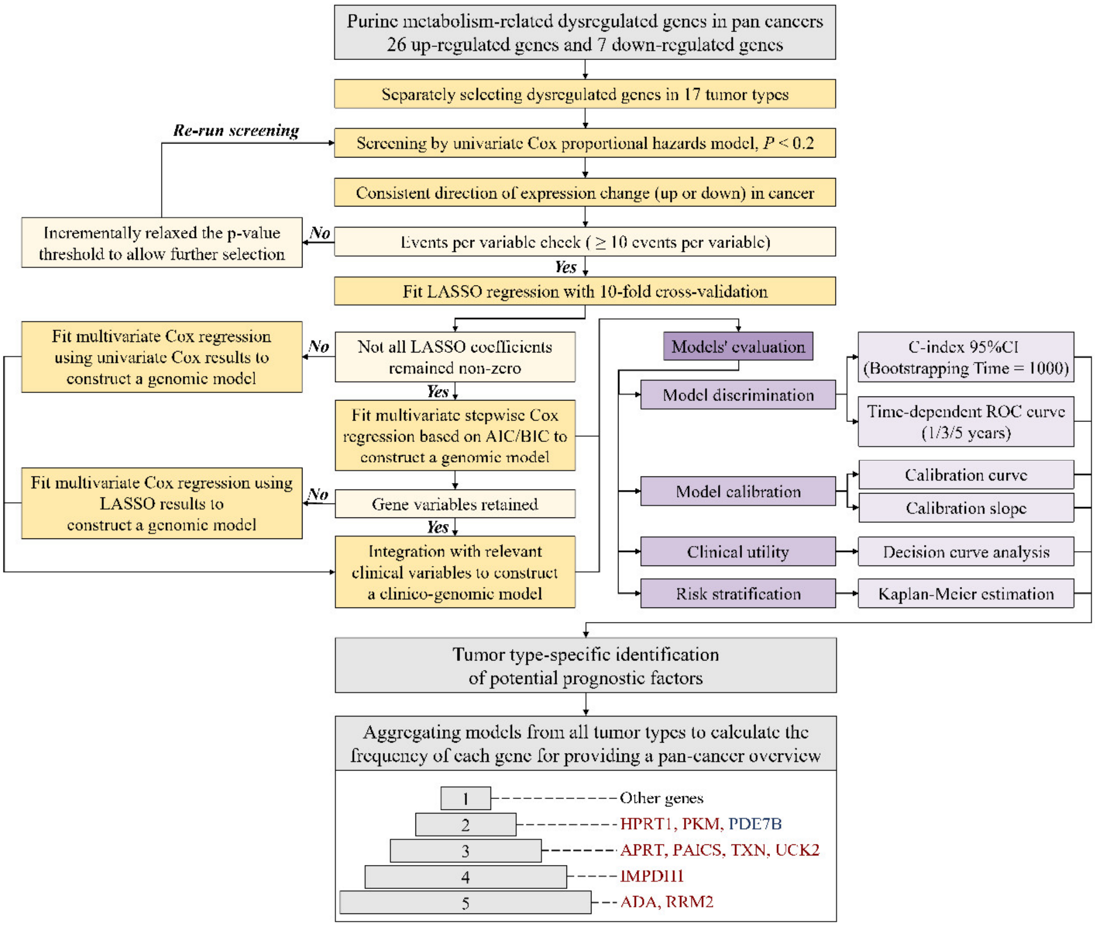

Supplementary Figure S8. Workflow for pan-cancer prognostic model construction and gene inclusion overview. This figure illustrates the comprehensive analytical workflow, from the identification of dysregulated purine metabolism-related genes to the construction and evaluation of tumor-specific genomic and clinico-genomic models, culminating in a pan-cancer overview of gene inclusion frequencies. Across the tumor-specific prognostic models, two genes, *ADA* and *RRM2*, were included in five different tumor types. *IMPDH1* was present in four tumor types. Four genes, *APRT*, *PAICS*, *TXN*, and *UCK2*, were each identified in three tumor types. Three genes, *HPRT1*, *PKM*, and *PDE7B*, occurred in two tumor types; among these, *PDE7B* was down-regulated. The remaining genes, including *ATIC*, *GART*, *HK2*, *HKDC1*, *NME1*, *NT5C3A*, *PPAT*, *TYMP*, *ACADM*, *ADCY5*, *PDE2A*, and *PDE5A*, appeared in only one tumor type. Red font indicates up-regulated genes, and blue font denotes down-regulated genes (*PDE7B* is highlighted in blue as it is a down-regulated gene frequently included across multiple tumor types). Detailed methodology is provided in the Methods section. **LASSO**, Least Absolute Shrinkage and Selection Operator; **AIC**, Akaike Information Criterion; **BIC**, Bayesian Information Criterion; **CI**, Confidence Interval; **ROC**, Receiver Operating Characteristic Curve.

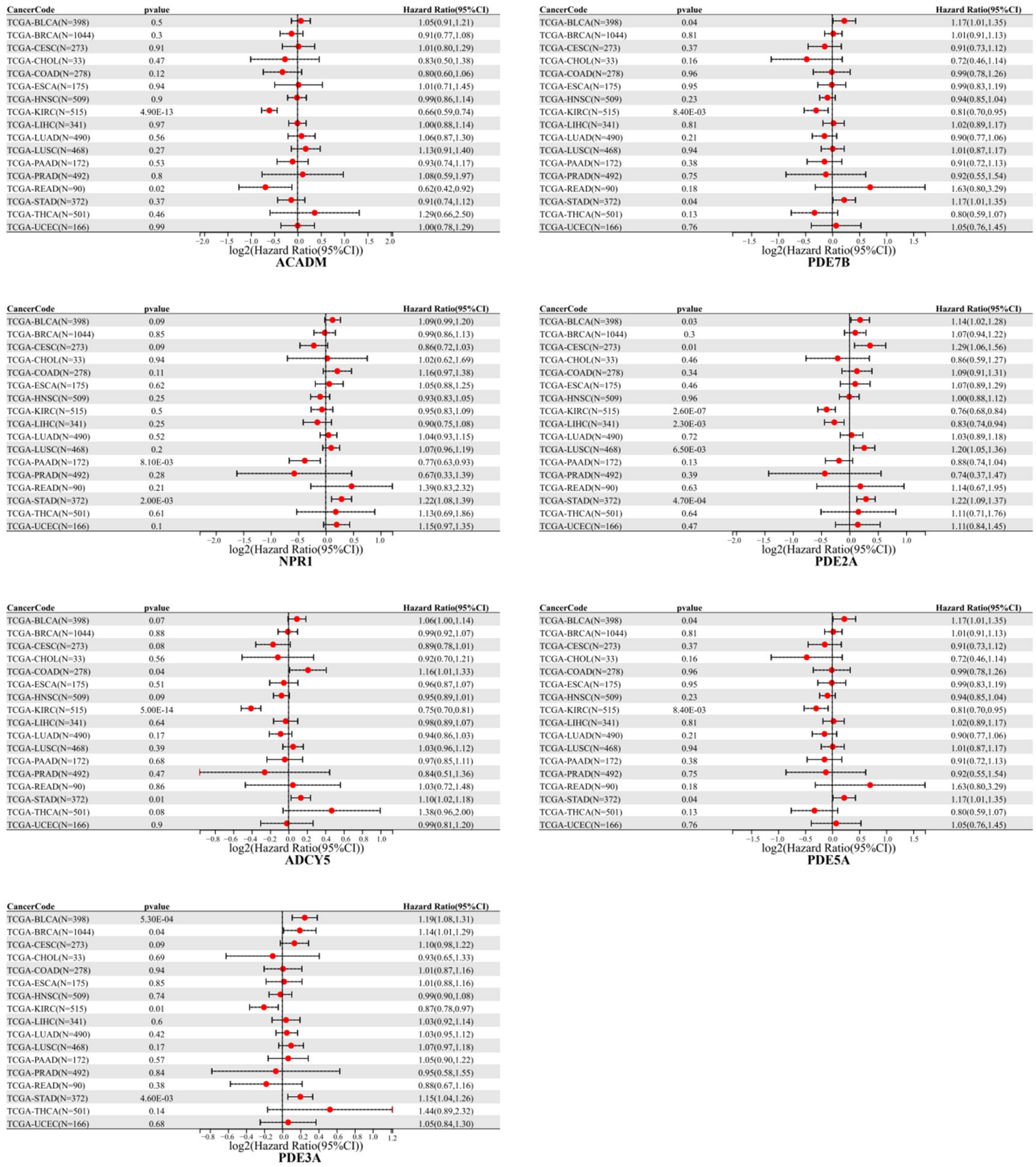

Supplementary Figure S9. Overall survival (OS) of down-regulated pan-cancer purine metabolism-related signatures. Forrest plots of prognostic values predicted in 17 cancer types. P values, hazard ratio (HR) and 95% confidence intervals (95%CI) were analyzed by Cox proportional hazards regression model.

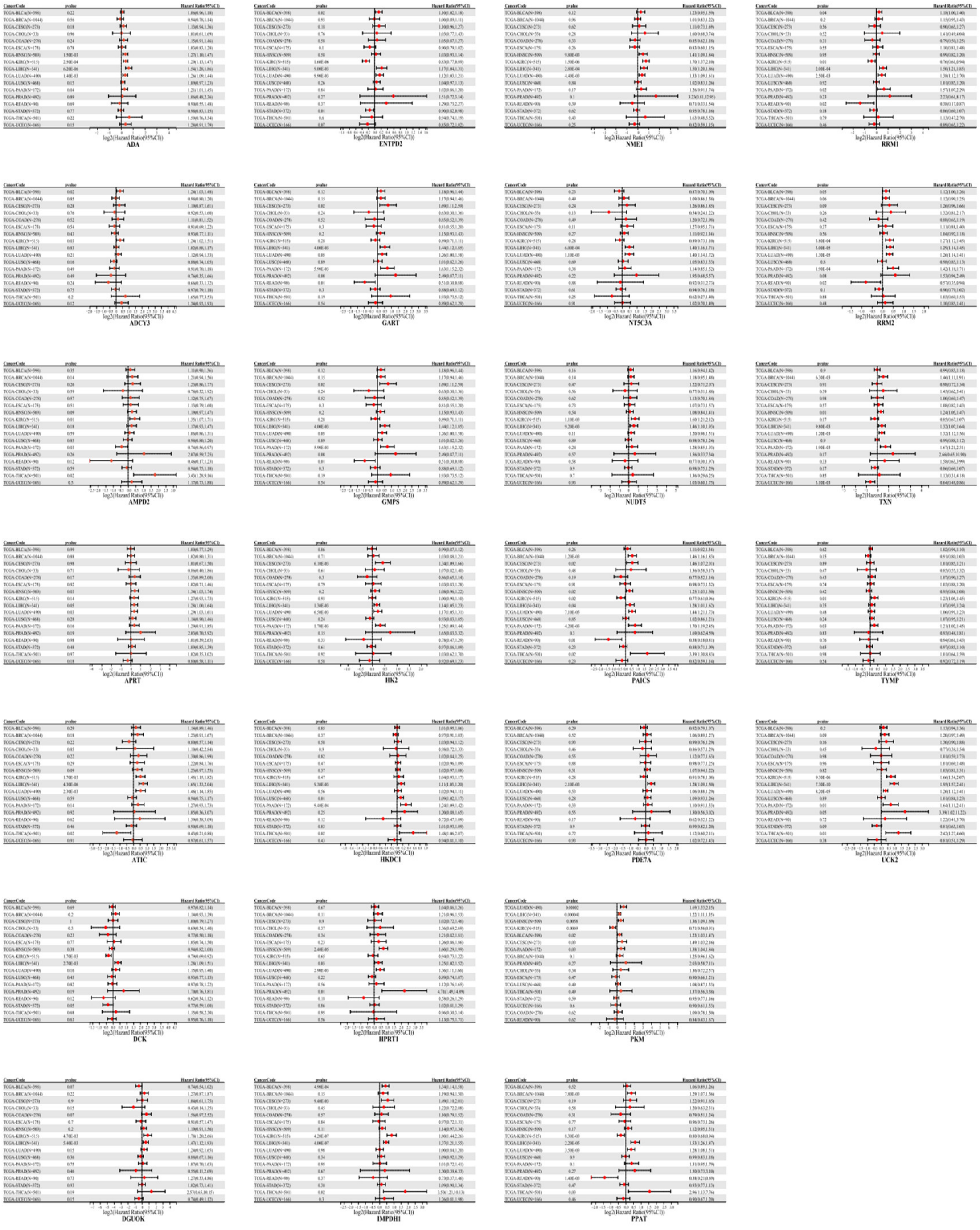

Supplementary Figure S10. Overall survival (OS) of and up-regulated pan-cancer purine metabolism-related signatures. Forrest plots of prognostic values predicted in 17 cancer types. P values, hazard ratio (HR) and 95% confidence intervals (95%CI) were analyzed by Cox proportional hazards regression model.

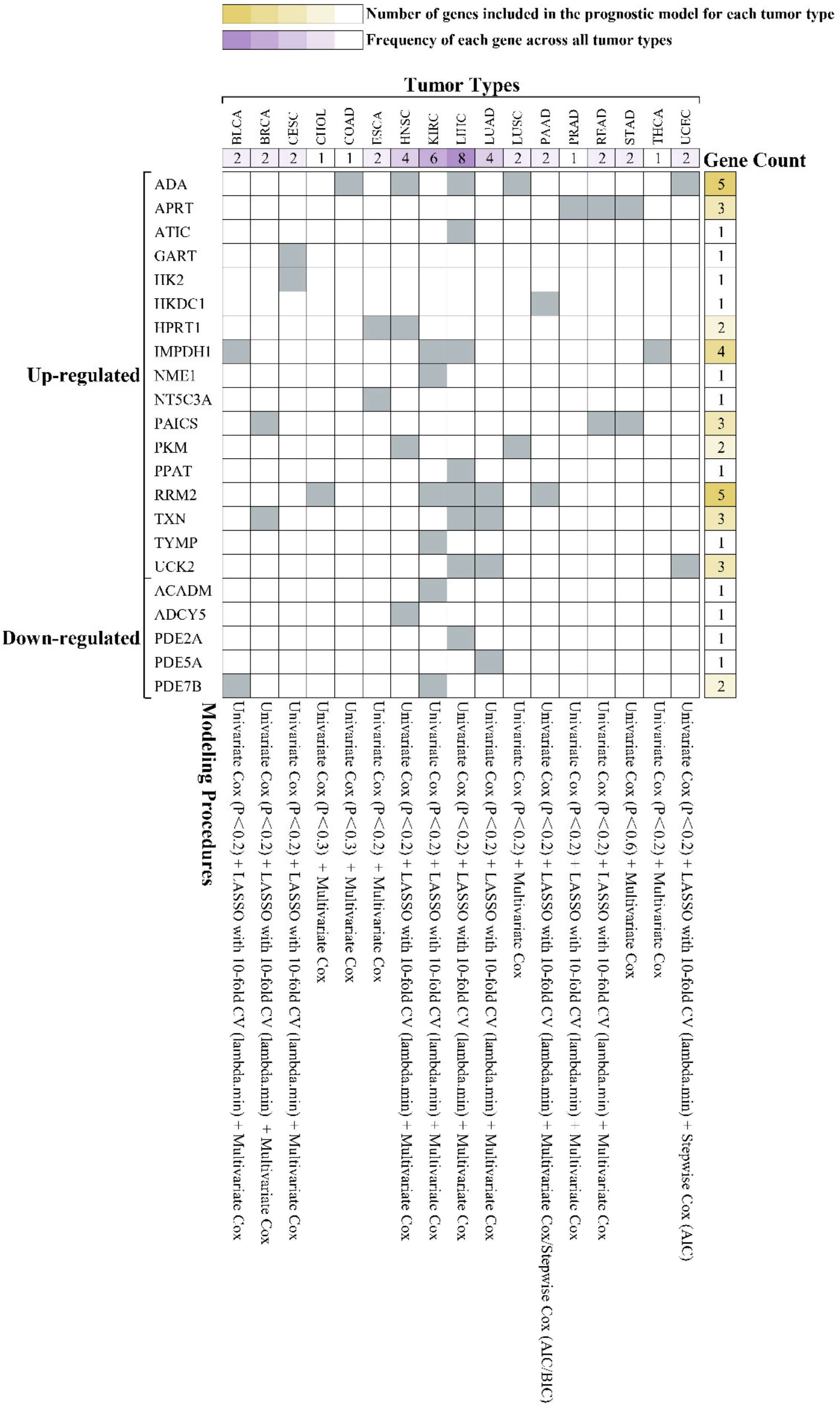

Supplementary Figure S11. Pan-cancer overview of prognostic gene signatures and their inclusion in tumor-specific models. This matrix displays the specific dysregulated purine metabolism-related genes (rows) included in the optimal prognostic model for each of the 17 tumor

types (columns). The rightmost 'Gene Count' column and its corresponding legend bar ('Frequency of each gene across all tumor types') quantify how often each gene was selected across the different tumor models. The 'Modeling Procedures' row at the bottom details the specific variable selection and model construction pipeline employed for each tumor type, which includes univariate Cox regression, LASSO regression with 10-fold cross-validation, and multivariate Cox regression (either direct or stepwise based on AIC or Bayesian BIC. Detailed methodology is provided in the Methods section and results are listed in the Supplementary Table S47. LASSO, Least Absolute Shrinkage and Selection Operator; CV, Cross Validation; AIC, Akaike Information Criterion; BIC, Bayesian Information Criterion.

***Genomic prognostic model for BRCA*:**
Univariate Cox (P < 0.2) + LASSO with 10-fold CV (lambda.min) + Multivariate Cox
Risk Score = 0.2478 * gene_PAICS + 0.1111 * gene_TXN

(A)

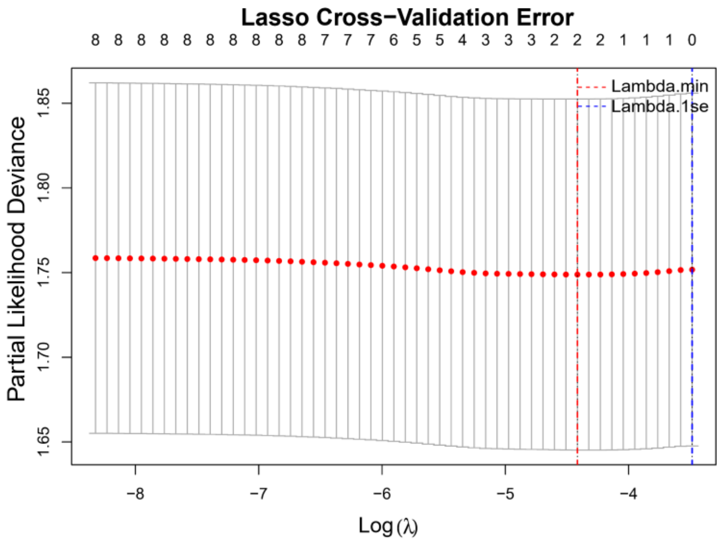

(B)

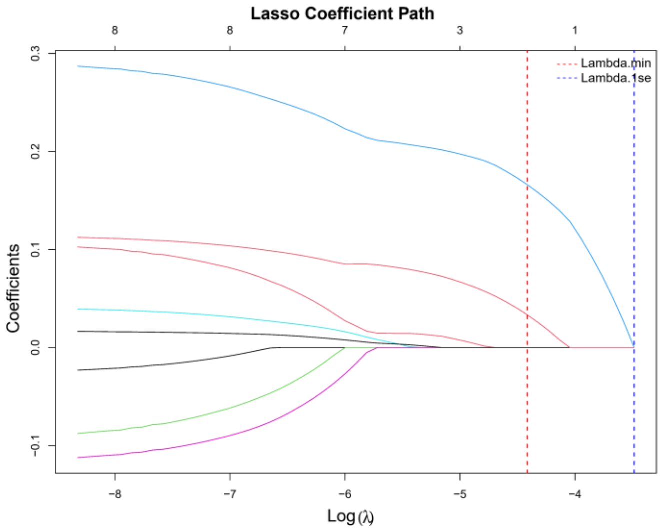

(C)

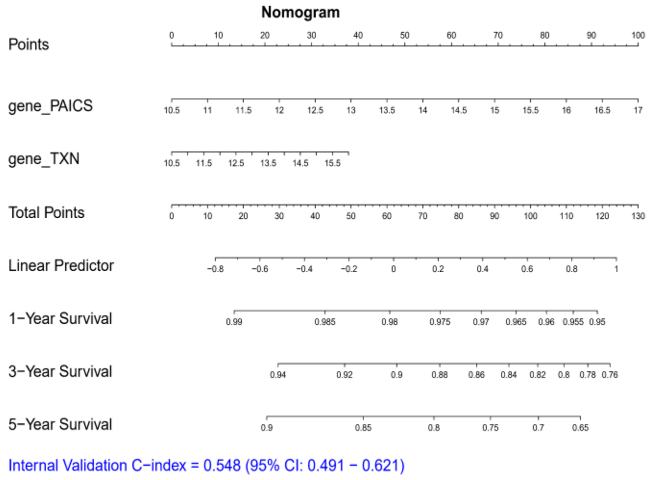

(D)

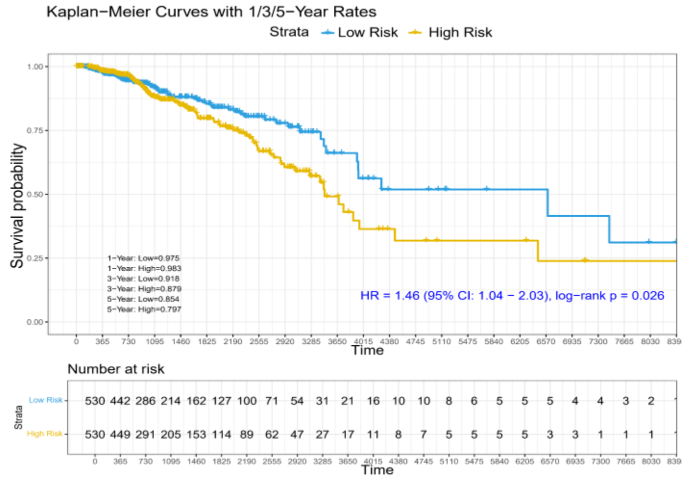

(E)

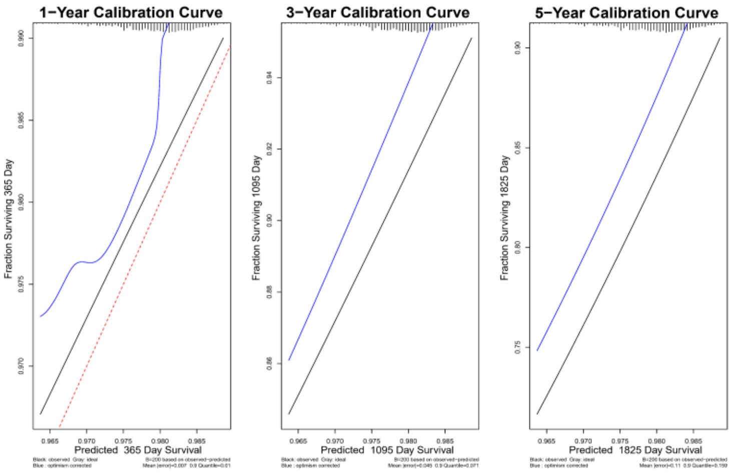

(F)

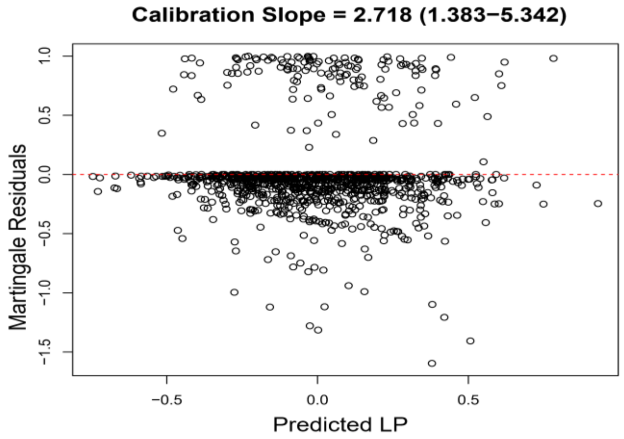

(G)

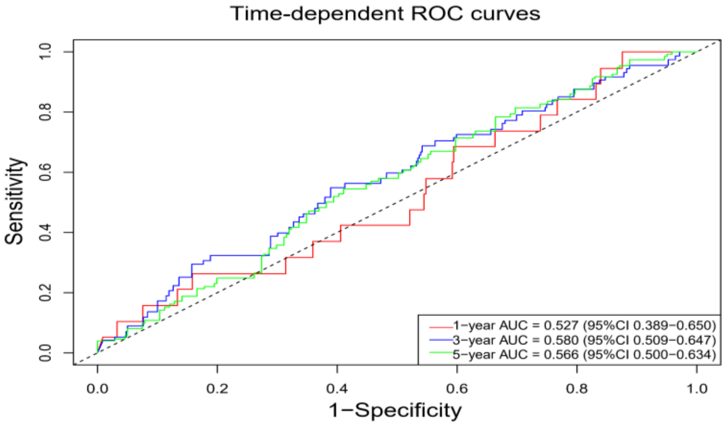

(H)

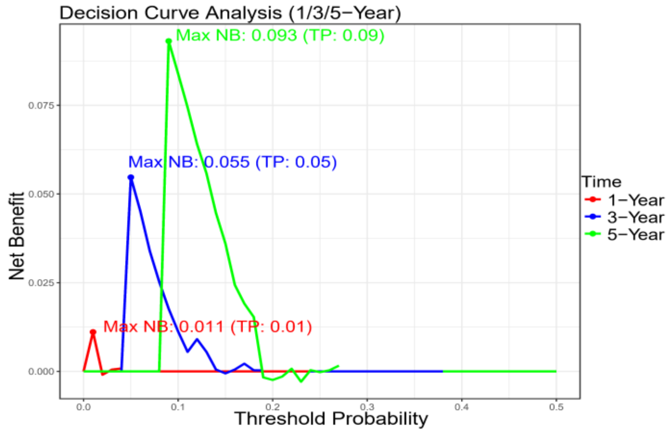

Clinico-genomic prognostic model for BRCA:

Univariate Cox (P < 0.2) + LASSO with 10-fold CV (lambda.min) + Multivariate Cox

Risk Score = 1.012 * risk_score_gene + 0.0367 * age + 1.0457 * stage + 0.0193 * race black or african american + -0.3684 * race unknow + -0.1394 * race white + -0.7625 * gender Male

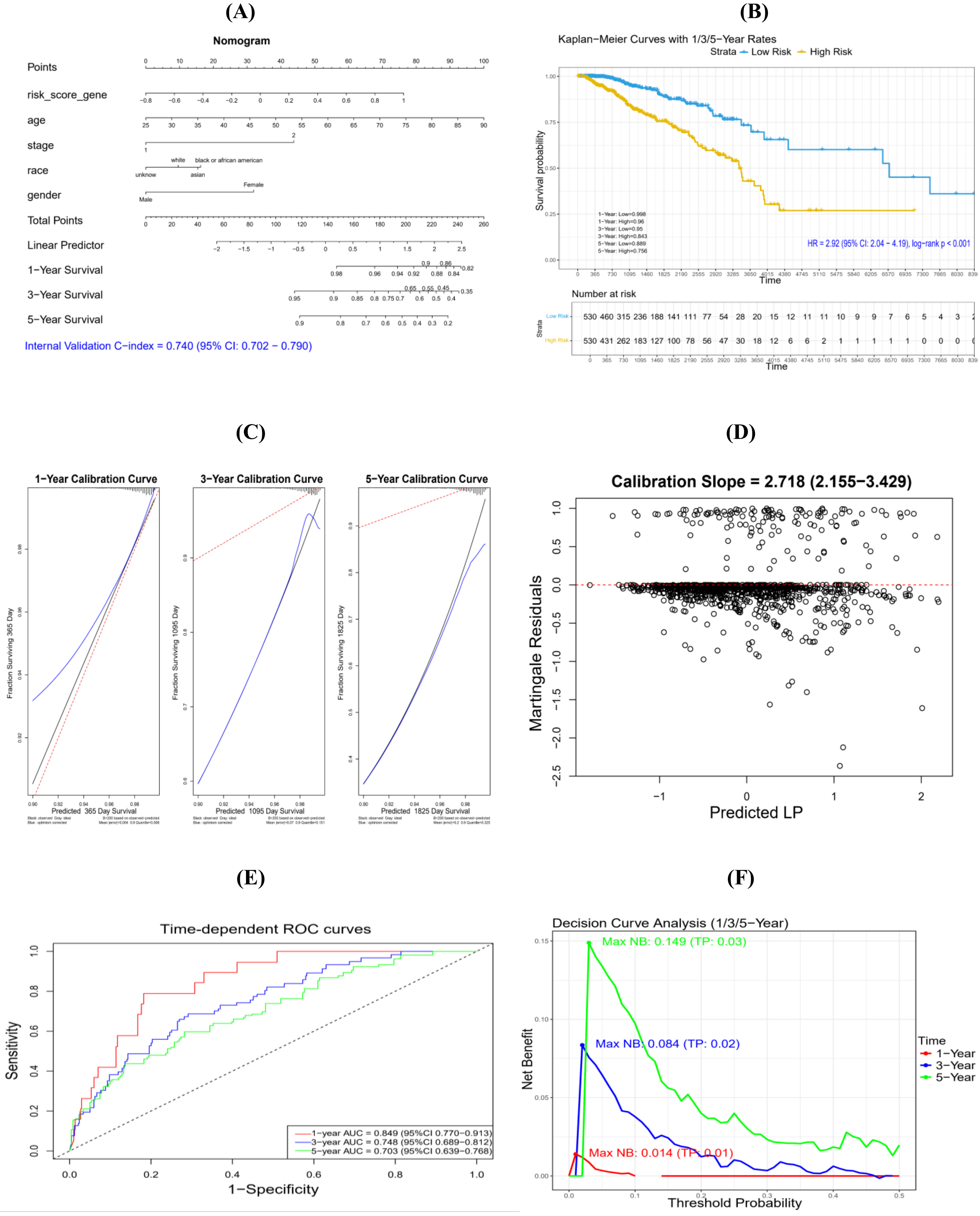

Supplementary Figure S12. Comprehensive evaluation of the prognostic models for BRCA. The models' construction followed the procedure: Univariate Cox (*P* <0.2) + LASSO with 10-fold CV (lambda.min) + Multivariate Cox. The resulting risk score formula of genomic model is: Risk Score = 0.2478 * gene_PAICS + 0.1111 * gene_TXN. The resulting risk score formula of integrated clinico-genomic model is: Risk Score = 1.012 * risk_score_gene + 0.0367 * age + 1.0457 * stage + 0.0193 * race black or african american + -0.3684 * race unknow + -0.1394 * race white + -0.7625 * gender Male.

(A) Lasso Cross-Validation Error Plot: Depicts the cross-validation error distribution across different lambda (λ) values, indicating the optimal lambda values (lambda.min and lambda.1se) for penalty parameter selection in LASSO regression. The best-performing model for this tumor type used lambda.min, with a λ value of 0.121.

(B) Lasso Coefficient Path: Illustrates how the coefficients of the selected genes change as the lambda (λ) regularization parameter varies, demonstrating the variable selection process.

(C) in genomic model figures and (A) in integrated clinico-genomic model figures: Nomogram for Survival Prediction: A graphical representation of the prognostic model, allowing for the visual estimation of 1-, 3-, and 5-year overall survival probabilities based on individual gene expression levels (and clinical features, if clinico-genomic model). The genomic model's internal validation C-index was 0.548 (95%CI: 0.491-0.621), integrated clinico-genomic model's internal validation C-index was 0.740 (95%CI: 0.702-0.790).

(D) in genomic model figures and (B) in integrated clinico-genomic model figures: Kaplan-Meier Survival Curves: Survival analysis demonstrated significant differences in overall survival between the high- and low-risk groups in both the genomic model (HR = 1.46, 95% CI: 1.04–2.03, log-rank *p* = 0.026) and the integrated clinico-genomic model (HR = 2.92, 95% CI: 2.04–4.19, log-rank *p* <0.001). The table below shows the number of patients at risk over time for each group.

(E) in genomic model figures and (C) in integrated clinico-genomic model figures: 1-, 3-, and 5-Year Calibration Curves: Assesses the agreement between the predicted

and observed overall survival probabilities at 1, 3, and 5 years, respectively. The diagonal dashed line represents perfect calibration.

(F) in genomic model figures and (D) in integrated clinico-genomic model figures: Calibration Slope Plot: Further evaluates the model's calibration, showing the relationship between predicted linear predictor and Martingale residuals. The calibration slope of genomic model was 2.718 (95% CI: 1.383–5.34). The calibration slope of integrated clinico-genomic model was 2.718 (95% CI: 2.155-3.429).

(G) in genomic model figures and (E) in integrated clinico-genomic model figures: Time-Dependent Receiver Operating Characteristic (ROC) Curves: Illustrates the discriminatory ability of the model over time. The area under the curve (AUC) values of genomic model were 0.527 (0.389-0.650), 0.580 (0.509-0.647), and 0.566 (0.500-0.634) for 1-, 3-, and 5-year survival, respectively. The AUC values of integrated clinico-genomic model were 0.849 (0.770-0.913), 0.748 (0.689-0.812), and 0.703 (0.639-0.768) for 1-, 3-, and 5-year survival, respectively.

(H) in genomic model figures and (F) in integrated clinico-genomic model figures: Decision Curve Analysis (DCA): Evaluates the clinical utility of the prognostic model by quantifying the net benefit across a range of threshold probabilities. The maximum net benefit (Max NB) and corresponding threshold probability (TP) for 1-, 3-, and 5-year survival in genomic model were: 1-year Max NB =0.011 (TP: 0.01); 3-year Max NB = 0.055 (TP: 0.55); 5-year Max NB = 0.093 (TP: 0.09). The Max NB and TP for 1-, 3-, and 5-year survival in integrated clinico-genomic model were: 1-year Max NB = 0.014 (TP: 0.01); 3-year Max NB = 0.84 (TP: 0.02); 5-year Max NB = 0.149 (TP: 0.03).

Genomic prognostic model for BLCA:
Univariate Cox (P < 0.2) + LASSO with 10-fold CV (lambda.min) + Multivariate Cox
Risk Score = 0.133 * gene_IMPDH1 + -0.0491 * gene_PDE7B

(A)

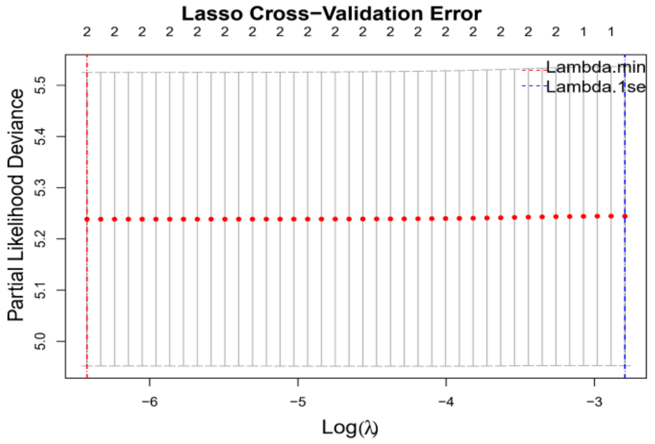

(B)

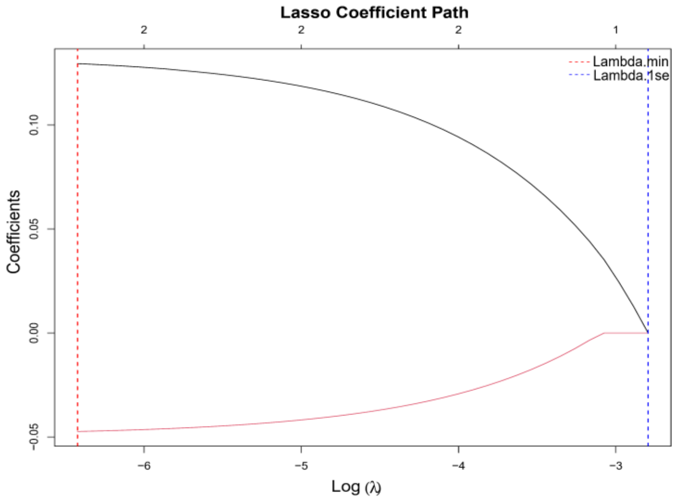

(C)

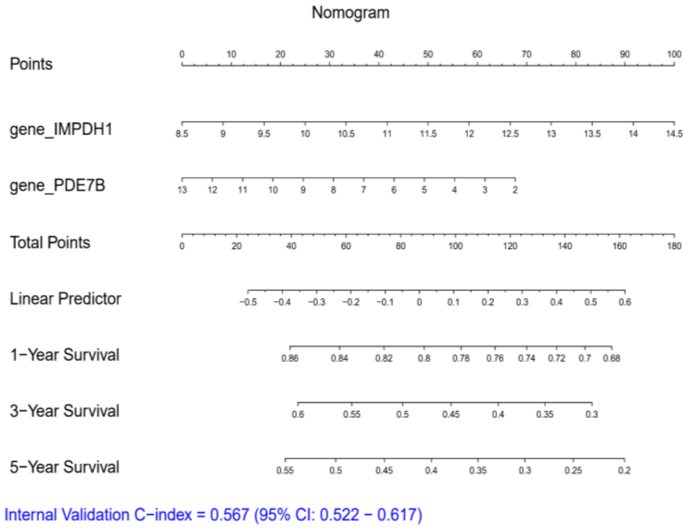

(D)

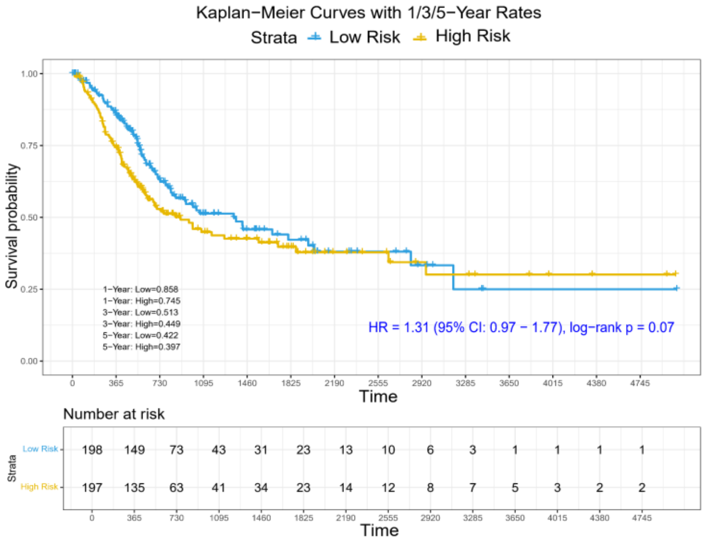

(E)

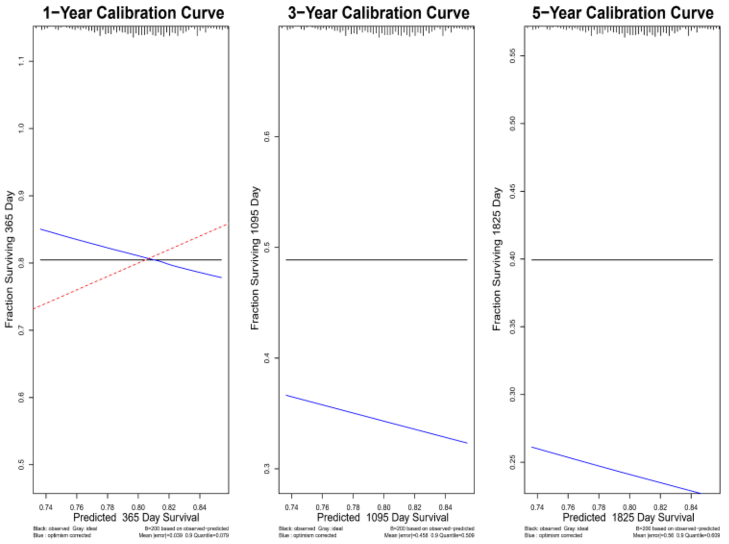

(F)

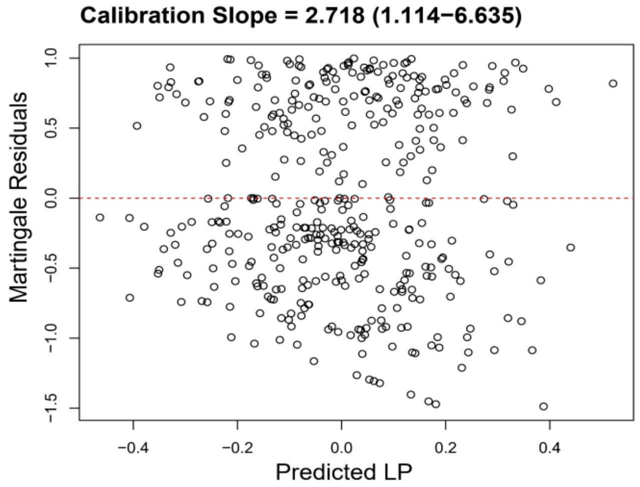

(G)

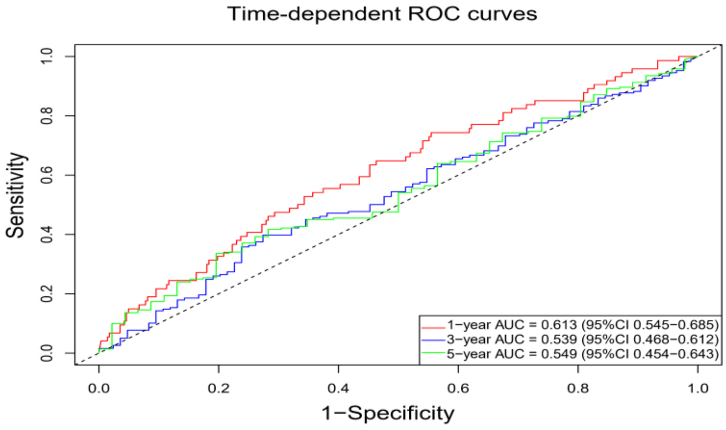

(H)

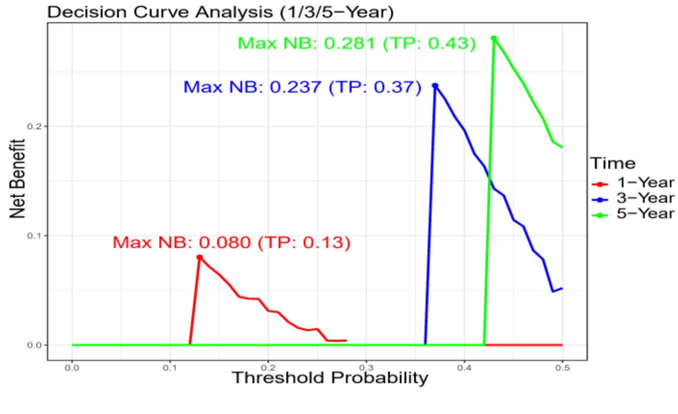

Clinico-genomic prognostic model for BLCA:

Univariate Cox (P < 0.2) + LASSO with 10-fold CV (lambda.min) + Multivariate Cox

Risk Score = 0.8562 * risk_score_gene + 0.0299 * age + 0.7467 * stage + 0.381 * race black or african american + 0.1053 * race unknow + 0.0295 * race white

(A)

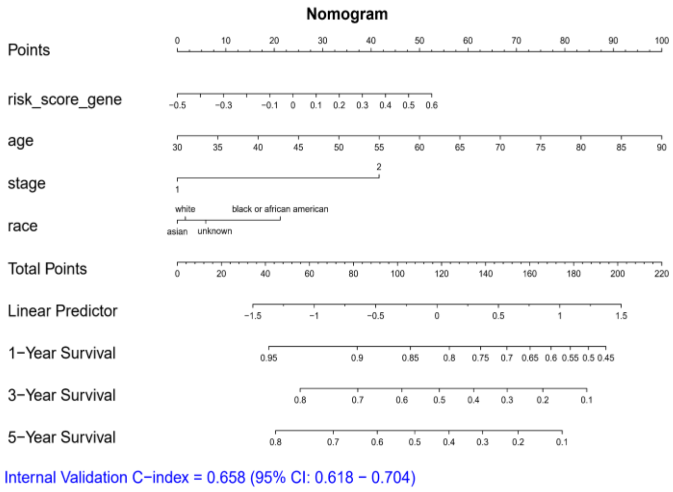

(B)

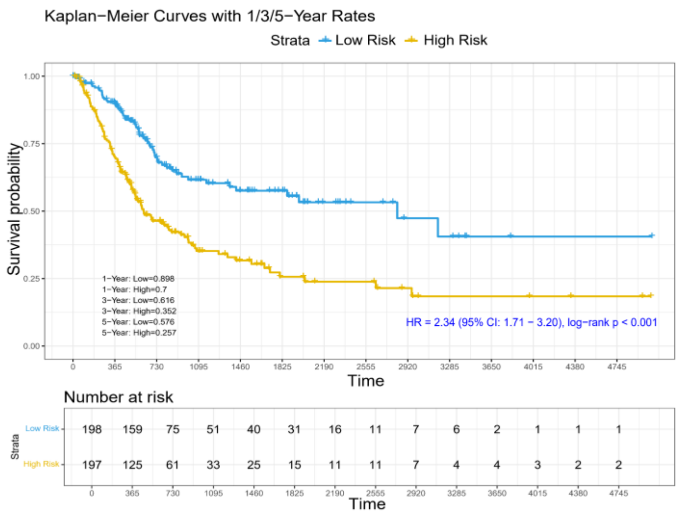

(C)

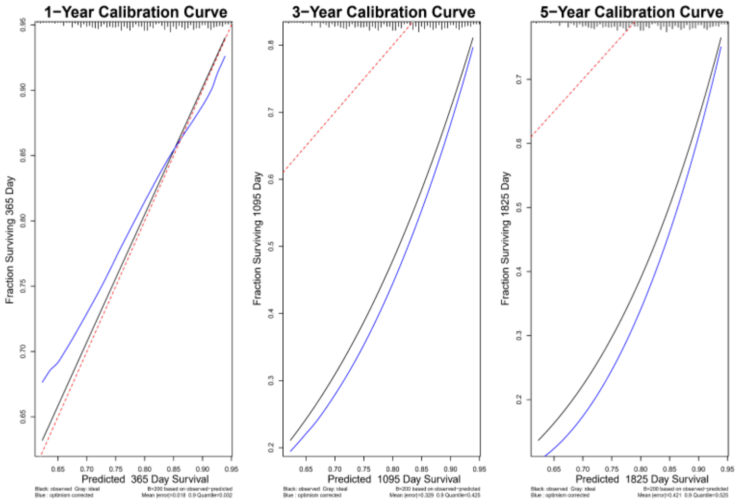

(D)

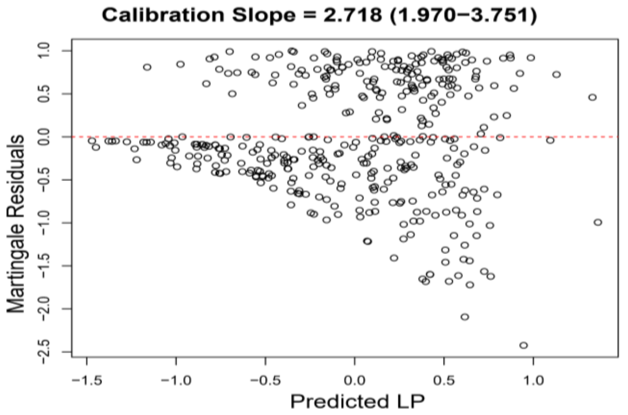

(E)

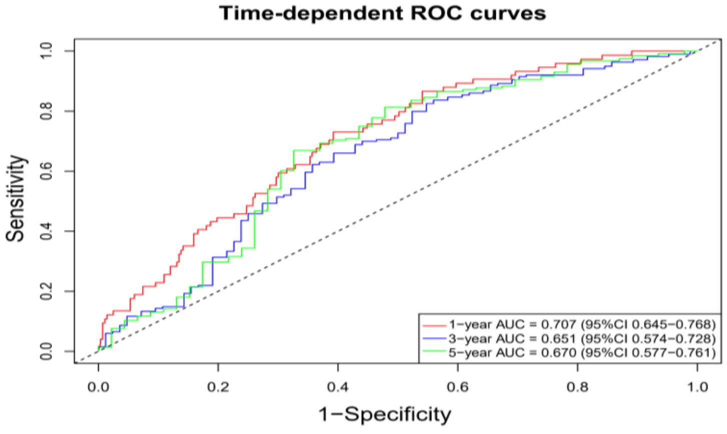

(F)

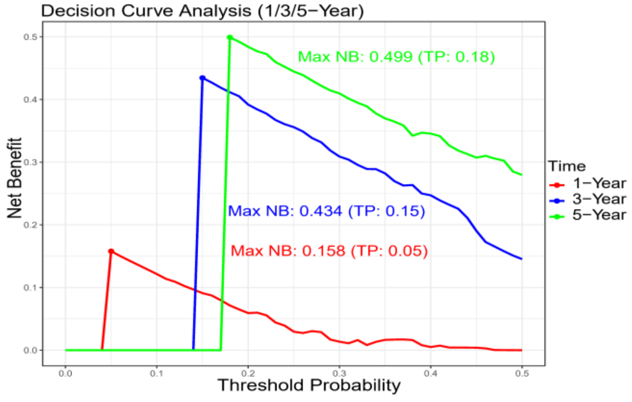

Supplementary Figure S13. Comprehensive evaluation of the prognostic models for BLCA.

The models' construction followed the procedure: Univariate Cox (*P* <0.2) + LASSO with 10-fold CV (lambda.min) + Multivariate Cox. The resulting risk score formula of genomic model is: Risk Score = 0.133 * gene_IMPDH1 + -0.0491 * gene_PDE7B. The resulting risk score formula of integrated clinico-genomic model is: Risk Score = 0.8562 * risk_score_gene + 0.0299 * age + 0.7467 * stage + 0.381 * race black or african american + 0.1053 * race unknow + 0.0295 * race white.

(A) Lasso Cross-Validation Error Plot: Depicts the cross-validation error distribution across different lambda (λ) values, indicating the optimal lambda values (lambda.min and lambda.1se) for penalty parameter selection in LASSO regression. The best-performing model for this tumor type used lambda.min, with a λ value of 0.002.

(B) Lasso Coefficient Path: Illustrates how the coefficients of the selected genes change as the lambda (λ) regularization parameter varies, demonstrating the variable selection process.

(C) in genomic model figures and (A) in integrated clinico-genomic model figures: Nomogram for Survival Prediction: A graphical representation of the prognostic model, allowing for the visual estimation of 1-, 3-, and 5-year overall survival probabilities based on individual gene expression levels (and clinical features, if clinico-genomic model). The genomic model's internal validation C-index was 0.567 (95%CI: 0.522-0.617), integrated clinico-genomic model's internal validation C-index was 0.658 (95%CI: 0.618–0.704).

(D) in genomic model figures and (B) in integrated clinico-genomic model figures: Kaplan-Meier Survival Curves: Compares the overall survival probabilities between patients stratified into high-risk and low-risk groups based on the prognostic model. A statistically significant difference was observed in the integrated clinico-genomic model (HR = 2.34, 95% CI: 1.71–3.20, log-rank p <0.001), whereas no significant difference was observed for the genomic model (HR = 1.31, 95% CI: 0.97–1.77, log-rank p = 0.07). The table below shows the number of patients at risk over time for each group.

(E) in genomic model figures and (C) in integrated clinico-genomic model figures:

1-, 3-, and 5-Year Calibration Curves: Assesses the agreement between the predicted and observed overall survival probabilities at 1, 3, and 5 years, respectively. The diagonal dashed line represents perfect calibration.

(F) in genomic model figures and (D) in integrated clinico-genomic model figures:

Calibration Slope Plot: Further evaluates the model's calibration, showing the relationship between predicted linear predictor and Martingale residuals. The calibration slope of genomic model was 2.718 (95% CI: 1.114-6.635). The calibration slope of integrated clinico-genomic model was 2.718 (95% CI: 1.970–3.751).

(G) in genomic model figures and (E) in integrated clinico-genomic model figures:

Time-Dependent Receiver Operating Characteristic (ROC) Curves: Illustrates the discriminatory ability of the model over time. The area under the curve (AUC) values of genomic model were 0.613 (0.545-0.685), 0.539 (0.468-0.612), and 0.549 (0.454-0.643) for 1-, 3-, and 5-year survival, respectively. The AUC values of integrated clinico-genomic model were 0.707 (0.645-0.768), 0.651 (0.574-0.728), and 0.670 (0.577-0.761) for 1-, 3-, and 5-year survival, respectively.

(H) in genomic model figures and (F) in integrated clinico-genomic model figures:

Decision Curve Analysis (DCA): Evaluates the clinical utility of the prognostic model by quantifying the net benefit across a range of threshold probabilities. The maximum net benefit (Max NB) and corresponding threshold probability (TP) for 1-, 3-, and 5-year survival in genomic model were: 1-year Max NB =0.080 (TP: 0.13); 3-year Max NB = 0.237 (TP: 0.37); 5-year Max NB = 0.281 (TP: 0.43). The Max NB and TP for 1-, 3-, and 5-year survival in integrated clinico-genomic model were: 1-year Max NB =0.158 (TP: 0.05); 3-year Max NB = 0.434 (TP: 0.15); 5-year Max NB = 0.499 (TP: 0.18).

***Genomic prognostic model for CESC*:**

Univariate Cox (P < 0.2) + LASSO with 10-fold CV (lambda.min) + Multivariate Cox

Risk Score = 0.4018 * gene_GART + 0.3136 * gene_HK2

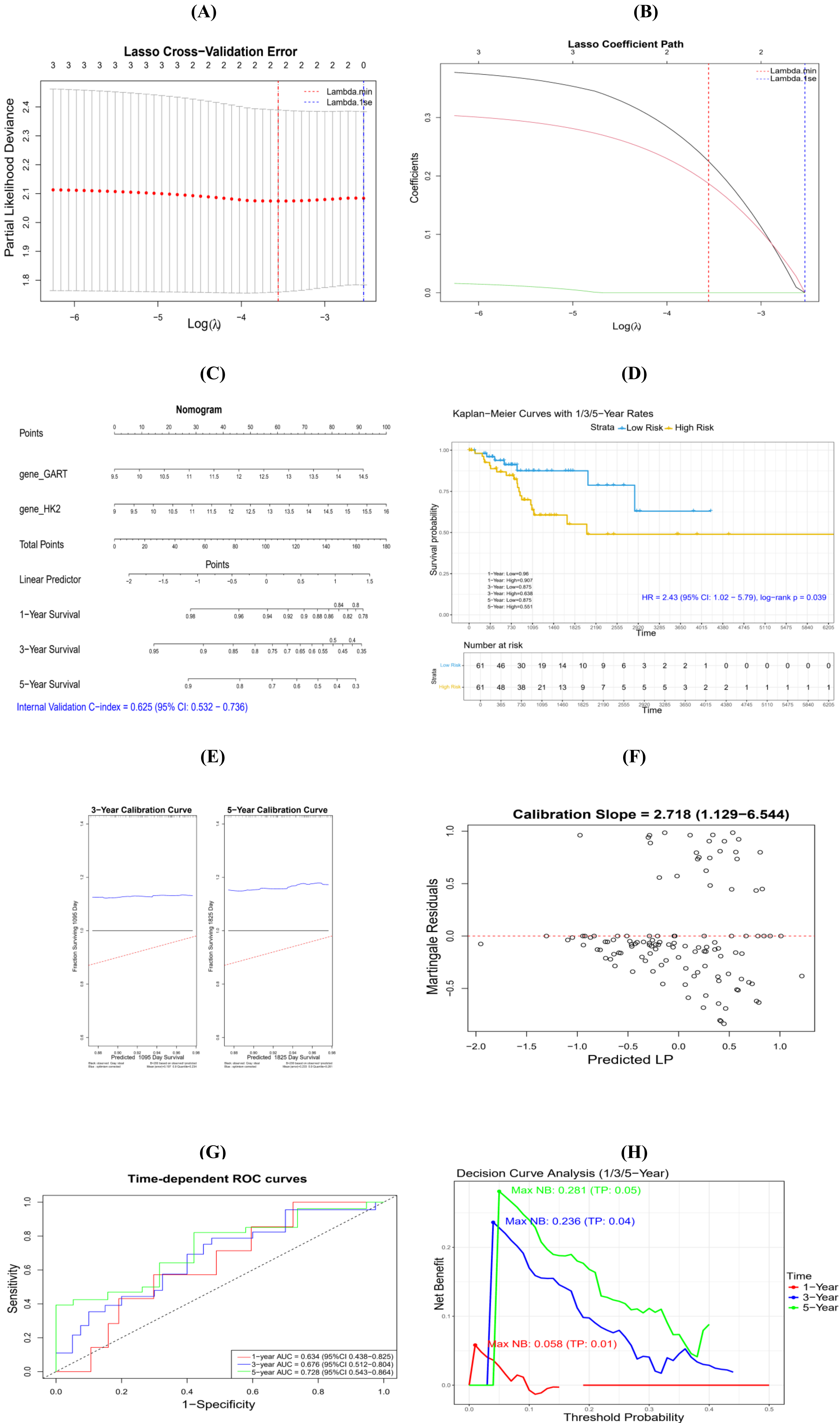

<u>Clinico-genomic prognostic model for CESC</u>:

Univariate Cox (P < 0.2) + LASSO with 10-fold CV (lambda.min) + Multivariate Cox

Risk Score = 1.0918 * risk_score_gene + 0.0301 * age + 1.7039 * stage + -3.8464 * race asian + -6.0833 * race black or african american + -4.9102 * race unknow + -4.4431 * race white

(A)

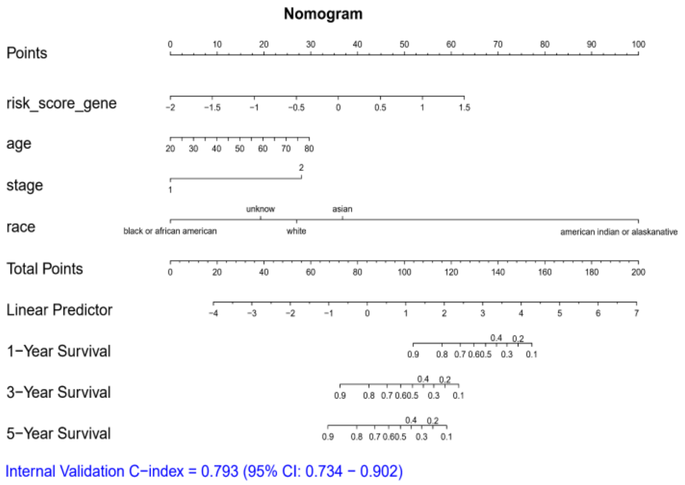

(B)

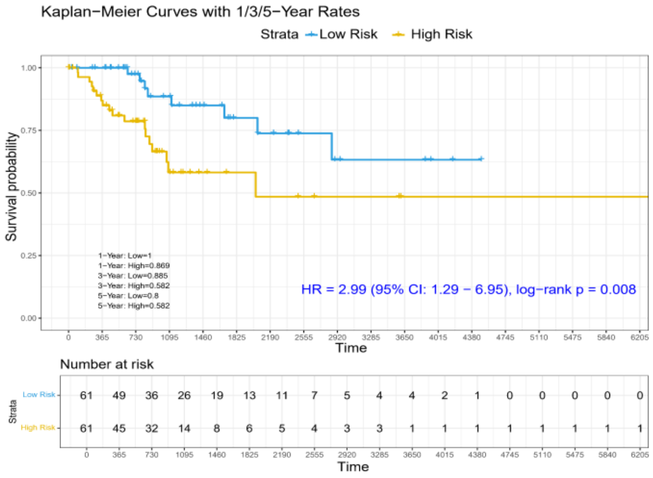

(C)

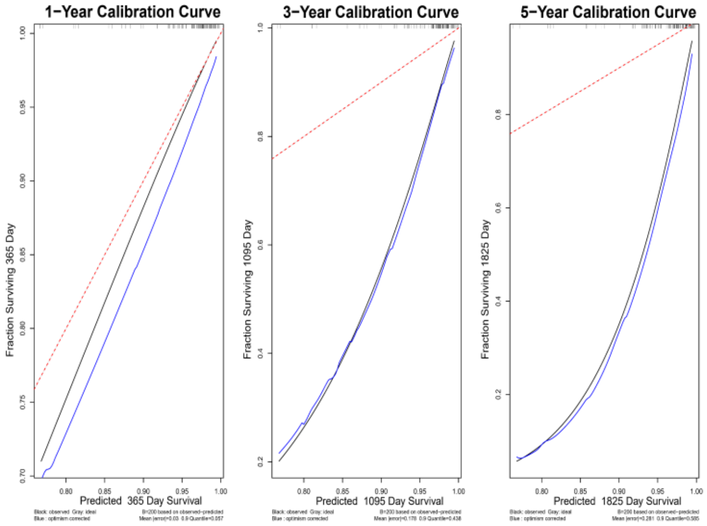

(D)

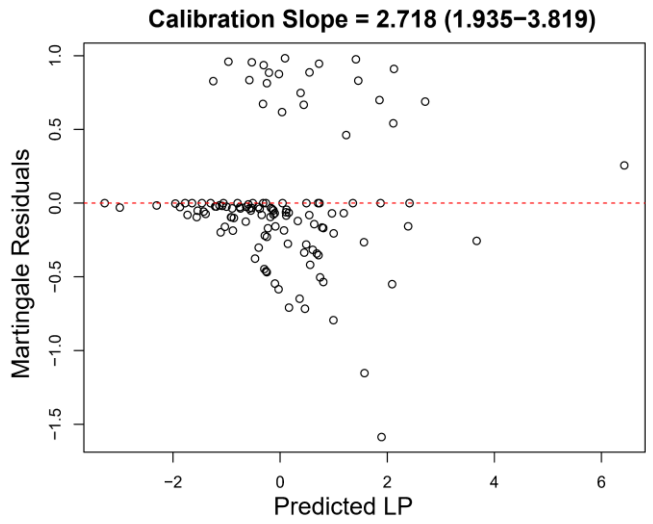

(E)

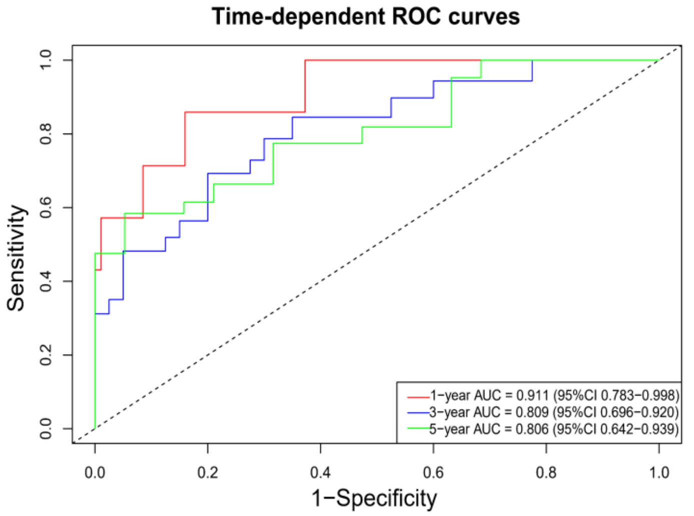

(F)

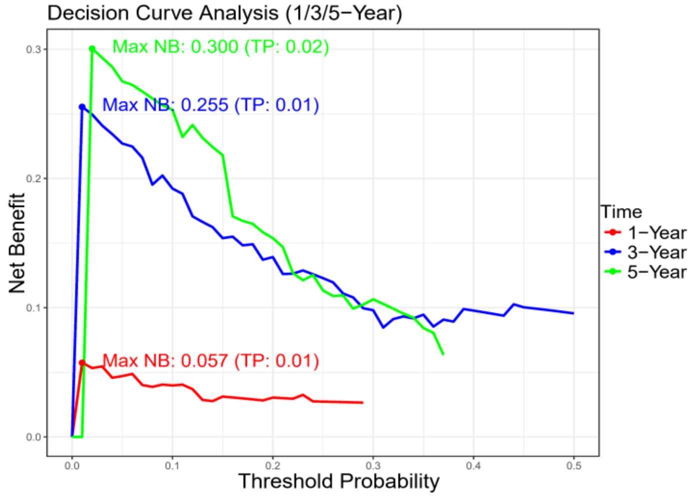

Supplementary Figure S14. Comprehensive evaluation of the prognostic models for CESC.

The models' construction followed the procedure: Univariate Cox (*P* <0.2) + LASSO with 10-fold CV (lambda.min) + Multivariate Cox. The resulting risk score formula of genomic model is: Risk Score = 0.4018 * gene_GART + 0.3136 * gene_HK2. The resulting risk score formula of integrated clinico-genomic model is: Risk Score = 1.0918 * risk_score_gene + 0.0301 * age + 1.7039 * stage + -3.8464 * race asian + -6.0833 * race black or african american + -4.9102 * race unknow + -4.4431 * race white.

(A) Lasso Cross-Validation Error Plot: Depicts the cross-validation error distribution across different lambda (λ) values, indicating the optimal lambda values (lambda.min and lambda.1se) for penalty parameter selection in LASSO regression. The best-performing model for this tumor type used lambda.min, with a λ value of 0.028.

(B) Lasso Coefficient Path: Illustrates how the coefficients of the selected genes change as the lambda (λ) regularization parameter varies, demonstrating the variable selection process.

(C) in genomic model figures and (A) in integrated clinico-genomic model figures: Nomogram for Survival Prediction: A graphical representation of the prognostic model, allowing for the visual estimation of 1-, 3-, and 5-year overall survival probabilities based on individual gene expression levels (and clinical features, if clinico-genomic model). The genomic model's internal validation C-index was 0.625 (95%CI: 0.532-0.736), integrated clinico-genomic model's internal validation C-index was 0.793 (95%CI: 0.734-0.902).

(D) in genomic model figures and (B) in integrated clinico-genomic model figures: Kaplan-Meier Survival Curves: Survival analysis demonstrated significant differences in overall survival between the high- and low-risk groups in both the genomic model (HR = 2.43, 95% CI: 1.02–5.79, log-rank $p = 0.039$) and the integrated clinico-genomic model (HR = 2.99, 95% CI: 1.29–6.95, log-rank $p = 0.008$). The table below shows the number of patients at risk over time for each group.

(E) in genomic model figures and (C) in integrated clinico-genomic model figures:

1-, 3-, and 5-Year Calibration Curves: Assesses the agreement between the predicted and observed overall survival probabilities at 1, 3, and 5 years, respectively. The diagonal dashed line represents perfect calibration.

(F) in genomic model figures and (D) in integrated clinico-genomic model figures: Calibration Slope Plot: Further evaluates the model's calibration, showing the relationship between predicted linear predictor and Martingale residuals. The calibration slope of genomic model was 2.718 (95% CI: 1.129-6.544). The calibration slope of integrated clinico-genomic model was 2.718 (95% CI: 1.935-3.819).

(G) in genomic model figures and (E) in integrated clinico-genomic model figures: Time-Dependent Receiver Operating Characteristic (ROC) Curves: Illustrates the discriminatory ability of the model over time. The area under the curve (AUC) values of genomic model were 0.634 (0.438-0.825), 0.676 (0.512-0.804), and 0.728 (0.543-0.864) for 1-, 3-, and 5-year survival, respectively. The AUC values of integrated clinico-genomic model were 0.911 (0.783-0.998), 0.809 (0.696-0.920), and 0.806 (0.642-0.939) for 1-, 3-, and 5-year survival, respectively.

(H) in genomic model figures and (F) in integrated clinico-genomic model figures: Decision Curve Analysis (DCA): Evaluates the clinical utility of the prognostic model by quantifying the net benefit across a range of threshold probabilities. The maximum net benefit (Max NB) and corresponding threshold probability (TP) for 1-, 3-, and 5-year survival in genomic model were: 1-year Max NB =0.058 (TP: 0.01); 3-year Max NB = 0.236 (TP: 0.04); 5-year Max NB = 0.281 (TP: 0.05). The Max NB and TP for 1-, 3-, and 5-year survival in integrated clinico-genomic model were: 1-year Max NB =0.057 (TP: 0.01); 3-year Max NB = 0.255 (TP: 0.01); 5-year Max NB = 0.300 (TP: 0.02).

Genomic prognostic model_for CHOL:

Univariate Cox (P < 0.3) + Multivariate Cox

Risk Score = 0.2673 * gene_RRM2

(A)

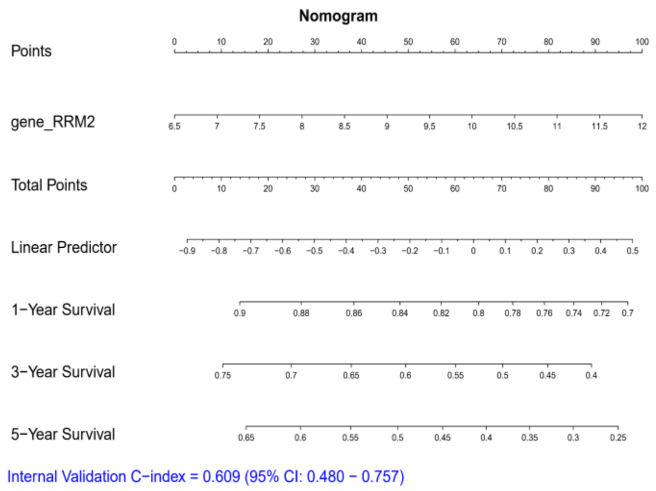

(B)

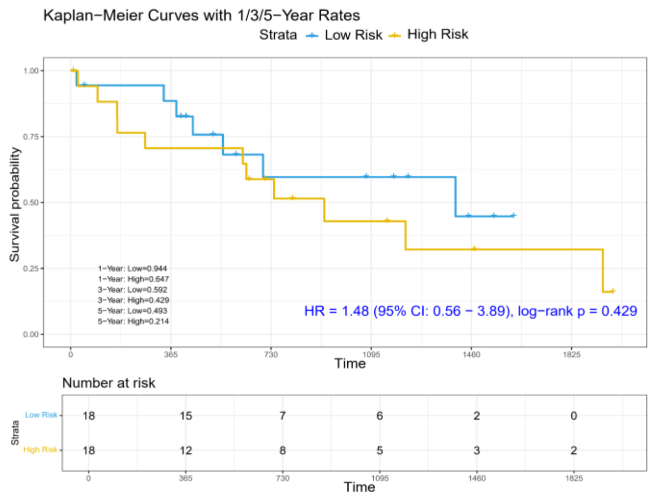

(C)

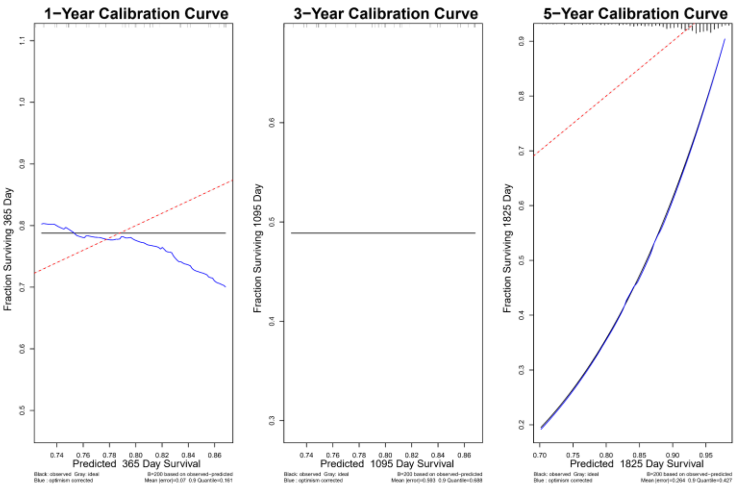

(D)

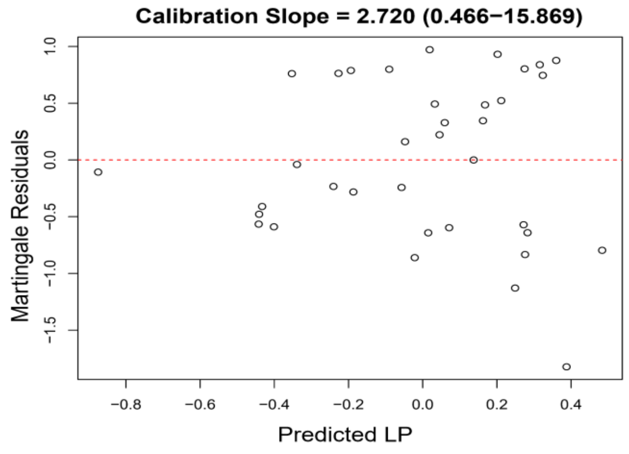

(E)

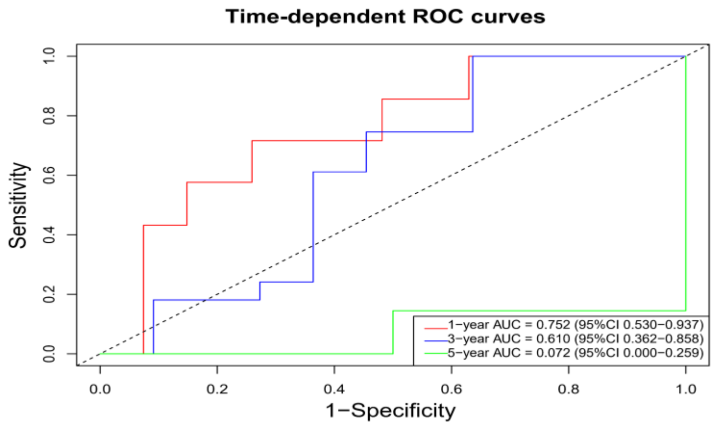

(F)

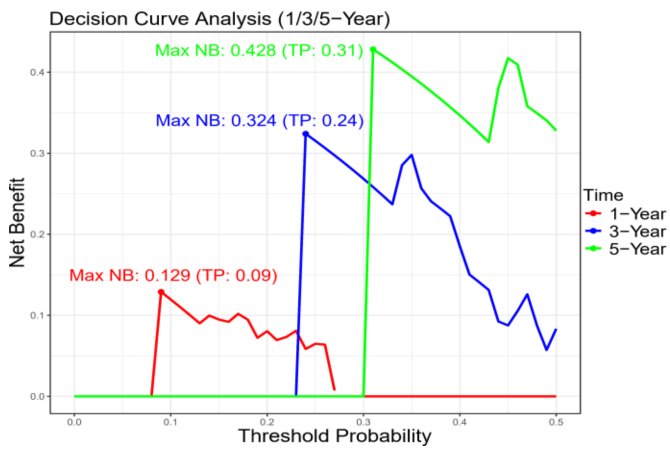

Clinico-genomic prognostic model for CHOL:

Univariate Cox (P < 0.3) + Multivariate Cox

Risk Score = 1.1981 * risk_score_gene + 0.0117 * age + 0.8274 * stage + -0.1113 * race black or african american + -1.0097 * race white + 0.2459 * gender Male

(A)

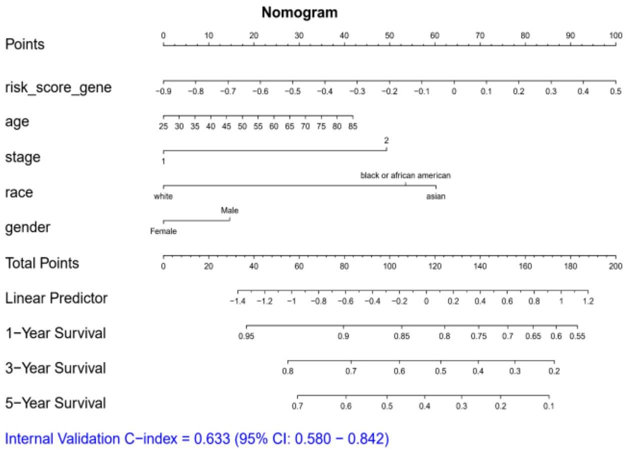

(B)

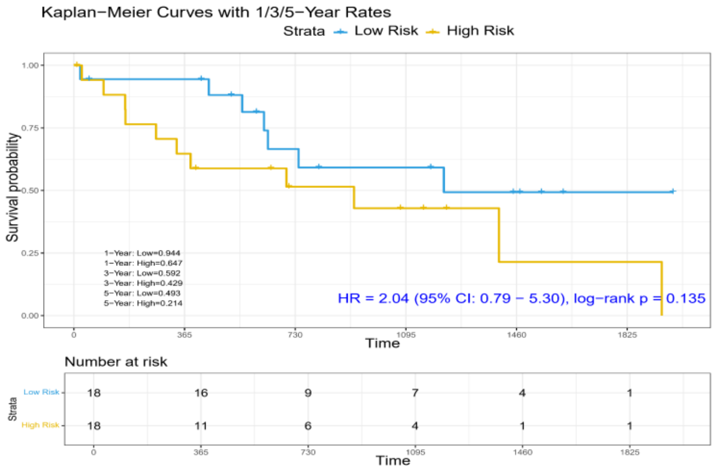

(C)

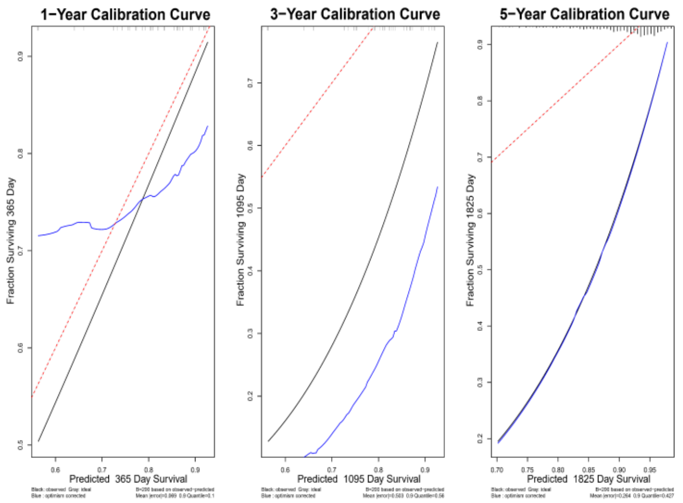

(D)

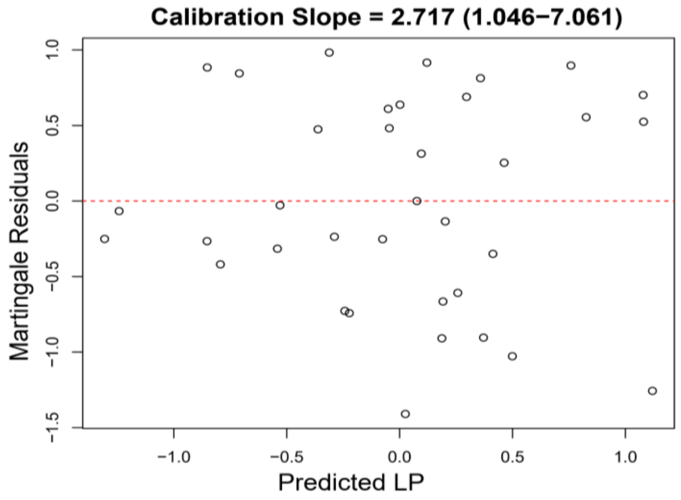

(E)

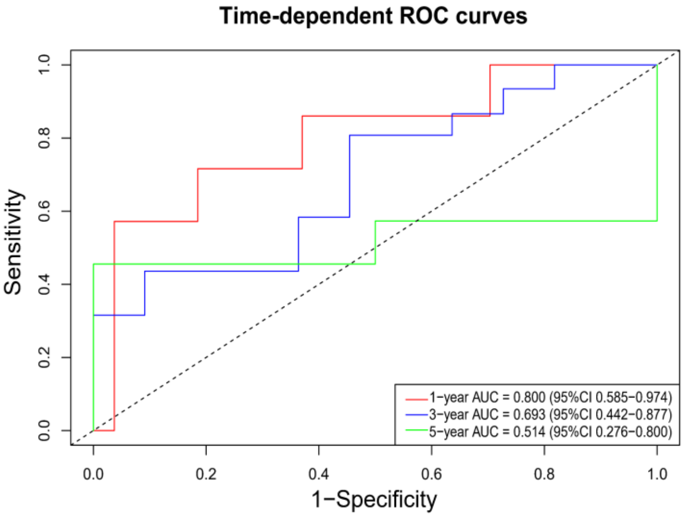

(F)

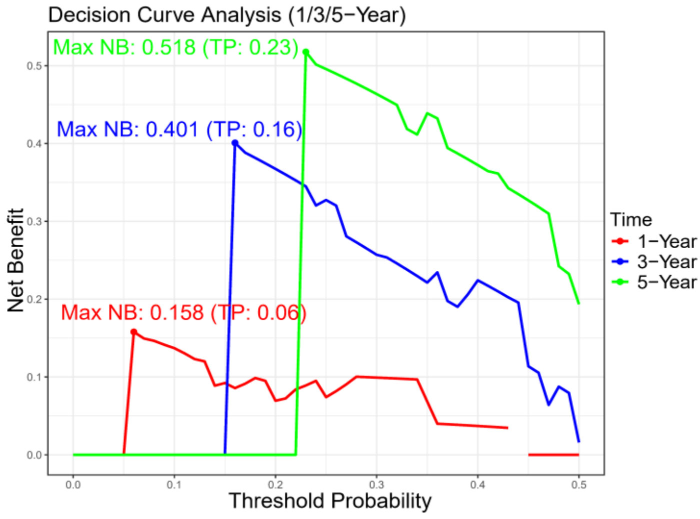

Supplementary Figure S15. Comprehensive evaluation of the prognostic models for CHOL.

The models' construction followed the procedure: Univariate Cox (*P* <0.3) + Multivariate Cox. The resulting risk score formula of genomic model is: Risk Score = 0.2673 * gene_RRM2. The resulting risk score formula of integrated clinico-genomic model is: Risk Score = 1.1981 * risk_score_gene + 0.0117 * age + 0.8274 * stage + -0.1113 * race black or african american + -1.0097 * race white + 0.2459 * gender Male.

(A) Nomogram for Survival Prediction: A graphical representation of the prognostic model, allowing for the visual estimation of 1-, 3-, and 5-year overall survival probabilities based on individual gene expression levels (and clinical features, if clinico-genomic model). The genomic model's internal validation C-index was 0.609 (95%CI: 0.480-0.757), integrated clinico-genomic model's internal validation C-index was 0.633 (95%CI: 0.580-0.842).

(B) Kaplan-Meier Survival Curves: Compares the overall survival probabilities between patients stratified into high-risk and low-risk groups based on the prognostic model. No statistically significant difference was observed in the genomic model (HR = 1.48, 95% CI: 0.56–3.89, log-rank $p = 0.429$) or the integrated clinico-genomic model (HR = 2.04, 95% CI: 0.79–5.30, log-rank $p = 0.135$). The table below shows the number of patients at risk over time for each group.

(C) 1-, 3-, and 5-Year Calibration Curves: Assesses the agreement between the predicted and observed overall survival probabilities at 1, 3, and 5 years, respectively. The diagonal dashed line represents perfect calibration.

(D) Calibration Slope Plot: Further evaluates the model's calibration, showing the relationship between predicted linear predictor and Martingale residuals. The calibration slope of genomic model was 2.720 (95% CI: 0.466-15.869). The calibration slope of integrated clinico-genomic model was 2.717 (95% CI: 1.046-7.061).

(E) Time-Dependent Receiver Operating Characteristic (ROC) Curves: Illustrates the discriminatory ability of the model over time. The area under the curve (AUC) values of genomic model were 0.752 (0.530-0.937), 0.610 (0.362-0.858), and 0.072 (0.000-0.259) for 1-, 3-, and 5-year survival, respectively. The AUC values of integrated

clinico-genomic model were 0.800 (0.585-0.974), 0.693 (0.442-0.877), and 0.514 (0.276-0.800) for 1-, 3-, and 5-year survival, respectively.

(F) Decision Curve Analysis (DCA): Evaluates the clinical utility of the prognostic model by quantifying the net benefit across a range of threshold probabilities. The maximum net benefit (Max NB) and corresponding threshold probability (TP) for 1-, 3-, and 5-year survival in genomic model were: 1-year Max NB =0.129 (TP: 0.09); 3-year Max NB = 0.324 (TP: 0.24); 5-year Max NB = 0.428 (TP: 0.31). The Max NB and TP for 1-, 3-, and 5-year survival in integrated clinico-genomic model were: 1-year Max NB =0.158 (TP: 0.06); 3-year Max NB = 0.401 (TP: 0.16); 5-year Max NB = 0.518 (TP: 0.23).

Genomic prognostic model for COAD:

Univariate Cox (P < 0.3) + Multivariate Cox

Risk Score = 0.1113 * gene_ADA

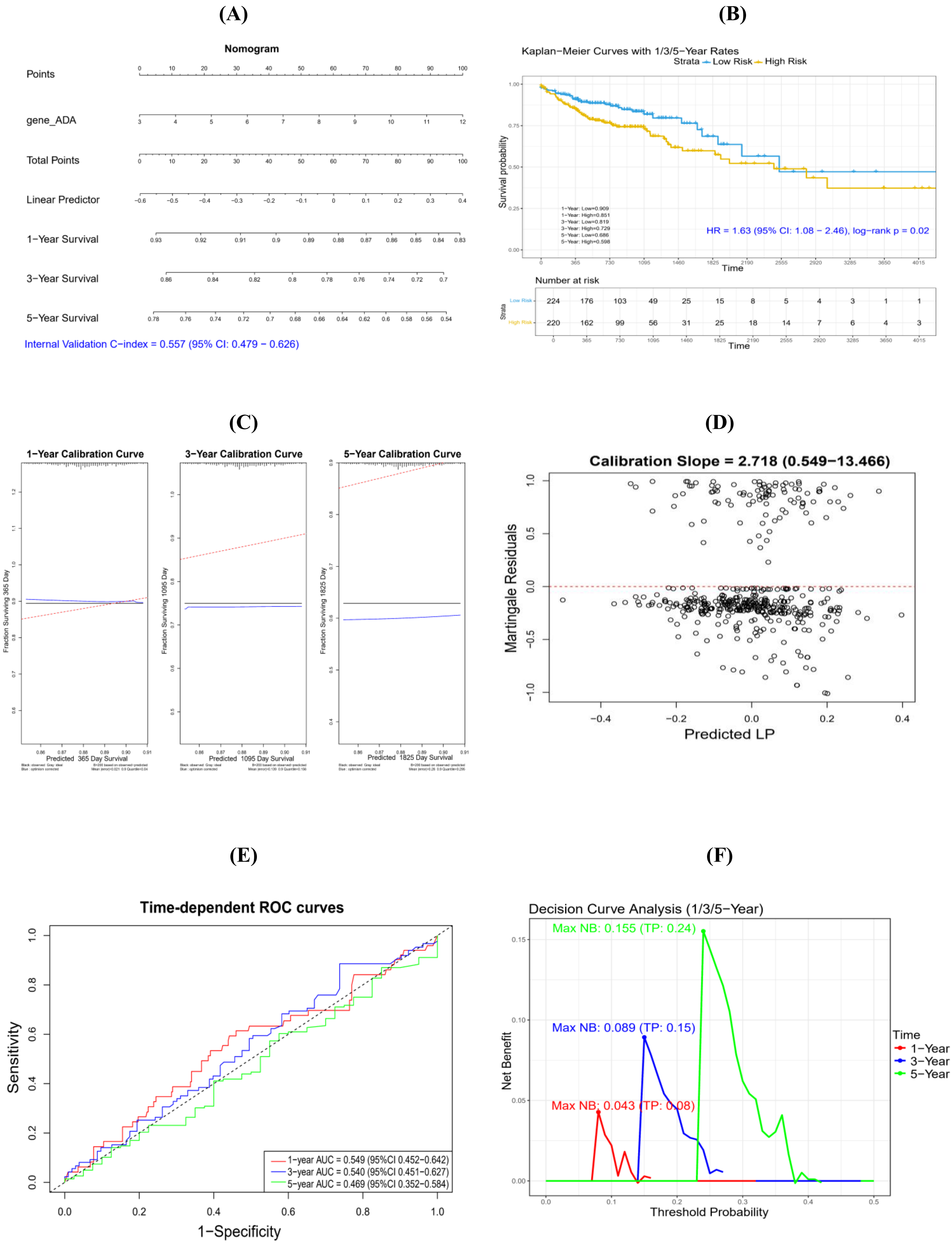

Clinico-genomic prognostic model for COAD:

Univariate Cox (P < 0.3) + Multivariate Cox

Risk Score = 0.7243 * risk_score_gene + 0.0357 * age + 1.2543 * stage + 13.1236 * race asian + 12.5608 * race black or african american + 12.4635 * race unknow + 12.3975 * race white + -0.0956 * gender Male

(A)

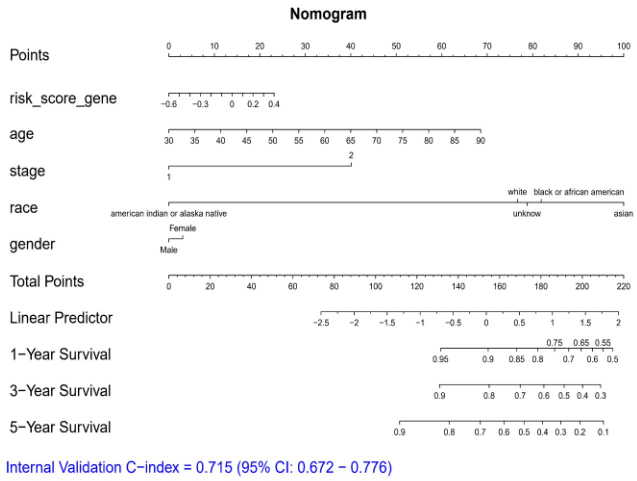

(B)

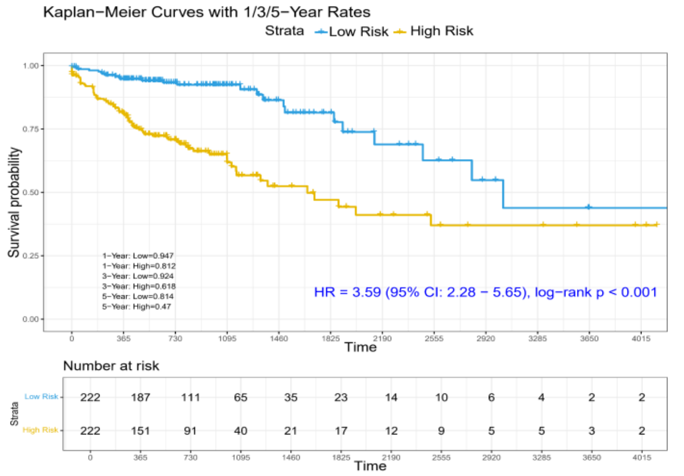

(C)

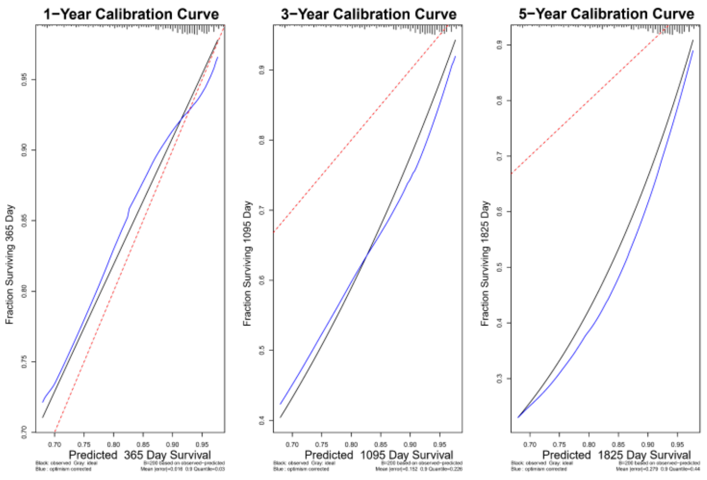

(D)

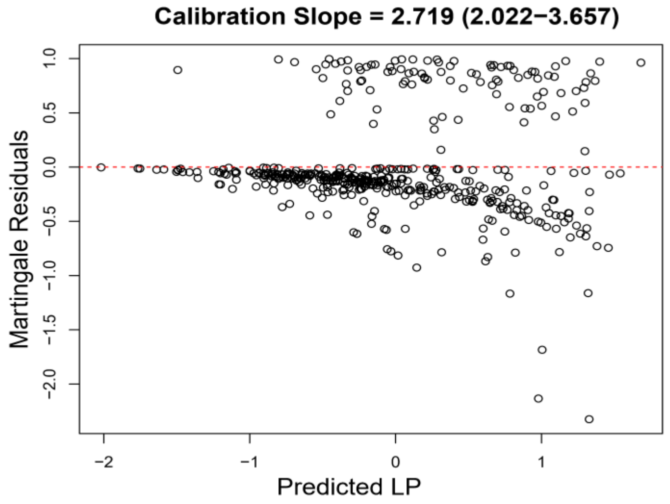

(E)

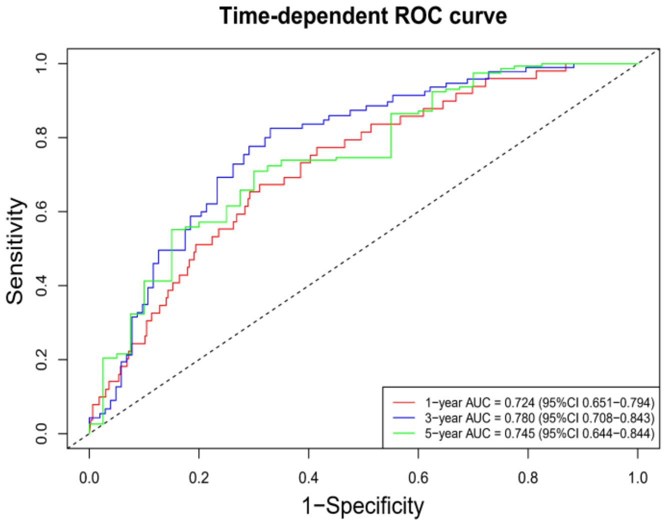

(F)

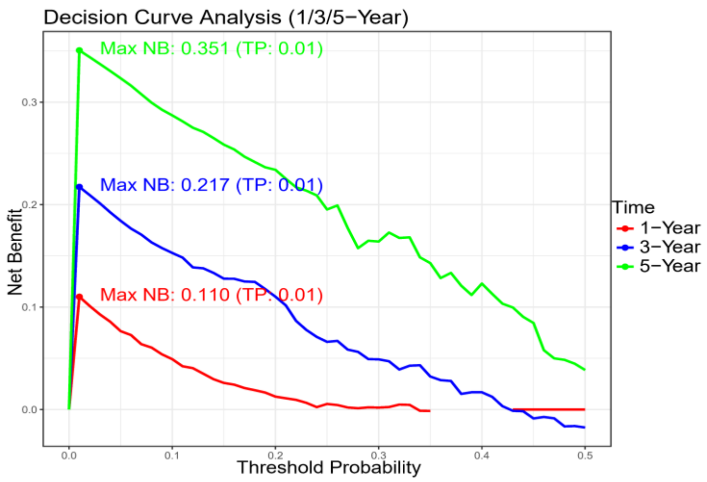

Supplementary Figure S16. Comprehensive evaluation of the prognostic models for COAD.

The models' construction followed the procedure: Univariate Cox (*P* <0.3) + Multivariate Cox. The resulting risk score formula of genomic model is: Risk Score = 0.1113 * gene_ADA. The resulting risk score formula of integrated clinico-genomic model is: Risk Score = 0.7243 * risk_score_gene + 0.0357 * age + 1.2543 * stage + 13.1236 * race asian + 12.5608 * race black or african american + 12.4635 * race unknow + 12.3975 * race white + -0.0956 * gender Male.

(A) Nomogram for Survival Prediction: A graphical representation of the prognostic model, allowing for the visual estimation of 1-, 3-, and 5-year overall survival probabilities based on individual gene expression levels (and clinical features, if clinico-genomic model). The genomic model's internal validation C-index was 0.557 (95%CI: 0.479-0.626), integrated clinico-genomic model's internal validation C-index was 0.715 (95%CI: 0.672-0.776).

(B) Kaplan-Meier Survival Curves: Survival analysis demonstrated significant differences in overall survival between the high- and low-risk groups in both the genomic model (HR = 1.63, 95% CI: 1.08–2.46, log-rank $p = 0.02$) and the integrated clinico-genomic model (HR = 3.59, 95% CI: 2.28–5.65, log-rank $p < 0.001$). The table below shows the number of patients at risk over time for each group.

(C) 1-, 3-, and 5-Year Calibration Curves: Assesses the agreement between the predicted and observed overall survival probabilities at 1, 3, and 5 years, respectively. The diagonal dashed line represents perfect calibration.

(D) Calibration Slope Plot: Further evaluates the model's calibration, showing the relationship between predicted linear predictor and Martingale residuals. The calibration slope of genomic model was 2.718 (95%CI: 0.549-13.466). The calibration slope of integrated clinico-genomic model was 2.719 (95%CI: 2.022-3.657).

(E) Time-Dependent Receiver Operating Characteristic (ROC) Curves: Illustrates the discriminatory ability of the model over time. The area under the curve (AUC) values of genomic model were 0.549 (0.452-0.642), 0.540 (0.451-0.627), and 0.469 (0.352-0.584) for 1-, 3-, and 5-year survival, respectively. The AUC values of integrated

clinico-genomic model were 0.724 (0.651-0.794), 0.780 (0.708-0.843), and 0.745 (0.644-0.844) for 1-, 3-, and 5-year survival, respectively.

(F) Decision Curve Analysis (DCA): Evaluates the clinical utility of the prognostic model by quantifying the net benefit across a range of threshold probabilities. The maximum net benefit (Max NB) and corresponding threshold probability (TP) for 1-, 3-, and 5-year survival in genomic model were: 1-year Max NB =0.043 (TP: 0.08); 3-year Max NB = 0.089 (TP: 0.15); 5-year Max NB = 0.155 (TP: 0.24). The Max NB and TP for 1-, 3-, and 5-year survival in integrated clinico-genomic model were: 1-year Max NB =0.110 (TP: 0.01); 3-year Max NB = 0.217 (TP: 0.01); 5-year Max NB = 0.351 (TP: 0.01).

***Genomic prognostic model for ESCA*:**

Univariate Cox (P < 0.2) + Multivariate Cox

Risk Score = 0.2613 * gene_HPRT1 + 0.2224 * gene_NT5C3A

(A)

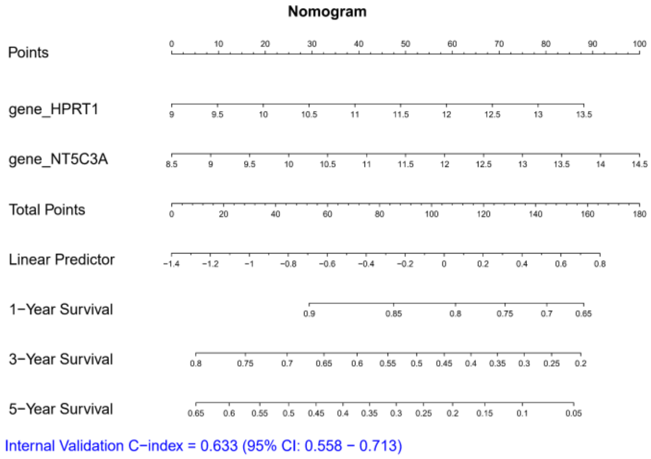

(B)

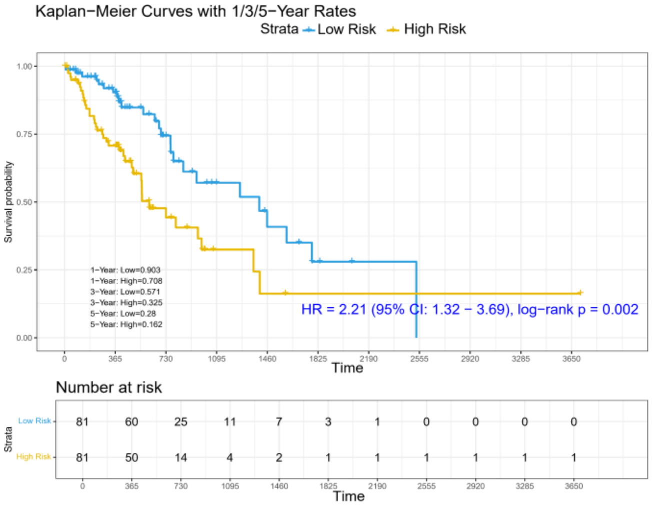

(C)

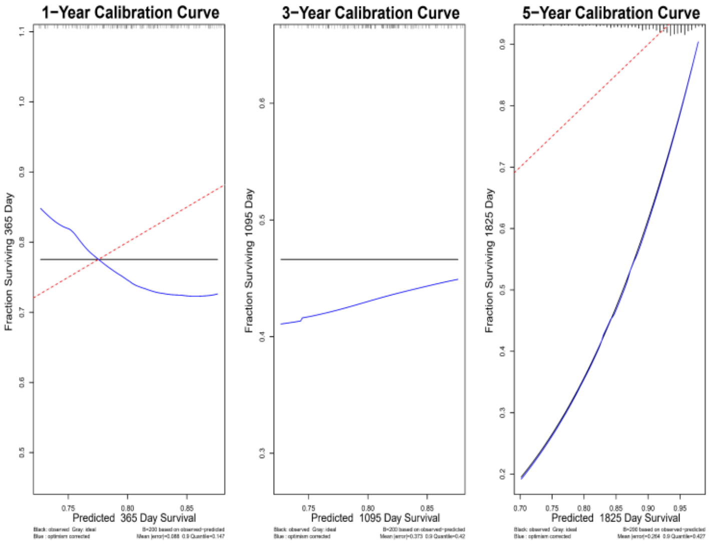

(D)

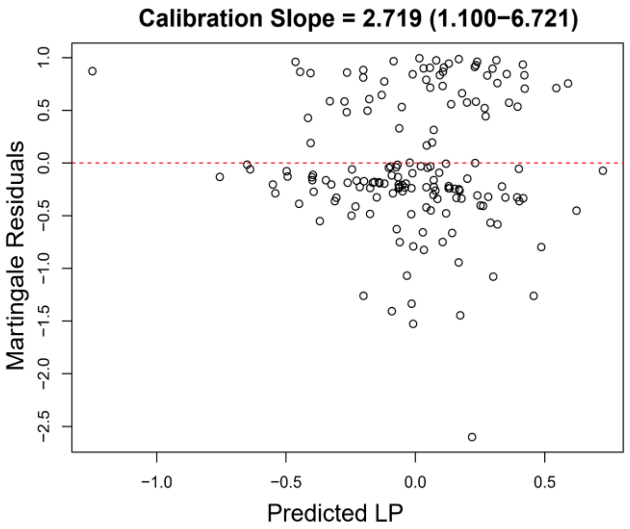

(E)

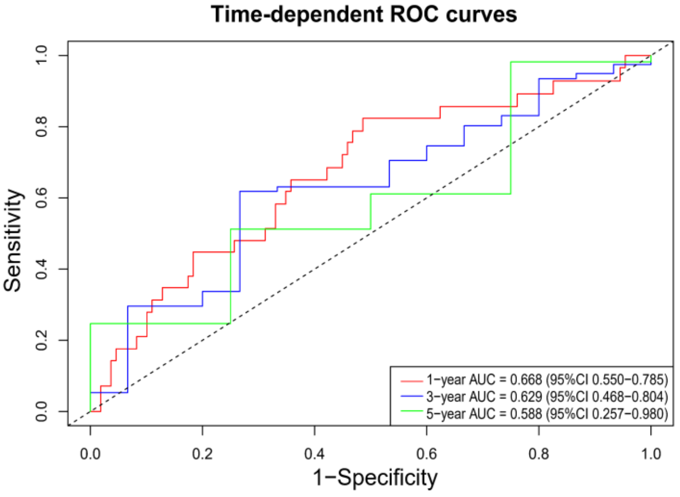

(F)

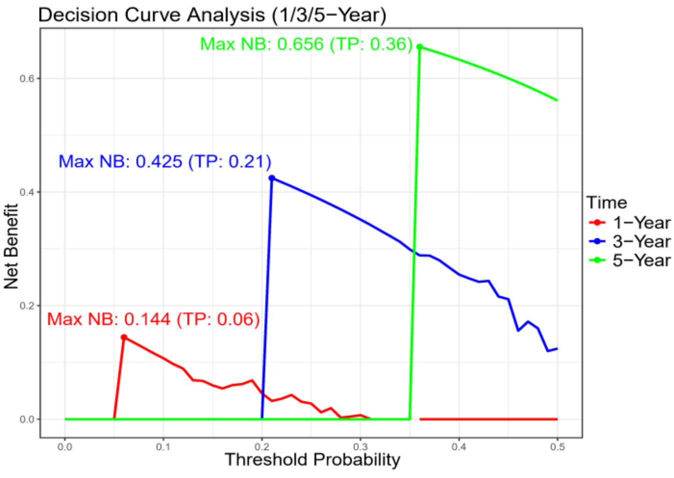

<u>Clinico-genomic prognostic model for ESCA</u>:

Univariate Cox (P < 0.2) + Multivariate Cox

Risk Score = 0.7812 * risk_score_gene + 0.007 * age + 0.9651 * stage + 0.6402 * race black or african american + 0.6733 * race unknow + -0.0117 * race white + 0.4156 * gender Male

(A)

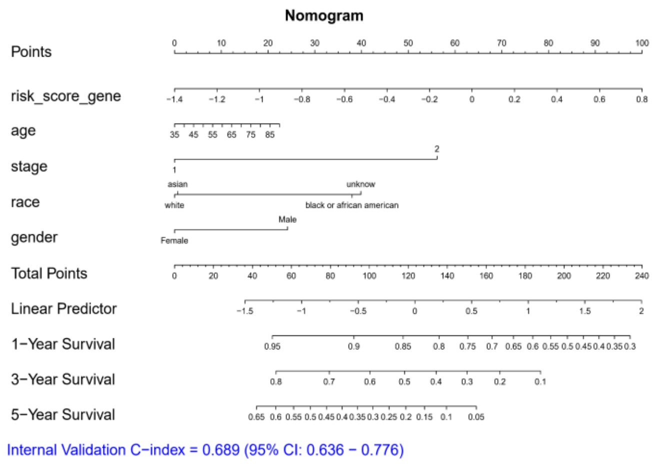

(B)

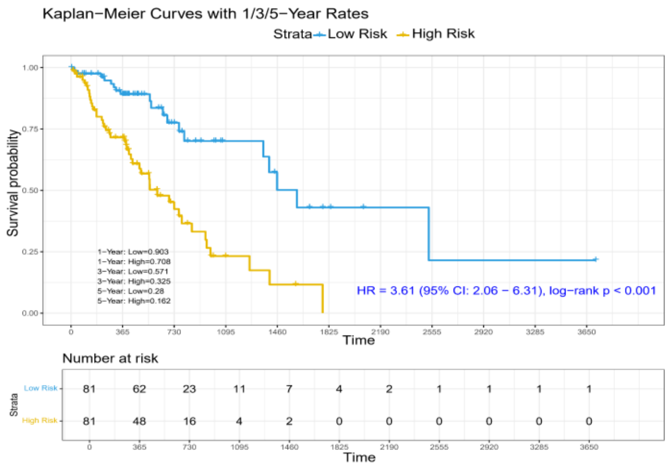

(C)

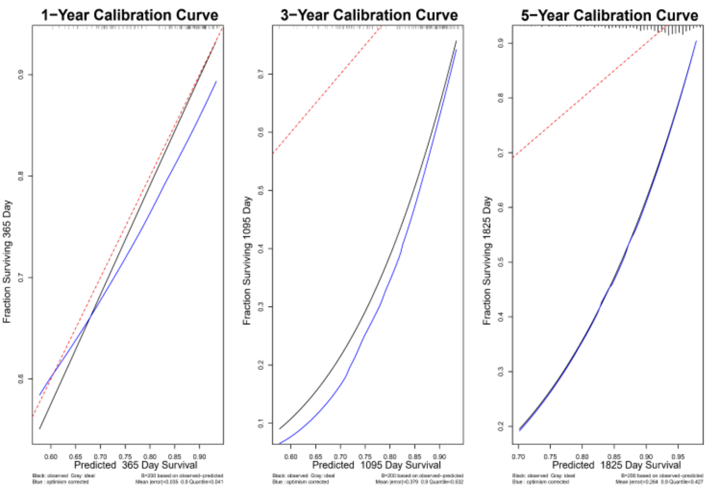

(D)

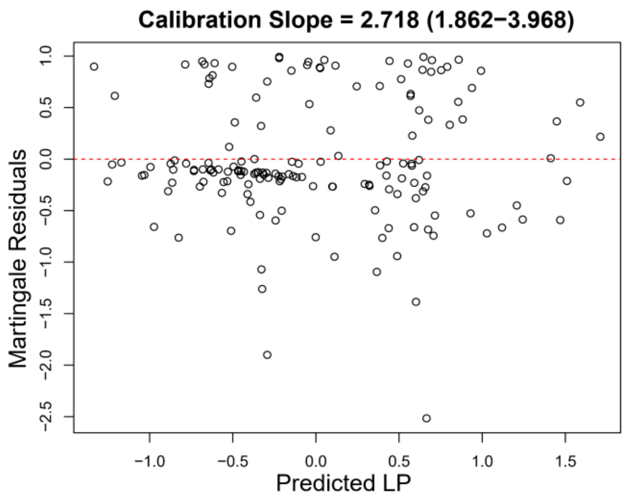

(E)

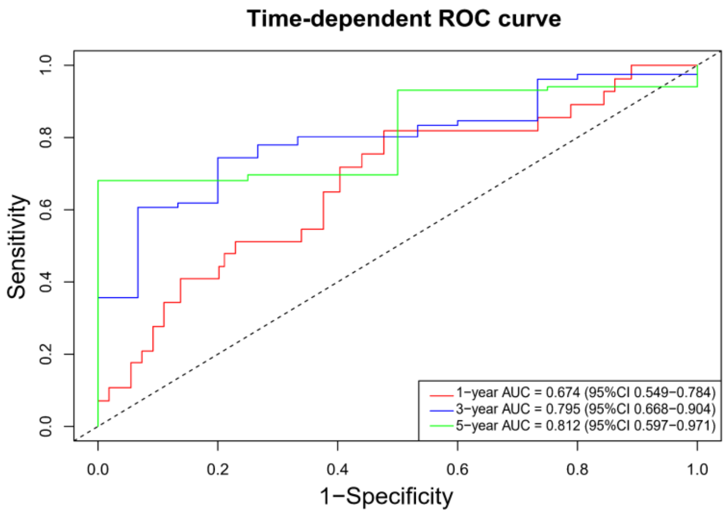

(F)

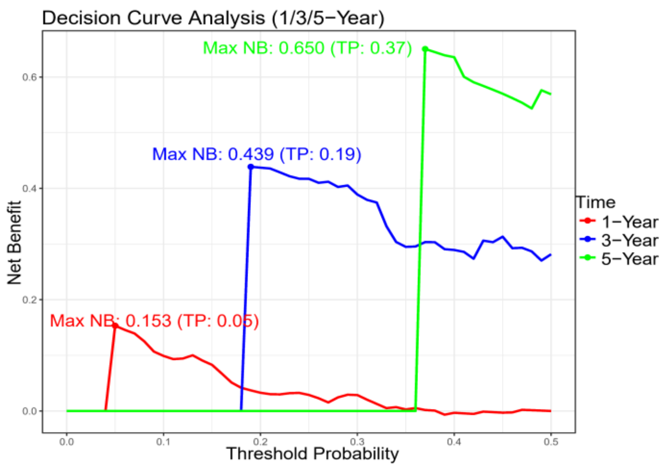

Supplementary Figure S17. Comprehensive evaluation of the prognostic models for ESCA.

The models' construction followed the procedure: Univariate Cox (*P* <0.2) + Multivariate Cox. The resulting risk score formula of genomic model is: Risk Score = 0.2613 * gene_HPRT1 + 0.2224 * gene_NT5C3A. The resulting risk score formula of integrated clinico-genomic model is: Risk Score = 0.7812 * risk_score_gene + 0.007 * age + 0.9651 * stage + 0.6402 * race black or african american + 0.6733 * race unknow + -0.0117 * race white + 0.4156 * gender Male.

(A) Nomogram for Survival Prediction: A graphical representation of the prognostic model, allowing for the visual estimation of 1-, 3-, and 5-year overall survival probabilities based on individual gene expression levels (and clinical features, if clinico-genomic model). The genomic model's internal validation C-index was 0.633 (95%CI: 0.558-0.713), integrated clinico-genomic model's internal validation C-index was 0.689 (95%CI: 0.636-0.776).

(B) Kaplan-Meier Survival Curves: Survival analysis demonstrated significant differences in overall survival between the high- and low-risk groups in both the genomic model (HR = 2.21, 95% CI: 1.32–3.69, log-rank *p* = 0.002) and the integrated clinico-genomic model (HR = 3.61, 95% CI: 2.06–6.31, log-rank *p* <0.001). The table below shows the number of patients at risk over time for each group.

(C) 1-, 3-, and 5-Year Calibration Curves: Assesses the agreement between the predicted and observed overall survival probabilities at 1, 3, and 5 years, respectively. The diagonal dashed line represents perfect calibration.

(D) Calibration Slope Plot: Further evaluates the model's calibration, showing the relationship between predicted linear predictor and Martingale residuals. The calibration slope of genomic model was 2.719 (95%CI: 1.100-6.721). The calibration slope of integrated clinico-genomic model was 2.718 (95%CI: 1.862-3.968).

(E) Time-Dependent Receiver Operating Characteristic (ROC) Curves: Illustrates the discriminatory ability of the model over time. The area under the curve (AUC) values of genomic model were 0.668 (0.550-0.785), 0.629 (0.468-0.804), and 0.588 (0.257-0.980) for 1-, 3-, and 5-year survival, respectively. The AUC values of integrated

clinico-genomic model were 0.674 (0.549-0.784), 0.795 (0.668-0.904), and 0.812 (0.597-0.971) for 1-, 3-, and 5-year survival, respectively.

(F) Decision Curve Analysis (DCA): Evaluates the clinical utility of the prognostic model by quantifying the net benefit across a range of threshold probabilities. The maximum net benefit (Max NB) and corresponding threshold probability (TP) for 1-, 3-, and 5-year survival in genomic model were: 1-year Max NB =0.144 (TP: 0.06); 3-year Max NB = 0.425 (TP: 0.21); 5-year Max NB = 0.656 (TP: 0.36). The Max NB and TP for 1-, 3-, and 5-year survival in integrated clinico-genomic model were: 1-year Max NB =0.153 (TP: 0.05); 3-year Max NB = 0.439 (TP: 0.19); 5-year Max NB = 0.650 (TP: 0.37).

***Genomic prognostic model for HNSC*:**

Univariate Cox (P < 0.2) + LASSO with 10-fold CV (lambda.min) + Multivariate Cox

Risk Score = 0.0772 * gene_ADA + -0.067 * gene_ADCY5 + 0.4992 * gene_HPRT1 + 0.1175 * gene_PKM

(A)

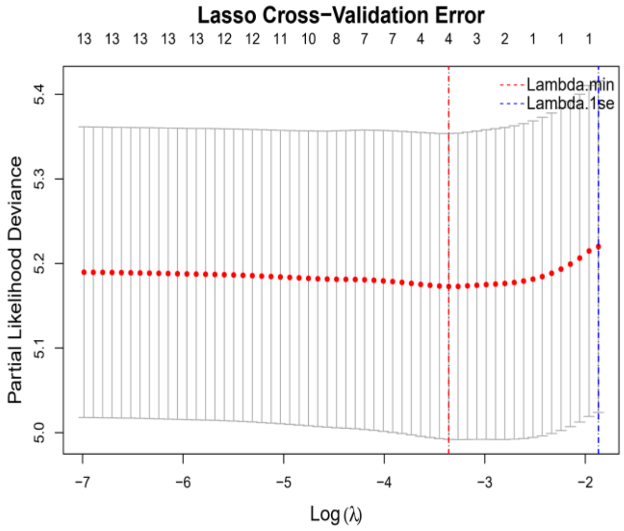

(B)

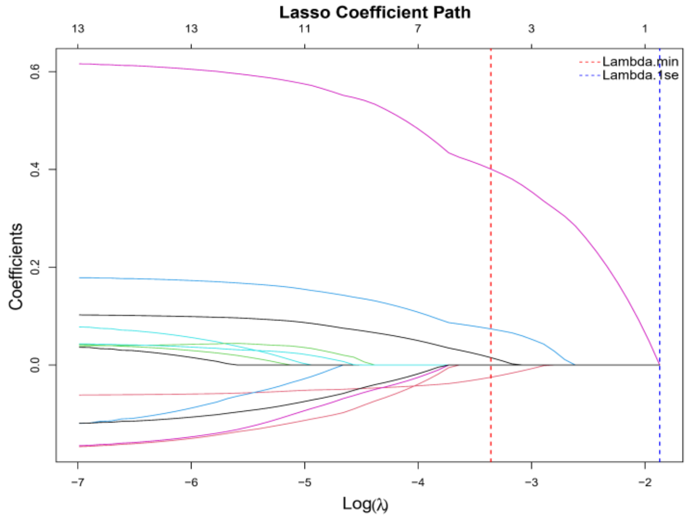

(C)

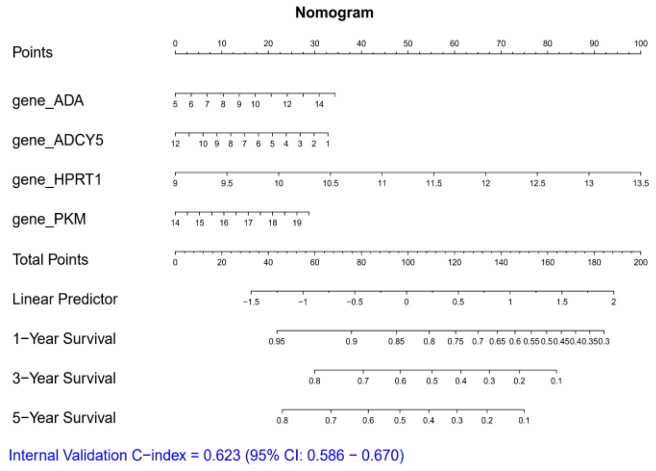

(D)

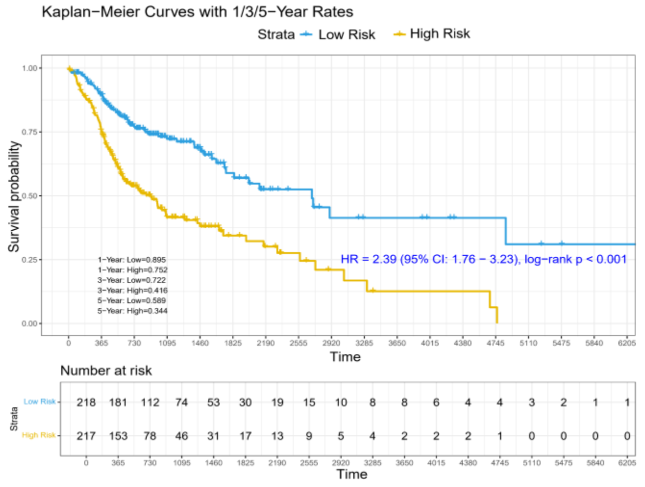

(E)

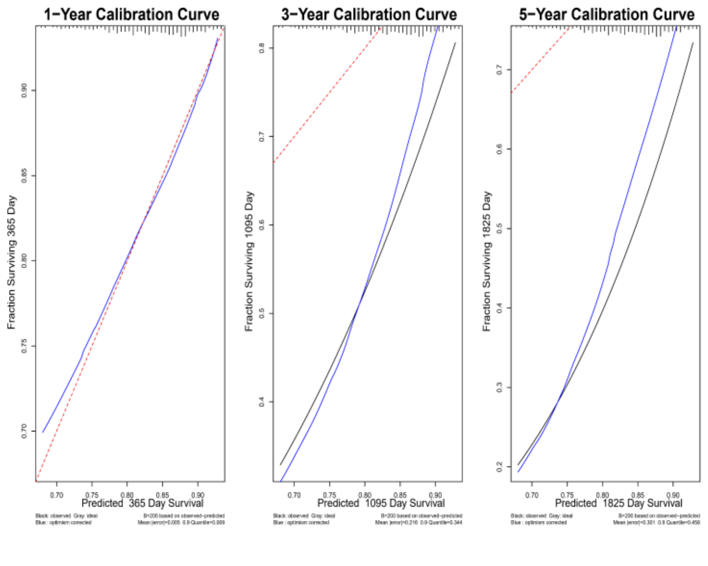

(F)

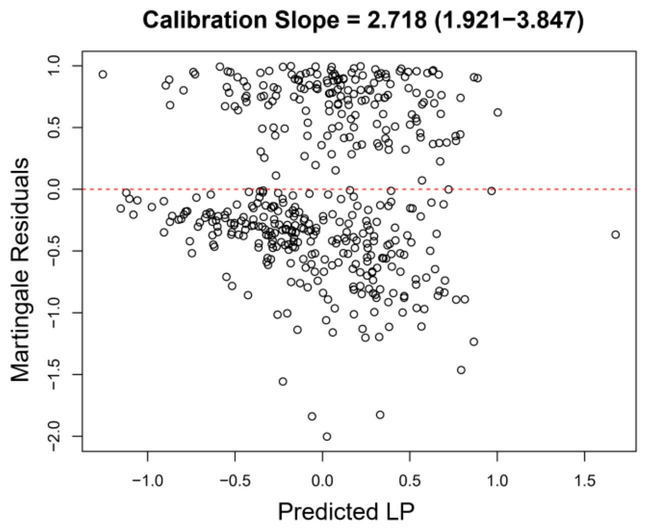

(G)

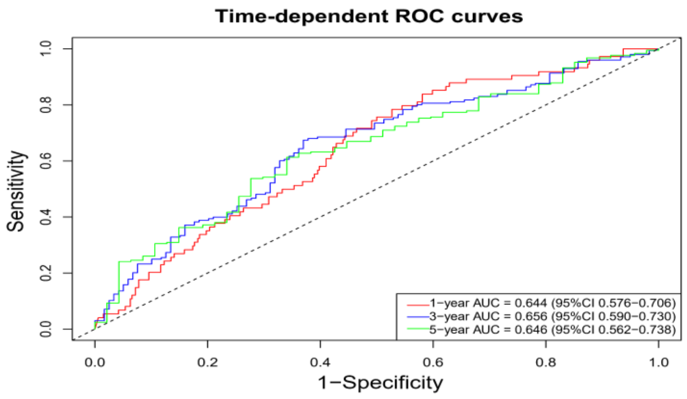

(H)

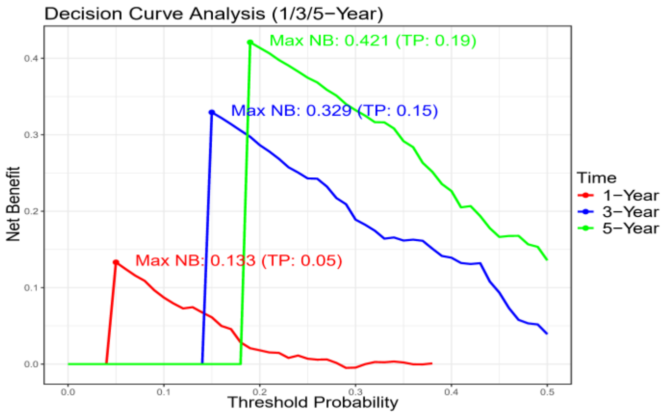

<u>Clinico-genomic prognostic model for HNSC</u>:

Univariate Cox ($P < 0.2$) + LASSO with 10-fold CV (lambda.min) + Multivariate Cox

Risk Score = 1.0333 * risk_score_gene + 0.0249 * age + 0.6851 * stage + 0.2134 * race asian + 0.0203 * race black or african american + -0.03 * race unknow + -0.2747 * race white

(A)

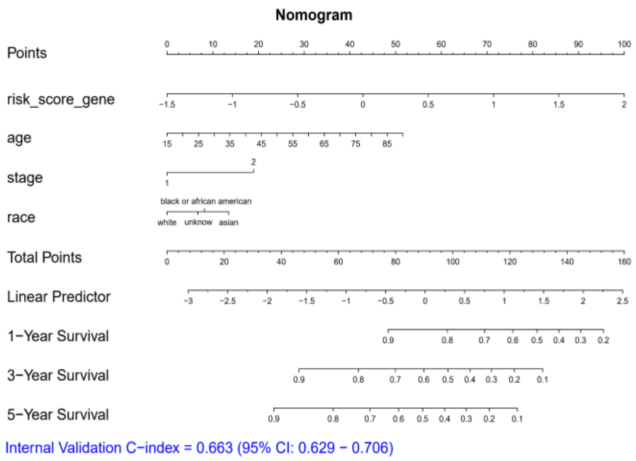

(B)

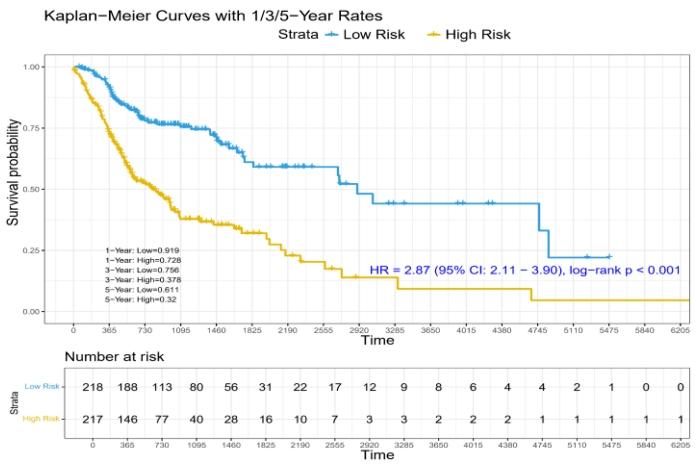

(C)

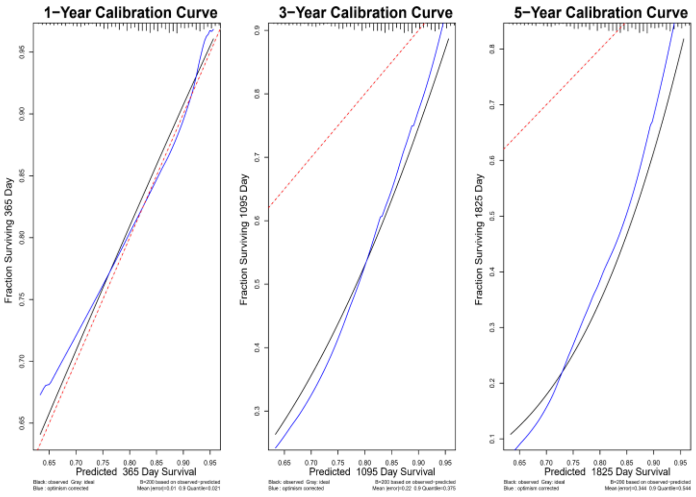

(D)

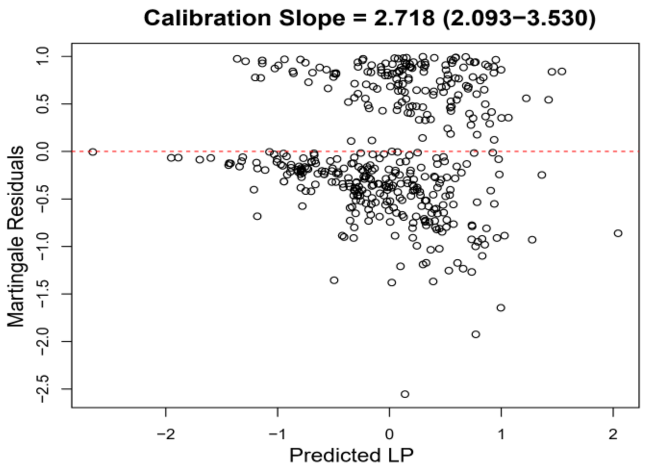

(E)

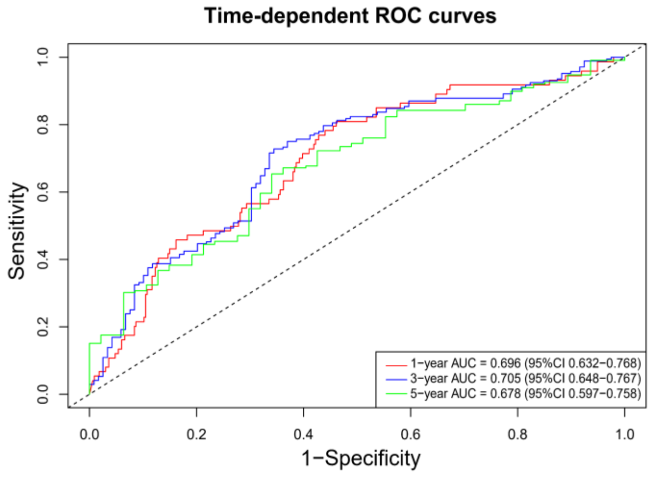

(F)

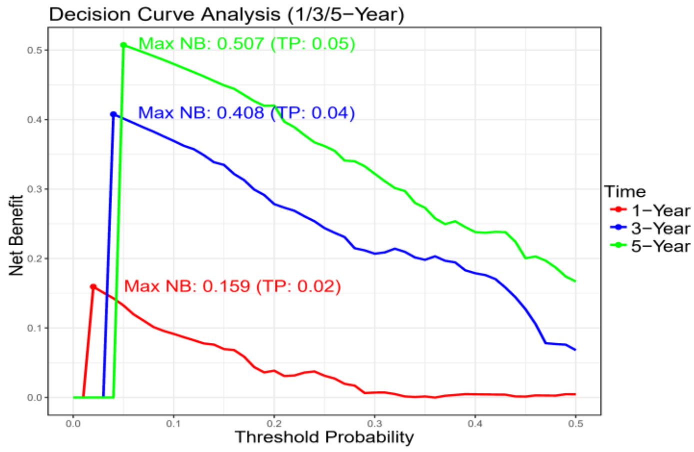

Supplementary Figure S18. Comprehensive evaluation of the prognostic models for HNSC. The models' construction followed the procedure: Univariate Cox (*P* <0.2) + LASSO with 10-fold CV (lambda.min) + Multivariate Cox. The resulting risk score formula of genomic model is: Risk Score = 0.0772 * gene_ADA + -0.067 * gene_ADCY5 + 0.4992 * gene_HPRT1 + 0.1175 * gene_PKM. The resulting risk score formula of integrated clinico-genomic model is: Risk Score = 1.0333 * risk_score_gene + 0.0249 * age + 0.6851 * stage + 0.2134 * race asian + 0.0203 * race black or african american + -0.03 * race unknow + -0.2747 * race white.

(A) Lasso Cross-Validation Error Plot: Depicts the cross-validation error distribution across different lambda (λ) values, indicating the optimal lambda values (lambda.min and lambda.1se) for penalty parameter selection in LASSO regression. The best-performing model for this tumor type used lambda.min, with a λ value of 0.035.

(B) Lasso Coefficient Path: Illustrates how the coefficients of the selected genes change as the lambda (λ) regularization parameter varies, demonstrating the variable selection process.

(C) in genomic model figures and (A) in integrated clinico-genomic model figures: Nomogram for Survival Prediction: A graphical representation of the prognostic model, allowing for the visual estimation of 1-, 3-, and 5-year overall survival probabilities based on individual gene expression levels (and clinical features, if clinico-genomic model). The genomic model's internal validation C-index was 0.623 (95%CI: 0.586-0.670), integrated clinico-genomic model's internal validation C-index was 0.663 (95%CI: 0.629-0.706).

(D) in genomic model figures and (B) in integrated clinico-genomic model figures: Kaplan-Meier Survival Curves: Survival analysis demonstrated significant differences in overall survival between the high- and low-risk groups in both the genomic model (HR = 2.39, 95% CI: 1.76–3.23, log-rank *p* <0.001) and the integrated clinico-genomic model (HR = 2.87, 95% CI: 2.11–3.90, log-rank *p* <0.001). The table below shows the number of patients at risk over time for each group.

(E) in genomic model figures and (C) in integrated clinico-genomic model figures: 1-, 3-, and 5-Year Calibration Curves: Assesses the agreement between the predicted

and observed overall survival probabilities at 1, 3, and 5 years, respectively. The diagonal dashed line represents perfect calibration.

(F) in genomic model figures and (D) in integrated clinico-genomic model figures: Calibration Slope Plot: Further evaluates the model's calibration, showing the relationship between predicted linear predictor and Martingale residuals. The calibration slope of genomic model was 2.718 (95%CI: 1.921-3.847). The calibration slope of integrated clinico-genomic model was 2.718 (95%CI: 2.093-3.53).

(G) in genomic model figures and (E) in integrated clinico-genomic model figures: Time-Dependent Receiver Operating Characteristic (ROC) Curves: Illustrates the discriminatory ability of the model over time. The area under the curve (AUC) values of genomic model were 0.644 (0.576-0.706), 0.656 (0.590-0.730), and 0.646 (0.562-0.738) for 1-, 3-, and 5-year survival, respectively. The AUC values of integrated clinico-genomic model were 0.696 (0.632-0.768), 0.705 (0.648-0.767), and 0.678 (0.597-0.758) for 1-, 3-, and 5-year survival, respectively.

(H) in genomic model figures and (F) in integrated clinico-genomic model figures: Decision Curve Analysis (DCA): Evaluates the clinical utility of the prognostic model by quantifying the net benefit across a range of threshold probabilities. The maximum net benefit (Max NB) and corresponding threshold probability (TP) for 1-, 3-, and 5-year survival in genomic model were: 1-year Max NB =0.133 (TP: 0.05); 3-year Max NB = 0.329 (TP: 0.15); 5-year Max NB = 0.421 (TP: 0.19). The Max NB and TP for 1-, 3-, and 5-year survival in integrated clinico-genomic model were: 1-year Max NB = 0.159 (TP: 0.02); 3-year Max NB = 0.408 (TP: 0.04); 5-year Max NB = 0.507 (TP: 0.05).

***Genomic prognostic model for KIRC*:**

Univariate Cox (P < 0.2) + LASSO with 10-fold CV (lambda.min) + Multivariate Cox Risk Score = -0.3494 * gene_ACADM + 0.3832 * gene_IMPDH1 + 0.1091 * gene_NME1 + -0.0502 * gene_PDE7B + 0.1416 * gene_RRM2 + 0.0301 * gene_TYMP

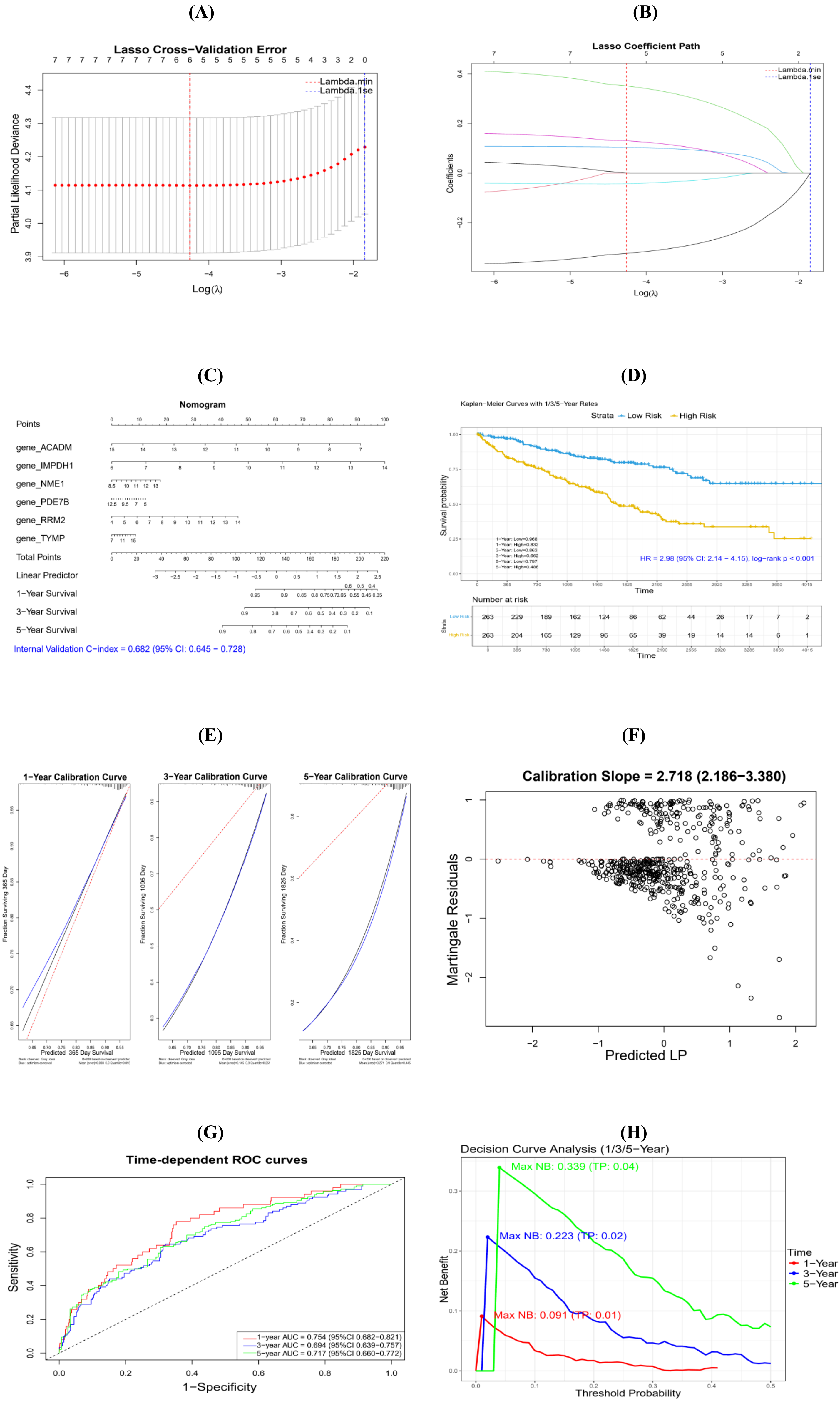

<u>Clinico-genomic prognostic model for KIRC</u>:

Univariate Cox (P < 0.2) + LASSO with 10-fold CV (lambda.min) + Multivariate Cox

Risk Score = 0.8337 * risk_score_gene + 0.0278 * age + 1.0553 * stage + 1.5305 * race black or african american + 1.4599 * race unknow + 1.6592 * race white

(A)

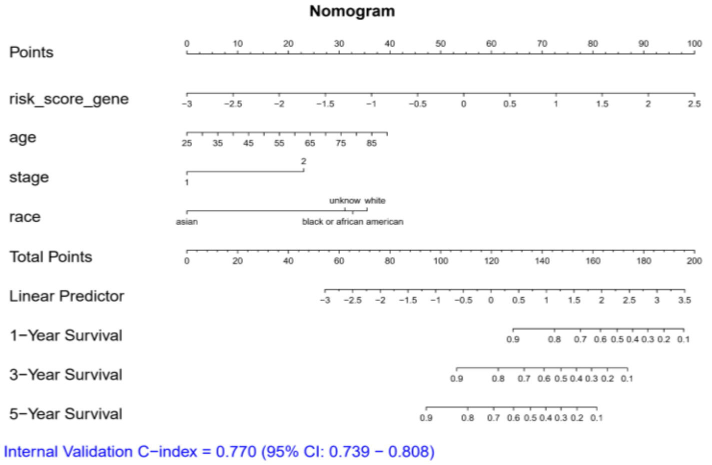

(B)

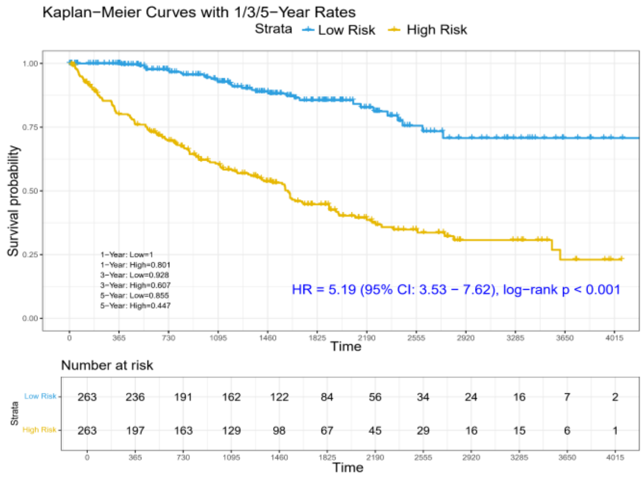

(C)

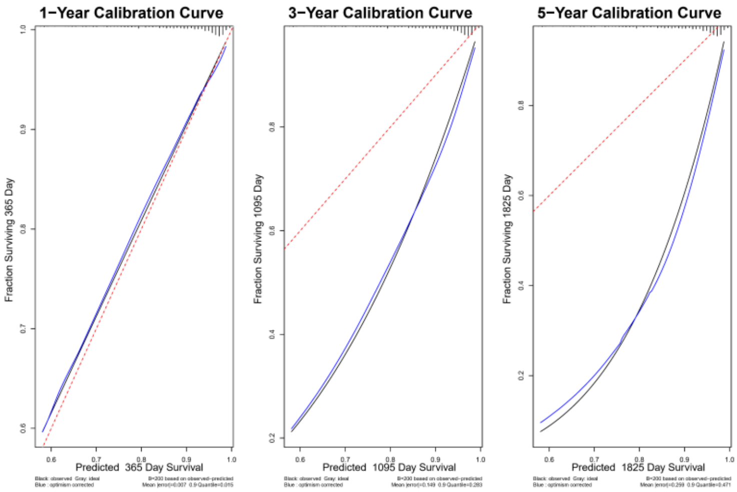

(D)

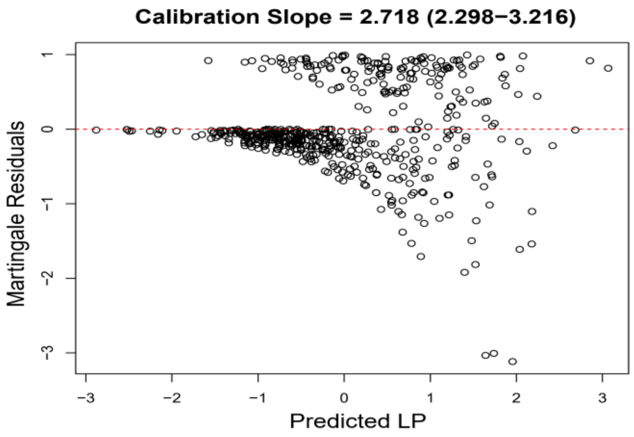

(E)

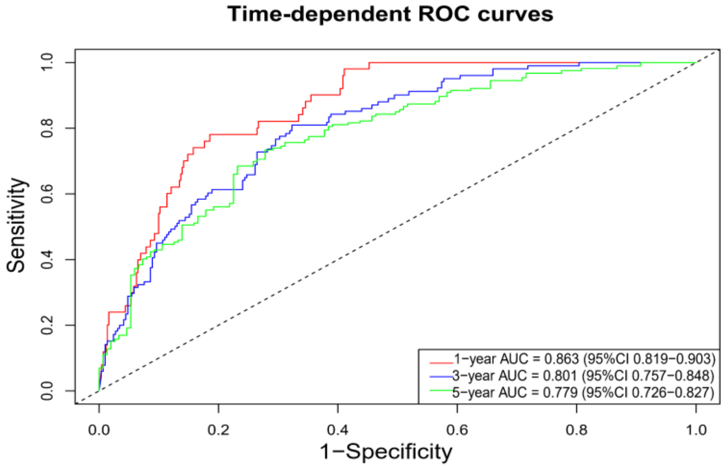

(F)

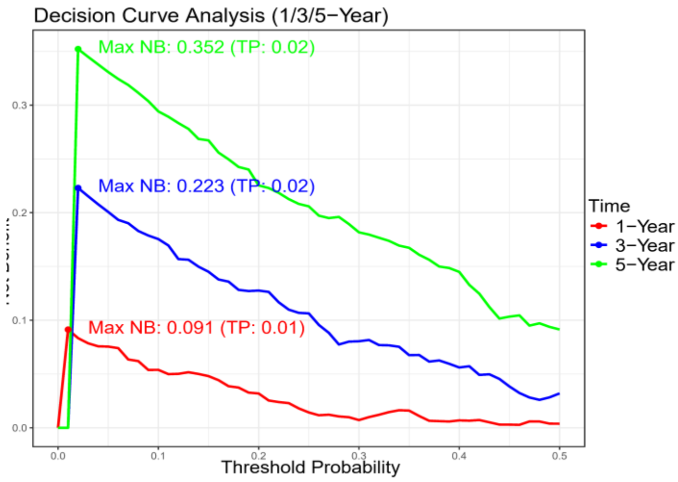

Supplementary Figure S19. Comprehensive evaluation of the prognostic models for KIRC. The models' construction followed the procedure: Univariate Cox (*P* <0.2) + LASSO with 10-fold CV (lambda.min) + Multivariate Cox. The resulting risk score formula of genomic model is: Risk Score = -0.3494 * gene_ACADM + 0.3832 * gene_IMPDH1 + 0.1091 * gene_NME1 + -0.0502 * gene_PDE7B + 0.1416 * gene_RRM2 + 0.0301 * gene_TYMP. The resulting risk score formula of integrated clinico-genomic model is: Risk Score = 0.8337 * risk_score_gene + 0.0278 * age + 1.0553 * stage + 1.5305 * race black or african american + 1.4599 * race unknow + 1.6592 * race white.

(A) Lasso Cross-Validation Error Plot: Depicts the cross-validation error distribution across different lambda (λ) values, indicating the optimal lambda values (lambda.min and lambda.1se) for penalty parameter selection in LASSO regression. The best-performing model for this tumor type used lambda.min, with a λ value of 0.014.

(B) Lasso Coefficient Path: Illustrates how the coefficients of the selected genes change as the lambda (λ) regularization parameter varies, demonstrating the variable selection process.

(C) in genomic model figures and (A) in integrated clinico-genomic model figures: Nomogram for Survival Prediction: A graphical representation of the prognostic model, allowing for the visual estimation of 1-, 3-, and 5-year overall survival probabilities based on individual gene expression levels (and clinical features, if clinico-genomic model). The genomic model's internal validation C-index was 0.682 (95%CI: 0.645-0.728), integrated clinico-genomic model's internal validation C-index was 0.770 (95%CI: 0.739-0.808).

(D) in genomic model figures and (B) in integrated clinico-genomic model figures: Kaplan-Meier Survival Curves: Survival analysis demonstrated significant differences in overall survival between the high- and low-risk groups in both the genomic model (HR = 2.98, 95% CI: 2.14–4.15, log-rank p <0.001) and the integrated clinico-genomic model (HR = 5.19, 95% CI: 3.53–7.62, log-rank p <0.001). The table below shows the number of patients at risk over time for each group.

(E) in genomic model figures and (C) in integrated clinico-genomic model figures:

1-, 3-, and 5-Year Calibration Curves: Assesses the agreement between the predicted and observed overall survival probabilities at 1, 3, and 5 years, respectively. The diagonal dashed line represents perfect calibration.

(F) in genomic model figures and (D) in integrated clinico-genomic model figures: Calibration Slope Plot: Further evaluates the model's calibration, showing the relationship between predicted linear predictor and Martingale residuals. The calibration slope of genomic model was 2.718 (95%CI: 2.186-3.380). The calibration slope of integrated clinico-genomic model was 2.718 (95%CI: 2.298-3.216).

(G) in genomic model figures and (E) in integrated clinico-genomic model figures: Time-Dependent Receiver Operating Characteristic (ROC) Curves: Illustrates the discriminatory ability of the model over time. The area under the curve (AUC) values of genomic model were 0.754 (0.682-0.821), 0.694 (0.639-0.757), and 0.717 (0.660-0.772) for 1-, 3-, and 5-year survival, respectively. The AUC values of integrated clinico-genomic model were 0.863 (0.819-0.903), 0.801 (0.757-0.848), and 0.779 (0.726-0.827) for 1-, 3-, and 5-year survival, respectively.

(H) in genomic model figures and (F) in integrated clinico-genomic model figures: Decision Curve Analysis (DCA): Evaluates the clinical utility of the prognostic model by quantifying the net benefit across a range of threshold probabilities. The maximum net benefit (Max NB) and corresponding threshold probability (TP) for 1-, 3-, and 5-year survival in genomic model were: 1-year Max NB =0.091 (TP: 0.01); 3-year Max NB = 0.223 (TP: 0.02); 5-year Max NB = 0.339 (TP: 0.04). The Max NB and TP for 1-, 3-, and 5-year survival in integrated clinico-genomic model were: 1-year Max NB = 0.091 (TP: 0.01); 3-year Max NB = 0.223 (TP: 0.02); 5-year Max NB = 0.352 (TP: 0.02).

***Genomic prognostic model for LIHC*:**

Univariate Cox (P < 0.2) + LASSO with 10-fold CV (lambda.min) + Multivariate Cox

Risk Score = 0.0327 * gene_ADA + 0.1165 * gene_ATIC + 0.1516 * gene_IMPDH1 + -0.1323 * gene_PDE2A + 0.2624 * gene_PPAT + 0.0328 * gene_RRM2 + 0.1176 * gene_TXN + 0.1242 * gene_UCK2

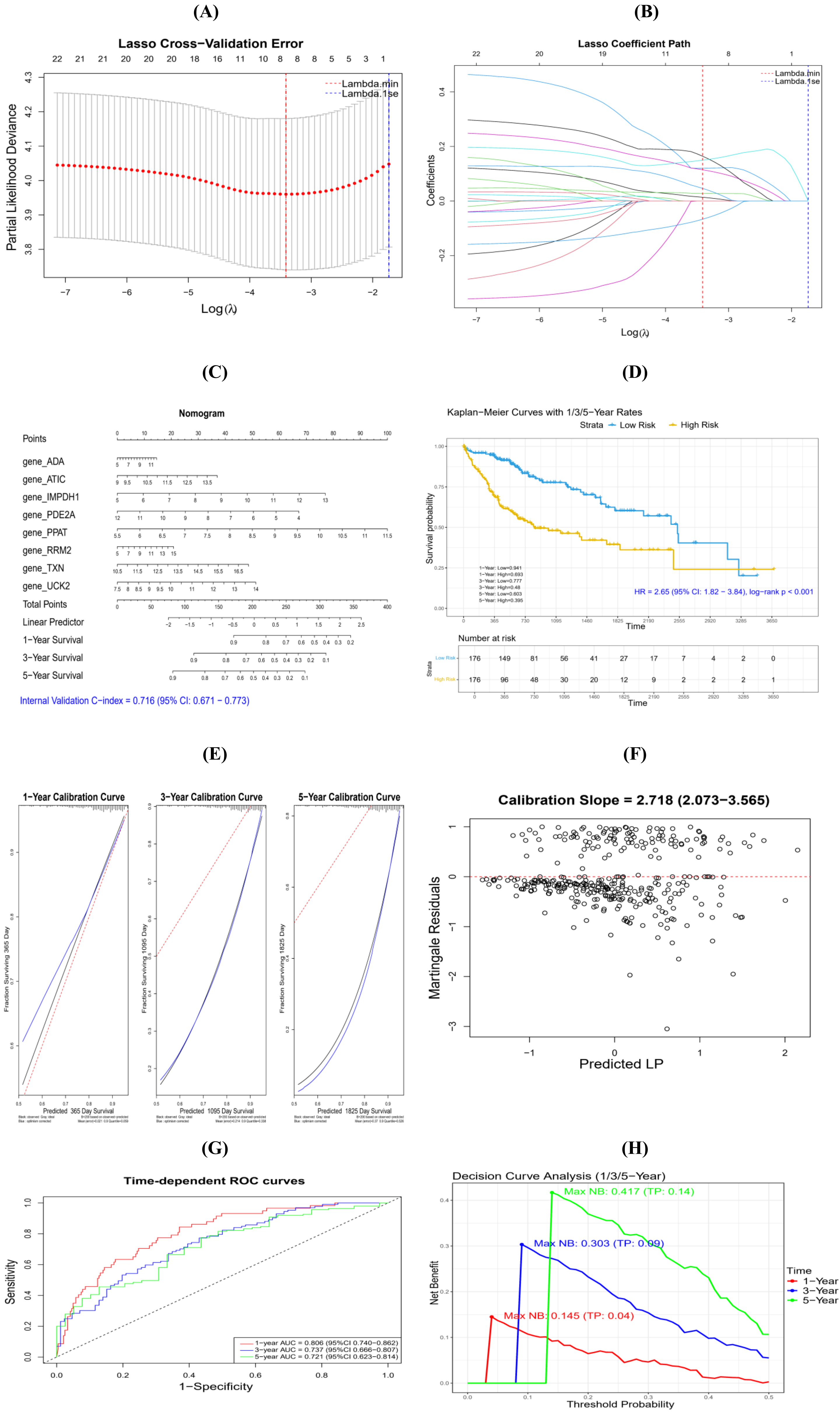

<u>Clinico-genomic prognostic model for LIHC</u>:

Univariate Cox (P < 0.2) + LASSO with 10-fold CV (lambda.min) + Multivariate Cox Risk Score = 0.9072 * risk_score_gene + 0.0134 * age + 0.7708 * stage + 13.2827 * race asian + 13.8283 * raceblack or african american + 14.1019 * race unknow + 13.2389 * race white

(A)

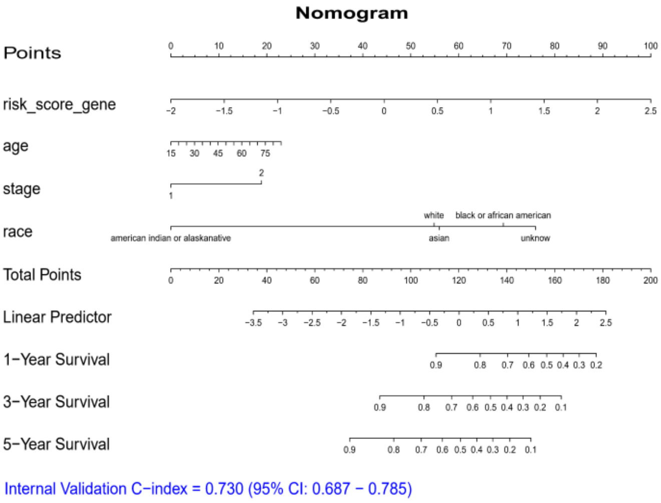

(B)

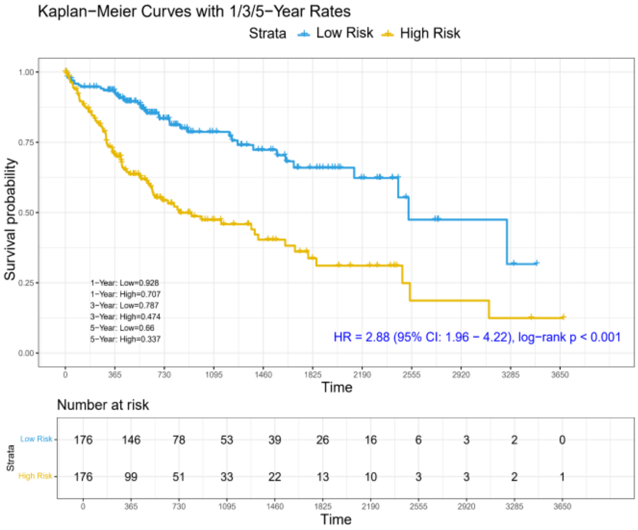

(C)

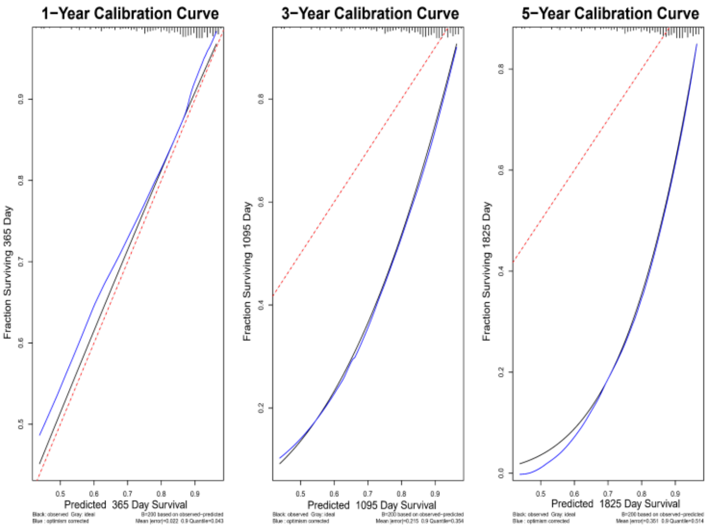

(D)

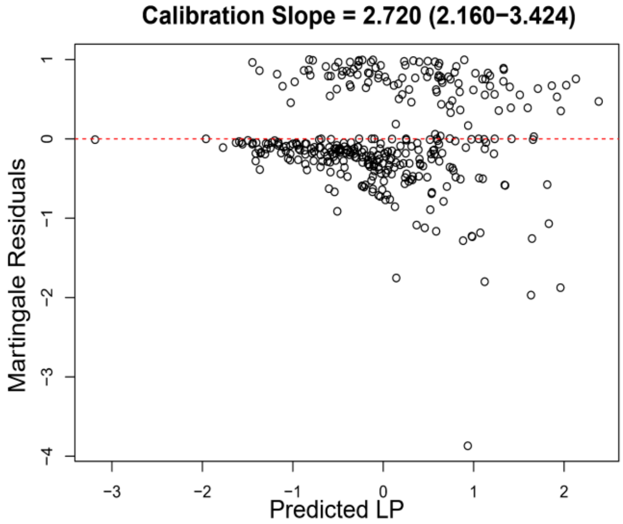

(E)

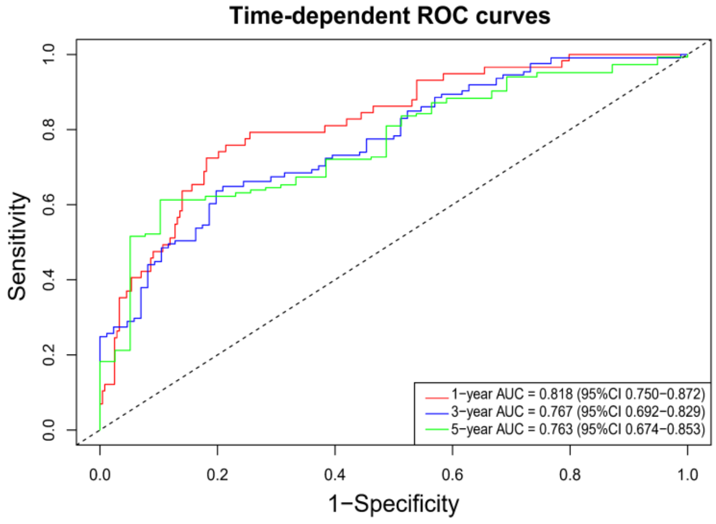

(F)

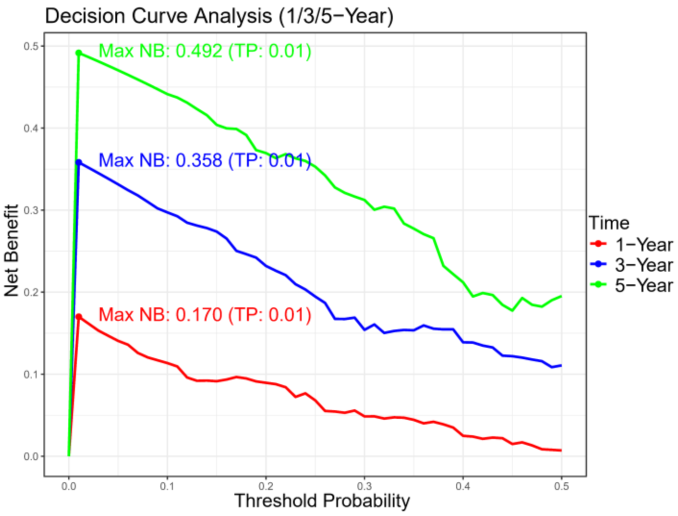

Supplementary Figure S20. Comprehensive evaluation of the prognostic models for LIHC. The models' construction followed the procedure: Univariate Cox (*P* <0.2) + LASSO with 10-fold CV (lambda.min) + Multivariate Cox. The resulting risk score formula of genomic model is: Risk Score = 0.0327 * gene_ADA + 0.1165 * gene_ATIC + 0.1516 * gene_IMPDH1 + -0.1323 * gene_PDE2A + 0.2624 * gene_PPAT + 0.0328 * gene_RRM2 + 0.1176 * gene_TXN + 0.1242 * gene_UCK2. The resulting risk score formula of integrated clinico-genomic model is: Risk Score = 0.9072 * risk_score_gene + 0.0134 * age + 0.7708 * stage + 13.2827 * race asian + 13.8283 * race black or african american + 14.1019 * race unknow + 13.2389 * race white.

(A) Lasso Cross-Validation Error Plot: Depicts the cross-validation error distribution across different lambda (λ) values, indicating the optimal lambda values (lambda.min and lambda.1se) for penalty parameter selection in LASSO regression. The best-performing model for this tumor type used lambda.min, with a λ value of 0.033.

(B) Lasso Coefficient Path: Illustrates how the coefficients of the selected genes change as the lambda (λ) regularization parameter varies, demonstrating the variable selection process.

(C) in genomic model figures and (A) in integrated clinico-genomic model figures: Nomogram for Survival Prediction: A graphical representation of the prognostic model, allowing for the visual estimation of 1-, 3-, and 5-year overall survival probabilities based on individual gene expression levels (and clinical features, if clinico-genomic model). The genomic model's internal validation C-index was 0.716 (95%CI: 0.671-0.773), integrated clinico-genomic model's internal validation C-index was 0.730 (95%CI: 0.687-0.785).

(D) in genomic model figures and (B) in integrated clinico-genomic model figures: Kaplan-Meier Survival Curves: Survival analysis demonstrated significant differences in overall survival between the high- and low-risk groups in both the genomic model (HR = 2.65, 95% CI: 1.82–3.84, log-rank *p* <0.001) and the integrated clinico-genomic model (HR = 2.88, 95% CI: 1.96–4.22, log-rank *p* <0.001). The table below shows the number of patients at risk over time for each group.

(E) in genomic model figures and (C) in integrated clinico-genomic model figures:

1-, 3-, and 5-Year Calibration Curves: Assesses the agreement between the predicted and observed overall survival probabilities at 1, 3, and 5 years, respectively. The diagonal dashed line represents perfect calibration.

(F) in genomic model figures and (D) in integrated clinico-genomic model figures: Calibration Slope Plot: Further evaluates the model's calibration, showing the relationship between predicted linear predictor and Martingale residuals. The calibration slope of genomic model was 2.718 (95%CI: 2.073-3.565). The calibration slope of integrated clinico-genomic model was 2.720 (95%CI: 2.160-3.424).

(G) in genomic model figures and (E) in integrated clinico-genomic model figures: Time-Dependent Receiver Operating Characteristic (ROC) Curves: Illustrates the discriminatory ability of the model over time. The area under the curve (AUC) values of genomic model were 0.806 (0.740-0.862), 0.737 (0.666-0.807), and 0.721 (0.623-0.814) for 1-, 3-, and 5-year survival, respectively. The AUC values of integrated clinico-genomic model were 0.818 (0.750-0.872), 0.767 (0.692-0.829), and 0.763 (0.674-0.853) for 1-, 3-, and 5-year survival, respectively.

(H) in genomic model figures and (F) in integrated clinico-genomic model figures: Decision Curve Analysis (DCA): Evaluates the clinical utility of the prognostic model by quantifying the net benefit across a range of threshold probabilities. The maximum net benefit (Max NB) and corresponding threshold probability (TP) for 1-, 3-, and 5-year survival in genomic model were: 1-year Max NB =0.145 (TP: 0.04); 3-year Max NB = 0.303 (TP: 0.09); 5-year Max NB = 0.417 (TP: 0.14). The Max NB and TP for 1-, 3-, and 5-year survival in integrated clinico-genomic model were: 1-year Max NB = 0.170 (TP: 0.01); 3-year Max NB = 0.358 (TP: 0.1); 5-year Max NB = 0.492 (TP: 0.01).

Genomic prognostic model for LUAD:

Univariate Cox ($P < 0.2$) + LASSO with 10-fold CV (lambda.min) + Multivariate Cox

Risk Score = -0.1749 * gene_PDE5A + 0.1731 * gene_RRM2 + 0.0595 * gene_TXN + 0.0544 * gene_UCK2

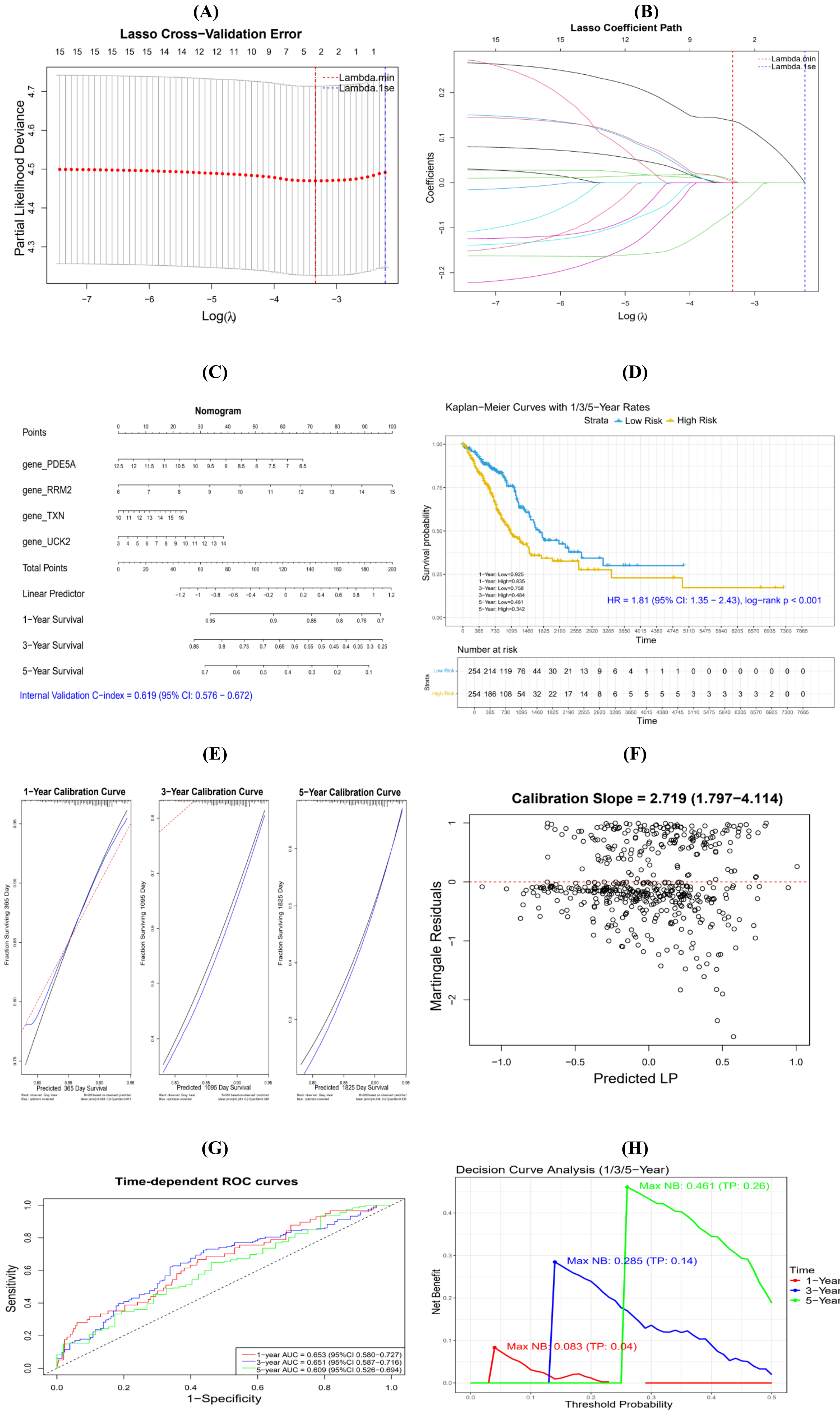

***Clinico-genomic prognostic model for LUAD*:**

Univariate Cox ($P < 0.2$) + LASSO with 10-fold CV (lambda.min) + Multivariate Cox

Risk Score = 1.0809 * risk_score_gene + 0.0146 * age + 0.8964 * stage + 12.3025 * race asian + 12.9908 * race black or african american + 13.034 * race unknow + 13.2488 * race white

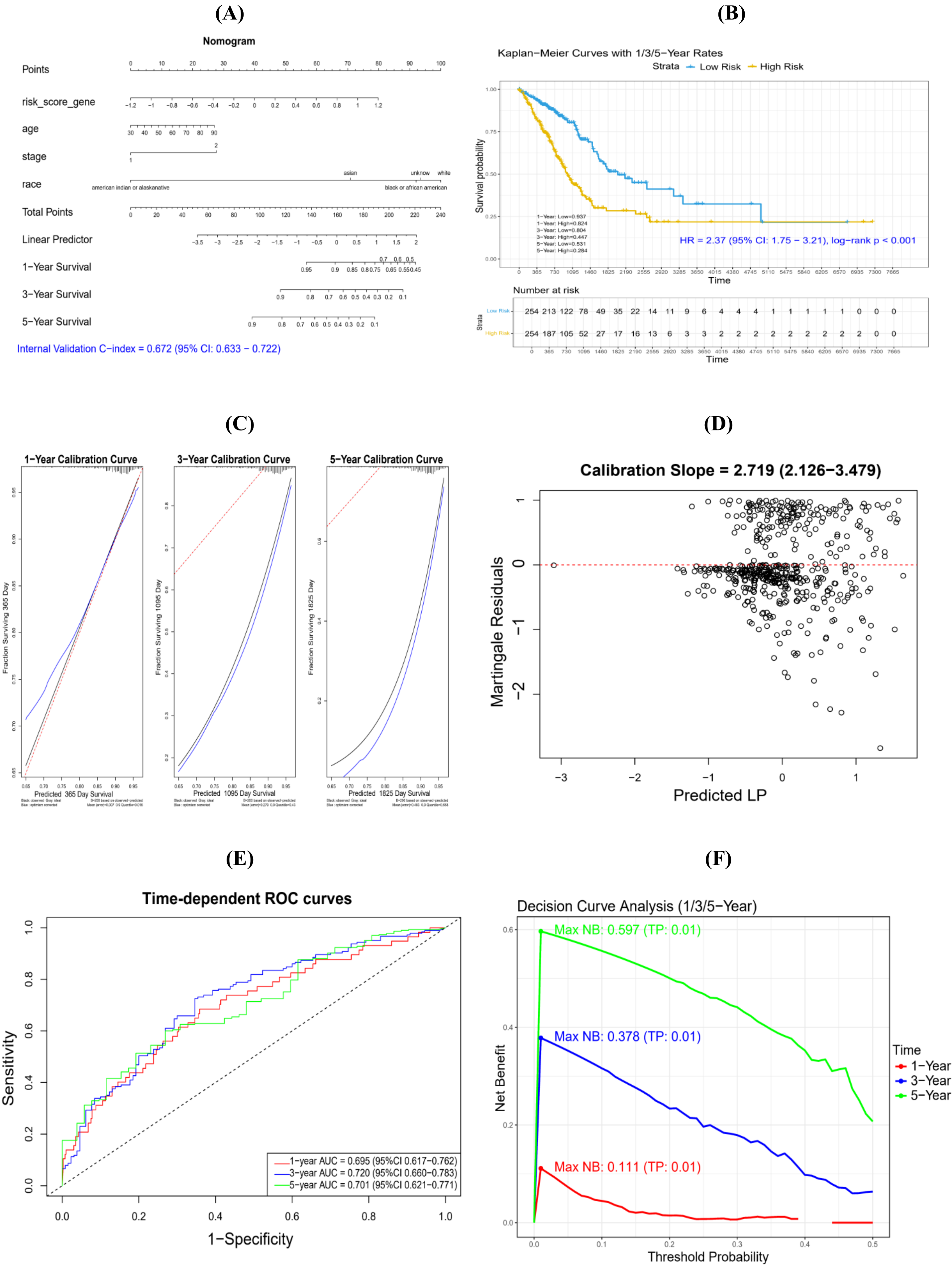

Supplementary Figure S21. Comprehensive evaluation of the prognostic models for LUAD. The models' construction followed the procedure: Univariate Cox (*P* <0.2) + LASSO with 10-fold CV (lambda.min) + Multivariate Cox. The resulting risk score formula of genomic model is: Risk Score = -0.1749 * gene_PDE5A + 0.1731 * gene_RRM2 + 0.0595 * gene_TXN + 0.0544 * gene_UCK2. The resulting risk score formula of integrated clinico-genomic model is: Risk Score = 1.0809 * risk_score_gene + 0.0146 * age + 0.8964 * stage + 12.3025 * race asian + 12.9908 * race black or african american + 13.034 * race unknow + 13.2488 * race white.

(A) Lasso Cross-Validation Error Plot: Depicts the cross-validation error distribution across different lambda (λ) values, indicating the optimal lambda values (lambda.min and lambda.1se) for penalty parameter selection in LASSO regression. The best-performing model for this tumor type used lambda.min, with a λ value of 0.035.

(B) Lasso Coefficient Path: Illustrates how the coefficients of the selected genes change as the lambda (λ) regularization parameter varies, demonstrating the variable selection process.

(C) in genomic model figures and (A) in integrated clinico-genomic model figures: Nomogram for Survival Prediction: A graphical representation of the prognostic model, allowing for the visual estimation of 1-, 3-, and 5-year overall survival probabilities based on individual gene expression levels (and clinical features, if clinico-genomic model). The genomic model's internal validation C-index was 0.619 (95%CI: 0.576-0.672), integrated clinico-genomic model's internal validation C-index was 0.672 (95%CI: 0.633-0.722).

(D) in genomic model figures and (B) in integrated clinico-genomic model figures: Kaplan-Meier Survival Curves: Survival analysis demonstrated significant differences in overall survival between the high- and low-risk groups in both the genomic model (HR = 1.81, 95% CI: 1.35–2.43, log-rank *p* <0.001) and the integrated clinico-genomic model (HR = 2.37, 95% CI: 1.75–3.21 log-rank *p* <0.001). The table below shows the number of patients at risk over time for each group.

(E) in genomic model figures and (C) in integrated clinico-genomic model figures: 1-, 3-, and 5-Year Calibration Curves: Assesses the agreement between the predicted

and observed overall survival probabilities at 1, 3, and 5 years, respectively. The diagonal dashed line represents perfect calibration.

(F) in genomic model figures and (D) in integrated clinico-genomic model figures: Calibration Slope Plot: Further evaluates the model's calibration, showing the relationship between predicted linear predictor and Martingale residuals. The calibration slope of genomic model was 2.719 (95%CI: 1.797-4.114). The calibration slope of integrated clinico-genomic model was 2.719 (95%CI: 2.126-3.479).

(G) in genomic model figures and (E) in integrated clinico-genomic model figures: Time-Dependent Receiver Operating Characteristic (ROC) Curves: Illustrates the discriminatory ability of the model over time. The area under the curve (AUC) values of genomic model were 0.653 (0.580-0.727), 0.651 (0.587-0.716), and 0.609 (0.526-0.694) for 1-, 3-, and 5-year survival, respectively. The AUC values of integrated clinico-genomic model were 0.695 (0.617-0.762), 0.720 (0.660-0.783), and 0.701 (0.621-0.771) for 1-, 3-, and 5-year survival, respectively.

(H) in genomic model figures and (F) in integrated clinico-genomic model figures: Decision Curve Analysis (DCA): Evaluates the clinical utility of the prognostic model by quantifying the net benefit across a range of threshold probabilities. The maximum net benefit (Max NB) and corresponding threshold probability (TP) for 1-, 3-, and 5-year survival in genomic model were: 1-year Max NB =0.083 (TP: 0.04); 3-year Max NB = 0.285 (TP: 0.14); 5-year Max NB = 0.461 (TP: 0.26). The Max NB and TP for 1-, 3-, and 5-year survival in integrated clinico-genomic model were: 1-year Max NB = 0.111 (TP: 0.01); 3-year Max NB = 0.378 (TP: 0.1); 5-year Max NB = 0.597 (TP: 0.01).

***Genomic prognostic model for LUSC*:**

Univariate Cox (P < 0.2) + Multivariate Cox

Risk Score = 0.0534 * gene_ADA + 0.1086 * gene_PKM

(A)

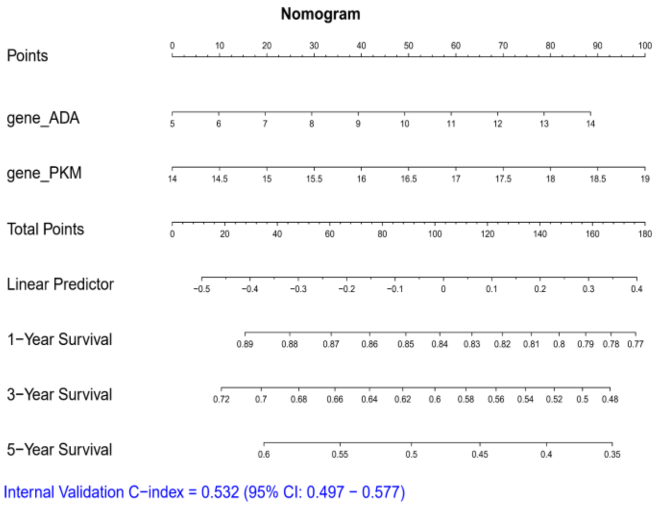

(B)

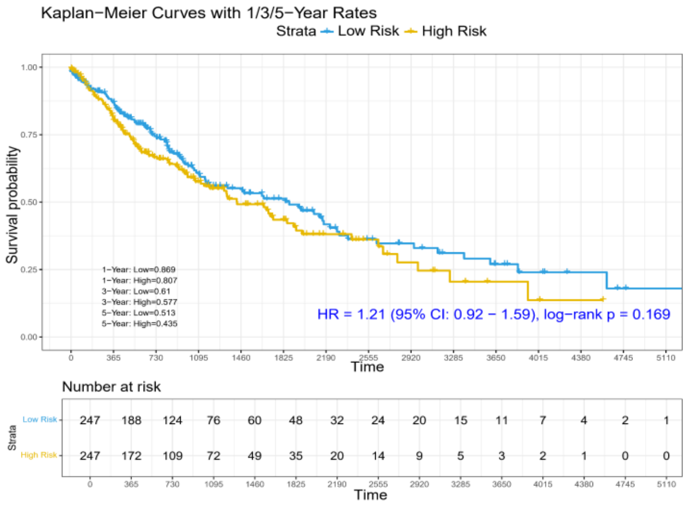

(C)

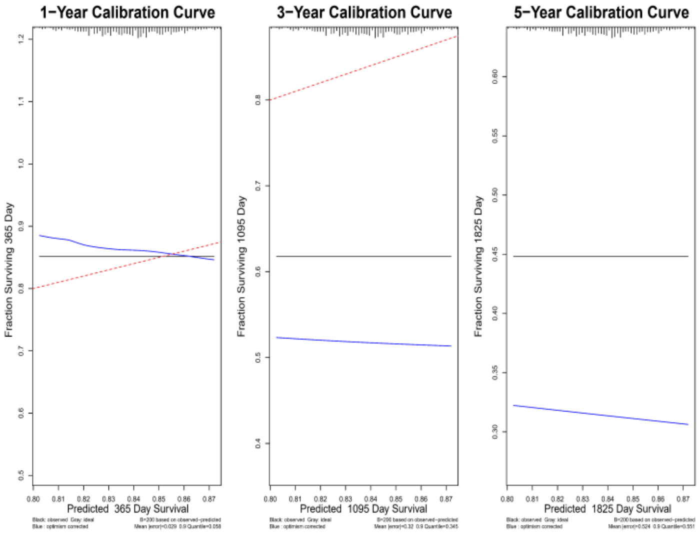

(D)

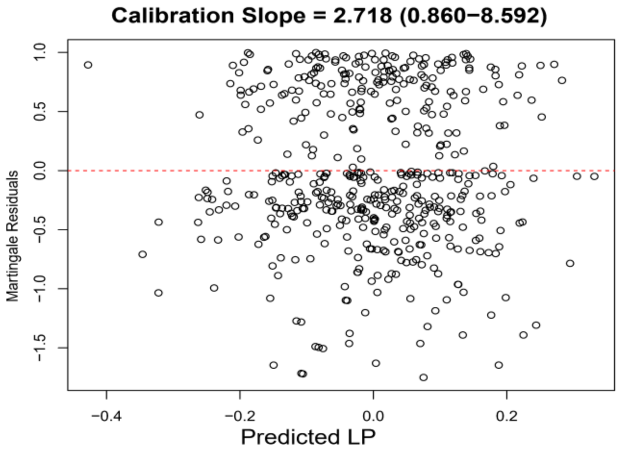

(E)

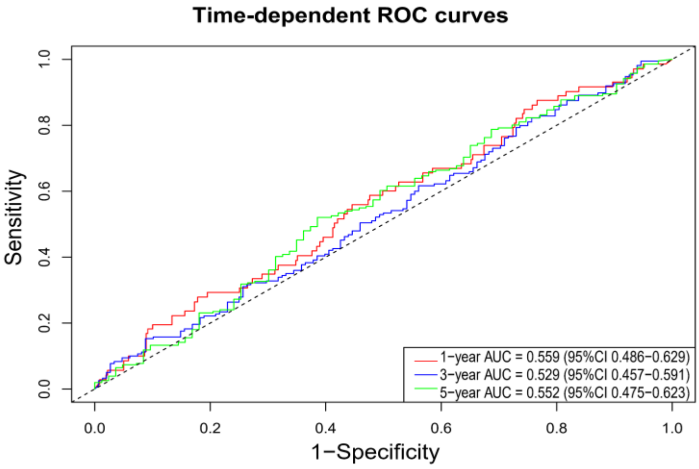

(F)

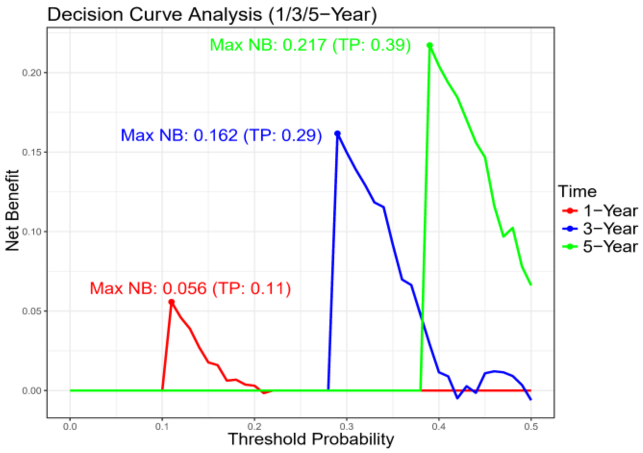

Clinico-genomic prognostic model for LUSC:

Univariate Cox (P < 0.2) + Multivariate Cox

Risk Score = 0.9639 * risk_score_gene + 0.0176 * age + 0.5152 * stage + -0.1557 * race black or african american + -0.9351 * race unknow + -0.6176 * race white + 0.2857 * gender Male

(A)

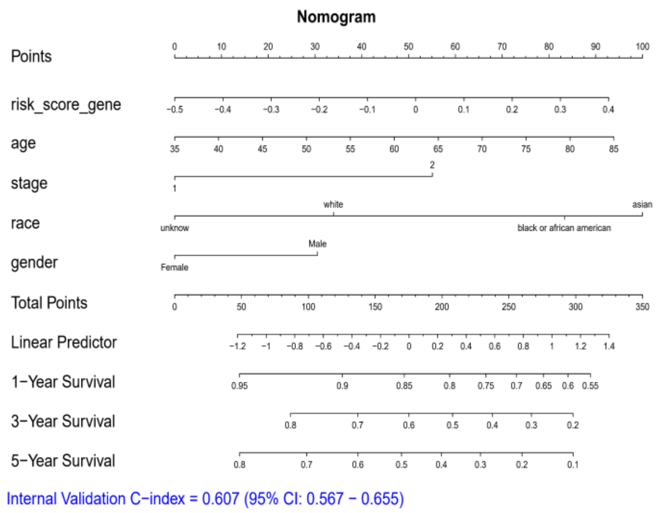

(B)

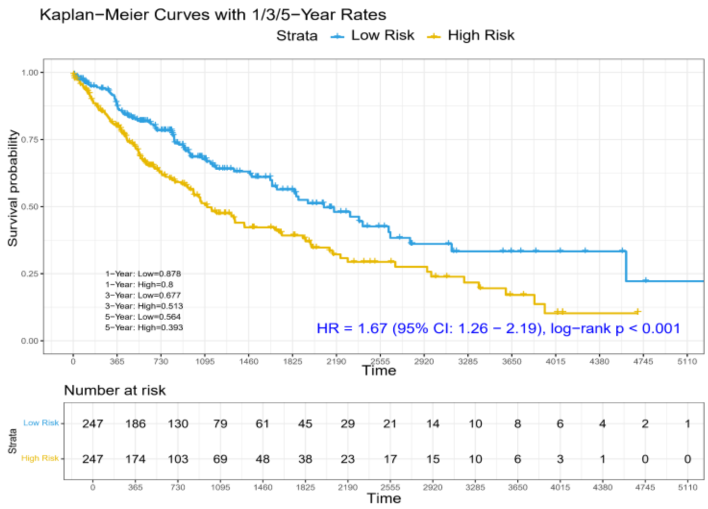

(C)

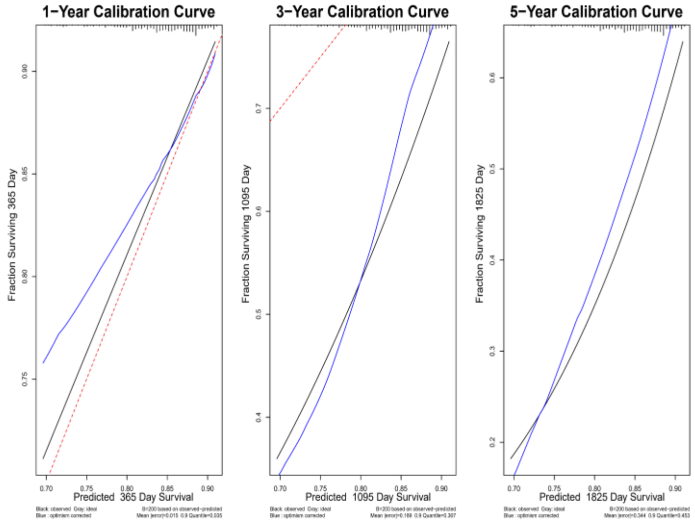

(D)

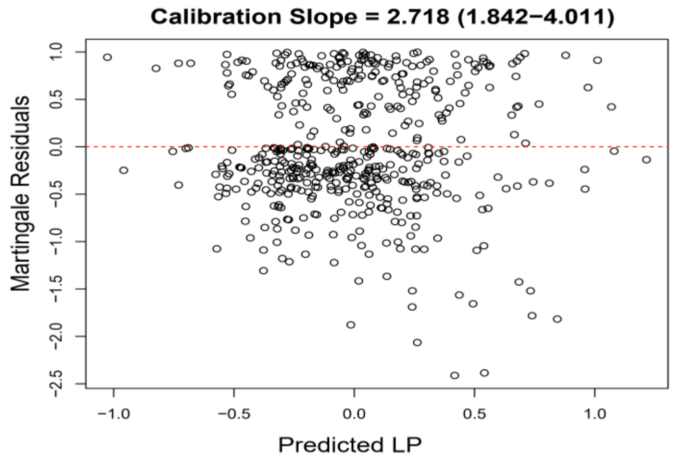

(E)

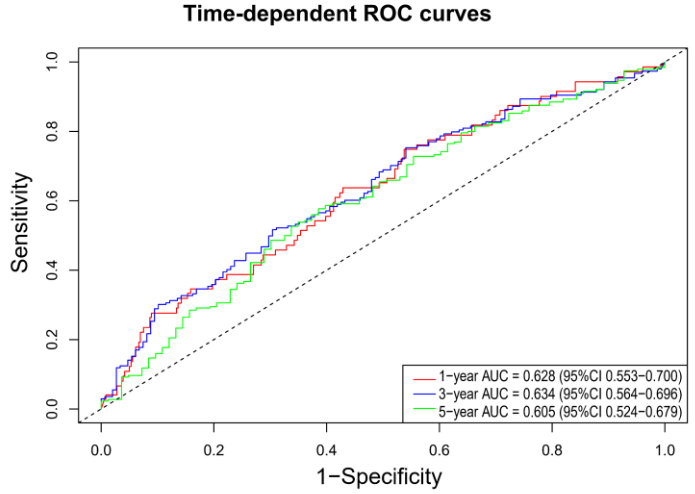

(F)

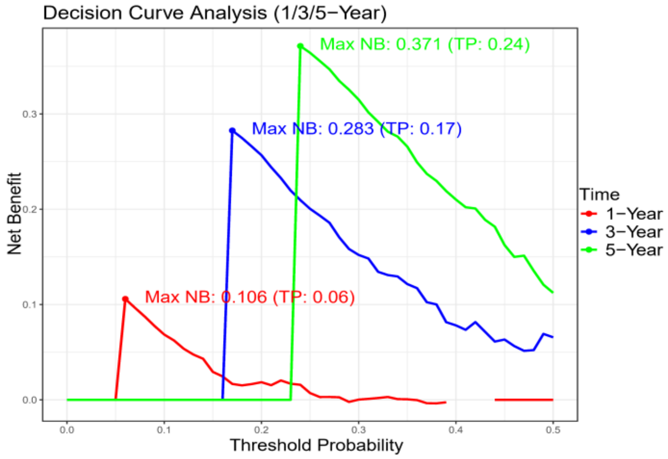

Supplementary Figure S22. Comprehensive evaluation of the prognostic models for LUSC.

The models' construction followed the procedure: Univariate Cox (*P* <0.2) + Multivariate Cox. The resulting risk score formula of genomic model is: Risk Score = 0.0534 * gene_ADA + 0.1086 * gene_PKM. The resulting risk score formula of integrated clinico-genomic model is: Risk Score = 0.9639 * risk_score_gene + 0.0176 * age + 0.5152 * stage + -0.1557 * race black or african american + -0.9351 * race unknow + -0.6176 * race white + 0.2857 * gender Male.

(A) Nomogram for Survival Prediction: A graphical representation of the prognostic model, allowing for the visual estimation of 1-, 3-, and 5-year overall survival probabilities based on individual gene expression levels (and clinical features, if clinico-genomic model). The genomic model's internal validation C-index was 0.532 (95%CI: 0.497-0.577), integrated clinico-genomic model's internal validation C-index was 0.607 (95%CI: 0.567-0.655).

(B) Kaplan-Meier Survival Curves: Survival analysis demonstrated a statistically significant differences in overall survival between the high- and low-risk groups in the integrated clinico-genomic model (HR = 1.67, 95% CI: 1.26–2.19, log-rank p <0.001), whereas no significant difference was observed for the genomic model (HR = 1.21, 95% CI: 0.92–1.59, log-rank p = 0.169). The table below shows the number of patients at risk over time for each group.

(C) 1-, 3-, and 5-Year Calibration Curves: Assesses the agreement between the predicted and observed overall survival probabilities at 1, 3, and 5 years, respectively. The diagonal dashed line represents perfect calibration.

(D) Calibration Slope Plot: Further evaluates the model's calibration, showing the relationship between predicted linear predictor and Martingale residuals. The calibration slope of genomic model was 2.718 (95%CI: 0.860-8.592). The calibration slope of integrated clinico-genomic model was 2.718 (95%CI: 1.842-4.011).

(E) Time-Dependent Receiver Operating Characteristic (ROC) Curves: Illustrates the discriminatory ability of the model over time. The area under the curve (AUC) values of genomic model were 0.559 (0.486-0.629), 0.529 (0.457-0.591), and 0.552 (0.475-

0.623) for 1-, 3-, and 5-year survival, respectively. The AUC values of integrated clinico-genomic model were 0.628 (0.553-0.700), 0.634 (0.564-0.696), and 0.605 (0.524-0.679) for 1-, 3-, and 5-year survival, respectively.

(F) Decision Curve Analysis (DCA): Evaluates the clinical utility of the prognostic model by quantifying the net benefit across a range of threshold probabilities. The maximum net benefit (Max NB) and corresponding threshold probability (TP) for 1-, 3-, and 5-year survival in genomic model were: 1-year Max NB =0.056 (TP: 0.11); 3-year Max NB = 0.162 (TP: 0.29); 5-year Max NB = 0.217 (TP: 0.39). The Max NB and TP for 1-, 3-, and 5-year survival in integrated clinico-genomic model were: 1-year Max NB =0.106 (TP: 0.06); 3-year Max NB = 0.283 (TP: 0.17); 5-year Max NB = 0.371 (TP: 0.24).

Genomic prognostic model for PAAD:

Univariate Cox (P < 0.2) + LASSO with 10-fold CV (lambda.min) + Multivariate Cox/Stepwise Cox (AIC/BIC)

Risk Score = 0.1034 * gene_HKDC1 + 0.2761 * gene_RRM2

(A)

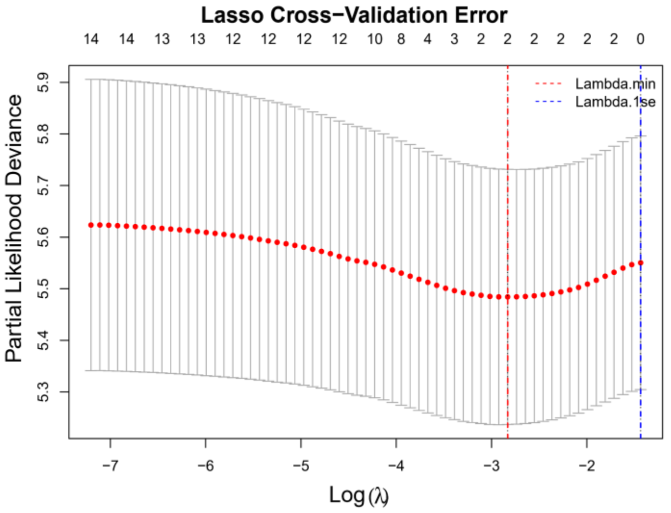

(B)

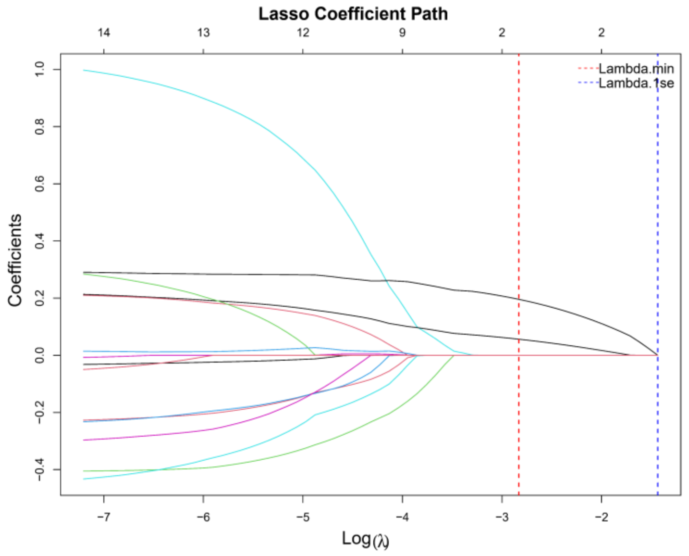

(C)

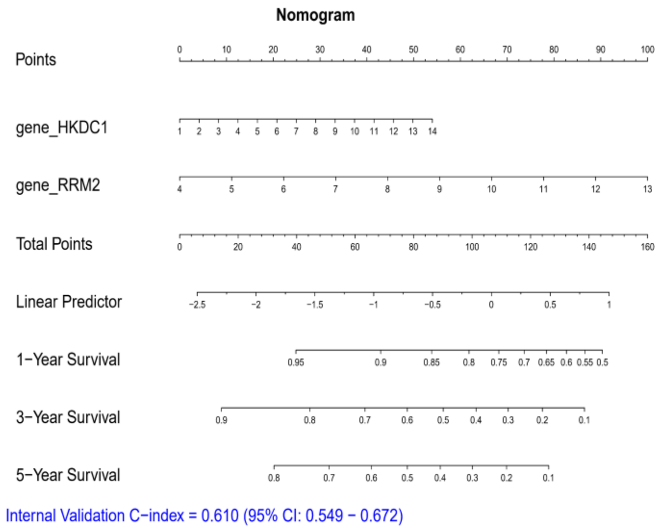

(D)

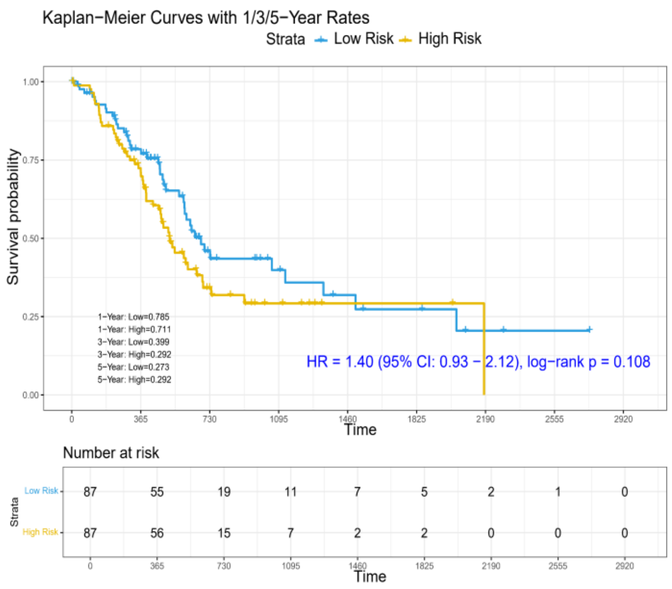

(E)

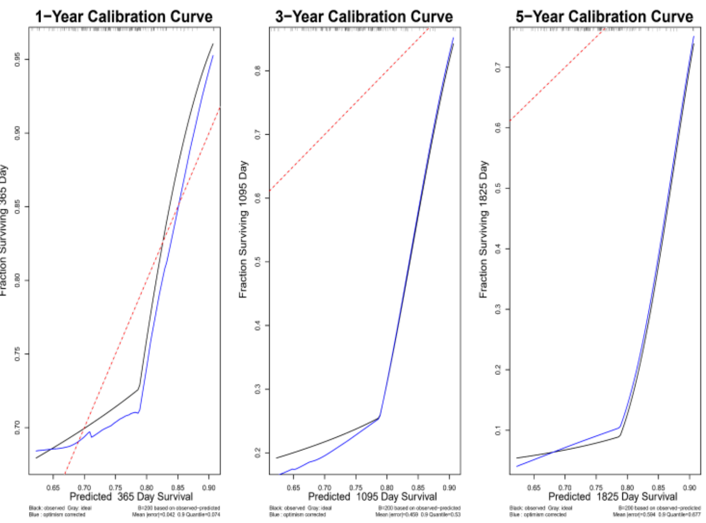

(F)

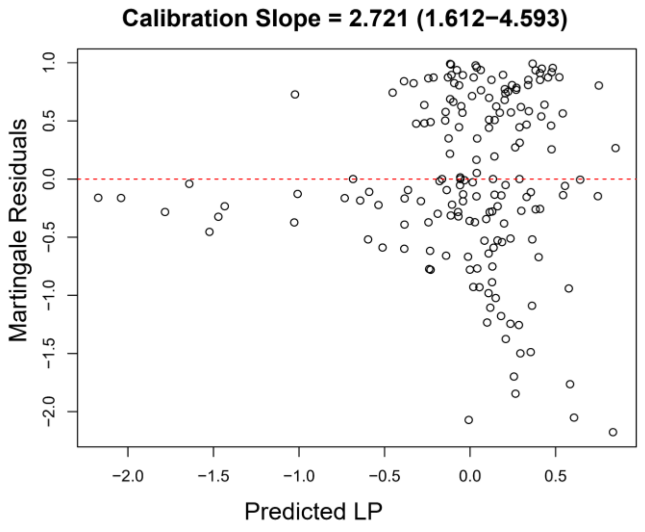

(G)

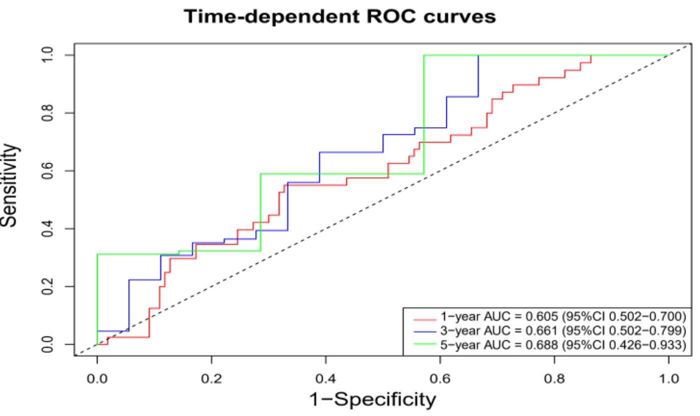

(H)

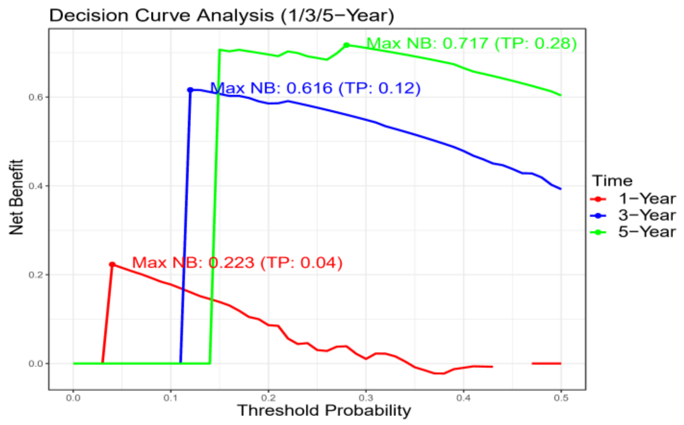

Clinico-genomic prognostic model for PAAD:

Univariate Cox ($P < 0.2$) + LASSO with 10-fold CV (lambda.min) + Multivariate Cox/Stepwise Cox (AIC/BIC)

Risk Score = 0.9836 * risk_score_gene + 0.0206 * age + -0.3658 * stage + 0.3228 * race black or african american + 0.636 * race unknow + 0.3715 * race white

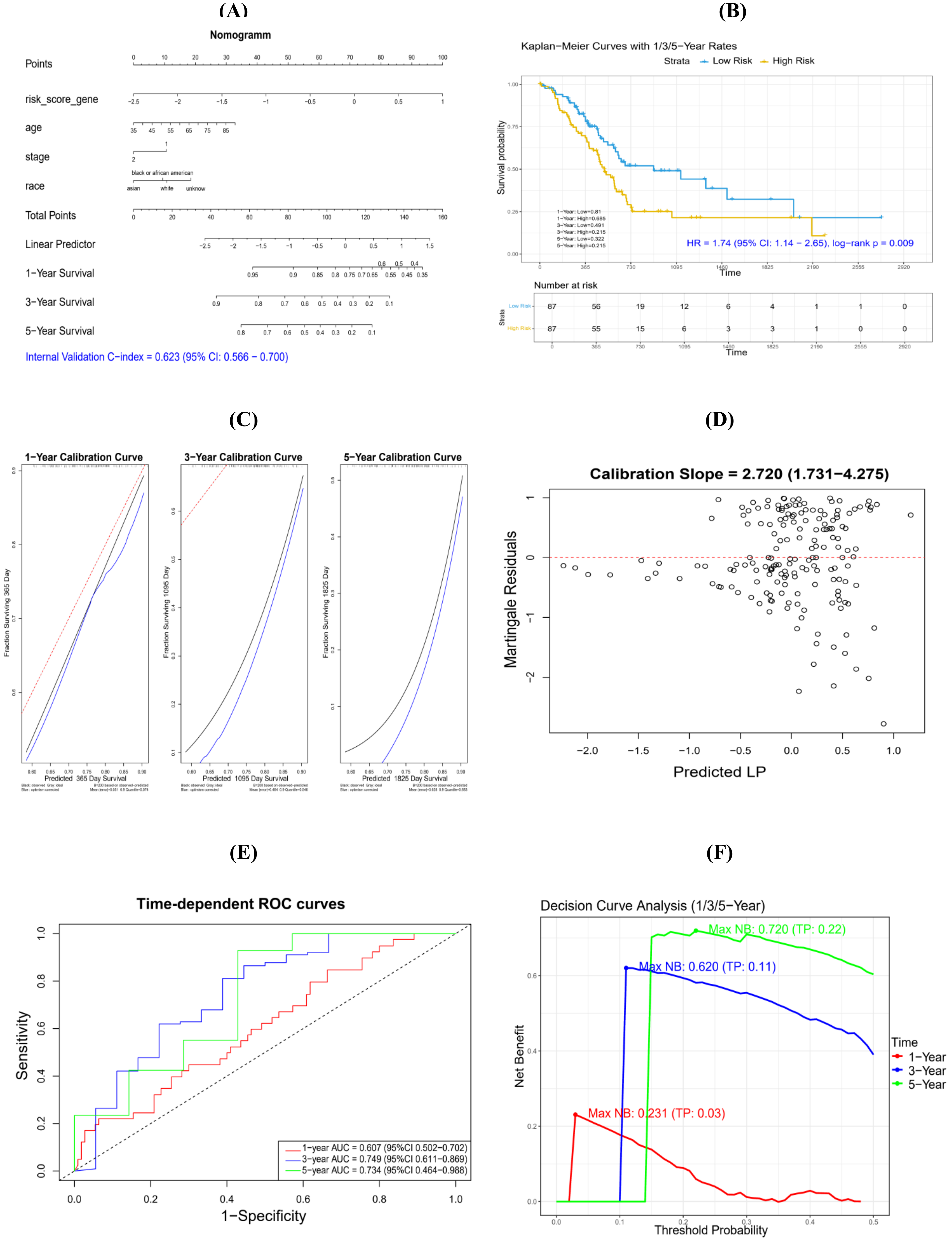

Supplementary Figure S23. Comprehensive evaluation of the prognostic models for PAAD. The models' construction followed the procedure: Univariate Cox (*P* <0.2) + LASSO with 10-fold CV (lambda.min) + Multivariate Cox/Stepwise Cox (AIC/BIC). The resulting risk score formula of genomic model is: Risk Score = 0.1034 * gene_HKDC1 + 0.2761 * gene_RRM2. The resulting risk score formula of integrated clinico-genomic model is: Risk Score = 0.9836 * risk_score_gene + 0.0206 * age + -0.3658 * stage + 0.3228 * race black or african american + 0.636 * race unknow + 0.3715 * race white.

(A) Lasso Cross-Validation Error Plot: Depicts the cross-validation error distribution across different lambda (λ) values, indicating the optimal lambda values (lambda.min and lambda.1se) for penalty parameter selection in LASSO regression. The best-performing model for this tumor type used lambda.min, with a λ value of 0.059.

(B) Lasso Coefficient Path: Illustrates how the coefficients of the selected genes change as the lambda (λ) regularization parameter varies, demonstrating the variable selection process.

(C) in genomic model figures and (A) in integrated clinico-genomic model figures: Nomogram for Survival Prediction: A graphical representation of the prognostic model, allowing for the visual estimation of 1-, 3-, and 5-year overall survival probabilities based on individual gene expression levels (and clinical features, if clinico-genomic model). The genomic model's internal validation C-index was 0.610 (95%CI: 0.549-0.672), integrated clinico-genomic model's internal validation C-index was 0.623 (95%CI: 0.566-0.700).

(D) in genomic model figures and (B) in integrated clinico-genomic model figures: Kaplan-Meier Survival Curves: Survival analysis demonstrated a statistically significant differences in overall survival between the high- and low-risk groups in the integrated clinico-genomic model (HR = 1.74, 95% CI: 1.14–2.65, log-rank $p = 0.009$), whereas no significant difference was observed in the genomic model (HR = 1.40, 95% CI: 0.93–2.12, log-rank $p = 0.108$). The table below shows the number of patients at risk over time for each group.

(E) in genomic model figures and (C) in integrated clinico-genomic model figures:

1-, 3-, and 5-Year Calibration Curves: Assesses the agreement between the predicted and observed overall survival probabilities at 1, 3, and 5 years, respectively. The diagonal dashed line represents perfect calibration.

(F) in genomic model figures and (D) in integrated clinico-genomic model figures: Calibration Slope Plot: Further evaluates the model's calibration, showing the relationship between predicted linear predictor and Martingale residuals. The calibration slope of genomic model was 2.721 (95%CI: 1.612-4.593). The calibration slope of integrated clinico-genomic model was 2.720 (95%CI: 1.731-4.275).

(G) in genomic model figures and (E) in integrated clinico-genomic model figures: Time-Dependent Receiver Operating Characteristic (ROC) Curves: Illustrates the discriminatory ability of the model over time. The area under the curve (AUC) values of genomic model were 0.605 (0.502-0.700), 0.661 (0.502-0.799), and 0.688 (0.426-0.933) for 1-, 3-, and 5-year survival, respectively. The AUC values of integrated clinico-genomic model were 0.607 (0.502-0.702), 0.749 (0.611-0.869), and 0.734 (0.464-0.988) for 1-, 3-, and 5-year survival, respectively.

(H) in genomic model figures and (F) in integrated clinico-genomic model figures: Decision Curve Analysis (DCA): Evaluates the clinical utility of the prognostic model by quantifying the net benefit across a range of threshold probabilities. The maximum net benefit (Max NB) and corresponding threshold probability (TP) for 1-, 3-, and 5-year survival in genomic model were: 1-year Max NB =0.223 (TP: 0.04); 3-year Max NB = 0.616 (TP: 0.12); 5-year Max NB = 0.717 (TP: 0.28). The Max NB and TP for 1-, 3-, and 5-year survival in integrated clinico-genomic model were: 1-year Max NB = 0.231 (TP: 0.03); 3-year Max NB = 0.620 (TP: 0.11); 5-year Max NB = 0.720 (TP: 0.22).

Genomic prognostic model for PRAD:

Univariate Cox (P < 0.2) + LASSO with 10-fold CV (lambda.min) + Multivariate Cox

Risk Score = 1.0725 * gene_APRT

(A)

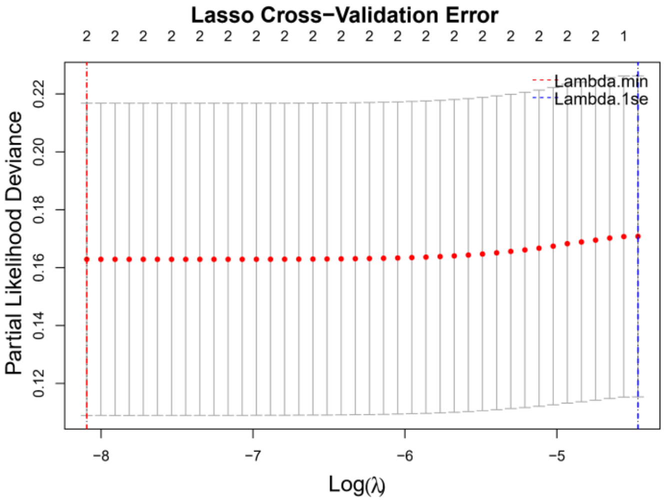

(B)

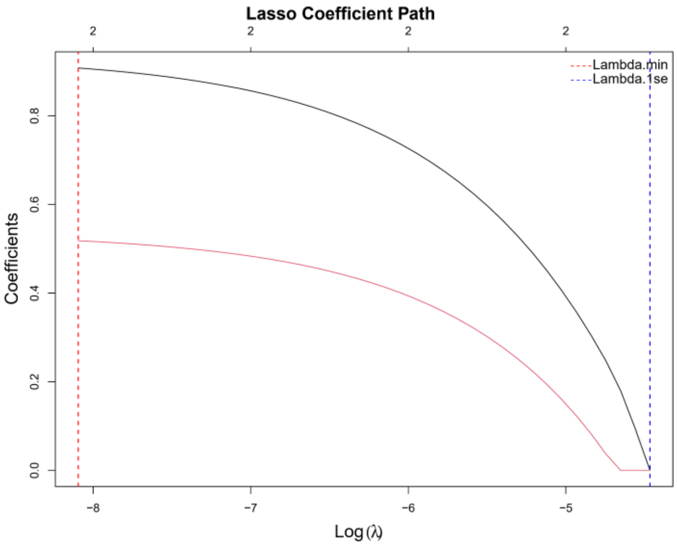

(C)

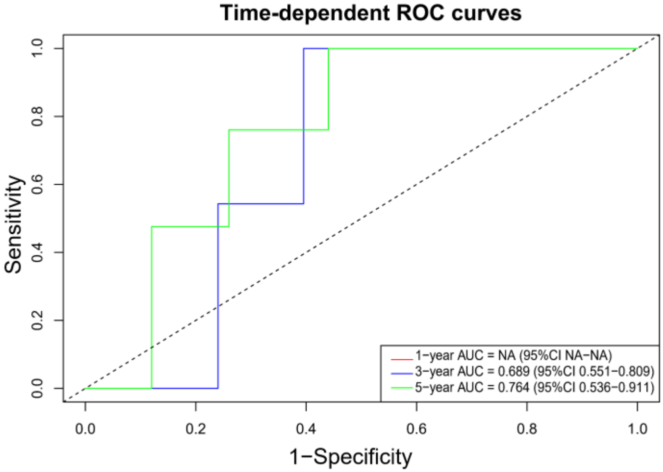

Supplementary Figure S24. Comprehensive evaluation of the prognostic models for PRAD. The model's construction followed the procedure: Univariate Cox (*P* <0.2) + LASSO with 10-fold CV (lambda.min) + Multivariate Cox. The resulting risk score formula of genomic model is: Risk Score = 1.0725 * gene_APRT. Notably, due to insufficient survival data and biased distribution of clinical information, clinico-genomic models could not be constructed or evaluated for this tumor type. The genomic model's internal validation C-index was 0.607 (95%CI: 0.545-0.669). The calibration slope of genomic model was 2.718 (95%CI: 0.787-9.389).

(A) Lasso Cross-Validation Error Plot: Depicts the cross-validation error distribution across different lambda (λ) values, indicating the optimal lambda values (lambda.min and lambda.1se) for penalty parameter selection in LASSO regression. The best-performing model for this tumor type used lambda.min, with a λ value of 0.278.

(B) Lasso Coefficient Path: Illustrates how the coefficients of the selected genes change as the lambda (λ) regularization parameter varies, demonstrating the variable selection process.

(C) Time-Dependent Receiver Operating Characteristic (ROC) Curves: Illustrates the discriminatory ability of the model over time. The area under the curve (AUC) values of genomic model were 0.609 (0.501-0.705), 0.695 (0.536-0.825), and 0.704 (0.453-0.954) for 1-, 3-, and 5-year survival, respectively. The AUC values of integrated clinico-genomic model were 0.607 (0.502-0.702), 0.749 (0.611-0.869), and 0.734 (0.464-0.988) for 1-, 3-, and 5-year survival, respectively.

Genomic prognostic model for READ:
Univariate Cox (P < 0.2) + LASSO with 10-fold CV (lambda.min) + Multivariate Cox
Risk Score = 0.9025 * gene_APRT + 0.5665 * gene_PAICS

(A)

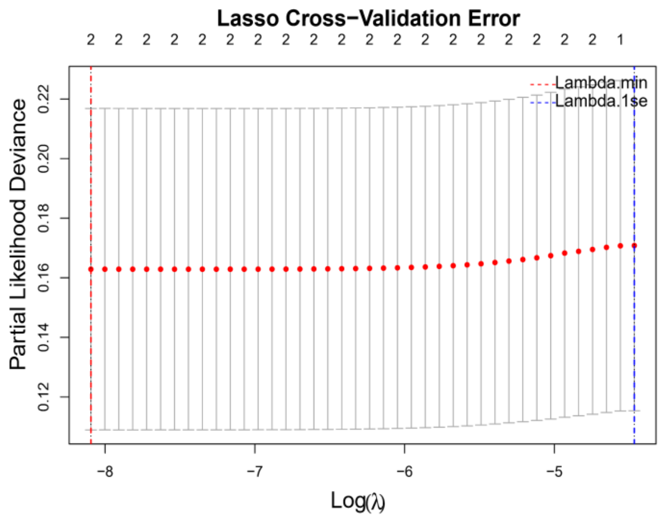

(B)

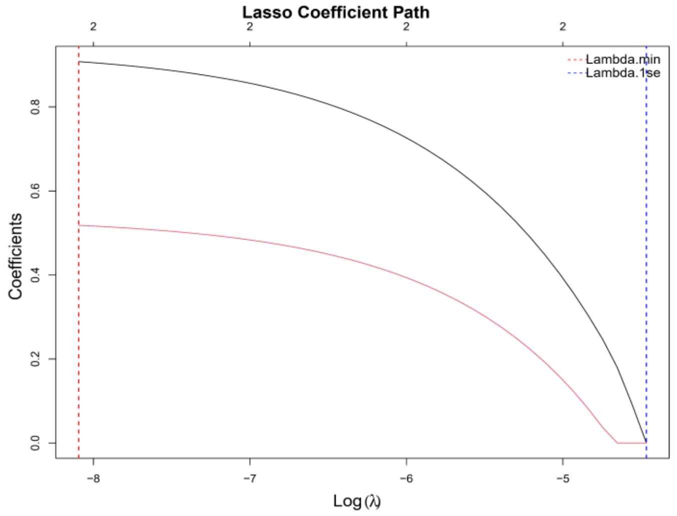

(C)

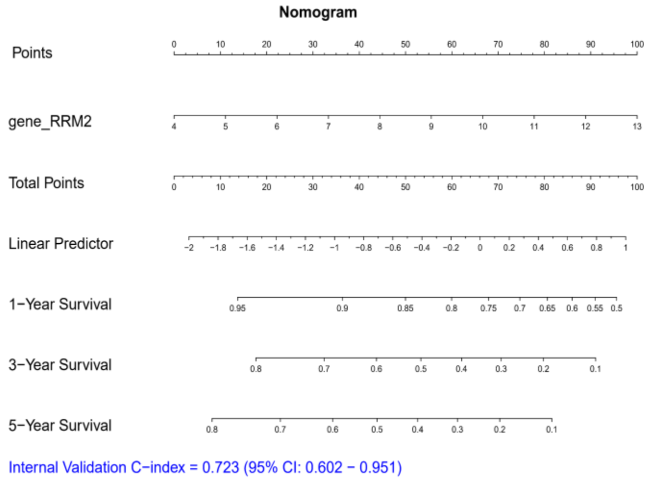

(D)

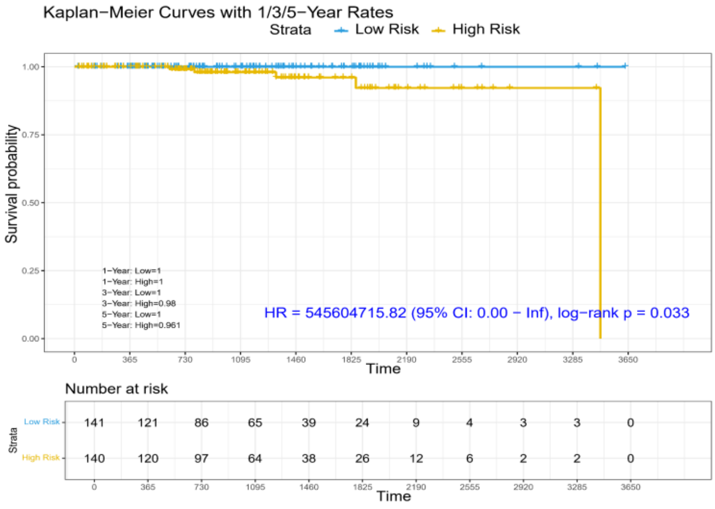

(E)

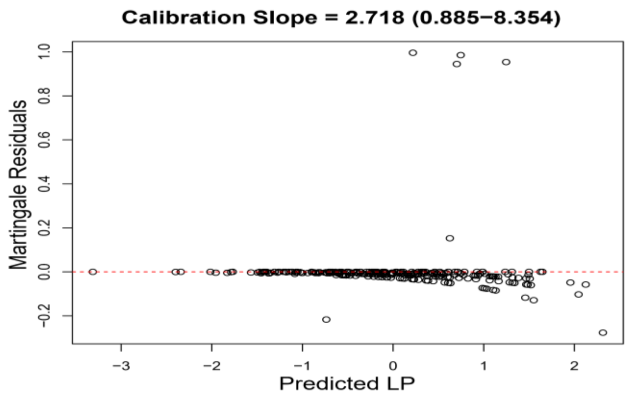

(F)

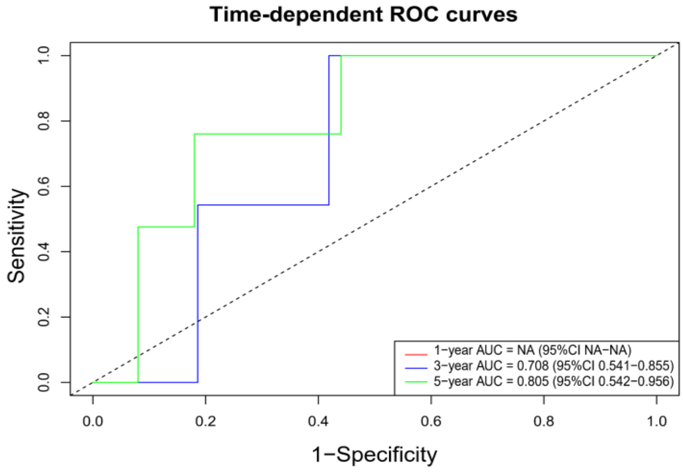

(G)

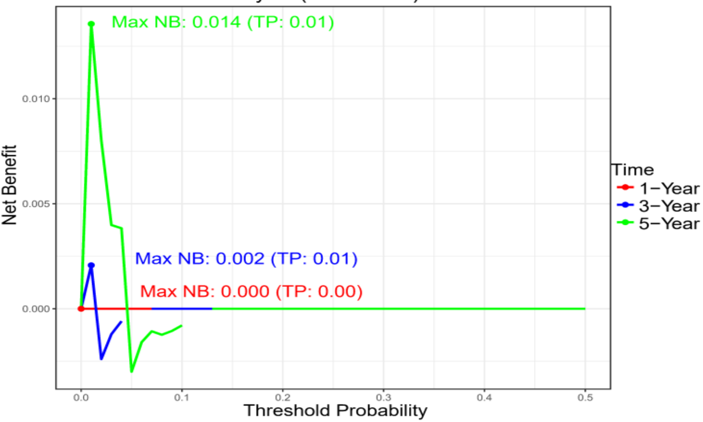

Supplementary Figure S25. Comprehensive evaluation of the prognostic models for READ. The model's construction followed the procedure: Univariate Cox (*P* <0.2) + LASSO with 10-fold CV (lambda.min) + Multivariate Cox. The resulting risk score formula of genomic model is: Risk Score = 0.9025 * gene_APRT + 0.5665 * gene_PAICS. Notably, due to insufficient survival data and biased distribution of clinical information, clinico-genomic models could not be constructed or evaluated for this tumor type. The genomic model's internal validation C-index was 0.723 (95%CI: 0.602-0.951). The calibration slope of genomic model was 2.718 (95%CI: 0.884-8.354).

(A) Lasso Cross-Validation Error Plot: Depicts the cross-validation error distribution across different lambda (λ) values, indicating the optimal lambda values (lambda.min and lambda.1se) for penalty parameter selection in LASSO regression. The best-performing model for this tumor type used lambda.min, with a λ value of 0.059.

(B) Lasso Coefficient Path: Illustrates how the coefficients of the selected genes change as the lambda (λ) regularization parameter varies, demonstrating the variable selection process.

(C) Nomogram for Survival Prediction: A graphical representation of the prognostic model, allowing for the visual estimation of 1-, 3-, and 5-year overall survival probabilities based on individual gene expression levels (and clinical features, if clinico-genomic model). The genomic model's internal validation C-index was 0.723 (95%CI: 0.602-0.951).

(D) Kaplan-Meier Survival Curves: Kaplan–Meier analysis yielded extremely unstable hazard ratio estimates (HR = 5.46 × 10^8, 95% CI: 0.00–inf, log-rank p = 0.033) due to data separation and scarcity of events, making the HR non-interpretable. In this tumor type, the HR values are presented for completeness but should be interpreted with caution.

(E) Calibration Slope Plot: Further evaluates the model's calibration, showing the relationship between predicted linear predictor and Martingale residuals. The calibration slope of genomic model was 2.718 (95%CI: 0.884-8.354).

(F) Time-Dependent Receiver Operating Characteristic (ROC) Curves: Illustrates the

discriminatory ability of the model over time. The area under the curve (AUC) values of genomic model were 0.708 (0.541-0.855), and 0.805 (0.542-0.956) for 3-, and 5-year survival, respectively. Owing to insufficient survival events within 1 year, the time-dependent ROC curve for 1-year survival could not be calculated.

(G) Decision Curve Analysis (DCA): Evaluates the clinical utility of the prognostic model by quantifying the net benefit across a range of threshold probabilities. The maximum net benefit (Max NB) and corresponding threshold probability (TP) for 1-, 3-, and 5-year survival in genomic model were: 3-year Max NB = 0.002 (TP: 0.01); 5-year Max NB = 0.014 (TP: 0.01). Owing to insufficient survival events within 1 year, DCA curve for 1-year survival also could not be calculated.

Genomic prognostic model for STAD:

Univariate Cox (P < 0.6) + Multivariate Cox

Risk Score = 0.0537 * gene_IMPDH1

(A)

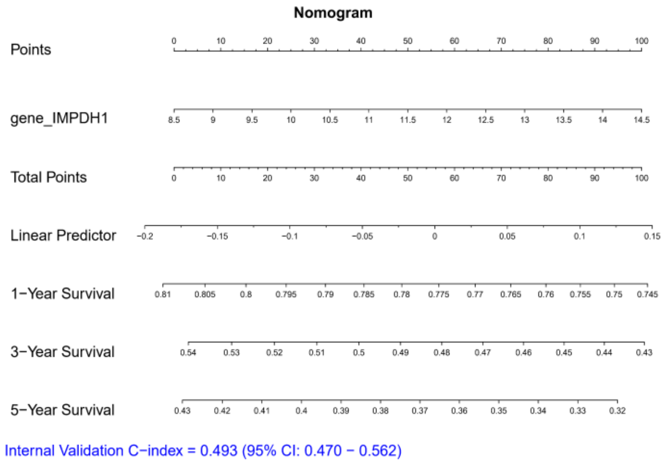

(B)

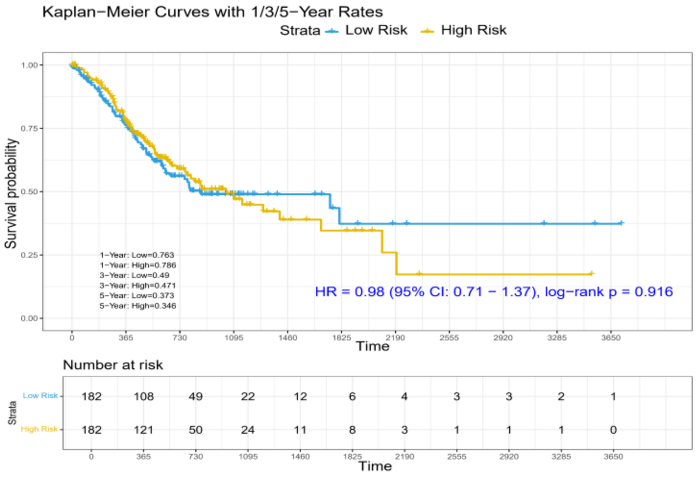

(C)

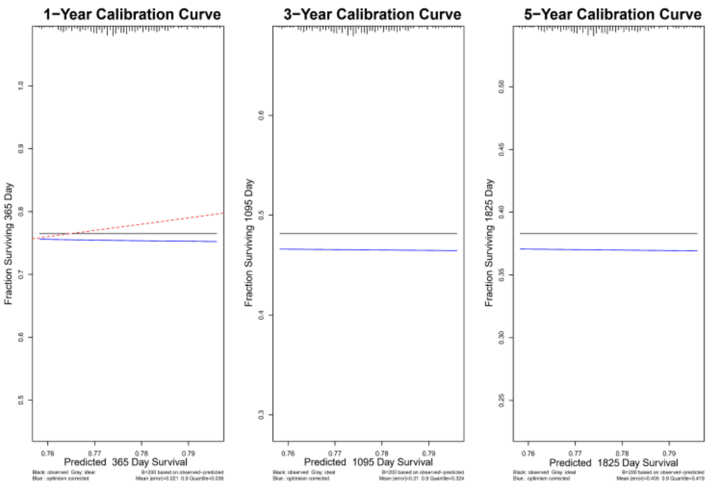

(D)

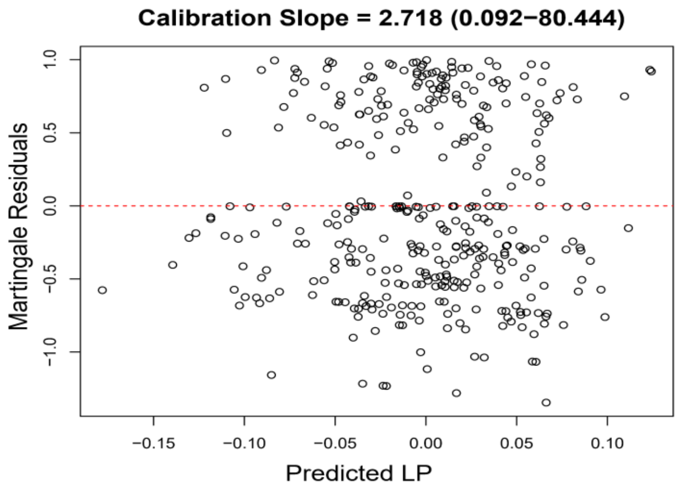

(E)

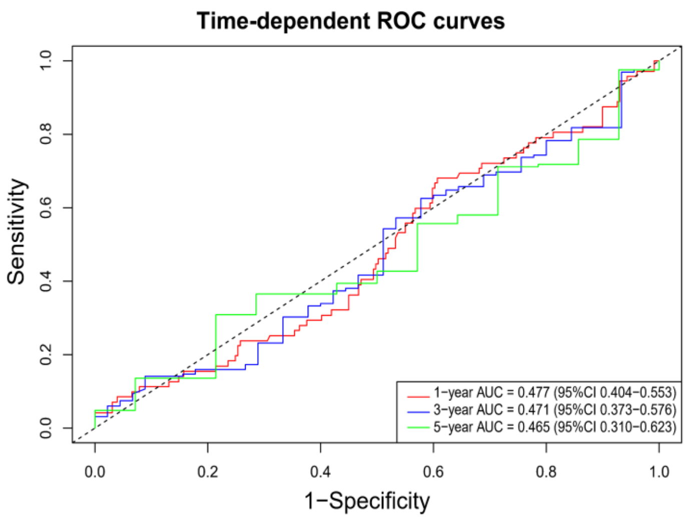

(F)

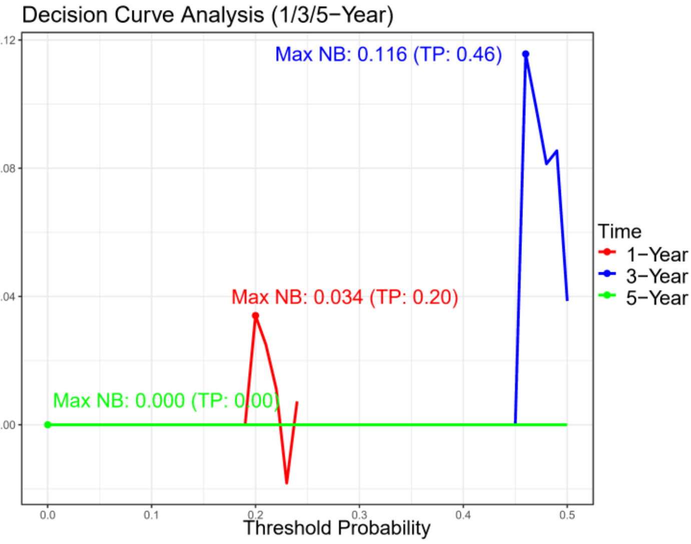

Clinico-genomic prognostic model for STAD:

Univariate Cox (P < 0.6) + Multivariate Cox

Risk Score = -0.1175 * risk_score_gene + 0.0228 * age + 0.7147 * stage + 0.347 * race black or african american + 0.273 * race unknow + 0.2355 * race white + 0.2354 * gender Male

(A)

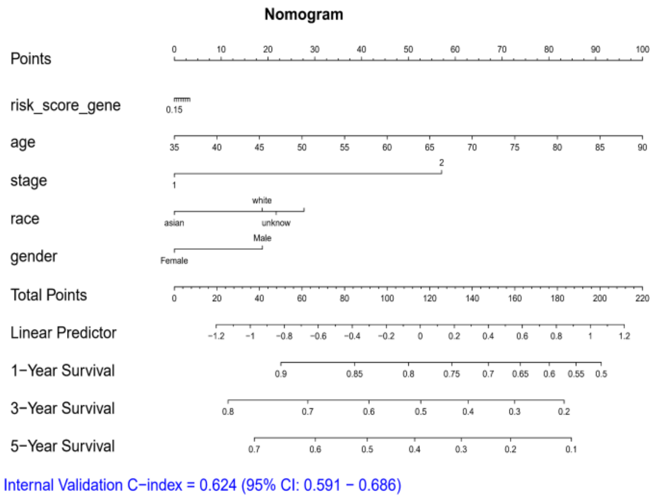

(B)

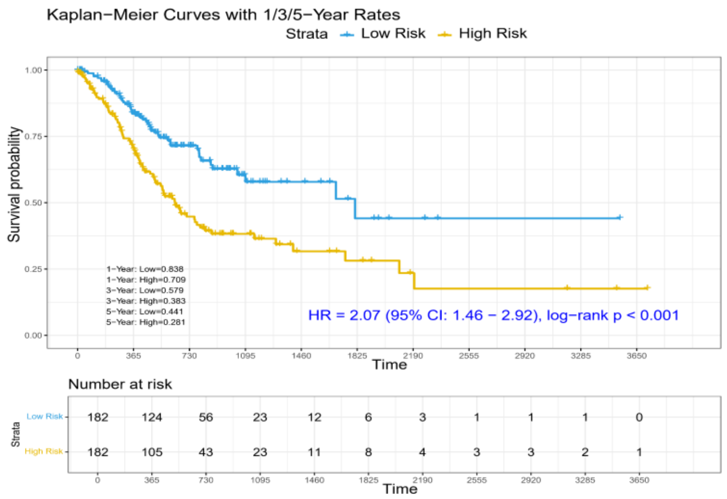

(C)

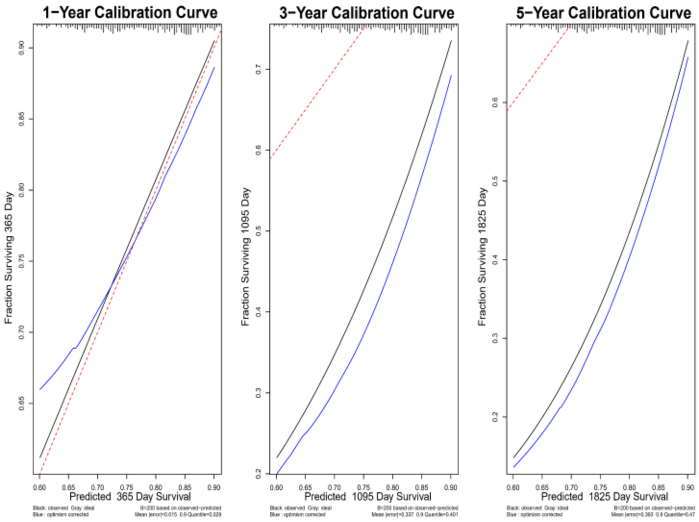

(D)

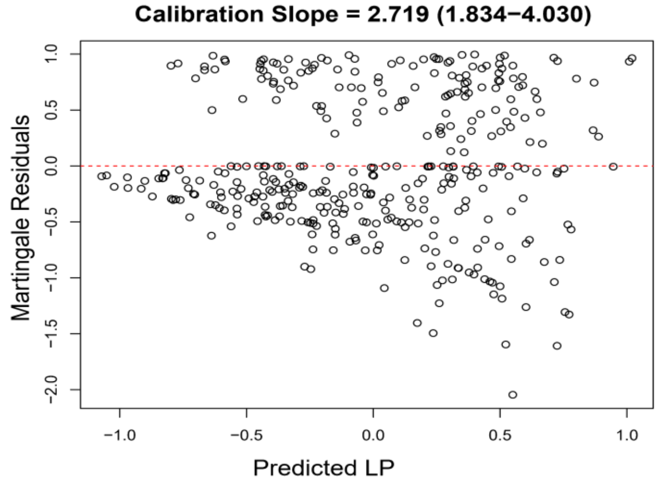

(E)

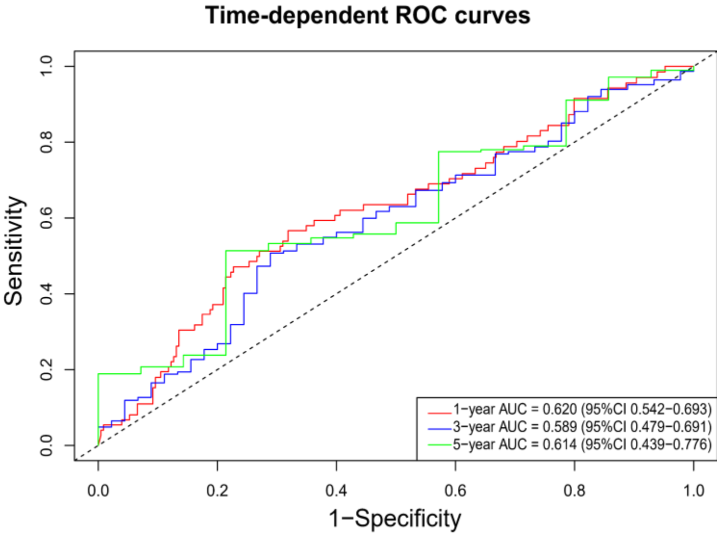

(F)

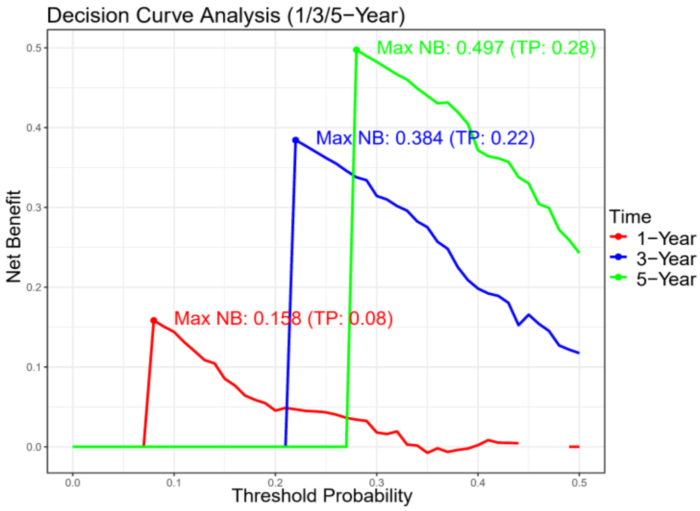

Supplementary Figure S26. Comprehensive evaluation of the prognostic models for STAD.

The models' construction followed the procedure: Univariate Cox (*P* <0.6) + Multivariate Cox. The resulting risk score formula of genomic model is: Risk Score = 0.0537 * gene_IMPDH1. The resulting risk score formula of integrated clinico-genomic model is: Risk Score = -0.1175 * risk_score_gene + 0.0228 * age + 0.7147 * stage + 0.347 * race black or african american + 0.273 * race unknow + 0.2355 * race white + 0.2354 * gender Male.

(A) Nomogram for Survival Prediction: A graphical representation of the prognostic model, allowing for the visual estimation of 1-, 3-, and 5-year overall survival probabilities based on individual gene expression levels (and clinical features, if clinico-genomic model). The genomic model's internal validation C-index was 0.493 (95%CI: 0.470-0.562), integrated clinico-genomic model's internal validation C-index was 0.624 (95%CI: 0.591-0.686).

(B) Kaplan-Meier Survival Curves: Survival analysis demonstrated a statistically significant differences in overall survival between the high- and low-risk groups in the integrated clinico-genomic model (HR = 2.07, 95% CI: 1.46–2.92, log-rank *p* <0.001), whereas no significant difference was observed for the genomic model (HR = 0.98, 95% CI: 0.71–1.37, log-rank *p* = 0.916). The table below shows the number of patients at risk over time for each group.

(C) 1-, 3-, and 5-Year Calibration Curves: Assesses the agreement between the predicted and observed overall survival probabilities at 1, 3, and 5 years, respectively. The diagonal dashed line represents perfect calibration.

(D) Calibration Slope Plot: Further evaluates the model's calibration, showing the relationship between predicted linear predictor and Martingale residuals. The calibration slope of genomic model was 2.718 (95%CI: 0.092-80.444). The calibration slope of integrated clinico-genomic model was 2.719 (95%CI: 1.834-4.030).

(E) Time-Dependent Receiver Operating Characteristic (ROC) Curves: Illustrates the discriminatory ability of the model over time. The area under the curve (AUC) values

of genomic model were 0.477 (0.404-0.553), 0.471 (0.373-0.576), and 0.465 (0.310-0.623) for 1-, 3-, and 5-year survival, respectively. The AUC values of integrated clinico-genomic model were 0.620 (0.542-0.693), 0.589 (0.479-0.691), and 0.614 (0.439-0.776) for 1-, 3-, and 5-year survival, respectively.

(F) Decision Curve Analysis (DCA): Evaluates the clinical utility of the prognostic model by quantifying the net benefit across a range of threshold probabilities. The maximum net benefit (Max NB) and corresponding threshold probability (TP) for 1-, 3-, and 5-year survival in genomic model were: 3-year Max NB = 0.034 (TP: 0.20); 5-year Max NB = 0.116 (TP: 0.46). Owing to insufficient survival events within 1 year, DCA curve for 1-year survival in genomic model could not be calculated. The Max NB and TP for 1-, 3-, and 5-year survival in integrated clinico-genomic model were: 1-year Max NB =0.158 (TP: 0.08); 3-year Max NB = 0.384 (TP: 0.22); 5-year Max NB = 0.497 (TP: 0.28).

<u>Genomic prognostic model for THCA</u>:

Univariate Cox (P < 0.2) + Multivariate Cox

Risk Score = 0.6769 * gene_IMPDH1

(A)

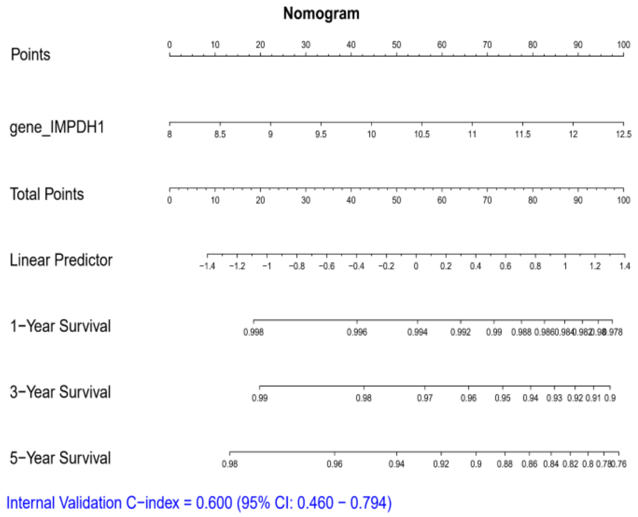

(B)

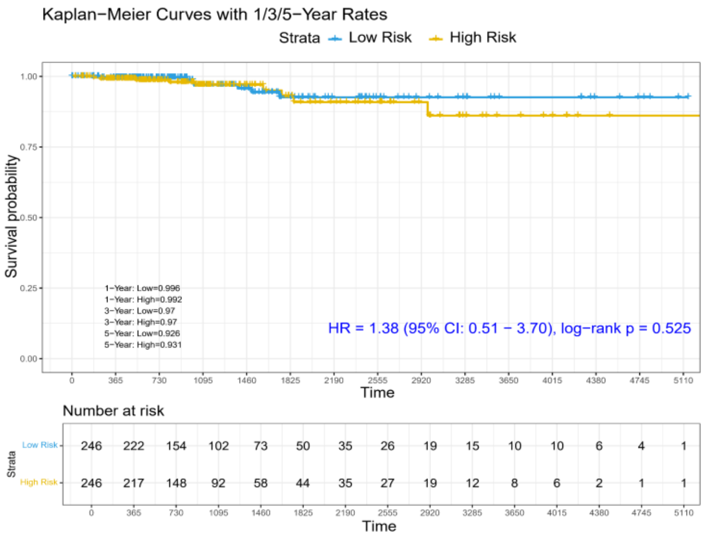

(C)

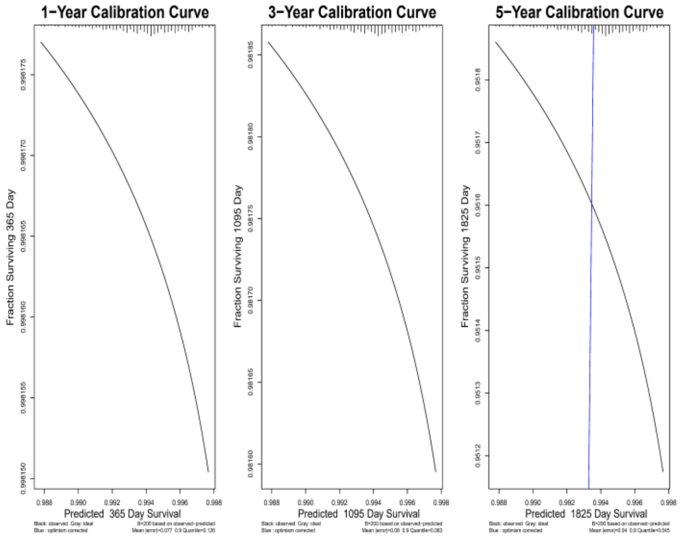

(D)

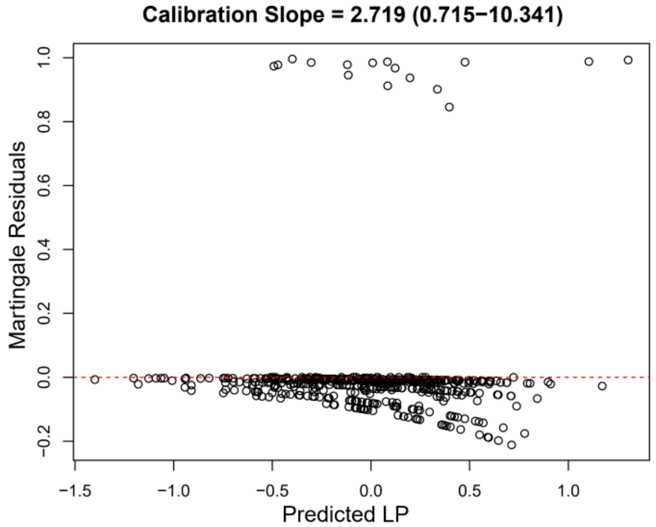

(E)

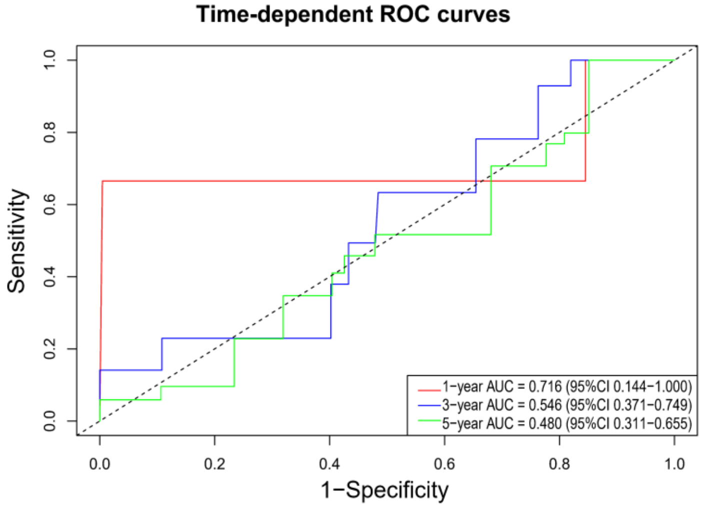

(F)

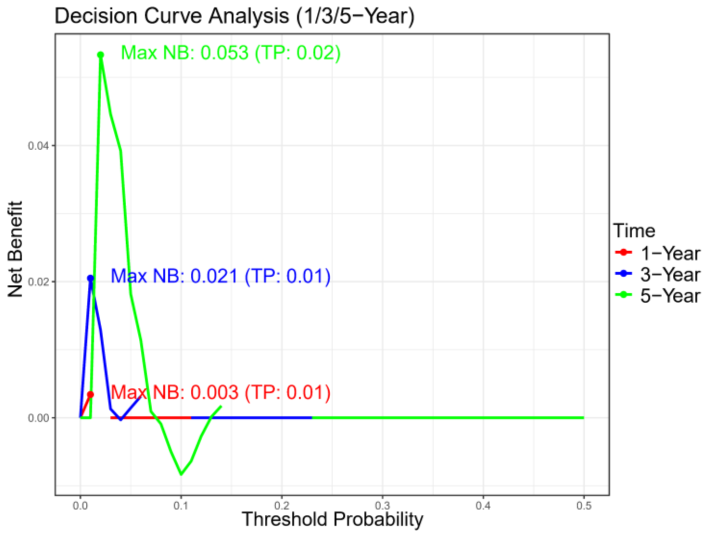

Clinico-genomic prognostic model for THCA:
Univariate Cox (P < 0.2) + Multivariate Cox
Risk Score = 0.4956 * risk_score_gene + 0.1082 * age + -0.0223 * stage + 0.376 * race asian + 18.9633 * race black or african american + 0.1647 * race unknow + 0 * race white + -0.0445 * gender Male

(A)

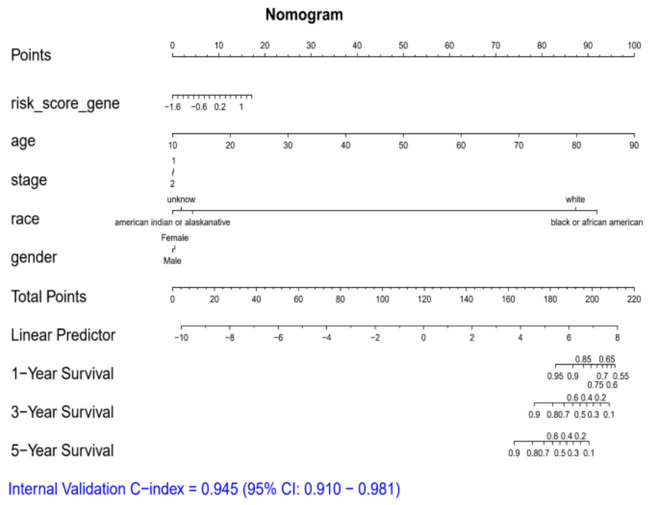

(B)

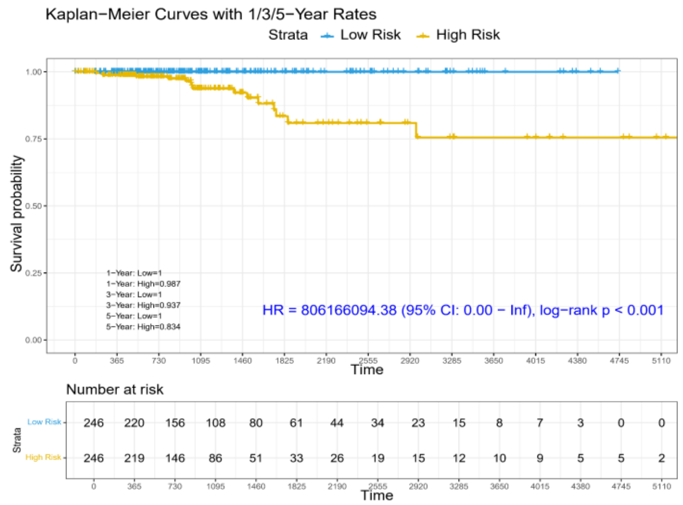

(C)

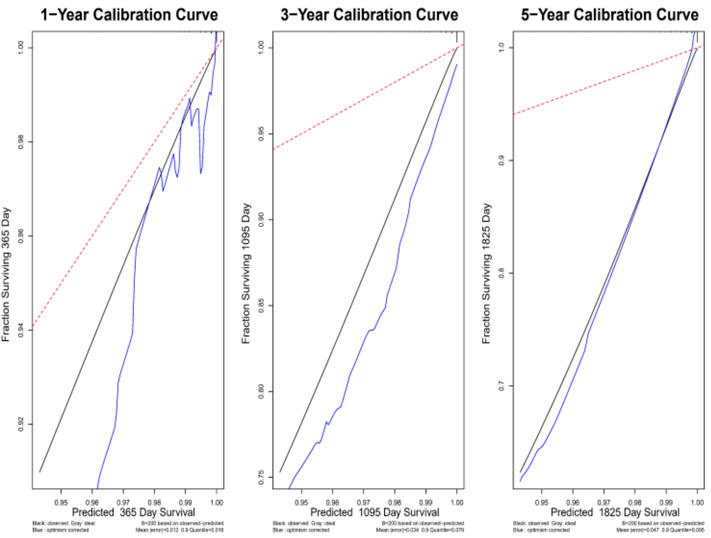

(D)

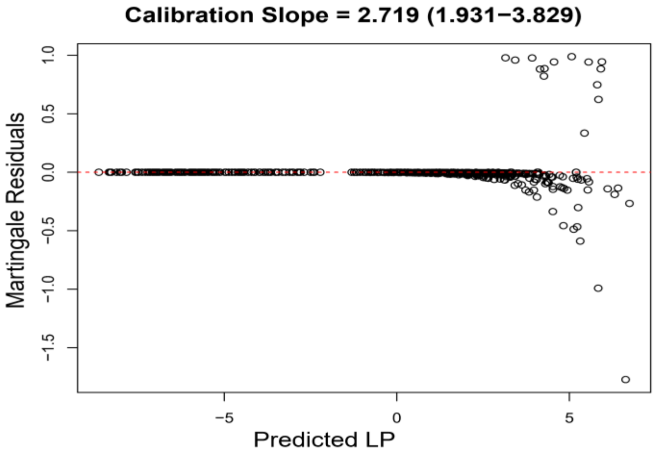

(E)

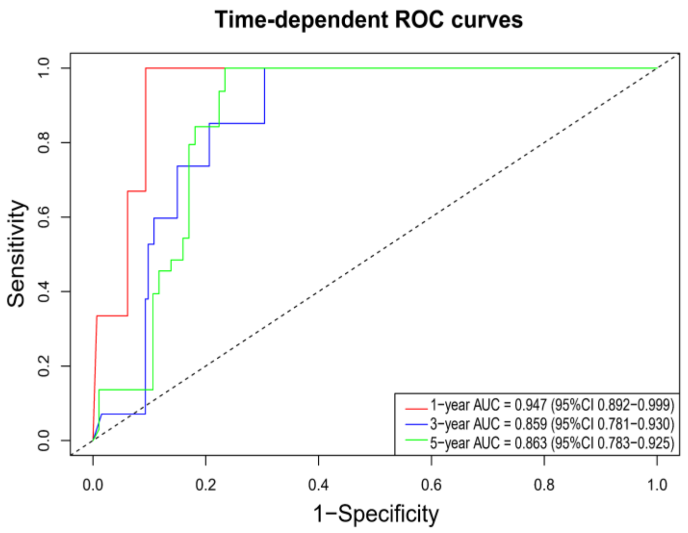

(F)

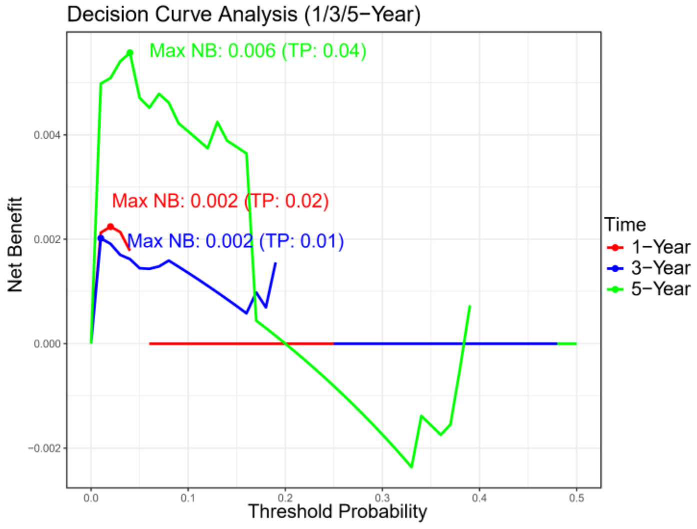

Supplementary Figure S27. Comprehensive evaluation of the prognostic models for THCA.

The models' construction followed the procedure: Univariate Cox (*P* <0.2) + Multivariate Cox. The resulting risk score formula of genomic model is: Risk Score = 0.6769 * gene_IMPDH1. The resulting risk score formula of integrated clinico-genomic model is: Risk Score = 0.4956 * risk_score_gene + 0.1082 * age + -0.0223 * stage + 0.376 * race asian + 18.9633 * race black or african american + 0.1647 * race unknow + 0 * race white + -0.0445 * gender Male.

(A) Nomogram for Survival Prediction: A graphical representation of the prognostic model, allowing for the visual estimation of 1-, 3-, and 5-year overall survival probabilities based on individual gene expression levels (and clinical features, if clinico-genomic model). The genomic model's internal validation C-index was 0.600 (95%CI: 0.460-0.794), integrated clinico-genomic model's internal validation C-index was 0.945 (95%CI: 0.910-0.981).

(B) Kaplan-Meier Survival Curves: Survival analysis demonstrated a statistically significant differences in overall survival between the high- and low-risk groups in the integrated clinico-genomic model (HR = 2.07, 95% CI: 1.46–2.92, log-rank p <0.001), whereas no significant difference was observed for the genomic model (HR = 1.38, 95% CI: 0.51–3.70, log-rank p = 0.525). The table below shows the number of patients at risk over time for each group.

(C) 1-, 3-, and 5-Year Calibration Curves: Assesses the agreement between the predicted and observed overall survival probabilities at 1, 3, and 5 years, respectively. The diagonal dashed line represents perfect calibration.

(D) Calibration Slope Plot: Further evaluates the model's calibration, showing the relationship between predicted linear predictor and Martingale residuals. The calibration slope of genomic model was 2.719 (95%CI: 0.715-10.341). The calibration slope of integrated clinico-genomic model was 0.945 (95%CI: 0.910-0.981).

(E) Time-Dependent Receiver Operating Characteristic (ROC) Curves: Illustrates the discriminatory ability of the model over time. The area under the curve (AUC) values of genomic model were 0.716 (0.144-1.000), 0.546 (0.371-0.749), and 0.480 (0.311-

0.655) for 1-, 3-, and 5-year survival, respectively. The AUC values of integrated clinico-genomic model were 0.947 (0.892-0.999), 0.859 (0.781-0.930), and 0.863 (0.783-0.925) for 1-, 3-, and 5-year survival, respectively.

(F) Decision Curve Analysis (DCA): Evaluates the clinical utility of the prognostic model by quantifying the net benefit across a range of threshold probabilities. The maximum net benefit (Max NB) and corresponding threshold probability (TP) for 1-, 3-, and 5-year survival in genomic model were: 1-year Max NB = 0.003 (TP: 0.01); 3-year Max NB = 0.021 (TP: 0.01); 5-year Max NB = 0.053 (TP: 0.02). The Max NB and TP for 1-, 3-, and 5-year survival in integrated clinico-genomic model were: 1-year Max NB =0.002 (TP: 0.01); 3-year Max NB = 0.002 (TP: 0.02); 5-year Max NB = 0.006 (TP: 0.04).

***Genomic prognostic model for UCEC*:**

Univariate Cox (P < 0.2) + LASSO with 10-fold CV (lambda.min) + Stepwise Cox (AIC)

Risk Score = 0.3327 * gene_ADA + 0.1929 * gene_UCK2

(A)

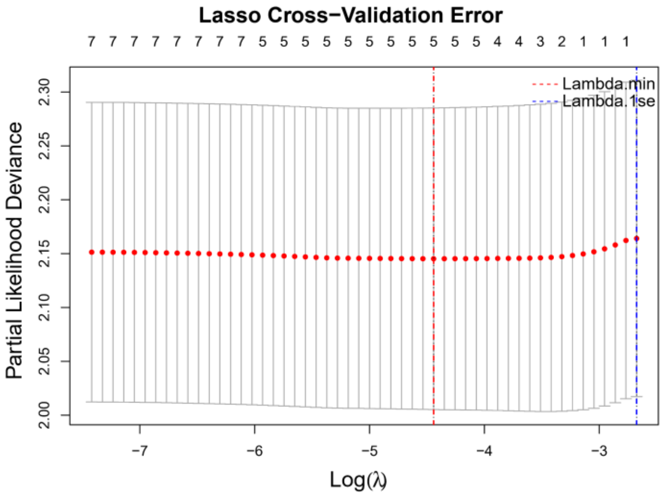

(B)

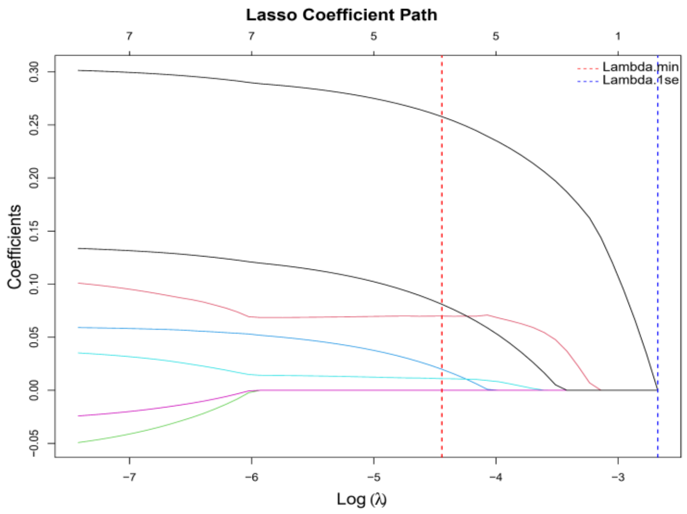

(C)

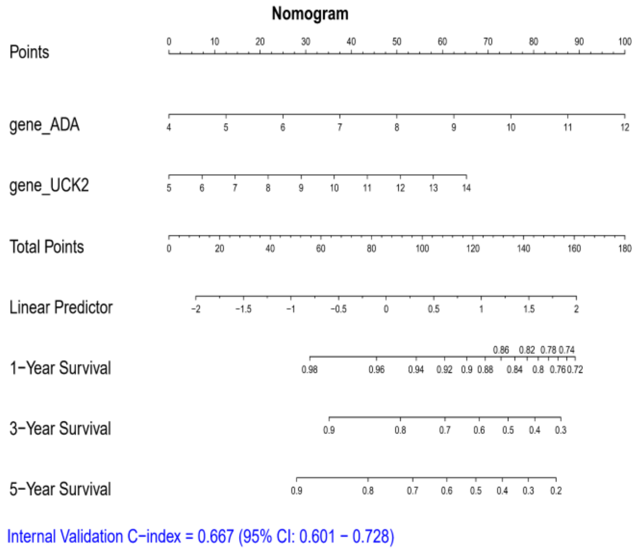

(D)

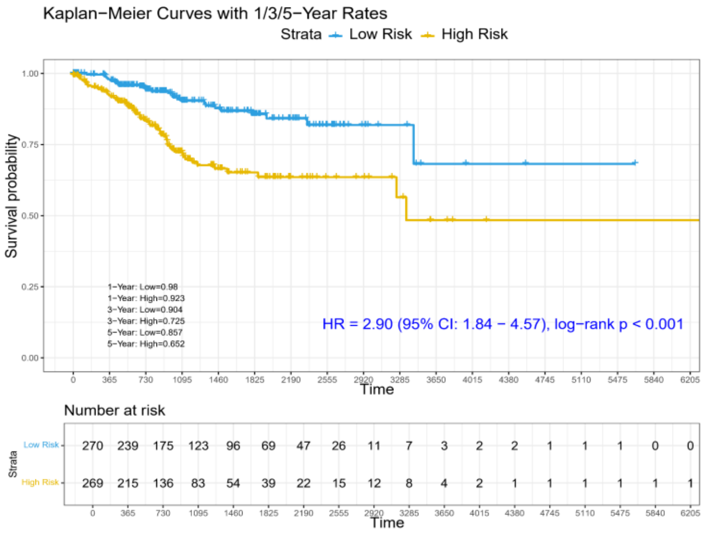

(E)

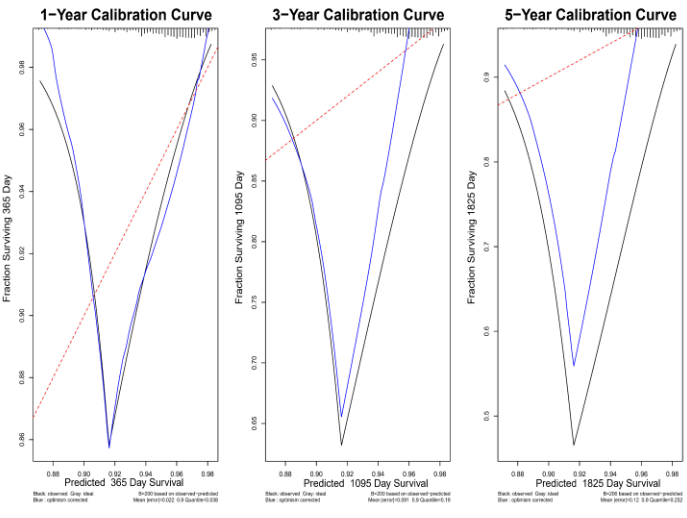

(F)

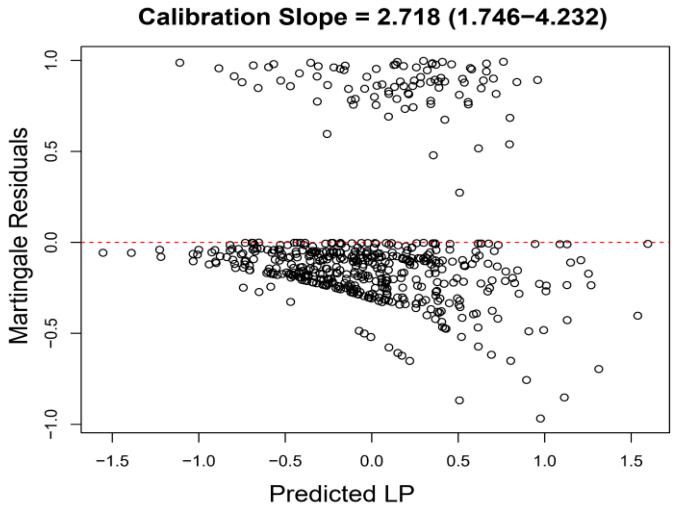

(G)

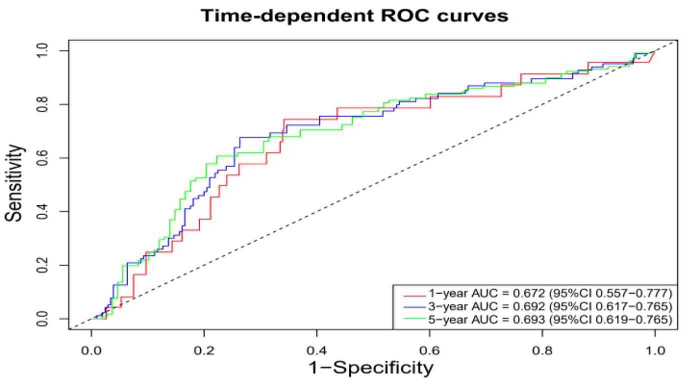

(H)

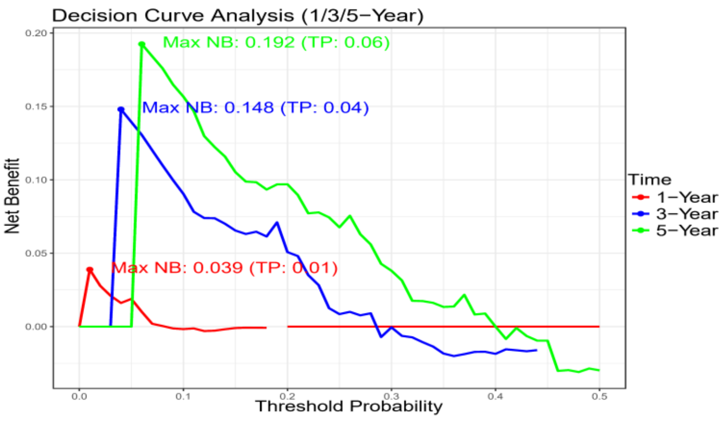

***Clinico-genomic prognostic model for UCEC*:**

Univariate Cox (P < 0.2) + LASSO with 10-fold CV (lambda.min) + Stepwise Cox (AIC)

Risk Score = 0.5492 * risk_score_gene + 0.0378 * age + 1.2954 * stage + -1.2188 * race asian + -0.8917 * race black or african american + -0.2097 * race native hawaiian or other pacific islander + -1.8262 * race unknow + -0.7062 * race white

(A)

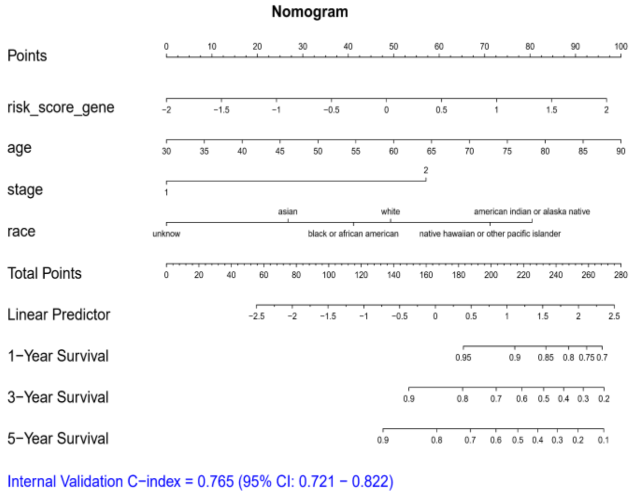

(B)

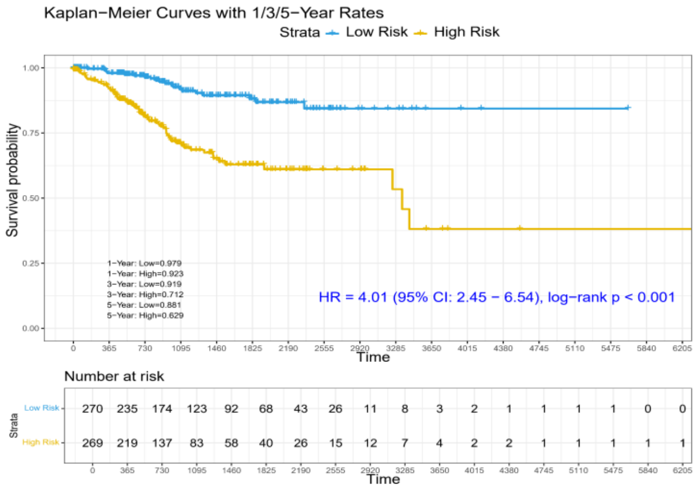

(C)

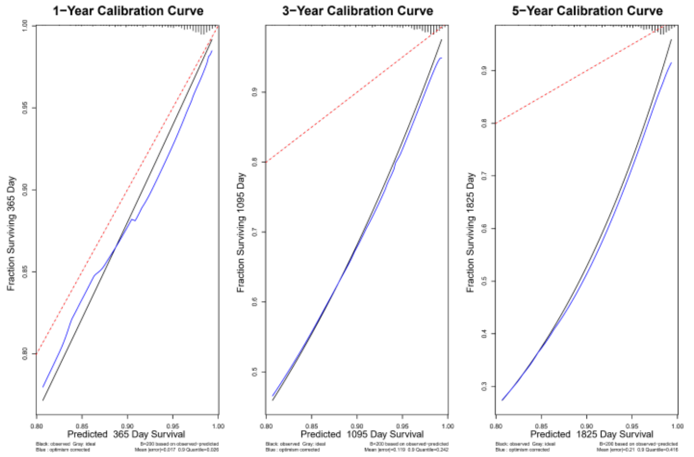

(D)

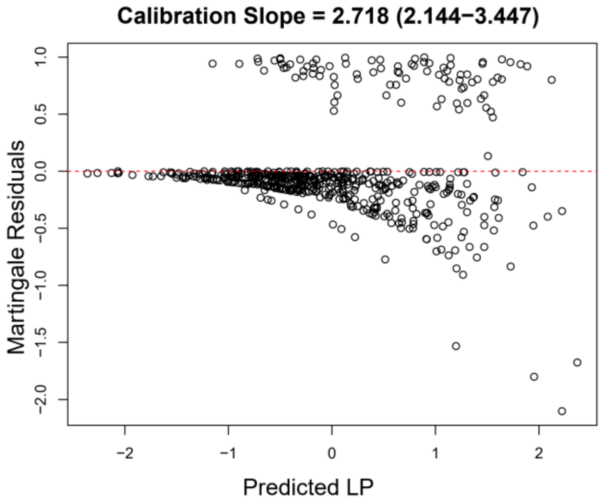

(E)

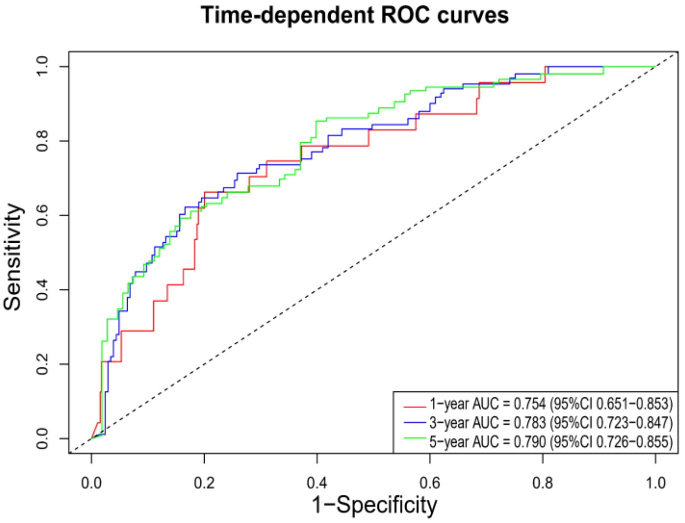

(F)

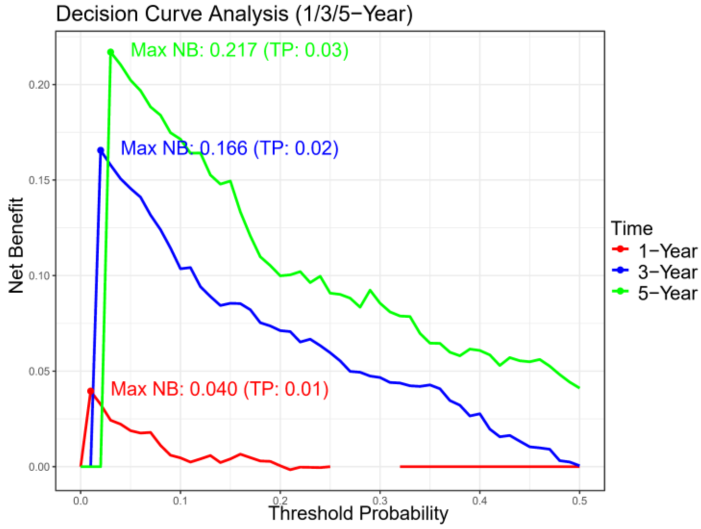

Supplementary Figure S28. Comprehensive evaluation of the prognostic models for UCEC. The models' construction followed the procedure: Univariate Cox (*P* <0.2) + LASSO with 10-fold CV (lambda.min) + Stepwise Cox (AIC). The resulting risk score formula of genomic model is: Risk Score = 0.3327 * gene_ADA + 0.1929 * gene_UCK2. The resulting risk score formula of integrated clinico-genomic model is: Risk Score = 0.5492 * risk_score_gene + 0.0378 * age + 1.2954 * stage + -1.2188 * race asian + -0.8917 * race black or african american + -0.2097 * race native hawaiian or other pacific islander + -1.8262 * race unknow + -0.7062 * race white.

(A) Lasso Cross-Validation Error Plot: Depicts the cross-validation error distribution across different lambda (λ) values, indicating the optimal lambda values (lambda.min and lambda.1se) for penalty parameter selection in LASSO regression. The best-performing model for this tumor type used lambda.min, with a λ value of 0.012.

(B) Lasso Coefficient Path: Illustrates how the coefficients of the selected genes change as the lambda (λ) regularization parameter varies, demonstrating the variable selection process.

(C) in genomic model figures and (A) in integrated clinico-genomic model figures: Nomogram for Survival Prediction: A graphical representation of the prognostic model, allowing for the visual estimation of 1-, 3-, and 5-year overall survival probabilities based on individual gene expression levels (and clinical features, if clinico-genomic model). The genomic model's internal validation C-index was 0.667 (95%CI: 0.601-0.728), integrated clinico-genomic model's internal validation C-index was 0.765 (95%CI: 0.721-0.822).

(D) in genomic model figures and (B) in integrated clinico-genomic model figures: Kaplan-Meier Survival Curves: Survival analysis demonstrated significant differences in overall survival between the high- and low-risk groups in both the genomic model (HR = 2.90 95% CI: 1.84–4.57, log-rank p <0.001) and the integrated clinico-genomic model (HR = 4.01, 95% CI: 2.45–6.54, log-rank p <0.001). The table below shows the number of patients at risk over time for each group.

(E) in genomic model figures and (C) in integrated clinico-genomic model figures: 1-, 3-, and 5-Year Calibration Curves: Assesses the agreement between the predicted

and observed overall survival probabilities at 1, 3, and 5 years, respectively. The diagonal dashed line represents perfect calibration.

(F) in genomic model figures and (D) in integrated clinico-genomic model figures: Calibration Slope Plot: Further evaluates the model's calibration, showing the relationship between predicted linear predictor and Martingale residuals. The calibration slope of genomic model was 2.718 (95%CI: 1.746-4.232). The calibration slope of integrated clinico-genomic model was 2.718 (95%CI: 2.144-3.447).

(G) in genomic model figures and (E) in integrated clinico-genomic model figures: Time-Dependent Receiver Operating Characteristic (ROC) Curves: Illustrates the discriminatory ability of the model over time. The area under the curve (AUC) values of genomic model were 0.672 (0.557-0.777), 0.692 (0.617-0.765), and 0.693 (0.619-0.765) for 1-, 3-, and 5-year survival, respectively. The AUC values of integrated clinico-genomic model were 0.754 (0.651-0.853), 0.783 (0.723-0.847), and 0.790 (0.726-0.855) for 1-, 3-, and 5-year survival, respectively.

(H) in genomic model figures and (F) in integrated clinico-genomic model figures: Decision Curve Analysis (DCA): Evaluates the clinical utility of the prognostic model by quantifying the net benefit across a range of threshold probabilities. The maximum net benefit (Max NB) and corresponding threshold probability (TP) for 1-, 3-, and 5-year survival in genomic model were: 1-year Max NB =0.039 (TP: 0.01); 3-year Max NB = 0.148 (TP: 0.04); 5-year Max NB = 0.192 (TP: 0.06). The Max NB and TP for 1-, 3-, and 5-year survival in integrated clinico-genomic model were: 1-year Max NB = 0.040 (TP: 0.01); 3-year Max NB = 0.166 (TP: 0.02); 5-year Max NB = 0.217 (TP: 0.03).

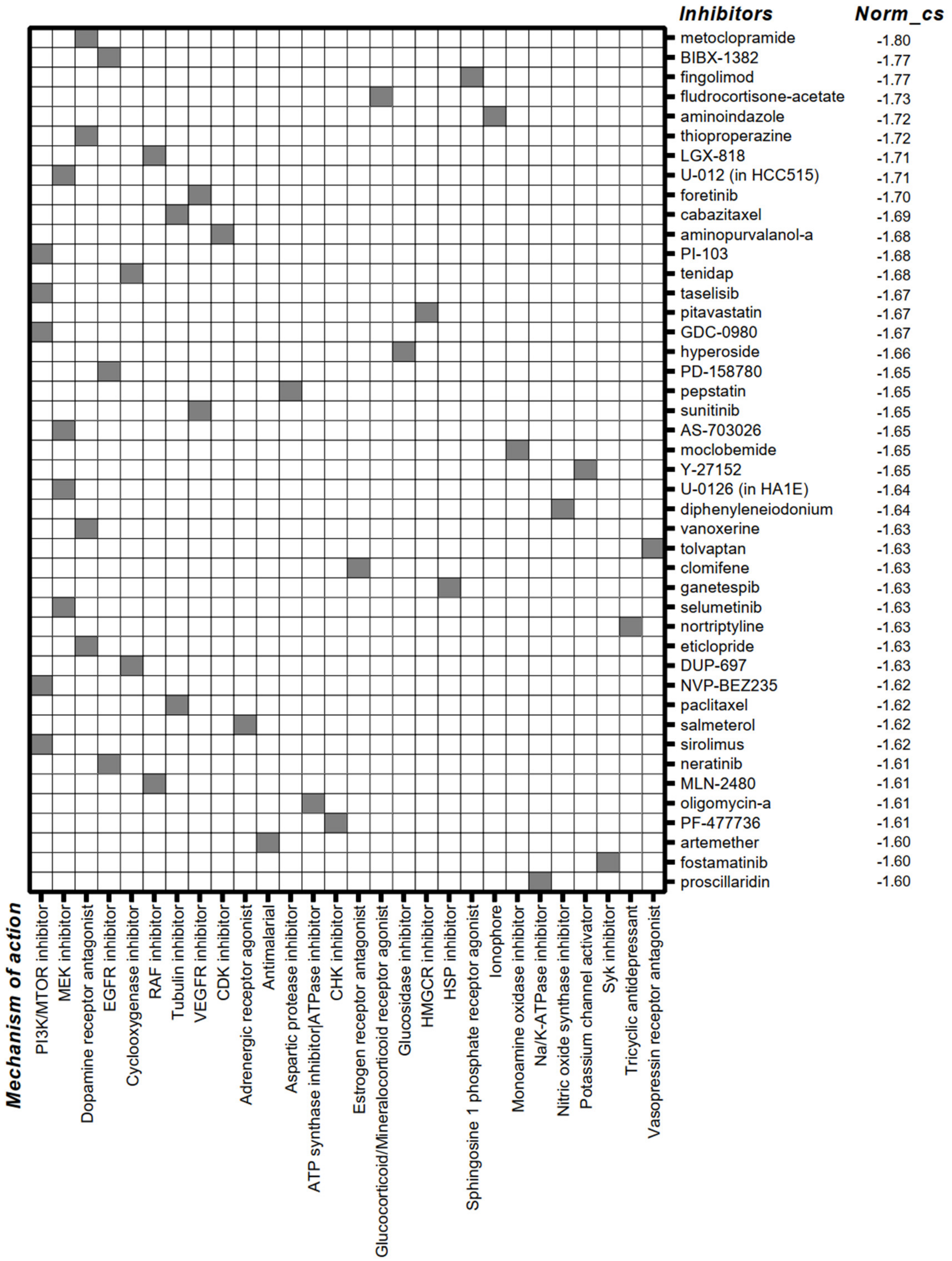

Supplementary Figure S29. Mechanisms of potential molecular compounds targeting a pan-cancer dysregulated purine metabolism pathway. Drugging the pan-cancer dysregulated genes in the purine synthesis pathway and further interpreting the roles in tumor immunity. Heatmap represents each compound (rows) from the Connectivity Map (CMap) database that shares a mode of action (MoA) (columns), sorted by the reduction of normalized connectivity score (Norm_cs).
