# Supplementary material for: Purine metabolism: a pan-cancer metabolic dysregulation across circulation and tissues
Source: Mol Cancer. 2025 Oct 14;24:255. doi: 10.1186/s12943-025-02482-9 (PMC12522462; doi:10.1186/s12943-025-02482-9)
Supplement: Supplementary file 2 — Supplementary Material 2. [file 12943_2025_2482_MOESM2_ESM.docx]

**Supplementary** Table S1. **The identified compounds in human plasma.**

| **Identified compounds** | **Derivatives** | **Rt (min)** | **Quant Mass (m/z)** | **Basis for identification** |
| --- | --- | --- | --- | --- |
| Caproic acid | 1TMS | 4.615 | 173 | Wiley, NIST, CPU library |
| Pyruvic acid | MEOX, 1TMS | 4.705 | 174 | Wiley, NIST, CPU library, match with authentic standard |
| Lactic acid | 2TMS | 4.85 | 191 | Wiley, NIST, CPU library, match with authentic standard |
| Glycolic acid | 2TMS | 5.01 | 177 | CPU library, match with authentic standard |
| Alanine | 2TMS | 5.32 | 116 | Wiley, NIST, CPU library, match with authentic standard |
| Oxalic acid | 2TMS | 5.73 | 220 | Wiley, NIST, CPU library, match with authentic standard |
| 3-Hydroxybutyric acid | 2TMS | 5.915 | 191 | Wiley, NIST, CPU library, match with authentic standard |
| 3-Hydroxyisobutyric acid | 2TMS | 5.93 | 177 | Wiley, NIST, CPU library |
| Valine | 2TMS | 6.455 | 218 | Wiley, NIST, CPU library, match with authentic standard |
| Urea | 2TMS | 6.625 | 189 | Wiley, NIST, CPU library, match with authentic standard |
| Leucine | 2TMS | 6.94 | 158 | Wiley, NIST, CPU library, match with authentic standard |
| Phosphate | 3TMS | 6.975 | 299 | Wiley, NIST, CPU library, match with authentic standard |
| Isoleucine | 2TMS | 7.125 | 158 | Wiley, NIST, CPU library, match with authentic standard |
| Proline | 2TMS | 7.155 | 142 | Wiley, NIST, CPU library, match with authentic standard |
| Glycine | 3TMS | 7.235 | 174 | Wiley, NIST, CPU library, match with authentic standard |
| Glyceric acid | 3TMS | 7.425 | 292 | Wiley, NIST, CPU library, match with authentic standard |
| Fumaric acid | 2TMS | 7.485 | 245 | Wiley, NIST, CPU library, match with authentic standard |
| Pelargonic acid | 1TMS | 7.58 | 215 | Wiley, NIST, CPU library, match with authentic standard |
| Serine | 3TMS | 7.65 | 204 | Wiley, NIST, CPU library, match with authentic standard |
| Threonine | 3TMS | 7.855 | 218 | Wiley, NIST, CPU library, match with authentic standard |
| Cystathionine | 2TMS | 7.91 | 221 | CPU library, match with authentic standard |
| Homocysteine | 2TMS | 8.01 | 234 | CPU library, match with authentic standard |
| Methylcysteine | 2TMS | 8.095 | 248 | CPU library, match with authentic standard |
| Aminomalonic acid | 3TMS | 8.435 | 218 | Wiley, NIST, CPU library, match with authentic standard |
| Malic acid | 3TMS | 8.54 | 233 | Wiley, NIST, CPU library, match with authentic standard |
| Methionine | 3TMS | 8.75 | 176 | Wiley, NIST, CPU library, match with authentic standard |
| Pyroglutamic acid | 2TMS | 8.78 | 156 | Wiley, NIST, CPU library, match with authentic standard |
| Aspartic acid | 3TMS | 8.805 | 232 | Wiley, NIST, CPU library, match with authentic standard |
| Cysteine | 3TMS | 8.99 | 220 | Wiley, NIST, CPU library, match with authentic standard |
| Creatinine | 3TMS | 9.025 | 115 | Wiley, NIST, CPU library, match with authentic standard |
| Glutamic acid | 3TMS | 9.36 | 246 | Wiley, NIST, CPU library, match with authentic standard |
| Phenylalanine | 2TMS | 9.44 | 218 | Wiley, NIST, CPU library, match with authentic standard |
| Lauric acid | 1TMS | 9.5 | 257 | Wiley, NIST, CPU library |
| Asparagine | 3TMS | 9.685 | 231 | Wiley, NIST, CPU library, match with authentic standard |
| Methyl myristate (external standard) | / | 9.9 | 199 | match with authentic standard |
| Glutamine | 3TMS | 10.26 | 156 | Wiley, NIST, CPU library, match with authentic standard |
| Hypoxanthine | 2TMS | 10.455 | 265 | Wiley, NIST, CPU library, match with authentic standard |
| Ornithine | 4TMS | 10.525 | 142 | Wiley, NIST, CPU library, match with authentic standard |
| Citric acid | 4TMS | 10.54 | 273 | Wiley, NIST, CPU library, match with authentic standard |
| Myristic acid-1,2-^13^C_2_ (internal standard) | 1TMS | 10.6 | 287 | match with authentic standard |
| Fucose | 4TMS | 10.74 | 217 | Wiley, CPU library |
| Fructose | 2MEOX, 5TMS | 10.875 | 307 | Wiley, NIST, CPU library, match with authentic standard |
| Glucose | MEOX, 5TMS | 11.025 | 319 | Wiley, NIST, CPU library, match with authentic standard |
| Lysine | 4TMS | 11.06 | 317 | Wiley, NIST, CPU library, match with authentic standard |
| Histidine | TMS | 11.08 | 254 | Wiley, NIST, CPU library, match with authentic standard |
| Tyrosine | 3TMS | 11.16 | 218 | Wiley, NIST, CPU library, match with authentic standard |
| Palmitic acid | 1TMS | 11.595 | 313 | Wiley, NIST, CPU library, match with authentic standard |
| Uric acid | 4TMS | 11.975 | 441 | Wiley, NIST, CPU library, match with authentic standard |
| myo-Inositol | 6TMS | 11.99 | 318 | Wiley, NIST, CPU library, match with authentic standard |
| Linoleic acid | 1TMS | 12.39 | 337 | Wiley, NIST, CPU library, match with authentic standard |
| Oleic acid | 1TMS | 12.405 | 339 | Wiley, NIST, CPU library |
| Stearic acid | 1TMS | 12.51 | 341 | Wiley, NIST, CPU library |
| Tryptophan | 3TMS | 12.54 | 202 | Wiley, NIST, CPU library, match with authentic standard |
| Cystine | 4TMS | 12.85 | 218 | Wiley, NIST, CPU library, match with authentic standard |
| Arachidonic acid | 1TMS | 13.09 | 117 | Wiley, NIST, CPU library, match with authentic standard |
| Glycerol 1-hexadecanoate | 2TMS | 13.965 | 371 | Wiley, NIST, CPU library |
| alpha-Tocopherol | 1TMS | 17.11 | 502 | Wiley, NIST, CPU library, match with authentic standard |
| Cholesterol | 1TMS | 17.33 | 458 | Wiley, NIST, CPU library, match with authentic standard |

Altogether, 58 compounds were identified as trimethylsilylated (TMS) and methoximated (MEOX) derivatives. Compound identities were confirmed by comparing mass spectra and retention times against authentic standards and three reference databases: the National Institute of Standards and Technology (NIST), Wiley (Wiley-VCH Verlag GmbH), and an in-house library from the State Key Laboratory of Natural Medicines, China Pharmaceutical University.

**Supplementary Table S2. The relative standard deviation (RSD) distribution of the quality control (QC) samples.**

| **Identified compounds** | **MEAN** | **SD** | **RSD （%）** |
| --- | --- | --- | --- |
| Fucose | 29139.72 | 871647.84 | 3.34% |
| 3-Hydroxybutyric acid | 9553.77 | 268196.13 | 3.56% |
| Cholesterol | 64509.07 | 1438228.50 | 4.49% |
| Threonine | 22000.66 | 483632.52 | 4.55% |
| Glutamic acid | 21074.01 | 437270.39 | 4.82% |
| Tyrosine | 50640.60 | 996174.22 | 5.08% |
| Oleic acid | 27272.52 | 519118.97 | 5.25% |
| Linoleic acid | 14828.71 | 268764.90 | 5.52% |
| Serine | 47525.93 | 841325.05 | 5.65% |
| myo-Inositol | 7552.69 | 133518.73 | 5.66% |
| Methylcysteine | 2107.01 | 36304.08 | 5.80% |
| Tryptophan | 43598.99 | 750072.58 | 5.81% |
| Glycine | 103553.58 | 1772416.91 | 5.84% |
| Lactic acid | 307355.98 | 5202249.43 | 5.91% |
| Palmitic acid | 60105.46 | 965229.74 | 6.23% |
| Phenylalanine | 26748.38 | 414691.32 | 6.45% |
| Hypoxanthine | 2229.22 | 34429.96 | 6.47% |
| 3-Hydroxyisobutyric acid | 1318.66 | 20039.04 | 6.58% |
| Leucine | 114744.47 | 1730173.26 | 6.63% |
| Alanine | 290541.33 | 4364305.54 | 6.66% |
| Glutamine | 131887.00 | 1925836.75 | 6.85% |
| Isoleucine | 58296.37 | 799267.83 | 7.29% |
| Fumaric acid | 3776.91 | 50926.40 | 7.42% |
| Phosphate | 185799.98 | 2326145.98 | 7.99% |
| Glucose | 451350.64 | 5405191.67 | 8.35% |
| Stearic acid | 37930.20 | 453315.85 | 8.37% |
| Methionine | 10359.28 | 122092.66 | 8.48% |
| Asparagine | 5956.70 | 70016.88 | 8.51% |
| Glycolic acid | 1795.59 | 20891.73 | 8.59% |
| Pyroglutamic acid | 236574.87 | 2716088.74 | 8.71% |
| Citric acid | 81794.05 | 882071.59 | 9.27% |
| Cysteine | 11208.30 | 117562.82 | 9.53% |
| Caproic acid | 8984.79 | 93194.04 | 9.64% |
| Malic acid | 1635.81 | 15614.89 | 10.48% |
| alpha-Tocopherol | 14820.74 | 122635.63 | 12.09% |
| Lauric acid | 2376.23 | 19633.63 | 12.10% |
| Pyruvic acid | 27124.45 | 216695.20 | 12.52% |
| Proline | 255068.87 | 1915669.75 | 13.31% |
| Aspartic acid | 92405.57 | 675414.44 | 13.68% |
| Glyceric acid | 3529.62 | 25043.28 | 14.09% |
| Histidine | 20023.42 | 135578.11 | 14.77% |
| Ornithine | 138719.20 | 825469.71 | 16.80% |
| Arachidonic acid | 19503.41 | 115937.65 | 16.82% |
| Aminomalonic acid | 40863.36 | 241388.47 | 16.93% |
| Lysine | 51599.32 | 279946.82 | 18.43% |
| Pelargonic acid | 4338.75 | 20938.69 | 20.72% |
| Cystathionine | 2468.34 | 11395.48 | 21.66% |
| Fructose | 44956.58 | 196987.05 | 22.82% |
| Oxalic acid | 349770.23 | 1525049.01 | 22.94% |
| Glycerol 1-hexadecanoate | 8175.42 | 32773.65 | 24.95% |
| Creatinine | 6566.00 | 21669.25 | 30.30% |
| Homocysteine | 10548.39 | 34672.69 | 30.42% |
| Cystine | 15325.71 | 47995.70 | 31.93% |

**Supplementary Table S3. Differential metabolites between nasopharyngeal carcinoma (NPC) patients and healthy controls (HC) in Center 1.**

|  | **NPC (n=17)** | **HC (n=15)** | **FDR** | **FC** |
| --- | --- | --- | --- | --- |
| Male, n (%) | 13 (76.47) | 11 (73.33) | 0.831 |  |
| Age (years) | 58±18 | 58±15 | 0.924 |  |
| Hypoxanthine | 33870±10435 | 7883±9290 | 6.96E-05 | 4.30 |
| Cysteine | 115783±42237 | 161904±33212 | 3.69E-02 | 0.72 |
| Pyruvic acid | 624129±136932 | 792438±196393 | 4.25E-02 | 0.79 |

**Supplementary Table S4. Differential metabolites between laryngeal squamous cell carcinoma (LSCC) patients and healthy controls (HC) in Center 1.**

|  | **LSCC (n=18)** | **HC (n=18)** | **FDR** | **FC** |
| --- | --- | --- | --- | --- |
| Male, n (%) | 18 (100.00) | 18 (100.00) | 1 |  |
| Age (years) | 69±10 | 68±10 | 0.893 |  |
| Hypoxanthine | 28304±12805 | 6628±8928 | 1.97E-04 | 4.27 |
| Aminomalonic acid | 271297±94317 | 173469±49350 | 5.68E-03 | 1.56 |
| Oxalic acid | 2243846±794000 | 1510988±598002 | 1.81E-02 | 1.49 |
| Glycolic acid | 21565±5323 | 16327±3662 | 2.14E-02 | 1.32 |
| Glycine | 2053054±429948 | 1558727±263078 | 1.22E-03 | 1.32 |
| Caproic acid | 145718±54948 | 117334±57725 | 2.14E-02 | 1.24 |
| Palmitic acid | 853485±173047 | 1044737±220756 | 4.74E-02 | 0.82 |
| Pyruvic acid | 600266±121507 | 769440±143713 | 1.81E-02 | 0.78 |
| Linoleic acid | 211089±86846 | 292213±83538 | 1.44E-02 | 0.72 |
| Cysteine | 113789±26060 | 173750±39081 | 1.97E-04 | 0.65 |

**Supplementary Table S5. Differential metabolites between Thyroid carcinoma (THCA) patients and healthy controls (HC) in Center 1.**

|  | **THCA (n=29)** | **HC (n=29)** | **FDR** | **FC** |
| --- | --- | --- | --- | --- |
| Male, n (%) | 16 (55.17) | 13 (44.83) | 0.410 |  |
| Age (years) | 47±13 | 51±9 | 0.626 |  |
| Hypoxanthine | 28578±10192 | 4770±5697 | 3.03E-11 | 5.99 |
| Creatinine | 50655±21542 | 32198±17609 | 7.06E-03 | 1.57 |
| Glycolic acid | 23955±4118 | 17177±2975 | 3.50E-08 | 1.39 |
| Glucose | 7928225±2187624 | 5953372±1324938 | 1.71E-04 | 1.33 |
| Pyruvic acid | 621189±125063 | 802368±156930 | 1.71E-04 | 0.77 |
| Malic acid | 11474±3188 | 15007±3212 | 6.95E-04 | 0.76 |
| Cysteine | 113120±32359 | 168091±24968 | 3.41E-06 | 0.67 |

**Supplementary Table S6. Differential metabolites between lung adenocarcinoma (LUAD) patients and healthy controls (HC) in Center 1.**

|  | **LUAD (n=380)** | **HC (n=352)** | **FDR** | **FC** |
| --- | --- | --- | --- | --- |
| Male, n (%) | 149 (39.21) | 175 (49.72) | 0.000 |  |
| Age (years) | 60±12 | 58±12 | 0.006 |  |
| Hypoxanthine | 29237±15331 | 5752±6868 | 6.11E-91 | 5.08 |
| Aspartic acid | 584368±362135 | 241844±178142 | 2.78E-50 | 2.42 |
| Homocysteine | 53782±56241 | 33035±47417 | 7.52E-17 | 1.63 |
| Cystine | 69981±32741 | 49348±30573 | 2.83E-20 | 1.42 |
| Glycolic acid | 22808±10539 | 16864±4652 | 2.33E-26 | 1.35 |
| Fucose | 950265±452075 | 760376±388470 | 1.13E-09 | 1.25 |
| Cysteine | 133105±34926 | 167652±35642 | 8.65E-31 | 0.79 |
| Linoleic acid | 230797±100823 | 292131±79218 | 1.75E-27 | 0.79 |
| Pelargonic acid | 12518±7949 | 16356±6226 | 3.68E-10 | 0.77 |
| Pyruvic acid | 613878±149941 | 804463±180969 | 1.32E-45 | 0.76 |

**Supplementary Table S7. Differential metabolites between lung squamous carcinoma (LUSC) patients and healthy controls (HC) in Center 1.**

|  | **LUSC (n=53)** | **HC (n=53)** | **FDR** | **FC** |
| --- | --- | --- | --- | --- |
| Male, n (%) | 53 (100.00) | 53 (100.00) | 1 |  |
| Age (years) | 67±9 | 67±9 | 0.990 |  |
| Hypoxanthine | 36348±19111 | 6330±8516 | 1.13E-13 | 5.74 |
| Homocysteine | 52128±49006 | 29037±36706 | 1.62E-03 | 1.80 |
| Creatinine | 46929±33241 | 26942±17782 | 1.95E-03 | 1.74 |
| Fucose | 1129629±638273 | 723828±396691 | 2.26E-03 | 1.56 |
| Aspartic acid | 481289±310362 | 322582±192591 | 1.31E-02 | 1.49 |
| Glycolic acid | 23729±7200 | 15922±4033 | 2.76E-09 | 1.49 |
| Oxalic acid | 2332599±919613 | 1760225±526405 | 4.01E-03 | 1.33 |
| Caproic acid | 181730±240392 | 139520±148114 | 9.03E-03 | 1.30 |
| Aminomalonic acid | 244661±104967 | 190960±56053 | 4.01E-03 | 1.28 |
| Pelargonic acid | 16633±8289 | 13597±5996 | 1.29E-02 | 1.22 |
| Histidine | 130806±48086 | 169193±38000 | 3.74E-05 | 0.77 |
| Pyruvic acid | 582366±134918 | 757172±149823 | 9.51E-07 | 0.77 |
| Citric acid | 672002±164372 | 908299±171013 | 5.04E-08 | 0.74 |
| Glycerol 1-hexadecanoate | 21671±12065 | 29826±11924 | 1.24E-03 | 0.73 |
| Linoleic acid | 201449±68725 | 284715±80167 | 5.38E-06 | 0.71 |
| Cysteine | 113581±38823 | 165616±40658 | 1.43E-07 | 0.69 |

**Supplementary Table S8. Differential metabolites between small cell lung carcinoma (SCLC) patients and healthy controls (HC) in Center 1.**

|  | **SCLC (n=25)** | **HC (n=25)** | **FDR** | **FC** |
| --- | --- | --- | --- | --- |
| Male, n (%) | 22 (88.00) | 21 (84.00) | 0.832 |  |
| Age (years) | 68±10 | 70±7 | 0.853 |  |
| Hypoxanthine | 33462±17960 | 4727±8368 | 2.73E-06 | 7.08 |
| Homocysteine | 74499±57155 | 33717±32497 | 2.98E-02 | 2.21 |
| Aspartic acid | 548760±354845 | 319978±158168 | 3.25E-02 | 1.71 |
| Creatinine | 36148±15900 | 23479±10948 | 2.98E-02 | 1.54 |
| Glycolic acid | 21937±7866 | 15701±3491 | 3.47E-03 | 1.40 |
| Glutamic acid | 588175±221955 | 442600±155095 | 4.76E-02 | 1.33 |
| Linoleic acid | 229655±82154 | 302110±88739 | 2.98E-02 | 0.76 |
| Pyruvic acid | 573716±143535 | 773092±178910 | 7.57E-04 | 0.74 |
| alpha-Tocopherol | 182158±40310 | 262709±86148 | 2.85E-03 | 0.69 |
| Cysteine | 118944±29340 | 179048±34389 | 2.73E-06 | 0.66 |

**Supplementary Table S9. Differential metabolites between breast carcinoma (BRCA) patients and healthy controls (HC) in Center 1.**

|  | **BRCA (n=194)** | **HC (n=177)** | **FDR** | **FC** |
| --- | --- | --- | --- | --- |
| Female, n (%) | 194 (100.00) | 177 (100.00) | 1 |  |
| Age (years) | 56±13 | 55±10 | 0.603 |  |
| Hypoxanthine | 31116±13347 | 5970±6848 | 1.09E-50 | 5.21 |
| Aspartic acid | 674214±490220 | 219242±172499 | 2.13E-21 | 3.08 |
| 3-Hydroxybutyric acid | 292324±774348 | 142332±145521 | 2.06E-03 | 2.05 |
| Glycolic acid | 24862±10657 | 17039±4693 | 2.12E-21 | 1.46 |
| Homocysteine | 43451±53206 | 31006±49620 | 2.01E-07 | 1.40 |
| Oleic acid | 553058±306153 | 443127±232011 | 1.58E-03 | 1.25 |
| Cystine | 52758±31387 | 43224±25051 | 3.88E-03 | 1.22 |
| Lactic acid | 4705609±1327070 | 6141347±1177969 | 1.98E-23 | 0.77 |
| Pyruvic acid | 615487±149559 | 828592±174391 | 1.83E-26 | 0.74 |
| Cysteine | 121136±37949 | 165379±33997 | 7.26E-24 | 0.73 |

**Supplementary Table S10. Differential metabolites between esophageal carcinoma (ESCA) patients and healthy controls (HC) in Center 1.**

|  | **ESCA (n=119)** | **HC (n=119)** | **FDR** | **FC** |
| --- | --- | --- | --- | --- |
| Male, n (%) | 89 (74.79) | 94 (78.99) | 0.504 |  |
| Age (years) | 68±9 | 68±10 | 0.388 |  |
| Hypoxanthine | 31963±11091 | 5277±6677 | 1.37E-34 | 6.06 |
| 3-Hydroxybutyric acid | 629635±968339 | 174007±209959 | 2.04E-06 | 3.62 |
| Aspartic acid | 476462±368419 | 270933±190854 | 7.30E-05 | 1.76 |
| Creatinine | 44312±21220 | 30233±18351 | 5.84E-07 | 1.47 |
| Aminomalonic acid | 279326±110239 | 198386±57367 | 1.23E-10 | 1.41 |
| Homocysteine | 45055±39683 | 32822±37640 | 4.65E-04 | 1.37 |
| Glycolic acid | 22475±7441 | 16379±4446 | 1.45E-13 | 1.37 |
| Oxalic acid | 2388703±861702 | 1863445±621452 | 2.94E-06 | 1.28 |
| Glycine | 2126465±580082 | 1722106±430832 | 2.41E-09 | 1.23 |
| Fucose | 898217±474382 | 733378±398244 | 6.17E-03 | 1.22 |
| Pyruvic acid | 590828±126939 | 769422±187006 | 7.31E-14 | 0.77 |
| alpha-Tocopherol | 181867±57418 | 237080±83846 | 4.22E-08 | 0.77 |
| Glycerol 1-hexadecanoate | 23647±12719 | 32166±11458 | 8.88E-07 | 0.74 |
| Cysteine | 113001±31711 | 173152±40039 | 1.26E-22 | 0.65 |

**Supplementary Table S11. Differential metabolites between adenocarcinoma of esophagogastric junction (AEG) patients and healthy controls (HC) in Center 1.**

|  | **AEG (n=81)** | **HC (n=81)** | **FDR** | **FC** |
| --- | --- | --- | --- | --- |
| Male, n (%) | 62 (76.54) | 62 (76.54) | 1 |  |
| Age (years) | 71±7 | 71±9 | 0.813 |  |
| Hypoxanthine | 28757±13192 | 5825±7481 | 6.71E-19 | 4.94 |
| 3-Hydroxybutyric acid | 403801±597087 | 164078±164925 | 6.11E-03 | 2.46 |
| Aspartic acid | 557590±310569 | 270936±176898 | 2.67E-09 | 2.06 |
| Creatinine | 44542±24745 | 27791±17530 | 2.42E-05 | 1.60 |
| Homocysteine | 47960±54570 | 32093±36528 | 2.99E-02 | 1.49 |
| Aminomalonic acid | 270770±117301 | 200674±55521 | 6.10E-05 | 1.35 |
| Fucose | 962117±445301 | 731170±428855 | 2.47E-03 | 1.32 |
| Glycolic acid | 20019±5207 | 16019±4479 | 4.59E-07 | 1.25 |
| Glycine | 2092227±573273 | 1711043±433224 | 5.80E-07 | 1.22 |
| alpha-Tocopherol | 195224±61881 | 248754±92408 | 7.89E-04 | 0.78 |
| Pyruvic acid | 577790±163435 | 745964±166491 | 3.12E-08 | 0.77 |
| Cysteine | 126684±42619 | 172644±40780 | 2.67E-09 | 0.73 |
| Glycerol 1-hexadecanoate | 21587±12361 | 32220±11092 | 6.02E-08 | 0.67 |

**Supplementary Table S12. Differential metabolites between stomach adenocarcinoma (STAD) patients and healthy controls (HC) in Center 1.**

|  | **STAD (n=185)** | **HC (n=159)** | **FDR** | **FC** |
| --- | --- | --- | --- | --- |
| Male, n (%) | 129 (69.73) | 99 (62.26) | 0.222 |  |
| Age (years) | 66±9 | 66±11 | 0.485 |  |
| Hypoxanthine | 28711±12741 | 5437±6916 | 3.84E-44 | 5.28 |
| 3-Hydroxybutyric acid | 729295±1443463 | 168710±188603 | 5.11E-11 | 4.32 |
| Aspartic acid | 513093±398525 | 254705±182997 | 1.27E-09 | 2.01 |
| Homocysteine | 47517±51193 | 29944±40334 | 1.85E-07 | 1.59 |
| Creatinine | 48524±25385 | 30809±18449 | 3.03E-11 | 1.57 |
| Fucose | 991452±538409 | 731394±394141 | 9.75E-06 | 1.36 |
| Glycolic acid | 22274±8040 | 16454±4458 | 1.07E-19 | 1.35 |
| Aminomalonic acid | 264174±100784 | 209333±72729 | 2.16E-08 | 1.26 |
| Alpha-Tocopherol | 193924±63099 | 245664±92110 | 4.32E-09 | 0.79 |
| Pyruvic acid | 588786±175814 | 777171±180386 | 2.10E-18 | 0.76 |
| Glycerol 1-hexadecanoate | 23200±12420 | 31522±11825 | 2.19E-09 | 0.74 |
| Cysteine | 114143±37804 | 168017±38726 | 2.61E-26 | 0.68 |

**Supplementary Table S13. Differential metabolites between colon adenocarcinoma (COAD) patients and healthy controls (HC) in Center 1.**

|  | **COAD (n=98)** | **HC (n=130)** | **FDR** | **FC** |
| --- | --- | --- | --- | --- |
| Male, n (%) | 56 (57.14) | 84 (64.62) | 0.315 |  |
| Age (years) | 67±10 | 66±9 | 0.296 |  |
| Hypoxanthine | 31747±12377 | 5687±7343 | 2.54E-30 | 5.58 |
| 3-Hydroxybutyric acid | 424269±587211 | 156225±156005 | 3.38E-04 | 2.72 |
| Aspartic acid | 491797±387085 | 243917±185993 | 3.28E-05 | 2.02 |
| Creatinine | 45007±22879 | 31877±19296 | 4.14E-05 | 1.41 |
| Glycolic acid | 23106±6554 | 16563±4233 | 9.45E-17 | 1.40 |
| Aminomalonic acid | 287003±124805 | 219761±89004 | 3.22E-06 | 1.31 |
| Glycerol 1-hexadecanoate | 24747±13070 | 32403±11829 | 5.27E-05 | 0.76 |
| Pyruvic acid | 563310±143678 | 778436±173074 | 1.17E-17 | 0.72 |
| Cysteine | 113147±32872 | 168460±37549 | 4.48E-20 | 0.67 |

**Supplementary Table S14. Differential metabolites between rectum adenocarcinoma (READ) patients and healthy controls (HC) in Center 1.**

|  | **READ (n=154)** | **HC (n=130)** | **FDR** | **FC** |
| --- | --- | --- | --- | --- |
| Male, n (%) | 95 (61.69) | 84 (64.62) | 0.679 |  |
| Age (years) | 66±11 | 66±9 | 0.515 |  |
| Hypoxanthine | 33566±14001 | 5687±7343 | 4.50E-39 | 5.9 |
| Aspartic acid | 515274±355102 | 243917±185993 | 1.61E-10 | 2.11 |
| Glycolic acid | 22622±6923 | 16563±4233 | 1.09E-17 | 1.37 |
| Homocysteine | 46504±47569 | 35705±41736 | 8.04E-04 | 1.3 |
| Aminomalonic acid | 283422±110326 | 219761±89004 | 1.48E-07 | 1.29 |
| Fucose | 930166±441064 | 762976±401980 | 2.01E-03 | 1.22 |
| Glycerol 1-hexadecanoate | 23727±11067 | 32403±11829 | 2.75E-08 | 0.73 |
| Cysteine | 122964±37522 | 168460±37549 | 1.49E-18 | 0.73 |
| Pyruvic acid | 567308±124392 | 778436±173074 | 7.29E-23 | 0.73 |

**Supplementary Table S15. Differential metabolites between liver hepatocellular carcinoma (LIHC) patients and healthy controls (HC) in Center 1.**

|  | **LIHC (n=13)** | **HC (n=13)** | **FDR** | **FC** |
| --- | --- | --- | --- | --- |
| Male, n (%) | 8 (61.54) | 9 (69.23) | 1 |  |
| Age (years) | 68±9 | 68±9 | 0.914 |  |
| Hypoxanthine | 37668±25078 | 3821±3259 | 6.80E-05 | 9.86 |
| Homocysteine | 48416±35652 | 14266±20720 | 4.73E-02 | 3.39 |
| Methionine | 328195±470117 | 130983±40055 | 9.96E-03 | 2.51 |
| Glutamic acid | 809106±387182 | 459694±189589 | 4.73E-02 | 1.76 |
| Tyrosine | 1690395±649613 | 1089831±180763 | 1.68E-02 | 1.55 |
| Cysteine | 101022±28653 | 163162±60178 | 4.73E-02 | 0.62 |
| Pyruvic acid | 501934±128574 | 826647±142977 | 1.94E-05 | 0.61 |

**Supplementary Table S16. Differential metabolites between pancreatic adenocarcinoma (PAAD) patients and healthy controls (HC) in Center 1.**

|  | **PAAD (n=11)** | **HC (n=11)** | **FDR** | **FC** |
| --- | --- | --- | --- | --- |
| Male, n (%) | 9 (81.82) | 9 (81.82) | 1 |  |
| Age (years) | 62±11 | 65±9 | 0.951 |  |
| Hypoxanthine | 38739±16289 | 4299±4085 | 2.86E-04 | 9.01 |
| Glyceric acid | 18250±3838 | 13320±2231 | 2.75E-02 | 1.37 |
| Cysteine | 123303±41218 | 173584±39920 | 2.02E-02 | 0.71 |
| Pyruvic acid | 537665±120809 | 835047±217330 | 9.95E-03 | 0.64 |

**Supplementary Table S17. Differential metabolites between cervical squamous cell carcinoma and endocervical adenocarcinoma (CESC) patients and healthy controls (HC) in Center 1.**

|  | **CESC (n=61)** | **HC (n=61)** | **FDR** | **FC** |
| --- | --- | --- | --- | --- |
| Female, n (%) | 61 (100.00) | 61 (100.00) | 1 |  |
| Age (years) | 57±11 | 58±10 | 0.963 |  |
| Hypoxanthine | 26260±12762 | 6313±6701 | 7.98E-14 | 4.16 |
| Aspartic acid | 550370±357989 | 239342±181319 | 2.59E-07 | 2.30 |
| Cystine | 64062±33395 | 43580±19795 | 9.47E-04 | 1.47 |
| Fucose | 976121±397184 | 707163±299633 | 1.25E-04 | 1.38 |
| Aminomalonic acid | 294010±113459 | 221367±89001 | 5.25E-04 | 1.33 |
| Glycolic acid | 21860±7539 | 16931±4509 | 3.42E-04 | 1.29 |
| Pyruvic acid | 687064±169470 | 862286±183359 | 9.96E-06 | 0.79 |
| Glycerol 1-hexadecanoate | 22510±10301 | 30649±11772 | 5.38E-04 | 0.73 |
| Cysteine | 116402±32821 | 174686±31493 | 1.89E-12 | 0.67 |

**Supplementary Table S18. Differential metabolites between uterine corpus endometrial carcinoma (UCEC) patients and healthy controls (HC) in Center 1.**

|  | **UCEC (n=31)** | **HC (n=31)** | **FDR** | **FC** |
| --- | --- | --- | --- | --- |
| Female, n (%) | 31 (100.00) | 31 (100.00) | 1 |  |
| Age (years) | 57±12 | 58±11 | 0.865 |  |
| Hypoxanthine | 26103±14048 | 5065±3727 | 3.29E-10 | 5.15 |
| Aspartic acid | 500979±360440 | 213534±197565 | 9.00E-03 | 2.35 |
| Homocysteine | 58292±88998 | 31209±54642 | 9.00E-03 | 1.87 |
| Cystine | 60298±30397 | 36106±17389 | 9.00E-03 | 1.67 |
| Pyruvic acid | 681592±170076 | 857880±196365 | 5.75E-03 | 0.79 |
| Glycerol 1-hexadecanoate | 22548±14636 | 31864±13756 | 2.73E-02 | 0.71 |
| Cysteine | 120859±39228 | 174693±35514 | 5.02E-05 | 0.69 |

**Supplementary Table S19. Differential metabolites between ovarian carcinoma (OC) patients and healthy controls (HC) in Center 1.**

|  | **OC (n=14)** | **HC (n=14)** | **FDR** | **FC** |
| --- | --- | --- | --- | --- |
| Female, n (%) | 14 (100.00) | 14 (100.00) | 1 |  |
| Age (years) | 59±12 | 60±12 | 0.860 |  |
| Hypoxanthine | 32113±18322 | 6950±9700 | 3.38E-03 | 4.62 |
| Glycolic acid | 25076±9910 | 15768±2729 | 2.37E-02 | 1.59 |
| Pyruvic acid | 667340±250821 | 838158±175403 | 3.55E-02 | 0.79 |
| Citric acid | 726362±154092 | 967048±150119 | 3.38E-03 | 0.75 |
| Cysteine | 105186±23621 | 162733±36494 | 5.37E-03 | 0.65 |

**Supplementary Table S20. Differential metabolites between prostate adenocarcinoma (PRAD) patients and healthy controls (HC) in Center 1.**

|  | **PRAD (n=32)** | **HC (n=32)** | **FDR** | **FC** |
| --- | --- | --- | --- | --- |
| Male, n (%) | 32 (100.00) | 32 (100.00) | 1 |  |
| Age (years) | 71±6 | 71±6 | 0.697 |  |
| Hypoxanthine | 30803±19229 | 4373±6667 | 1.97E-10 | 7.04 |
| Fucose | 1096964±495158 | 683815±418201 | 9.09E-03 | 1.60 |
| Creatinine | 38651±21945 | 25384±14951 | 3.50E-02 | 1.52 |
| Glycolic acid | 22966±5647 | 15764±4421 | 1.24E-05 | 1.46 |
| Aminomalonic acid | 257187±68466 | 188082±63275 | 1.20E-04 | 1.37 |
| Glycine | 2081510±571492 | 1708610±580881 | 1.19E-02 | 1.22 |
| Pyruvic acid | 575977±156138 | 733292±128222 | 5.73E-04 | 0.79 |
| Palmitic acid | 774212±219399 | 992810±206894 | 9.23E-04 | 0.78 |
| Cysteine | 124212±34723 | 163828±38860 | 5.73E-04 | 0.76 |
| Glycerol 1-hexadecanoate | 24529±10327 | 33184±11268 | 1.72E-02 | 0.74 |
| Linoleic acid | 201014±77065 | 281037±64872 | 3.89E-04 | 0.72 |
| alpha-Tocopherol | 174945±51334 | 252861±85117 | 2.38E-04 | 0.69 |

**Supplementary Table S21. Differential metabolites between kidney renal clear cell carcinoma (KIRC) patients and healthy controls (HC) in Center 1.**

|  | **KIRC (n=45)** | **HC (n=45)** | **FDR** | **FC** |
| --- | --- | --- | --- | --- |
| Male, n (%) | 30 (66.67) | 33 (73.33) | 0.705 |  |
| Age (years) | 63±10 | 62±10 | 0.466 |  |
| Hypoxanthine | 29611±14526 | 5064±5385 | 5.83E-17 | 5.85 |
| Homocysteine | 60975±62459 | 28277±39262 | 1.31E-03 | 2.16 |
| Aspartic acid | 554590±326324 | 278415±192753 | 1.62E-04 | 1.99 |
| Creatinine | 42850±29884 | 29087±18257 | 4.35E-02 | 1.47 |
| Glycolic acid | 22498±8280 | 16490±4134 | 1.62E-04 | 1.36 |
| Palmitic acid | 772644±191654 | 980143±184715 | 3.95E-05 | 0.79 |
| Pyruvic acid | 621280±163426 | 800273±174953 | 3.38E-05 | 0.78 |
| Cysteine | 124976±38303 | 161731±42195 | 2.25E-04 | 0.77 |
| Oleic acid | 340043±186425 | 453700±209880 | 8.46E-03 | 0.75 |
| Linoleic acid | 204916±71719 | 274135±69590 | 1.28E-04 | 0.75 |
| Glycerol 1-hexadecanoate | 20865±9226 | 29266±13102 | 1.17E-02 | 0.71 |

**Supplementary Table S22. Differential metabolites between bladder urothelial carcinoma (BLCA) patients and healthy controls (HC) in Center 1.**

|  | **BLCA (n=83)** | **HC (n=83)** | **FDR** | **FC** |
| --- | --- | --- | --- | --- |
| Male, n (%) | 63 (75.90) | 65 (78.31) | 0.595 |  |
| Age (years) | 69±8 | 68±9 | 0.794 |  |
| Hypoxanthine | 28074±12371 | 6196±7263 | 3.85E-20 | 4.53 |
| Aspartic acid | 501456±391494 | 260389±189233 | 3.69E-04 | 1.93 |
| Creatinine | 43320±23319 | 30618±19149 | 1.56E-03 | 1.41 |
| Glycolic acid | 23069±7211 | 16468±4693 | 1.29E-10 | 1.40 |
| Aminomalonic acid | 275912±118293 | 199686±57132 | 1.45E-05 | 1.38 |
| Glycine | 2127649±676110 | 1697720±421828 | 1.93E-06 | 1.25 |
| Fucose | 916538±421390 | 731668±444904 | 1.26E-02 | 1.25 |
| Urea | 4286361±1268732 | 3544483±1077119 | 3.69E-04 | 1.21 |
| Palmitic acid | 804249±218513 | 1023680±224778 | 2.12E-08 | 0.79 |
| Pyruvic acid | 626901±139444 | 798741±179447 | 2.85E-08 | 0.78 |
| Alpha-Tocopherol | 191128±58395 | 246814±92030 | 3.06E-05 | 0.77 |
| Glycerol 1-hexadecanoate | 24468±11596 | 32452±11582 | 2.40E-04 | 0.75 |
| Cysteine | 126244±39797 | 169801±39200 | 5.85E-09 | 0.74 |
| Linoleic acid | 212672±75775 | 291110±80316 | 1.67E-08 | 0.73 |
| 3-Hydroxyisobutyric acid | 19698±5663 | 28696±28752 | 5.83E-03 | 0.69 |

**Supplementary Table S23. Differential metabolites between lung adenocarcinoma (LUAD) patients and healthy controls (HC) in Center 2.**

|  | **LUAD (n=489)** | **HC (n=208)** | **FDR** | **FC** |
| --- | --- | --- | --- | --- |
| Male, n (%) | 212 (43.35) | 91 (43.75) | 1 |  |
| Age (years) | 60±11 | 53±15 | 0.001 |  |
| Hypoxanthine | 47798±67246 | 2138±1386 | 7.95E-92 | 22.36 |
| Creatinine | 48649±15836 | 30781±13006 | 2.06E-36 | 1.58 |
| Aminomalonic acid | 415734±153929 | 295266±125721 | 6.67E-22 | 1.41 |
| Cystine | 10260±7850 | 7902±6629 | 2.41E-05 | 1.3 |
| Glucose | 1044847±622289 | 840617±338874 | 1.04E-07 | 1.24 |
| Oxalic acid | 301458±121439 | 245659±68036 | 1.88E-07 | 1.23 |
| Ornithine | 92026±65563 | 115992±60253 | 2.61E-09 | 0.79 |
| Cysteine | 261853±6269 | 28018±5541 | 7.84E-05 | 0.78 |
| gamma-Butyrolactone | 55913±24947 | 72454±24283 | 5.65E-14 | 0.77 |
| Monopalmitin | 16150±3924 | 20881±5165 | 1.12E-27 | 0.77 |
| Pyruvic acid | 37722±57481 | 49636±16411 | 5.00E-21 | 0.76 |
| Glutamine | 894024±280301 | 1192736±364171 | 2.21E-24 | 0.75 |
| alpha-Tocopherol | 91328±40670 | 129852±38273 | 1.23E-29 | 0.7 |
| Asparagine | 20253±8015 | 29805±11531 | 1.31E-27 | 0.68 |
| Malic acid | 6748±3857 | 10030±2424 | 4.25E-46 | 0.67 |
| 3-Hydroxybutyric acid | 62238±65083 | 95365±111053 | 2.13E-07 | 0.65 |
| Succinic acid | 4158±2102 | 6512±1604 | 2.58E-52 | 0.64 |

**Supplementary Table S24. Differential metabolites between lung squamous carcinoma (LUSC) patients and healthy controls (HC) in Center 2.**

|  | **LUSC (n=26)** | **HC (n=26)** | **FDR** | **FC** |
| --- | --- | --- | --- | --- |
| Male, n (%) | 23 (88.46) | 14 (53.85) | 0.014 |  |
| Age (years) | 64±10 | 65±17 | 0.515 |  |
| Hypoxanthine | 34881±25926 | 2396±1514 | 4.21E-12 | 14.56 |
| Pelargonic acid | 11858±6660 | 5895±6710 | 4.45E-04 | 2.01 |
| Arachidonic acid | 30731±12026 | 16367±5210 | 5.03E-06 | 1.88 |
| Oxalic acid | 400797±121236 | 261224±54775 | 6.05E-05 | 1.53 |
| Monomethylphosphate | 48480±18697 | 37106±11578 | 3.33E-02 | 1.31 |
| Glyceric acid | 10365±3075 | 8245±1144 | 1.27E-02 | 1.26 |
| Succinic acid | 5045±1817 | 6346±1126 | 3.80E-03 | 0.79 |
| Lactic acid | 2151949±692093 | 2743151±489561 | 1.32E-03 | 0.78 |
| Leucine | 661509±153431 | 847789±268866 | 1.27E-02 | 0.78 |
| Linoleic acid | 124685±64154 | 158939±36161 | 5.10E-03 | 0.78 |
| Cysteine | 22360±6417 | 29416±3696 | 6.28E-05 | 0.76 |
| Monopalmitin | 15266±3015 | 20662±4584 | 9.61E-05 | 0.74 |
| Methionine | 41115±6559 | 57386±20570 | 5.45E-04 | 0.72 |
| Isoleucine | 253725±120422 | 357078±118032 | 2.04E-02 | 0.71 |
| Valine | 155681±33047 | 219019±70635 | 1.65E-03 | 0.71 |
| Urea | 60911±16400 | 87363±40678 | 2.44E-03 | 0.7 |
| Alanine | 1671085±432993 | 2472475±774132 | 1.21E-04 | 0.68 |
| Pyrophosphate | 84565±14509 | 126436±27905 | 2.78E-06 | 0.67 |
| Serine | 238748±44966 | 358614±110412 | 1.08E-05 | 0.67 |
| myo-Inositol | 44523±12061 | 67789±29997 | 9.10E-04 | 0.66 |
| 3-Hydroxybutyric acid | 78097±122179 | 119278±127507 | 1.37E-03 | 0.65 |
| Citric acid | 370799±120004 | 567767±123604 | 1.72E-06 | 0.65 |
| Malic acid | 6829±2776 | 10445±2619 | 3.77E-05 | 0.65 |
| Palmitelaidic acid | 10359±12775 | 16980±6187 | 1.38E-04 | 0.61 |
| Glycine | 59890±19209 | 101073±34043 | 7.30E-06 | 0.59 |
| Lysine | 22606±7408 | 39367±16111 | 1.05E-05 | 0.57 |
| Glutamine | 654839±132204 | 1162736±320840 | 1.63E-09 | 0.56 |
| Asparagine | 16073±2858 | 29570±11389 | 1.08E-08 | 0.54 |
| alpha-Tocopherol | 72132±48300 | 134922±37608 | 5.01E-06 | 0.53 |
| Pyruvic acid | 34005±23302 | 67738±20256 | 3.93E-07 | 0.5 |
| Ornithine | 60298±37923 | 126662±68197 | 4.78E-05 | 0.48 |
| Fructose | 34792±38732 | 90120±27132 | 2.41E-05 | 0.39 |

**Supplementary Table S25. Differential metabolites between esophageal carcinoma (ESCA) patients and healthy controls (HC) in Center 2.**

|  | **ESCA (n=330)** | **HC (n=252)** | **FDR** | **FC** |
| --- | --- | --- | --- | --- |
| Male, n (%) | 264 (80.00) | 137 (54.36) | 1.26E-08 |  |
| Age (years) | 64±7 | 49±17 | 3.09E-09 |  |
| Hypoxanthine | 77977±117498 | 2071±1335 | 8.55E-89 | 37.64 |
| Glucose | 1366282±837391 | 821730±332354 | 4.52E-24 | 1.66 |
| Oxalic acid | 363667±118697 | 245110±67518 | 6.47E-36 | 1.48 |
| Creatinine | 43926±15373 | 30714±12923 | 1.26E-22 | 1.43 |
| Aminomalonic acid | 411711±169583 | 293386±122635 | 2.71E-19 | 1.4 |
| Glyceric acid | 10867±6442 | 8444±1735 | 1.01E-07 | 1.29 |
| Cystine | 9722±8356 | 7815±6314 | 1.71E-03 | 1.24 |
| Monopalmitin | 16159±3739 | 20612±5031 | 9.94E-28 | 0.78 |
| Serine | 286552±86578 | 367479±108173 | 1.51E-23 | 0.78 |
| Pyrophosphate | 100925±33438 | 132496±41544 | 1.39E-25 | 0.76 |
| Cysteine | 20865±7151 | 27455±5667 | 3.34E-16 | 0.76 |
| Alanine | 1735358±583405 | 2301224±776641 | 2.92E-20 | 0.75 |
| Pyruvic acid | 36554±64359 | 49150±15915 | 3.85E-33 | 0.74 |
| Linoleic acid | 100614±43528 | 137336±40465 | 4.46E-25 | 0.73 |
| Succinic acid | 4697±2072 | 6432±1604 | 1.46E-37 | 0.73 |
| Malic acid | 7061±3819 | 9832±2425 | 5.61E-36 | 0.72 |
| Glutamine | 841693±236805 | 1185991±355699 | 1.07E-33 | 0.71 |
| Ornithine | 76645±42433 | 112375±60052 | 1.05E-15 | 0.68 |
| Proline | 372747±257123 | 550629±389217 | 2.28E-10 | 0.68 |
| Asparagine | 18556±6792 | 29836±11193 | 5.28E-39 | 0.62 |
| alpha-Tocopherol | 66803±39099 | 127104±36188 | 4.87E-58 | 0.53 |

**Supplementary Table S26. Differential metabolites between adenocarcinoma of esophagogastric junction (AEG) patients and healthy controls (HC) in Center 2.**

|  | **AEG (n=58)** | **HC (n=60)** | **FDR** | **FC** |
| --- | --- | --- | --- | --- |
| Male, n (%) | 39 (67.24) | 36 (60.00) | 0.531 |  |
| Age (years) | 65±8 | 63±15 | 0.483 |  |
| Hypoxanthine | 83886±139767 | 2139±1456 | 1.74E-18 | 39.21 |
| Cystine | 12786±7476 | 7857±6059 | 9.41E-05 | 1.63 |
| Oxalic acid | 392029±153779 | 259006±57504 | 1.94E-07 | 1.51 |
| Aminomalonic acid | 464047±128753 | 323712±114215 | 2.52E-07 | 1.43 |
| Glucose | 1291896±505429 | 907154±372921 | 1.35E-05 | 1.42 |
| Creatinine | 48202±17106 | 34581±12216 | 2.22E-05 | 1.39 |
| Cysteine | 22864±7433 | 28942±4910 | 0.000623 | 0.79 |
| Ornithine | 88004±46771 | 114610±62150 | 0.022299 | 0.77 |
| Palmitic acid | 405581±105051 | 528936±108967 | 3.02E-07 | 0.77 |
| Alanine | 1801663±615183 | 2374709±906087 | 0.000435 | 0.76 |
| Glutamine | 863524±176656 | 1156298±336129 | 3.02E-07 | 0.75 |
| Monopalmitin | 15106±3100 | 20070±4072 | 8.23E-09 | 0.75 |
| myo-Inositol | 46613±13223 | 62796±27428 | 3.55E-05 | 0.74 |
| Pyrophosphate | 94834±32063 | 129452±28279 | 7.88E-08 | 0.73 |
| Pyruvic acid | 42671±28395 | 59570±20412 | 1.08E-05 | 0.72 |
| Asparagine | 19828±6935 | 28432±11024 | 6.75E-06 | 0.7 |
| Proline | 397796±252438 | 574138±380444 | 0.016669 | 0.69 |
| Malic acid | 6664±2844 | 9957±2446 | 2.7E-08 | 0.67 |
| Succinic acid | 4156±1456 | 6166±1559 | 1.69E-09 | 0.67 |
| Linoleic acid | 90628±32268 | 150720±41624 | 1.08E-10 | 0.6 |
| alpha-Tocopherol | 68457±38430 | 135362±34980 | 1.53E-14 | 0.51 |

**Supplementary Table S27. Differential metabolites between stomach adenocarcinoma (STAD) patients and healthy controls (HC) in Center 2.**

|  | **STAD (n=15)** | **HC (n=15)** | **FDR** | **FC** |
| --- | --- | --- | --- | --- |
| Male, n (%) | 10 (66.67) | 9 (60.00) | 1 |  |
| Age (years) | 65±9 | 64±10 | 0.740 |  |
| Hypoxanthine | 58719±48326 | 2495±1589 | 4.96E-06 | 23.53 |
| Oxalic acid | 427270±150656 | 245267±52177 | 1.62E-04 | 1.74 |
| Glucose | 1338519±824072 | 814406±247796 | 7.55E-03 | 1.64 |
| Aminomalonic acid | 478271±144568 | 309549±100253 | 5.68E-03 | 1.55 |
| Creatinine | 51378±14131 | 36078±9303 | 9.78E-03 | 1.42 |
| Leucine | 745575±122428 | 946078±255227 | 3.35E-02 | 0.79 |
| Serine | 303299±63669 | 384336±80054 | 1.66E-02 | 0.79 |
| Pyruvic acid | 52361±60221 | 66554±11661 | 1.83E-02 | 0.79 |
| Cysteine | 23443±8796 | 30055±3823 | 4.31E-02 | 0.78 |
| Pyroglutamic acid | 353548±189129 | 462815±83362 | 4.08E-03 | 0.76 |
| Valine | 183052±29530 | 239692±59401 | 5.68E-03 | 0.76 |
| Monopalmitin | 16412±3704 | 22321±4480 | 4.56E-03 | 0.74 |
| Palmitic acid | 404103±90593 | 549775±93438 | 2.44E-03 | 0.74 |
| Tyrosine | 346775±97739 | 474717±145800 | 7.08E-03 | 0.73 |
| Aspartic acid | 19267±10435 | 26873±7566 | 7.08E-03 | 0.72 |
| myo-Inositol | 46155±10357 | 64941±19064 | 3.65E-03 | 0.71 |
| Glutamic acid | 162137±80709 | 241278±122594 | 4.11E-02 | 0.67 |
| Lactic acid | 1950292±951339 | 2928899±530636 | 2.19E-03 | 0.67 |
| Glutamine | 863379±131690 | 1307400±287882 | 2.43E-04 | 0.66 |
| Pyrophosphate | 85988±25959 | 129908±18042 | 2.43E-04 | 0.66 |
| Succinic acid | 4270±2167 | 6446±1322 | 1.62E-03 | 0.66 |
| Malic acid | 7119±5746 | 10900±3211 | 8.58E-04 | 0.65 |
| Alanine | 1685699±502460 | 2767938±726822 | 4.41E-04 | 0.61 |
| Linoleic acid | 95978±32773 | 156614±39986 | 8.58E-04 | 0.61 |
| Asparagine | 19107±4053 | 33601±8838 | 1.62E-04 | 0.57 |
| Ornithine | 84071±28900 | 147002±49983 | 9.58E-04 | 0.57 |
| alpha-Tocopherol | 78186±40613 | 146399±38286 | 1.62E-04 | 0.53 |

**Supplementary Table S28. Sample information of TCGA RNA-Seq datasets for transcriptomics analysis.**

| **Site** | **Solid cancer type (n=21)** | **Abbreviation** |  | **Transcriptomics Analysis (tissue)** | | |
| --- | --- | --- | --- | --- | --- | --- |
|  |  |  |  | **Tumor** | **Adjacent tissue** | **Total samples** |
| Bladder | Bladder Urothelial Carcinoma | BLCA |  | 406 | 19 | 425 |
| Breast | Breast invasive carcinoma | BRCA |  | 917 | 83 | 1000 |
| Cervix | Cervical squamous cell carcinoma | CSCC |  | 296 | 3 | 299 |
|  | Endocervical adenocarcinoma | ECAC |  |  |  |  |
| Bile duct | Cholangiocarcinoma | CHOL |  | 35 | 9 | 44 |
| Colon | Colon adenocarcinoma | COAD |  | 465 | 41 | 506 |
| Esophagus | Esophageal carcinoma | ESCA |  | 173 | 13 | 186 |
| Kidney | Kidney renal clear cell carcinoma | KIRC |  | 533 | 72 | 605 |
| Liver | Liver hepatocellular carcinoma | LIHC |  | 369 | 50 | 419 |
| Head and neck | Laryngeal squamous cell carcinoma | LSCC |  | 369 | 50 | 419 |
|  | Nasopharyngeal carcinoma | NPC |  |  |  |  |
| Lung | Lung adenocarcinoma | LUAD |  | 524 | 58 | 582 |
|  | Lung squamous cell carcinoma | LUSC |  | 497 | 51 | 548 |
|  | Small-cell lung cancer | SCLC |  | / | / | / |
| Ovary | Ovarian adenocarcinoma | OV |  | 400 | / | 400 |
| Pancreas | Pancreatic adenocarcinoma | PAAD |  | 178 | 4 | 182 |
| Prostate | Prostate adenocarcinoma | PRAD |  | 483 | 51 | 534 |
| Rectum | Rectum adenocarcinoma | READ |  | 163 | 10 | 173 |
| Stomach | Stomach adenocarcinoma | STAD |  | 410 | 36 | 446 |
| Thyroid | Thyroid carcinoma | THCA |  | 500 | 57 | 557 |
| Uterus | Uterine Corpus Endometrial Carcinoma | UCEC |  | 543 | 35 | 578 |
